# Large-scale functional RNAi screen in *C. elegans *identifies genes that regulate the dysfunction of mutant polyglutamine neurons

**DOI:** 10.1186/1471-2164-13-91

**Published:** 2012-03-13

**Authors:** François-Xavier Lejeune, Lilia Mesrob, Frédéric Parmentier, Cedric Bicep, Rafael P Vazquez-Manrique, J Alex Parker, Jean-Philippe Vert, Cendrine Tourette, Christian Neri

**Affiliations:** 1 INSERM, Unit 894, Laboratory of Neuronal Cell Biology and Pathology, 75014 Paris, France; 2 University of Paris Descartes, EA 4059, 75014 Paris, France; 3 Université de Montreal, CRCHUM Centre d'excellence en neuromique, Hôpital Notre-Dame, Montreal, QC,H2W 1T8 Canada; 4 Mines ParisTech, CBIO, Fontainebleau, 75006 Paris, France; 5 Curie Institute, Research Center, 75005 Paris, France; 6 INSERM, Unit 900, Paris, 75005 France; 7 Buck Institute, Novato, CA 94945, USA

## Abstract

**Background:**

A central goal in Huntington's disease (HD) research is to identify and prioritize candidate targets for neuroprotective intervention, which requires genome-scale information on the modifiers of early-stage neuron injury in HD.

**Results:**

Here, we performed a large-scale RNA interference screen in *C. elegans *strains that express N-terminal huntingtin (htt) in touch receptor neurons. These neurons control the response to light touch. Their function is strongly impaired by expanded polyglutamines (128Q) as shown by the nearly complete loss of touch response in adult animals, providing an *in vivo *model in which to manipulate the early phases of expanded-polyQ neurotoxicity. In total, 6034 genes were examined, revealing 662 gene inactivations that either reduce or aggravate defective touch response in 128Q animals. Several genes were previously implicated in HD or neurodegenerative disease, suggesting that this screen has effectively identified candidate targets for HD. Network-based analysis emphasized a subset of high-confidence modifier genes in pathways of interest in HD including metabolic, neurodevelopmental and pro-survival pathways. Finally, 49 modifiers of 128Q-neuron dysfunction that are dysregulated in the striatum of either R/2 or CHL2 HD mice, or both, were identified.

**Conclusions:**

Collectively, these results highlight the relevance to HD pathogenesis, providing novel information on the potential therapeutic targets for neuroprotection in HD.

## Background

Huntington's disease (HD) is a dominantly-inherited disorder caused by expanded polyglutamine (polyQ) tracts in the N-terminal portion of huntingtin (htt) and characterized by striatal and cortical degeneration [1]. While HD pathogenesis might involve a gain of toxic properties by mutant htt as well as a loss of normal htt function, several studies have emphasized a critical role of misfolded N-terminal fragments of mutant htt [2,3] that are natural products of htt processing [4]. Huntingtin is thought to have a large number of partner proteins involved in a variety of biological processes [5-8], suggesting that polyglutamine expansion in htt may alter several biological processes that are essential to cellular homeostasis and neuron survival. Consistent with this possibility, transcriptomic analyses have revealed that mutant/polyQ-expanded htt expression may change the expression of a large number of genes in the caudate nucleus of HD subjects [9] and in the brain of fragment, knock-in and full-length mouse models of HD [10]. Given the complexity of alterations in cells and tissues expressing expanded polyQ/htt, gene perturbation screens are expected to shed light on the mechanisms that may be critical for cell response to mutant polyQ expression. Additionally, the integration of gene perturbation data with other types of 'omics data such as for example gene expression profiles in HD mice [10] might fulfill the need for target profiling and prioritisation in HD. This may be particularly important when comparing target gene activity for the regulation of neuronal dysfunction versus that of neuronal death. Before the occurrence of neuronal cell loss, neuron dysfunction is indeed thought to be an early-stage component of the pathogenic process in HD [3,11]. However, neuronal cell dysfunction is not easily tractable *in vivo*. Interestingly, the genetic regulation of response to light touch by mechanosensory neurons in *C. elegans *has been the subject of detailed analysis [12]. By targeting polyQ expression to *C. elegans *touch cells, we previously generated a model that recapitulates an early phase of expanded polyQ neurotoxicity, namely neuronal dysfunction in the absence of cell death [13,14]. In these animals, exon 1 like htt fused to CFP was expressed in touch receptor neurons under the control of the promoter for the *mec-3 *gene (*mec-3p*) [14]. This promoter is active from the early stages of embryonic development through adulthood [15]. Young adults expressing normal polyQs (19Q) have a moderate loss of touch response with no or little change in touch cell morphology, whereas young adults expressing expanded polyQs (128Q) show a dramatic loss of touch response, a behavioral phenotype that is accompanied by a strong level of axonal dystrophy [14]. Axonal dystrophy is followed by late-onset axonal degeneration as observed in older adult animals (unpublished data). The loss of touch response in expanded-polyQ nematodes can be manipulated by genetic means, as exemplified by the protective effect of sirtuin overexpression and aggravating effect of loss-of-function in the *sir-2.1*/SIRT1--*daf-16/*FOXO cell survival pathway [14]. These animals thus provide an *in vivo *model in which to screen for modifiers of neuron dysfunction induced by expanded polyQs. Here, we used 128Q nematodes in a large-scale functional RNA interference (RNAi) screen for modulation of response to touch. Additionally, we used 19Q nematodes to test a subset of the 128Q-confirmed hits for modulation of response to touch in 19Q nematodes. To this end, we generated polyQ strains with enhanced response of touch receptor neurons to double-standed RNA delivered by feeding. We then undertook a semi-directed screening approach to test a total of 6034 genes including 5128 genes tested at random and 906 genes that belong to major signaling pathways (16-19). This led us to identify 662 genes that either reduce or aggravate the loss of response to light touch when knocked-down by RNAi in 128Q nematodes. Screening in 19Q nematodes suggested that greater than 90% of these genes specifically modify 128Q cytotoxicity. In-depth data analysis emphasized a role for cell differentiation/survival, metabolic and neurodegenerative disease pathways in the modulation of expanded-polyQ cytotoxicity. Additionally, data analysis and comparisons to gene expression data from HD mice emphasized the potential value of this RNAi dataset for target discovery in HD.

## Methods

### *C. elegans *strains

Nematode strains were handled using standard methods [16]. Integrated polyQ strains used in this study were previously described [17,18]. Briefly, ID245 (*igIs245[mec-3p::htt57-19Q::CFP; lin-15(+); mec-7p::YFP*]) and ID1 (*igIs1[mec-3p::htt57-128Q::CFP; lin-15(+); mec-7p::YFP*]) stably co-express the first 57 amino acids of human huntingtin (htt) fused to CFP under the control of the promoter of the *mec-3 *gene and YFP under the control of the promoter of the *mec-7 *gene. We previously reported that ID245 animals show a moderate loss of response to light touch at the tail whereas ID1 animals show a strong loss of response to light touch at the tail, the latter phenotype accompanied by axonal swelling [14]. The enhanced RNAi strain YY13 which carries the *rrf-3(mg373) *mutation [19] was provided by the Caenorhabditis Genetics Center, which is funded by the NIH National Center for Research Resources (NCRR). To construct polyQ strains with enhanced RNAi sensitivity in neurons, we crossed YY13 animals with either ID245 or ID1 animals to generate strain ID448 (*igIs245[mec-3p::htt57-19Q::CFP;lin-15(+);mec-7p::YFP*];*rrf-3(pk1426)*) and strain ID447 (*igIs1[mec-3p::htt57-128Q::CFP;lin-15(+);mec-7p::YFP*];*rrf-3(pk1426)*), respectively. The ID448 and ID447 strains were tested for sensitivity of touch receptor neurons to RNAi and subsequently used to screen the *C. elegans *ORF-RNAi library (Open Biosystems, AL) that includes clones from the *C. elegans *ORFeome library v1.1 [20] as detailed below.

### RNAi screen

Bacterial clones from the *C. elegans *ORF-RNAi library were grown in 500 μl of LB culture medium supplemented with ampicillin (100 μg/ml) and tetracyclin (12.5 μg/ml), and they were induced by adding 10 μl of IPTG (12.5 mg/ml) 4 hours prior to incubation with nematodes. Synchronized L1 larvae were obtained by hypochlorite extraction using standard methods [16] and they were grown at 20°C on Petri dishes until they reach the young adult stage. The day of incubation in 96-well plates (D0), two young adult animals were incubated per well. Each of the wells contained 33.6 μl of bacterial culture and 16.6 μl of M9 media supplemented with cholesterol, ampicillin, tetracyclin, fungizone and IPTG. Incubation was performed at 20°C during 4 days until the animals reached adulthood and produce late L4 larvae. To ensure appropriate feeding of the nematodes in the well, 16.6 μl of bacterial cultures expressing RNAi clones were added at day 1. At day 4, animals were transferred to agar plates and allowed to recover for 15 minutes. RNAi clones producing lethality, larval arrest, egg-laying defect, developmental delay or any other abnormalities such as morphological abnormalities (2243 RNAi clones) were excluded from further analysis. For RNAi clones that did not alter nematode growth and morphology, the two P0 animals (old and dark nematodes) initially incubated in the well were manually removed from the plate and young adults (F1 progeny) were assayed for light touch response as previously described [13]. Briefly, touch tests involved scoring for the response to light touch at the tail by using a fine hair. Touch test were performed by scoring 5 touches at the tail of the animal for 70-90 animals/RNAi clone/test. Ordinarily, wild-type animals will respond by backing away from the touch. The responses were recorded for every animal such that, for example, 4 responses out of 5 at the tail is given as 80% responsiveness, and the mean values for responsiveness were retained for further analysis of the data. A score for change in touch response (S) was calculated as [(Percent response - mean baseline)/mean baseline]. Using this formula, the maximally-achievable score is 4.55 (maximal suppression of 128Q cytotoxicity, *i.e*. 100% responsiveness) and the minimally-achievable score is -1 (maximal aggravation of 128Q cytotoxicity). The baseline in the assay was monitored across the entire time of the screening from the incubation of the animals treated with the empty vector L4440 in all of the 96-well plates assayed. The cumulated baseline over the entire time of the screening was observed to be stable (20.6 ± 3%) and the cumulated baseline/month was used to calculate S scores. Statistical analysis was performed using one-way ANOVA and the Tukey's Multiple Comparison Test. To decrease the risk for false positives, RNAi clones were scored as positive hits (suppressors or enhancers) if the S ± SD value was outside the baseline ± (SD*2.6). Initial hits (suppressors, enhancers) were tested for confirmation or invalidation of effect on touch response and the resulting data were retained for biological content analysis. All of the screening assays were performed by investigators that were trained to score touch response of 128Q animals in a similar way using blind assays.

Prior to performing the RNAi screen, the efficiency of gene inactivation in touch receptor neurons was tested by incubating ID447 L1 larvae in 96-well plates (1 larvae/well) with bacteria expressing either RNAi against GFP (plasmid pL4417) or empty vector (plasmid pL4440). Incubation was performed as described above until F1 young adults are produced. At the end of incubation, 70-90 animals/genotype were moved to agar plates and mounted on slides, and YFP levels in touch cells (*mec-7p *target cells) were measured at high (63X) magnification using a fluorescence microscope (Zeiss Axioplan Imaging II) and image analysis software (Image J). Additionally, YFP mRNA levels were measured by qRT-PCR from whole animals using standard methods. All of the nematode experiments were performed at least three times. Student's t-tests were used for statistical analysis of YFP mRNA levels and YFP expression levels.

### Data analysis

The final screening results were initially classified using Gene Ontology (GO) annotations for Biological Process, Molecular Function and Cellular Component http://www.geneontology.org/[21]. GO enrichment tests were performed using Ontologizer v 2.0 [22]. Additionally, we used hypergeometric tests to test for gene set enrichment [23,24]. The gene sets used for enrichment analysis were established using core components of (i) canonical pathways based on Wormbook http://www.wormbook.org, KEGG http://www.genome.ad.jp/kegg/[25] and Panther http://www.pantherdb.org/pathway/.

To analyze further the biological content of RNAi clones confirmed to modulate 128Q toxicity, we performed network-boosted data analysis. To this end, we subjected the RNAi data to spectral decomposition analysis using the integrated *C. elegans *network Wormnet v.2 [26]. This large-coverage network contains gene interaction data that were established according to several types of evidences derived from multiple organisms, including several types of gene interactions based on gene co-expression (microarray measurements of the expression of *C. elegans *mRNAs), protein-protein interactions including genome-wide yeast two-hybrid interactions between *C. elegans *proteins and protein-protein interactions from the Worm Interactome database [27], genetic interactions from WormBase [28], human and fly 'interologs' *i.e*. human and fly protein interactions [29,30], Yeast 'associalogs' *i.e*. functional interactions transferred from the yeast functional gene network [31], phylogenic profiles (comparative genomics linkages derived from the analysis of bacterial and archaeal genomes), gene neighbors and co-citation (literature-mined *C. elegans *gene associations). To increase the power of spectral decomposition analysis in detecting pathways, we also used a more homogenous network containing high confidence interactions, namely Core Wormnet [26] in which we omitted co-expression and co-citation links. To perform spectral decomposition analysis, we developed an enhanced version of the previously described [32] Fourier-like analysis. This approach is based on the spectral decomposition of gene activity profiles with respect to the connectivity of the reference network. S scores in the RNAi screen are considered as a 'signal' and the Fourier transformation decomposes the signal as a superposition of signals at different frequencies, with 40 successive attenuations performed [32]. The aim of these transformations is to keep the low-frequency signals and discard the high frequency signals, considered as noise, in the context of gene connectivity. The logic behind this procedure is that highly inter-connected genes may be implicated in the same pathway and may fluctuate in the same direction [32]. Starting from the raw RNAi screen data, we iteratively removed the high-frequency components 2.5% at a time, resulting in 40 increasingly smoothed versions of the data. Given a smoothed gene activity data, we considered modules as groups of connected genes with a signal > mean S-score + SD or < mean S-score-SD. Additionally, and to generate an even more relevant signal and robust information, we selected the modules that were present after the 4^th ^attenuation and that showed a stable gene content (≥ 40% of the genes with a value over the threshold) for at least 2 successive attenuations. The resulting modules were filtered for redundant gene content and functional content of the remaining modules analyzed using Ontologizer v 2.0 [22]. Additionally, we used hypergeometric tests to annotate for signaling pathways (gene lists extracted from Wormbook, KEGG and Panther), druggable genes [33], htt partners [7,34] and genes involved in autophagy, mitochondrial function and synaptic activity (gene lists kindly provided by the CHDI Foundation). Information on conservation in humans was derived from Inparanoid clusters [35]. In addition to tables included herein, the results of network-boosted data analysis are displayed in a freely-accessible database (see http://www.broca.inserm.fr/EHDN2/RNAiscreen) for the vizualisation of modules. This database allows the users to browse the results using GO/KEGG/Panther annotations and other annotations of interest, and it uses VisANT 3.5 for graph visualization [36]. We used confidence graphs for display of modules by normalizing the statistical weights for functional interactions as available in Wormnet v 2.0 [26].

## Results

### The touch receptor neurons of F1 128Q;*rrf-3 *animals are sensitive to RNAi

**Figure 1:**
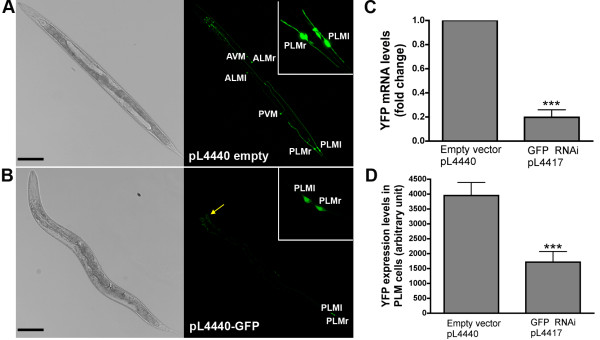
**Reduction of YFP expression in 128Q;*rff-3 *nematodes treated with RNAi against GFP**. Following incubation of ID447 L1 larvae with bacteria expressing either empty vector (pL4440) or RNAi against GFP (pL4417) until F1 young adults are produced, YFP levels in PLM touch receptor cells were measured by image analysis, and YFP mRNA levels were measured by qRT-PCR from whole animals. (**A**) Representative image showing YFP expression (pseudocolored in green) in animals treated with empty vector. The name of the *mec-7p *target cells is indicated. The insert shows a closer view of the left (PLMl) and right (PLMr) PLM cell. (**B**) Representative image showing reduced YFP expression in animals treated with RNAi against GFP. The yellow arrow indicates natural auto-fluorescence of the gut granules. This fluorescence is also present in the control animals and works as an internal control of light intensity, showing that pictures were collected in the confocal microscope under the same conditions. The insert shows a closer view of PLM cell left (PLMl) and right (PLMr). (**C**) YFP mRNA levels are reduced by treatment with pL4417. Data are means ± SE (n = 5). ***P < 0.001 compared to pL4440. (**D**) YFP expression levels in PLM cells are reduced by treatment with pL4417. Data are means ± SEM (n = 3) as compiled from greater than 70 animals/experiment. ***P < 0.001 compared to pL4440. Scale bar is 100 μm in all panels.

To construct polyQ strains with enhanced RNAi sensitivity in neurons, we crossed the enhanced RNAi strain YY13 which carries the rrf-3(mg373)II mutation [19] with integrated strains that co-express N-terminal (amino acids 1-57) htt under the control of *mec3-p *and YFP under the control of *mec7-p *[14]. This resulted in strain ID448 (19Q;*rrf-3*) and strain ID447(128Q;*rrf-3*). The efficiency of gene inactivation in touch receptor neurons was tested by incubating strain ID447 L1 larvae in 96-well plates with bacteria expressing either RNAi against GFP (plasmid L4417) or empty vector (plasmid L4440) until F1 young adults are produced. Treatment with RNAi against GFP induced an overall reduction of YFP expression levels in *mec7-p *target cells as illustrated in Figure 1A/B. The level of YFP mRNA expression was strongly reduced (about 80%) by RNAi against GFP compared to empty vector (Figure 1C). Additionally, the level of YFP expression in the mechanosensory neurons that control the response to touch at the tail (PLM neurons) was reduced by RNAi against GFP compared to empty vector (Figure 1D). Collectively, these results indicated that the touch receptor neurons of F1 young adults are sensitive to RNAi. Consistent with previous reports [37], no or little reduction of YFP levels was detected in F0 young adults (data not shown).

### A large-scale functional RNAi screen identifies 662 genes that modulate neuron dysfunction in expanded-polyglutamine nematodes

**Figure 2:**
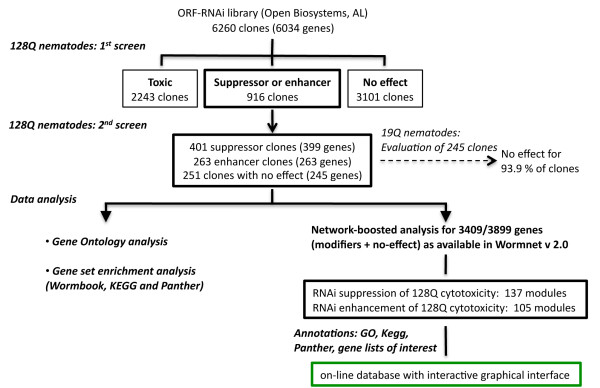
**Overview of the screening and data analysis strategy**.

Using 128Q nematodes in the *rrf-3 *sensitized background, we then conducted a primary RNAi screen for the modulation of neuronal dysfunction. This screen was based on the manual quantification of response to light touch at the tail of F1 young adult animals. We screened 6260 RNAi clones equaling 6034 genes in the *C. elegans *genome (Figure 2). To decrease the risk for false positive, we applied a stringent threshold where the modification of touch response was considered significant if the score for touch response was outside the mean baseline ± SD*2.6 (Additional file 1: Figure S1). This screen identified 2243 RNAi clones (2211 genes) that caused toxicity phenotypes such as lethality, larval arrest, egg-laying defect, developmental delay and other abnormalities such as morphological abnormalities, 3101 RNAi clones (3054 genes) that showed no effect and 916 RNAi clones (769 genes) that modulated neuronal dysfunction (Figure 2, Additional file 2: Table S1; Additional file 3: Table S2). The latter class of RNAi clones was subjected to a second screen in 128Q nematodes, which led to the identification of 664 RNAi clones confirmed to modulate neuronal dysfunction. These clones corresponded to 662 genes, consisting of 399 genes that reduced neuronal dysfunction when knocked-down by RNAi and 263 genes that enhanced neuronal dysfunction when knocked-down by RNAi (Additional file 4: Table S3). As previously reported, 128Q nematodes show at least 80% decreased touch response at the tail with a strong level of axonal dystrophy, whereas 19Q nematodes show a 45-50% reduction of touch response at the tail without significant axonal dystrophy [14]. Since our screen was based on the manual quantification of touch response in 70-90 animals/RNAi clone, which is considerably more labour-intensive and time-consuming compared to faster RNAi screens such as those looking for modifiers of mutant htt aggregation [38,39], we evaluated whether the modifiers of 128Q-neuron dysfunction might also modify neuronal dysfunction in 19Q;*rrf-3 *nematodes by testing a subset of the 662 genes (245 genes choosen at random). Only 15/245 genes (6.1%) were found to modulate neuronal dysfunction in 19Q;*rrf-3 *nematodes when knocked-down by RNAi (Additional file 5: Table S4), suggesting that most of the 662 genes emphasized by screens in 128Q;*rrf-3 *nematodes specifically modify 128Q cytotoxicity. Genome-wide RNAi screens previously aimed at identifying modifiers of polyQ aggregation in *C. elegans *[40,41], Consistent with the notion that aggregation and cytotoxicity can be genetically uncoupled [41], the overlap with our RNAi screen was small with 12 modifiers of 128Q-neuron dysfunction previously found to enhance the aggregation of 35Q-peptides when knocked-down by RNAi [40] and three other modifiers of 128Q-neuron dysfunction previously found to suppress the aggregation of 35Q-peptides when knocked-down by RNAi [41] (Additional file 6: Table S5).

### In-depth data analysis emphasizes modifiers of 128Q-neuron dysfunction in pathways associated with metabolism, development, survival and neurodegenerative disease

**Figure 3:**
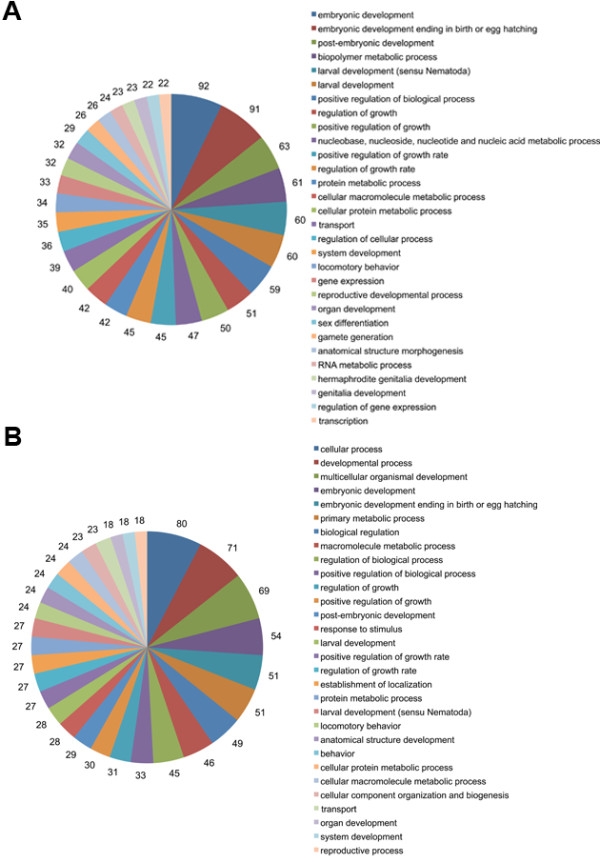
**Gene Ontology classification of genes that modulate neuronal dysfunction induced by expanded polyQ expression**. Genes were classified based on their functional annotations in the GO categories 'Biological Process', 'Molecular Function' and 'Cellular component'. Pie-charts for the most frequent 'Biological process' categories (corresponding to at least 18 genes) are shown for gene inactivations that reduced 128Q-neuron dysfunction (**A**) and those that aggravated neuronal dysfunction (**B**). The number of genes is indicated. See the indicated supplementary tables (Supplementary Tables 4-5) for the complete lists of genes in each GO category.

The 662 genes associated with the modification of 128Q-neuron dysfunction appeared to encompass a variety of biological processes (cell death, protein folding, intracellular transport, metabolic processes, response to stress, stress-activated pathways) that may have a role in neurodegenerative disease pathogenesis as suggested by their functional classification using GO annotations (Figure 3, Additional file 7: Tables S6; Additional file 8: Table S7). To further assess the biological content corresponding to modifiers of 128Q-neuron dysfunction, we performed two types of analysis. First, we used gene lists describing major signaling pathways (see Materials and Methods) to perform gene set enrichment analyses. This analysis was performed for genes that either aggravate (herein referred to as 'protection-associated' or PA genes) or suppress (herein referred to as 'pathogenic process-associated' or PPA genes) 128Q-neuron dysfunction when knocked-down by RNAi. Pathway analysis for PA genes identified eight metabolic pathways such as for example those involved in glycolysis and amino acid metabolism (Table 1). Consistent with the notion that mutant htt expression may dysregulate a large number of genes and alter a variety of biological processes [3,10], pathway analysis for PPA genes identified three main pathway categories, including pathways primarily involved in metabolism (glycolysis, amino acid metabolism), cytokine signaling/immune system (interferon-gamma signaling, interleukin signaling, Toll receptor signaling) and cellular differentiation and homeostasis (IGF pathway, angiogenesis, integrin signaling, p53 pathway by glucose deprivation, FGF signaling, EGF signaling, Toll receptor signaling) (Table 2). Interestingly, the latter category included the Alzheimer's disease (AD)-amyloid secretase pathway, pathways previously associated to neuroprotection in HD such as IGF signaling [14] and FGF signaling [42], pathways that could play a role in neurodegenerative disease pathogenesis such as EGF signaling [43], and pathways having a dual role in neurodegeneration and neuroprotection such as Toll receptor signaling [44]. These observations illustrated the revelance of our RNAi dataset to neurodegeneration. Given that PPA genes might constitute preferred targets for therapeutic intervention, these results suggested that several of the targets for early-stage neuroprotection in HD may lie in the pathways that are important to neuronal cell differentiation and survival.

**Table 1:** Pathway analysis for 263 genes that enhanced 128Q-neuron dysfunction when knocked-down by RNAi

Pathway	P value	Gene
Ascorbate and aldarate metabolism	0.00000	B0218.8/clec-52, C01G6.1/aqp-2, C08B11.8, C12C8.2, C13B9.1, C36E8.3/pxd-1, C50D2.8, C52B9.1/cka-2, C52E4.1/cpr-1, C53B7.3, C54C8.9/nlp-39, D1025.4/nspc-20, D1086.3, F08B1.1/vhp-1, F29G6.2, F38E11.1/hsp-12.3, F45C12.7/btb-6, F46H5.3, F49E12.1, F54B11.3/unc-84, F54F7.3, F56D6.2/clec-67, K07E3.3/dao-3, K08D8.3, K09C4.5, M02F4.7/clec-265, M03F4.7/calu-1, T07H3.3/math-38, T16H12.1, T23B3.2, T23H2.2/snt-4, W08D2.4/fat-3, Y38H6C.3/dct-14, ZC410.5, ZC416.6, ZK6.11
Starch and sucrose metabolism	0.00119	F57B10.7/tre-1, R05F9.6, T22F3.3, Y87G2A.8/gpi-1
Tyrosine metabolism	0.00535	B0334.11/ooc-3, F46C3.1/pek-1, M110.5/dab-1
Amino sugar and nucleotide sugar metabolism	0.01885	F22B3.4, R05F9.6, Y87G2A.8/gpi-1
Glycolysis	0.03209	T03F1.3/pgk-1, Y87G2A.8/gpi-1
Glycolysis/Gluconeogenesis	0.03332	R05F9.6, T03F1.3/pgk-1, Y87G2A.8/gpi-1
Fatty acid biosynthesis	0.03415	W09B6.1/pod-2
Pentose phosphate pathway	0.04375	R05F9.6, Y87G2A.8/gpi-1

Pathway analysis was based on hypergeomeric tests using pathways from Wormbook, KEGG and Panther. *P *< 0.05 was considered significant and results are shown by increasing *P *values.

**Table 2:** Pathway analysis for 399 genes that reduced 128Q-neuron dysfunction when knocked-down by RNAi

Pathway	P value	Gene
Ascorbate and aldarate metabolism	0.00000	B0281.5, B0286.3, B0478.1/jnk-1, C06B3.4/stdh-1, C08E3.4/fbxa-161, C08F8.5/fbxb-9, C09G1.1/pqn-11, C10C5.4, C10C5.5, C10G11.5/pnk-1, C17G10.5/lys-8, C17H12.8, C39D10.7, C45B11.3/dhs-18, C46E10.7/srh-99, C54D10.3, D1054.10, D2045.6/cul-1, F07F6.1, F07F6.5/dct-5, F09B12.3, F17E5.1/lin-2, F28D1.5/thn-2, F29G9.1, F31C3.6, F36F2.1, F38E11.2/hsp-12.6, F38H4.8/ech-2, F41B5.4/cyp-33C3, F42G10.1, F43G9.5, F44D12.8, F47C10.2/btb-21, F52H3.5, F53C3.5, F56D5.5, F56G4.3/pes-2.2, F57C9.4, F57H12.7/mec-17, H04M03.1, H10D18.2/scl-12, K02B12.2, K02E11.5, K10B3.8/gpd-2, M01E11.2, M02D8.4, R03E9.1/mdl-1, R05F9.10/sgt-1, R12B2.5/mdt-15, R12H7.2/asp-4, T03F6.1/qdpr-1, T17H7.1, T20B5.3/oga-1, T21C9.8/ttr-23, T22G5.2/lbp-7, T24B8.5, W02D3.1, W03D2.6, W05H9.1, Y106G6H.7/sec-8, Y106G6H.9, Y38H6C.1/dct-16, Y43C5B.2, Y47D3B.7/sbp-1, ZK1127.10, ZK1290.6/rnh-1.1, ZK384.2/scl-20, ZK520.4/cul-2, ZK550.6, ZK637.8/unc-32, ZK829.6/tgt-1
Sulfate assimilation	0.00001	B0218.3/pmk-1, B0412.2/daf-7, B0478.1/jnk-1, C47G2.2/unc-130, C53D6.2/unc-129, F38A6.1/pha-4, F43C1.2/mpk-1, T23H2.5/rab-10, Y11D7A.4/rab-28
Pyrimidine metabolism	0.00113	B0304.1/hlh-1, B0478.1/jnk-1, B0547.1/csn-5, C04E7.2/sor-3, C07B5.5/nuc-1, C25D7.3/sdc-3, C34E10.7/cnd-1, C37A2.4/cye-1, C47G2.2/unc-130, F14F3.1/vab-3, F38A6.1/pha-4, F56A8.7/unc-64, K01G5.2/hpl-2, Y47D3A.6/tra-1, ZK520.4/cul-2
Pyridoxal phosphate salvage pathway	0.00176	B0218.3/pmk-1, B0478.1/jnk-1, F43C1.2/mpk-1, Y18D10A.5/gsk-3, Y38F1A.10/max-2
Oxytocin receptor mediated signaling	0.00236	B0478.1/jnk-1, B0547.1/csn-5, C44H4.6, F43C1.2/mpk-1, Y11D7A.4/rab-28, Y18D10A.5/gsk-3
Interferon-gamma signaling	0.00251	B0218.3/pmk-1, B0478.1/jnk-1, F43C1.2/mpk-1
Glycosphingolipid biosynthesis - ganglio series	0.00297	T14F9.3/hex-1, T26C5.3
Cyanoamino acid metabolism	0.00340	B0035.8/his-48, B0304.1/hlh-1, B0478.1/jnk-1, B0547.1/csn-5, C02B8.4/hlh-8, C04E7.2/sor-3, C07B5.5/nuc-1, C09G9.7, C18A3.1, C25A1.11/aha-1, C25D7.3/sdc-3, C29F9.5, C34E10.7/cnd-1, C37A2.4/cye-1, C47G2.2/unc-130, F11A10.1/lex-1, F14F3.1/vab-3, F22D3.1/ceh-38, F22F1.1/hil-3, F38A6.1/pha-4, F45F2.4/his-7, F54C8.2/cpar-1, F56A8.7/unc-64, K01G5.2/hpl-2, K02B9.4/elt-3, R03E9.1/mdl-1, R05F9.10/sgt-1, R07B1.1/vab-15, R13H8.1/daf-16, T05A6.1/cki-1, T19B10.11/mxl-1, T27F2.1/skp-1, W06E11.1, Y39B6A.2/pph-5, Y47D3A.6/tra-1, Y47D3B.7/sbp-1, Y49E10.1/rpt-6, Y57E12AL.5/mdt-6, ZC204.2, ZK131.7/his-13, ZK520.4/cul-2, ZK652.5/ceh-23
Taurine/hypotaurine metabolism	0.00348	F56A8.7/unc-64, K02D10.5, T10H9.3, Y22F5A.3/ric-4
Glycolysis/Gluconeogenesis	0.00365	AC3.7/ugt-1, C05C8.3/fkb-3, C06B3.4/stdh-1, C07B5.5/nuc-1, F11A5.12/stdh-2, F38E11.2/hsp-12.6, K10B3.8/gpd-2, R03E9.1/mdl-1, R12B2.5/mdt-15, R13H8.1/daf-16, T22G5.2/lbp-7, T28B8.2/ins-18
Selenocompound metabolism	0.00365	AC3.7/ugt-1, C05C8.3/fkb-3, C06B3.4/stdh-1, C07B5.5/nuc-1, F11A5.12/stdh-2, F38E11.2/hsp-12.6, K10B3.8/gpd-2, R03E9.1/mdl-1, R12B2.5/mdt-15, R13H8.1/daf-16, T22G5.2/lbp-7, T28B8.2/ins-18
Insulin_IGF pathway-protein kinase B signaling cascade	0.00404	C44H4.6, C47G2.2/unc-130, F38A6.1/pha-4, Y18D10A.5/gsk-3
Salvage pyrimidine ribonucleotides	0.00417	F42G9.7/snt-2, K02D10.5, Y22F5A.3/ric-4
Tetrahydrofolate biosynthesis	0.00417	B0218.3/pmk-1, B0478.1/jnk-1, F43C1.2/mpk-1
Apoptosis signaling	0.00503	B0218.3/pmk-1, B0478.1/jnk-1, F43C1.2/mpk-1, Y18D10A.5/gsk-3, Y38F1A.10/max-2
Glycosaminoglycan degradation	0.00520	K09E4.4, T14F9.3/hex-1
Angiogenesis	0.00520	C06B3.4/stdh-1, F11A5.12/stdh-2, K04A8.5
mRNA splicing	0.00534	F56A8.7/unc-64, K02D10.5, Y22F5A.3/ric-4, Y48B6A.8/ace-3
Muscarinic acetylcholine receptor 1 and 3 signaling	0.00608	F56A8.7/unc-64, K02D10.5, Y22F5A.3/ric-4, Y48B6A.8/ace-3
Alanine biosynthesis	0.00769	K02D10.5, K08F8.4/pah-1, Y22F5A.3/ric-4
Butanoate metabolism	0.00777	C06B3.4/stdh-1, F11A5.12/stdh-2, K03A1.5/sur-5, T02G5.7
5-Hydroxytryptamine biosynthesis	0.00798	K02D10.5, Y22F5A.3/ric-4
Interleukin signaling	0.00974	C47G2.2/unc-130, F38A6.1/pha-4, F43C1.2/mpk-1, Y18D10A.5/gsk-3
Amino sugar and nucleotide sugar metabolism	0.01084	C01F1.3, C53B4.7/bre-1, K08E3.5, T14F9.3/hex-1
Metabotropic glutamate receptor group II pathway	0.01254	F56A8.7/unc-64, K02D10.5, Y22F5A.3/ric-4
Linoleic acid metabolism	0.01511	C06B3.4/stdh-1, F11A5.12/stdh-2
Beta3 adrenergic receptor signaling	0.01511	K02D10.5, Y22F5A.3/ric-4
Integrin signaling	0.01742	B0218.3/pmk-1, B0478.1/jnk-1, C27B7.8/rap-1, C36B1.1/cle-1, F43C1.2/mpk-1
p53 pathway by glucose deprivation	0.01908	C47G2.2/unc-130, F38A6.1/pha-4, F48E8.1/lon-1, Y18D10A.5/gsk-3
5-Hydroxytryptamine degredation	0.01939	K02D10.5, Y22F5A.3/ric-4
Ornithine degradation	0.02121	B0218.3/pmk-1, B0478.1/jnk-1, T19B10.11/mxl-1
Cortocotropin releasing factor receptor signaling	0.02412	K02D10.5, Y22F5A.3/ric-4
FGF signaling	0.02627	B0218.3/pmk-1, B0478.1/jnk-1, F26E4.1/sur-6, F43C1.2/mpk-1
N-acetylglucosamine metabolism	0.02712	F56A8.7/unc-64, K02D10.5, T02C12.1/hum-5, Y22F5A.3/ric-4, Y48B6A.8/ace-3
5HT3 type receptor mediated signaling	0.02928	K02D10.5, Y22F5A.3/ric-4
B cell activation	0.02939	B0218.3/pmk-1, B0478.1/jnk-1, F43C1.2/mpk-1
Fructose and mannose metabolism	0.03565	C06B3.4/stdh-1, C53B4.7/bre-1, F11A5.12/stdh-2
Pantothenate and CoA biosynthesis	0.04074	F25H9.6, T04G9.4
Alzheimer disease-amyloid secretase pathway	0.04074	K02D10.5, Y22F5A.3/ric-4
Beta1 adrenergic receptor signaling	0.04074	K02D10.5, Y22F5A.3/ric-4
Beta2 adrenergic receptor signaling	0.04074	K02D10.5, Y22F5A.3/ric-4
EGF receptor signaling	0.04229	B0218.3/pmk-1, B0478.1/jnk-1, C27B7.8/rap-1, F43C1.2/mpk-1
Toll receptor signaling	0.04494	C08B6.9/aos-1, F11A10.1/lex-1, F40G9.3/ubc-20, Y49E10.1/rpt-6
Alanine, aspartate and glutamate metabolism	0.04494	C04E7.2/sor-3, C34E10.7/cnd-1, C47G2.2/unc-130, F57H12.7/mec-17
Serine glycine biosynthesis	0.04620	B0478.1/jnk-1, F43C1.2/mpk-1, Y38F1A.10/max-2
Lysine biosynthesis	0.04869	C25A11.4/ajm-1, C36B1.1/cle-1, F17E5.1/lin-2, F22B5.1/evl-20, F43C9.4/mig-13, F56A8.7/unc-64, Y22F5A.3/ric-4

Pathway analysis was based on hypergeomeric tests using pathways from Wormbook, KEGG and Panther. *P *< 0.05 was considered significant and results are shown by increasing *P *values.

In contrast to metabolic pathways which may show a rather simple structure, pathways that respond to external stimuli to regulate gene expression may be highly inter-connected, and they may have highly-branched structures and several biological outcomes. Additionally, RNAi-based screens may generate noise due to off-target effects and variations in the degree of knockdown of target genes. Therefore, to map RNAi activity to functional clusters and to identify a more reliable set of genes from the RNAi hits, we used a network-based approach. To this end, we used a Fourier-like analysis [32] based on the reference network Wormnet [26]. To enhance the discriminative power and reliability of this analysis, we developed and applied an optimized version of the Fourier-like analysis (see Materials and Methods). We analyzed the gene set for which a score for activity (S score) was available (genes that either modulate 128Q-neuron dysfunction or produce no effect when knocked-down by RNAi). The modules resulting from this analysis were annotated by using Gene Ontology, KEGG and Panther pathways and gene lists of interest (conserved genes, druggable genes, htt partners, genes involved in autophagy, mitochondrial function and synaptic activity). These analyses identified a more reliable set of 286 modifier genes as distributed in 137 modules for suppression of 128Q-neuron dysfunction by RNAi and 105 modules for aggravation of 128Q-neuron dysfunction by RNAi (Additional file 9: Table S8; Additional file 10: Table S9). Modules of variable sizes were obtained (2-50 genes) for either suppression or aggravation of 128Q-neuron dysfunction. Of note, small modules are equally significant compared to large modules as the size of the resulting modules was always driven by the stable and best balance between gene connectivity and gene activity during the spectral decomposition process (see Materials and Methods). The modules and their content can be visualized using a freely-accessible database (see http://www.broca.inserm.fr/EHDN2/RNAiscreen) that contains a query page and interactive graphical interface [36]. Our network-based approach confirmed the trends observed in gene set enrichment analysis, namely the detection of pathways involved in metabolism, immune response and development. Additionally, it provided insights into the possible mechanisms of how RNAi hits potentially interact to modify 128Q-neuron dysfunction. We notably detected modules seeded by RNAi hits that were annotated for 'Huntington's disease', 'Parkinson's disease' and 'panthotenate kinase activity', the latter previously associated with neurodegeneration [45]. More largely, we detected modules annotated for biological processes established or strongly suspected (see [3] for review of HD mechanisms) to have a role in neurodegenerative disease pathogenesis such as 'oxidative phosphorylation', 'cholesterol biosynthesis' and 'cell cycle'. Interestingly, several modules were annotated for cell differentiation and survival pathways such as 'Wnt signaling', a pathway suspected to have a role in HD pathogenesis [46], 'apoptosis signaling' and 'integrin signaling', suggesting that such pathways might be targeted for therapeutic intervention in HD. Annotations for metabolic pathways such as 'sucrose metabolism', 'pentose phosphate pathway' and 'cholesterol biosynthesis' were also detected, consistent with the notion that metabolic pathways may be targeted for therapeutic intervention in HD [3,47]. Additionally, several modules were annotated for genes of interest in HD such as druggable genes (as notably observed in the modules for suppression of 128Q-neuron dysfunction by RNAi; see Additional file 9: Table S8), htt partners, and genes involved in autophagy, mitochondrial function and synaptic activity, which further illustrated the relevance of our dataset to neuroprotective target discovery in HD.

### Modifiers of 128Q-neuron dysfunction in *C. elegans *are dysregulated in the striatum of either CHL2 knock-in mice or R6/2 transgenic mice

**Figure 4:**
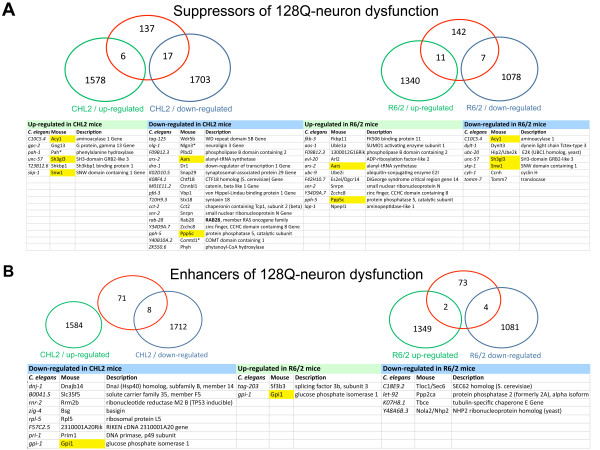
**Overlap between 239 conserved modifiers of 128Q-neuron dysfunction in *C-elegans *and genes dysregulated in the striatum of either CHL2 knock-in mice or R6/2 transgenic mice**. (**A**) Overlap between dysregulated genes in the mouse striatum and suppressors of 128Q-neuron dysfunction that have mouse orthologs. Numbers of genes are indicated. The lists of genes at the intersection of the nematode and mouse datasets are indicated below the graphs. (**B**) Overlap between dysregulated genes in the mouse striatum and enhancers of 128Q-neuron dysfunction that have mouse orthologs. The numbers of genes is indicated. The lists of genes at the intersection of the nematode and mouse datasets are indicated below the graphs. Some genes are oppositely dysregulated in the CHL2 mice compared to R6/2 mice (yellow tags). In all panels, red circles indicate *C. elegans *genes that modify 128Q-neuron toxicity. *Druggable genes.

To further assess the relevance of our RNAi data to candidate target profiling and discovery in HD, we next compared our dataset to striatal gene expression data collected from mouse models of HD. Given that 128Q nematodes recapitulate progressive neuron dysfunction, which may occur in HD before cell loss, we compared our RNAi data to striatal gene expression data collected from the CHL2(Q150/Q150) knock-in mice, a model that may recapitulate progressive neuronal injury similar to that found early in the course of HD [48]. To reach a more specific level of comparison, we also compared our data to striatal gene expression data collected from the R6/2 transgenic mice, a model that exhibits rapid phenotypic changes as induced by a short N-terminal fragment of mutant htt [10]. Among the 662 modifiers of 128Q-neuron dysfunction in *C. elegans*, 239 genes were conserved in the mouse. Forty-nine genes were found at the intersection of the *C. elegans *RNAi data and gene dysregulation data in HD mice (Figure 4). Six of these 49 genes including Acy1, Sh3gl3, Snw1, Aars, Ppp5 and Gpi1 were oppositely dysregulated in the CHL2 mice compared to R6/2 mice (Figure 4), illustrating the differences that may exist between these two models at the molecular level. In contrast, genes that are involved in a variety of biological processes and that modulate 128Q-neuron dysfunction when knocked-down by RNAi were dysregulated in either the CHL2 or R6/2 mice (Figure 4), illustrating how data comparison using substractive analysis may be useful for target prioritisation. Notably, 12 genes that suppress 128Q-neuron dysfunction when knocked-down by RNAi in *C. elegans *were observed to be up-regulated in either the CHL2 or R6/2 mice, suggesting that inhibiting these genes might elicit neuroprotection in HD. One of them, the phenylalanine-4-hydroxylase Pah, appeared to be particularly interesting since it is a druggable gene that acts upstream of tyrosine-dopamine biosynthesis and since increased dopamine levels were previously associated with early HD phenotypes such as hyperkinesia [49].

## Discussion

The aim of our study was to collect information on the genes and pathways that may act as modifiers of the early stage of mutant htt toxicity in HD such as neuron dysfunction. To this end, we used an *in vivo *model to screen for gene inactivations that modulate the neuronal dysfunction induced by expanded-polyQ expression in *C. elegans*, and we used network-boosted analysis to enhance the identification of candidate pathways that may be further evaluated in mammalian systems. We identified 662 hits from the evaluation of 6034 genes. Compared to RNAi screens based on biochemical processes such as protein aggregation [38-40], the relatively high number of hits in our study is likely to reflect the multigenic nature of behavioral phenotypes such as response to touch rather than changes in polyQ-expanded htt expression levels. Previously-identified modulators of neuron dysfunction in expanded-polyQ nematodes do not change transgene expression levels [14,18], further suggesting most RNAi effects are not due to changes in htt expression levels.

In contrast to 128Q-neuron dysfunction, neuronal dysfunction produced by normal polyQ expression in our trangenic nematodes is not directly relevant to HD pathogenesis. Microarray analysis of gene expression in touch receptor cells isolated by fluorescence-activated cell sorting indeed indicated that only 18 out of 2000 genes dysregulated by 128Q expression are also dysregulated by 19Q expression (submitted elsewhere), and several of the expanded-polyQ cytotoxicity that we previously identified showed no effect on normal polyQ cytotoxicity [14,18]. Additionally, we observed that greater than 90% of the modifiers of 128Q-neuron dysfunction showed no effect in 19Q nematodes using a subset of 243 genes identified by RNAi screening in 128Q nematodes. Collectively, these observations suggest that the mechanisms of neuron dysfunction in 19Q nematodes [14] are different from those underlying the strong level of neuron dysfunction in 128Q nematodes. While specific modifiers of expanded-polyQ cytotoxicity might constitute preferred HD targets, one cannot exclude the possibility that non-specific modifiers of neuron dysfunction in polyQ nematodes may belong to the core machinery for neuron differentiation and survival, thus being of potential interest, at least from a screening perspective. This was for example illustrated by two N-acetylgalactosaminyltransferases (*gly-9 *and *gly-3*), a family of enzymes involved in neuron differentiation and survival [50].

A major feature of network-boosted analysis is to reduce data complexicity by highlighting individual pathways and possibly inter-connected pathways that are embedded into modules seeded with gene activity. In this respect, the network-boosted data analysis of our RNAi dataset was more instructive compared to the sole use of GO annotations or gene set enrichment analysis. While a role for a variety of pathways was suggested by gene set enrichment analysis, network-boosted analysis indeed emphasized a role for these pathways in a more comprehensive fashion, providing new insights of how the genes associated to these pathways may interact within modules. Furthermore, network-boosted analysis identified pathways not detected by gene set enrichment analysis. As indicated by the pathways embedded in the modules resulting from network-boosted analysis (see the on-line database at http://www.broca.inserm.fr/EHDN2/RNAiscreen), a role for neurodegenerative disease pathways and neurodevelopmental/cell survival pathways in the modulation of expanded-polyQ cytotoxicity was emphasized, which may provide guidance to the selection of candidate targets for neuroprotection in HD. The annotation of the modules resulting from network-boosted analysis identified clusters enriched in genes of interest such as druggable genes, htt partners and genes involved in autophagy, mitochondrial function and synaptic activity (all gene classes which can be queried using the on-line database), providing additional guidance to neuroprotective target discovery in HD. Here, it is important to note that the hypothesis underlying network-based approaches is that highly-interconnected genes might show similar biological activities. In this respect, the network-boosted analysis of our data is predictive of the genes that might decrease or increase 128Q-neuron dysfunction when knocked-down by RNAi, even though they showed no effect or borderline effects in the RNAi screen. While this may increase the number of candidate targets to be considered for neuroprotective intervention, it decreases the risk for noise associated with RNAi screens and favors the inclusion of potentially-interesting information for multi-model target discovery.

Multi-model target discovery is strongly expected to enhance the drug discovery process for neuroprotection in HD. A standard method for the validation of modifiers of neuronal dysfunction as instructed by simple models of HD pathogenesis relies on target validation in the mouse. Because it is not yet feasible to test in the mouse all genes supported as modifiers of neuronal decline in simple models, the outcome of our functional screen and other screens will be analyzed using data integration procedures in which the selection of the best targets for validation will rely on target expression levels and activity in multiple models of HD. One of the objectives of data integration is to assess the relevance of simple-model data to mouse models, and more largely to human neurodegenerative disease. In this respect, it is notable that several modifiers of 128Q-neuron dysfunction found in our screen were previously associated with the pathogenesis of neurodegenerative diseases such as HD or AD, suggesting that our *C. elegans *dataset is relevant to HD pathogenesis in higher order models of the disease. This is for example the case for four genes retained by network-boosted analysis including (*i*) *dnj-1/*DNAJ, a gene that encodes a DNAJ chaperone [51], aggravates neuron dysfunction when knocked-down by RNAi and is down-regulated in the CHL2 mice, (*ii*) *sel-8*, a *C. elegans *gene that encodes a nuclear protein required for *lin-12*/NOTCH signaling, which may have neuron survival activities [52], and that aggravates 128Q-neuron dysfunction when knocked-down by RNAi, (*iii*) *jnk-1*/JNK, a gene that promote HD pathology in a rat model of the disease [53] and suppresses 128Q-neuron dysfunction when knocked-down by RNAi, and (*iv*) *pnk-1*/PanK4, a gene that encodes a panthotenate kinase and suppresses 128Q-neuron dysfunction when knocked-down by RNAi, which is consistent with the pathological role of panthotenate kinases in neurodegeneration [45]. Additionally, the comparison of our data to gene dysregulation in the striatum of CHL2 knock-in mice *versus *that of R6/2 transgenic mice further indicated that our dataset contains information relevant to HD target selection as illustrated by the proposed inhibition of phenylalanine-4-hydroxylase as a candidate strategy to reduce early HD phenotypes that may be associated to a transient excess of dopamine [49].

## Conclusions

In conclusion, our results provide new insights into the genes and pathways that might regulate the dysfunction and death of neurons expressing mutant polyQs, emphasizing a role for specific metabolic, developmental and pro-survival pathways. Several models of HD are being used to screen for modifiers of mutant polyQ toxicity and for genes dysregulated by mutant htt. In this context, the use of cross-species data integration for the analysis of our RNAi dataset and other datasets is anticipated to boost the prediction and prioritisation of neuroprotective targets in HD, and this will be investigated in additional studies.

## Competing interests

The authors declare that they have no competing interests.

## Authors' contributions

FXL, LM and FP designed and performed data analysis and developed database tools. CB contributed to database developments. JPV contributed essential tools for data analysis. RVM, JAP and CT designed and performed *C. elegans *experiments. CN conceived and supervised experiments. FXL and CN wrote the manuscript. All authors read and approved the final manuscript.

## Supplementary Material

Additional file 1 **Figure S1**. Distribution of S scores for the primary screen in 128Q;*rrf-3 *nematodes. The graph shows the S scores for 4017 RNAi clones that did not elicit lethality or developmental abnormalities. The S score was calculated as [(Percent response - mean baseline)/mean baseline]. The maximally-achievable score is 4.55 (100% response to touch) and the smallest score is -1 (complete loss of touch response). The blue line indicate the mean baseline (S = 0). Dooted blue lines show the mean baseline ± SD*2.6. Clones with S scores falling outside the mean baseline ± SD*2.6 interval were retained for the secondary screen. Click here for file

Additional file 2 **Table S1**. List of the 2211 genes that caused lethality and developmental abnormalities when knocked-down by RNAi. Click here for file

Additional file 3 **Table S2**. List of the 3823 genes that either showed no effect or modified 128Q-neuron dysfunction when knocked-down by RNAi in the primary screen. Click here for file

Additional file 4 **Table S3**. List of the 662 genes that modified 128Q-neuron dysfunction when knocked-down by RNAi in the secondary screen. Click here for file

Additional file 5 **Table S4**. List of the 15 genes that modified 19Q-neuron dysfunction when knocked-down by RNAi. Click here for file

Additional file 6 **Table S5**. List of the 15 genes that modified 128Q-neuron dysfunction in the secondary screen and were previously reported to modify polyQ aggregation when knocked-down by RNAi. Click here for file

Additional file 7 **Table S6**. Gene Ontology classification of genes that suppressed 128Q-neuron dysfunction when knocked-down by RNAi. Click here for file

Additional file 8 **Table S7**. Gene Ontology classification of genes that aggravated 128Q-neuron dysfunction when knocked-down by RNAi. Click here for file

Additional file 9 **Table S8**. Modules (n = 137) generated by network-boosted analysis for suppression of 128Q-neuron dysfunction by RNAi. Click here for file

Additional file 10 **Table S9**. Modules (n = 105) generated by network-boosted analysis for aggravation of 128Q-neuron dysfunction by RNAi. Click here for file
