# Supplementary figures and images for: Large-scale functional RNAi screen in C. elegans identifies genes that regulate the dysfunction of mutant polyglutamine neurons
Source: BMC Genomics. 2012 Mar 13;13:91. doi: 10.1186/1471-2164-13-91 (PMC3331833; doi:10.1186/1471-2164-13-91)

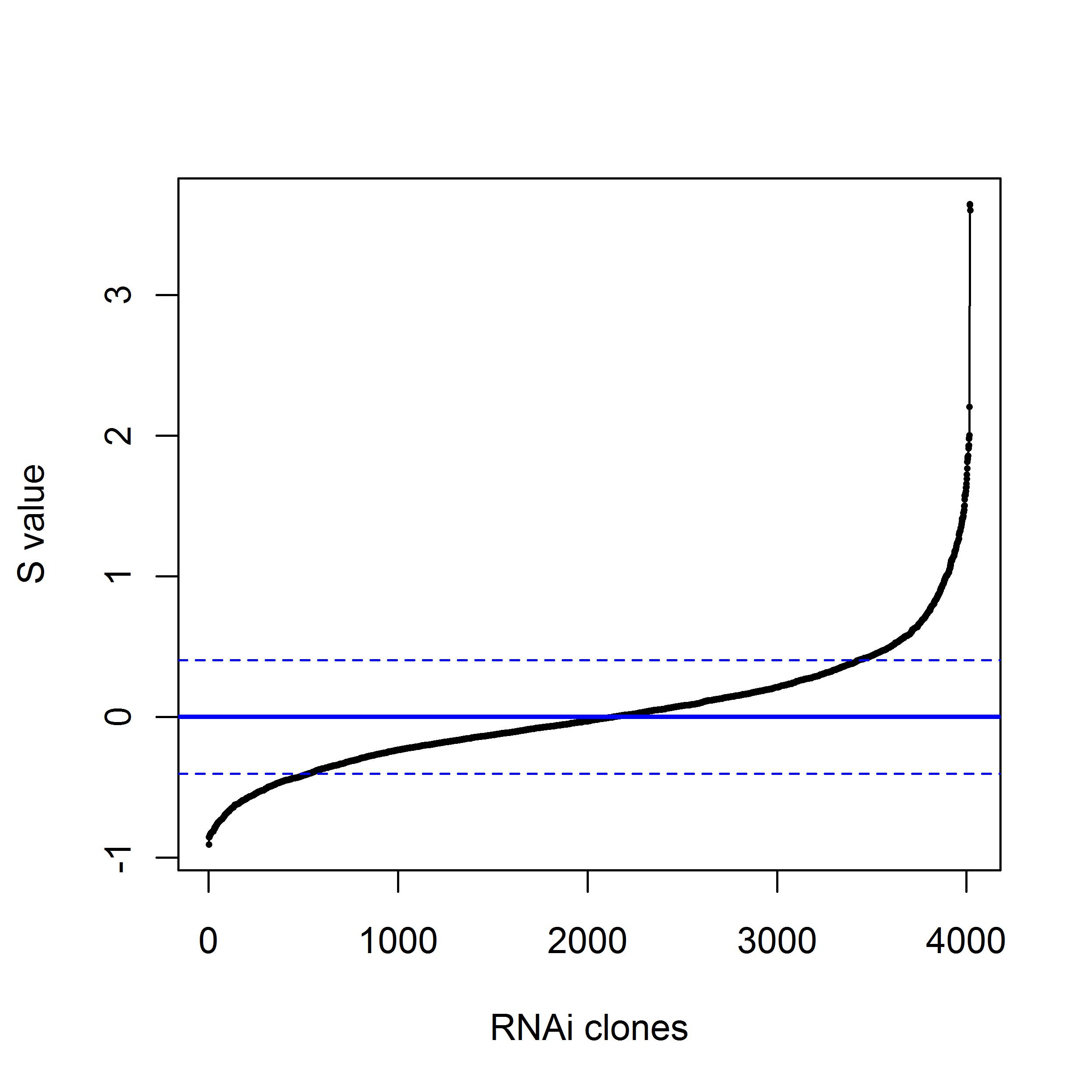

Supplement: Additional file 1 — Figure S1. Distribution of S scores for the primary screen in 128Q;rrf-3 nematodes. The graph shows the S scores for 4017 RNAi clones that did not elicit lethality or developmental abnormalities. The S score was calculated as [(Percent response - mean baseline)/mean baseline]. The maximally-achievable score is 4.55 (100% response to touch) and the smallest score is -1 (complete loss of touch response). The blue line indicate the mean baseline (S = 0). Dooted blue lines show the mean baseline ± SD*2.6. Clones with S scores falling outside the mean baseline ± SD*2.6 interval were retained for the secondary screen. [file 1471-2164-13-91-S1.JPEG]
