# Supplementary material for: Large-scale functional RNAi screen in C. elegans identifies genes that regulate the dysfunction of mutant polyglutamine neurons
Source: BMC Genomics. 2012 Mar 13;13:91. doi: 10.1186/1471-2164-13-91 (PMC3331833; doi:10.1186/1471-2164-13-91)
Supplement: Additional file 2 — Table S1. List of the 2211 genes that caused lethality and developmental abnormalities when knocked-down by RNAi. [file 1471-2164-13-91-S2.DOC]

**Supplementary Table 1.** List of the 2211 genes that caused lethality and developmental abnormalities when knocked-down by RNAi.

| **Gene ID** | **Gene name** |
| --- | --- |
| T01C8.1 | *aak-2* |
| C56E6.5 | *abch-1* |
| M79.1 | *abl-1* |
| T01D1.6 | *abu-11* |
| AC8.6 | *AC8.6* |
| F47B10.7 | *acbp-3* |
| Y17G7B.1 | *acbp-6* |
| T27E9.9 | *acc-4* |
| C55B7.4 | *acdh-1* |
| F08G5.2 | *acl-13* |
| ZK809.2 | *acl-3* |
| F08F3.2 | *acl-6* |
| Y46G5A.21 | *acl-7* |
| C42D8.5 | *acn-1* |
| T09A5.3 | *acr-7* |
| T04C12.6 | *act-1* |
| T04C12.4 | *act-3* |
| M03F4.2 | *act-4* |
| F17C8.1 | *acy-1* |
| H15N14.1 | *adr-1* |
| Y87G2A.4 | *aex-6* |
| F32A6.4 | *ags-3* |
| F09E5.13 | *agt-2* |
| AH10.2 | *AH10.2* |
| AH6.3 | *AH6.3* |
| C41G7.5 | *ahr-1* |
| C12D8.10 | *akt-1* |
| R10E12.1 | *alx-1* |
| F58H1.1 | *aman-2* |
| B0019.1 | *amx-2* |
| Y71H2B.10 | *apb-1* |
| R11A5.1 | *apb-3* |
| F35G12.9 | *apc-11* |
| ZC434.6 | *aph-2* |
| K04G2.8 | *apr-1* |
| F29G9.3 | *aps-1* |
| F45E4.1 | *arf-1.1* |
| Y116A8C.12 | *arf-6* |
| F31A9.3 | *arg-1* |
| C27A12.8 | *ari-1* |
| F54C9.10 | *arl-1* |
| F19H8.3 | *arl-3* |
| W02B12.6 | *ars-1* |
| C15F1.6 | *art-1* |
| Y71F9AL.16 | *arx-1* |
| Y6D11A.2 | *arx-4* |
| Y37D8A.1 | *arx-5* |
| M01B12.3 | *arx-7* |
| T21G5.5 | *asd-2* |
| T28F4.2 | *asic-2* |
| T08H4.3 | *ast-1* |
| K08F8.2 | *atf-2* |
| F45E6.2 | *atf-6* |
| C07G2.2 | *atf-7* |
| D2085.2 | *atg-10* |
| K06A1.5 | *atg-16.2* |
| Y87G2A.3 | *atg-4.1* |
| K04G2.5 | *ath-1* |
| W04D2.1 | *atn-1* |
| C34E10.6 | *atp-2* |
| B0001.2 | *B0001.2* |
| B0001.4 | *B0001.4* |
| B0024.10 | *B0024.10* |
| B0024.4 | *B0024.4* |
| B0034.1 | *B0034.1* |
| B0034.4 | *B0034.4* |
| B0205.9 | *B0205.9* |
| B0207.7 | *B0207.7* |
| B0228.1 | *B0228.1* |
| B0228.7 | *B0228.7* |
| B0244.9 | *B0244.9* |
| B0252.1 | *B0252.1* |
| B0261.1 | *B0261.1* |
| B0261.7 | *B0261.7* |
| B0280.1 | *B0280.1* |
| B0284.1 | *B0284.1* |
| B0285.3 | *B0285.3* |
| B0286.4 | *B0286.4* |
| B0310.1 | *B0310.1* |
| B0334.3 | *B0334.3* |
| B0334.5 | *B0334.5* |
| B0336.10 | *B0336.10* |
| B0336.2 | *B0336.2* |
| B0353.1 | *B0353.1* |
| B0361.9 | *B0361.9* |
| B0379.2 | *B0379.2* |
| B0379.7 | *B0379.7* |
| B0393.4 | *B0393.4* |
| B0395.3 | *B0395.3* |
| B0432.1 | *B0432.1* |
| B0432.8 | *B0432.8* |
| B0454.9 | *B0454.9* |
| B0478.3 | *B0478.3* |
| B0491.6 | *B0491.6* |
| B0495.9 | *B0495.9* |
| B0496.5 | *B0496.5* |
| B0511.5 | *B0511.5* |
| B0511.7 | *B0511.7* |
| B0513.4 | *B0513.4* |
| B0513.7 | *B0513.7* |
| B0546.1 | *B0546.1* |
| Y71F9AR.1 | *bam-2* |
| F59H6.9 | *bath-1* |
| F52C6.7 | *bath-11* |
| F59H5.3 | *bath-12* |
| F14D2.12 | *bath-30* |
| W02A11.5 | *bath-34* |
| W02A11.8 | *bath-35* |
| Y75B12B.4 | *bath-36* |
| T16H12.5 | *bath-43* |
| T07H3.1 | *bath-47* |
| F52C6.10 | *bath-7* |
| Y105E8A.5 | *bbs-1* |
| R01H10.6 | *bbs-5* |
| T25F10.5 | *bbs-8* |
| Y46G5A.24 | *bcmo-1* |
| BE10.1 | *BE10.1* |
| C50B8.2 | *bir-2* |
| K04F10.4 | *bli-4* |
| F25D7.3 | *blmp-1* |
| F40G9.4 | *btb-1* |
| ZC204.3 | *btb-12* |
| F57C2.2 | *btb-19* |
| F45C12.12 | *btb-7* |
| F45C12.6 | *btb-8* |
| C01A2.2 | *C01A2.2* |
| C01B12.4 | *C01B12.4* |
| C01B12.7 | *C01B12.7* |
| C01B4.8 | *C01B4.8* |
| C01F1.2 | *C01F1.2* |
| C01F4.2 | *C01F4.2* |
| C01G10.7 | *C01G10.7* |
| C01G5.9 | *C01G5.9* |
| C01G6.7 | *C01G6.7* |
| C01G6.9 | *C01G6.9* |
| C01G8.6 | *C01G8.6* |
| C01H6.6 | *C01H6.6* |
| C01H6.9 | *C01H6.9* |
| C02B10.6 | *C02B10.6* |
| C02F4.3 | *C02F4.3* |
| C02F5.5 | *C02F5.5* |
| C03B1.4 | *C03B1.4* |
| C03B1.7 | *C03B1.7* |
| C03C10.4 | *C03C10.4* |
| C03D6.1 | *C03D6.1* |
| C04C3.6 | *C04C3.6* |
| C04E6.7 | *C04E6.7* |
| C04F12.1 | *C04F12.1* |
| C04F12.2 | *C04F12.2* |
| C04G2.10 | *C04G2.10* |
| C04H5.1 | *C04H5.1* |
| C05C12.5 | *C05C12.5* |
| C05D10.1 | *C05D10.1* |
| C05D10.2 | *C05D10.2* |
| C05D11.10 | *C05D11.10* |
| C05D11.13 | *C05D11.13* |
| C05D12.2 | *C05D12.2* |
| C05D12.5 | *C05D12.5* |
| C05D9.3 | *C05D9.3* |
| C05G6.2 | *C05G6.2* |
| C06A8.1 | *C06A8.1* |
| C06A8.2 | *C06A8.2* |
| C06B8.5 | *C06B8.5* |
| C06C3.7 | *C06C3.7* |
| C06C3.8 | *C06C3.8* |
| C06E1.1 | *C06E1.1* |
| C06E1.2 | *C06E1.2* |
| C07A12.7 | *C07A12.7* |
| C07A9.8 | *C07A9.8* |
| C07E3.4 | *C07E3.4* |
| C07F11.2 | *C07F11.2* |
| C07G1.6 | *C07G1.6* |
| C07H6.3 | *C07H6.3* |
| C08H9.1 | *C08H9.1* |
| C08H9.3 | *C08H9.3* |
| C09B9.1 | *C09B9.1* |
| C09B9.2 | *C09B9.2* |
| C09D4.2 | *C09D4.2* |
| C09D4.4 | *C09D4.4* |
| C09F9.1 | *C09F9.1* |
| C09F9.2 | *C09F9.2* |
| C09G5.2 | *C09G5.2* |
| C09G9.2 | *C09G9.2* |
| C10C5.1 | *C10C5.1* |
| C10C5.2 | *C10C5.2* |
| C10G8.8 | *C10G8.8* |
| C12D8.1 | *C12D8.1* |
| C13A10.2 | *C13A10.2* |
| C13B4.1 | *C13B4.1* |
| C13B9.4 | *C13B9.4* |
| C14A4.13 | *C14A4.13* |
| C14A4.3 | *C14A4.3* |
| C14B1.2 | *C14B1.2* |
| C14B1.3 | *C14B1.3* |
| C14C10.4 | *C14C10.4* |
| C14C6.12 | *C14C6.12* |
| C14F11.2 | *C14F11.2* |
| C15A11.4 | *C15A11.4* |
| C15A11.7 | *C15A11.7* |
| C15C6.1 | *C15C6.1* |
| C15C6.3 | *C15C6.3* |
| C15C7.4 | *C15C7.4* |
| C15C8.1 | *C15C8.1* |
| C16A11.5 | *C16A11.5* |
| C16C8.2 | *C16C8.2* |
| C16C8.6 | *C16C8.6* |
| C16C8.9 | *C16C8.9* |
| C16D9.5 | *C16D9.5* |
| C16E9.2 | *C16E9.2* |
| C17C3.5 | *C17C3.5* |
| C17E4.9 | *C17E4.9* |
| C17G10.7 | *C17G10.7* |
| C18A3.7 | *C18A3.7* |
| C18E3.1 | *C18E3.1* |
| C18E9.6 | *C18E9.6* |
| C18F10.7 | *C18F10.7* |
| C18H7.9 | *C18H7.9* |
| C18H9.5 | *C18H9.5* |
| C23G10.6 | *C23G10.6* |
| C23G10.8 | *C23G10.8* |
| C23H3.5 | *C23H3.5* |
| C24A1.2 | *C24A1.2* |
| C24D10.2 | *C24D10.2* |
| C24D10.6 | *C24D10.6* |
| C24G6.3 | *C24G6.3* |
| C24H12.8 | *C24H12.8* |
| C25A1.13 | *C25A1.13* |
| C25E10.5 | *C25E10.5* |
| C26C6.8 | *C26C6.8* |
| C26F1.1 | *C26F1.1* |
| C26F1.3 | *C26F1.3* |
| C27A12.6 | *C27A12.6* |
| C27A12.9 | *C27A12.9* |
| C27F2.1 | *C27F2.1* |
| C27F2.6 | *C27F2.6* |
| C28C12.4 | *C28C12.4* |
| C28H8.1 | *C28H8.1* |
| C30B5.2 | *C30B5.2* |
| C30C11.1 | *C30C11.1* |
| C30F12.1 | *C30F12.1* |
| C30F12.2 | *C30F12.2* |
| C30F12.7 | *C30F12.7* |
| C30H7.2 | *C30H7.2* |
| C31B8.12 | *C31B8.12* |
| C31C9.2 | *C31C9.2* |
| C31E10.7 | *C31E10.7* |
| C31H5.1 | *C31H5.1* |
| C31H5.5 | *C31H5.5* |
| C32B5.13 | *C32B5.13* |
| C32B5.14 | *C32B5.14* |
| C32D5.1 | *C32D5.1* |
| C32D5.10 | *C32D5.10* |
| C32D5.7 | *C32D5.7* |
| C32E12.1 | *C32E12.1* |
| C32E8.5 | *C32E8.5* |
| C32E8.6 | *C32E8.6* |
| C32F10.8 | *C32F10.8* |
| C32H11.3 | *C32H11.3* |
| C32H11.5 | *C32H11.5* |
| C32H11.6 | *C32H11.6* |
| C33A12.7 | *C33A12.7* |
| C33B4.2 | *C33B4.2* |
| C33C12.3 | *C33C12.3* |
| C33C12.8 | *C33C12.8* |
| C33F10.2 | *C33F10.2* |
| C33H5.7 | *C33H5.7* |
| C34B2.10 | *C34B2.10* |
| C34B2.3 | *C34B2.3* |
| C34B2.6 | *C34B2.6* |
| C34B7.2 | *C34B7.2* |
| C34C12.2 | *C34C12.2* |
| C34C6.3 | *C34C6.3* |
| C34D4.3 | *C34D4.3* |
| C35A5.6 | *C35A5.6* |
| C35D10.12 | *C35D10.12* |
| C35E7.10 | *C35E7.10* |
| C35E7.3 | *C35E7.3* |
| C36A4.5 | *C36A4.5* |
| C36A4.9 | *C36A4.9* |
| C36B1.8 | *C36B1.8* |
| C36E8.1 | *C36E8.1* |
| C36F7.2 | *C36F7.2* |
| C36F7.5 | *C36F7.5* |
| C37A2.8 | *C37A2.8* |
| C37C3.2 | *C37C3.2* |
| C38D4.1 | *C38D4.1* |
| C39D10.3 | *C39D10.3* |
| C39D10.5 | *C39D10.5* |
| C39E9.8 | *C39E9.8* |
| C40H1.6 | *C40H1.6* |
| C41D11.6 | *C41D11.6* |
| C41H7.4 | *C41H7.4* |
| C42C1.3 | *C42C1.3* |
| C42D4.1 | *C42D4.1* |
| C42D4.3 | *C42D4.3* |
| C43G2.3 | *C43G2.3* |
| C44B7.2 | *C44B7.2* |
| C44B7.5 | *C44B7.5* |
| C44F1.1 | *C44F1.1* |
| C45G9.12 | *C45G9.12* |
| C45G9.2 | *C45G9.2* |
| C46A5.6 | *C46A5.6* |
| C46A5.8 | *C46A5.8* |
| C46E10.4 | *C46E10.4* |
| C46G7.2 | *C46G7.2* |
| C46H11.10 | *C46H11.10* |
| C46H11.2 | *C46H11.2* |
| C47B2.2 | *C47B2.2* |
| C47D12.8 | *C47D12.8* |
| C47E12.10 | *C47E12.10* |
| C48B4.3 | *C48B4.3* |
| C48B4.7 | *C48B4.7* |
| C48B6.9 | *C48B6.9* |
| C48C5.1 | *C48C5.1* |
| C48E7.7 | *C48E7.7* |
| C48E7.8 | *C48E7.8* |
| C49A1.3 | *C49A1.3* |
| C49A9.6 | *C49A9.6* |
| C49A9.7 | *C49A9.7* |
| C49C8.3 | *C49C8.3* |
| C49C8.6 | *C49C8.6* |
| C49G7.3 | *C49G7.3* |
| C49G7.5 | *C49G7.5* |
| C49H3.8 | *C49H3.8* |
| C50A2.2 | *C50A2.2* |
| C50A2.3 | *C50A2.3* |
| C50B6.3 | *C50B6.3* |
| C50D2.3 | *C50D2.3* |
| C50E10.1 | *C50E10.1* |
| C50F2.5 | *C50F2.5* |
| C50F2.8 | *C50F2.8* |
| C50F7.3 | *C50F7.3* |
| C52A10.1 | *C52A10.1* |
| C53D5.1 | *C53D5.1* |
| C53D5.2 | *C53D5.2* |
| C53D5.5 | *C53D5.5* |
| C54G6.1 | *C54G6.1* |
| C55A6.6 | *C55A6.6* |
| C56C10.6 | *C56C10.6* |
| C56E6.4 | *C56E6.4* |
| W03H9.4 | *cacn-1* |
| M02B7.6 | *cal-3* |
| T07G12.1 | *cal-4* |
| C01G6.8 | *cam-1* |
| B0034.3 | *casy-1* |
| W01C8.6 | *cat-1* |
| F32G8.6 | *cat-4* |
| K01A2.11 | *cbn-1* |
| CC8.1 | *CC8.1* |
| T05C12.7 | *cct-1* |
| K01C8.10 | *cct-4* |
| C07G2.3 | *cct-5* |
| T10B5.5 | *cct-7* |
| Y55F3AR.3 | *cct-8* |
| C17G10.4 | *cdc-14* |
| K04G2.3 | *cdc-48.3* |
| F49E8.4 | *cdd-2* |
| R05H10.6 | *cdh-7* |
| T27E9.3 | *cdk-5* |
| F39H11.3 | *cdk-8* |
| H25P06.2 | *cdk-9* |
| T23F11.3 | *cdka-1* |
| C09G12.8 | *ced-10* |
| Y106G6E.5 | *ced-12* |
| F20D12.6 | *ceh-19* |
| F46F3.1 | *ceh-27* |
| C28A5.4 | *ceh-43* |
| C03D6.3 | *cel-1* |
| F33A8.3 | *cey-1* |
| T23D8.8 | *cfi-1* |
| C07H6.5 | *cgh-1* |
| T06C12.10 | *cgt-1* |
| C55B7.12 | *che-1* |
| F56H1.1 | *che-14* |
| Y110A7A.13 | *chp-1* |
| F22E12.2 | *chw-1* |
| F55F8.4 | *cir-1* |
| Y18D10A.10 | *clec-104* |
| C54C8.7 | *clec-11* |
| F47G4.1 | *clec-113* |
| W10G11.15 | *clec-129* |
| F35D11.7 | *clec-136* |
| C04H5.2 | *clec-147* |
| F10F2.8 | *clec-153* |
| F26A1.12 | *clec-157* |
| E03H4.10 | *clec-17* |
| ZK896.6 | *clec-187* |
| C49C3.12 | *clec-197* |
| C49C3.13 | *clec-198* |
| C41H7.7 | *clec-3* |
| R07C3.1 | *clec-43* |
| T09F5.9 | *clec-47* |
| T03F1.10 | *clec-53* |
| ZK666.3 | *clec-58* |
| F35C5.5 | *clec-62* |
| F35C5.9 | *clec-66* |
| Y46C8AL.1 | *clec-73* |
| Y54G2A.14 | *clec-83* |
| ZK858.3 | *clec-91* |
| ZK39.2 | *clec-95* |
| C07H4.2 | *clh-5* |
| C06G4.2 | *clp-1* |
| F56B3.1 | *col-103* |
| Y42H9B.1 | *col-115* |
| F55C10.2 | *col-154* |
| ZK1193.1 | *col-19* |
| F11G11.11 | *col-20* |
| C15A11.1 | *col-35* |
| F54C9.4 | *col-38* |
| F53G12.7 | *col-45* |
| Y54E10BL.2 | *col-48* |
| T28F2.6 | *col-50* |
| T08B2.2 | *col-56* |
| C01H6.1 | *col-61* |
| F52F12.2 | *col-64* |
| K01A2.7 | *col-69* |
| C09G5.5 | *col-80* |
| Y81G3A.5 | *col-86* |
| Y57G11C.11 | *coq-3* |
| C35D10.4 | *coq-8* |
| C40H1.1 | *cpb-1* |
| D1069.2 | *cpn-2* |
| C25B8.3 | *cpr-6* |
| Y41C4A.4 | *crh-1* |
| K07G5.1 | *crml-1* |
| C14A4.4 | *crn-3* |
| T20G5.2 | *cts-1* |
| Y76A2A.2 | *cua-1* |
| ZK856.1 | *cul-5* |
| F59H6.7 | *cya-2* |
| T06E6.2 | *cyb-3* |
| E04A4.7 | *cyc-2.1* |
| Y49F6B.1 | *cyh-1* |
| K08E3.6 | *cyk-4* |
| T01B7.4 | *cyn-11* |
| C34D4.12 | *cyn-12* |
| Y87G2A.6 | *cyn-15* |
| Y17G7B.9 | *cyn-16* |
| F59E10.2 | *cyn-4* |
| F31C3.1 | *cyn-5* |
| T10B9.7 | *cyp-13A2* |
| T10B9.5 | *cyp-13A3* |
| T10B9.10 | *cyp-13A7* |
| T10B9.4 | *cyp-13A8* |
| T19B10.1 | *cyp-29A2* |
| Y17G9B.3 | *cyp-31A3* |
| C49C8.4 | *cyp-33E1* |
| C34B7.3 | *cyp-36A1* |
| F20D12.4 | *czw-1* |
| D1005.1 | *D1005.1* |
| D1007.10 | *D1007.10* |
| D1007.15 | *D1007.15* |
| D1007.3 | *D1007.3* |
| D1022.2 | *D1022.2* |
| D1037.1 | *D1037.1* |
| D1043.1 | *D1043.1* |
| D1054.9 | *D1054.9* |
| D1069.3 | *D1069.3* |
| D1081.9 | *D1081.9* |
| D1086.1 | *D1086.1* |
| D1086.2 | *D1086.2* |
| D1086.5 | *D1086.5* |
| D2023.6 | *D2023.6* |
| D2030.1 | *D2030.1* |
| D2030.3 | *D2030.3* |
| D2030.4 | *D2030.4* |
| D2030.7 | *D2030.7* |
| D2045.5 | *D2045.5* |
| D2062.1 | *D2062.1* |
| D2063.1 | *D2063.1* |
| D2089.2 | *D2089.2* |
| D2092.1 | *D2092.1* |
| D2096.9 | *D2096.9* |
| B0412.1 | *dac-1* |
| R13H8.1 | *daf-16* |
| R05D11.1 | *daf-8* |
| K12C11.4 | *dapk-1* |
| ZC477.9 | *deb-1* |
| K03B8.9 | *deg-3* |
| T28B8.5 | *del-4* |
| F44G4.8 | *dep-1* |
| T26H10.1 | *des-2* |
| F46H6.2 | *dgk-2* |
| T21B6.1 | *dgn-1* |
| DH11.5 | *DH11.5* |
| R06C7.3 | *dhp-1* |
| C01G8.3 | *dhs-1* |
| K08F4.9 | *dhs-12* |
| C10F3.2 | *dhs-16* |
| T05F1.10 | *dhs-4* |
| K10H10.3 | *dhs-8* |
| C18D1.1 | *die-1* |
| W09C5.5 | *dkf-1* |
| T25E12.4 | *dkf-2* |
| T26A5.9 | *dlc-1* |
| F33E2.2 | *dlk-1* |
| F10C1.5 | *dmd-5* |
| F38A5.13 | *dnj-11* |
| T05C3.5 | *dnj-19* |
| B0035.2 | *dnj-2* |
| T19B4.4 | *dnj-21* |
| Y39C12A.8 | *dnj-26* |
| Y63D3A.6 | *dnj-29* |
| C01G8.4 | *dnj-4* |
| K10D11.1 | *dod-17* |
| Y66H1A.2 | *dpm-1* |
| F46E10.9 | *dpy-11* |
| Y39A1B.3 | *dpy-28* |
| F27C1.8 | *dpy-5* |
| F46C8.6 | *dpy-7* |
| C54A12.4 | *drn-1* |
| B0464.1 | *drs-1* |
| F10C2.6 | *drs-2* |
| F26E4.10 | *drsh-1* |
| W09G12.4 | *dsl-1* |
| Y106G6H.12 | *duo-3* |
| K07A1.2 | *dut-1* |
| C33G3.1 | *dyc-1* |
| D1009.5 | *dylt-2* |
| C02C6.1 | *dyn-1* |
| T24H10.6 | *dyrb-1* |
| F15D3.1 | *dys-1* |
| E01A2.1 | *E01A2.1* |
| E01G4.2 | *E01G4.2* |
| E02H1.1 | *E02H1.1* |
| E02H1.2 | *E02H1.2* |
| E03H12.5 | *E03H12.5* |
| E03H4.1 | *E03H4.1* |
| E03H4.2 | *E03H4.2* |
| E03H4.8 | *E03H4.8* |
| E04A4.5 | *E04A4.5* |
| E04F6.10 | *E04F6.10* |
| E04F6.5 | *E04F6.5* |
| E04F6.9 | *E04F6.9* |
| Y48B6A.4 | *eat-2* |
| B0365.3 | *eat-6* |
| Y59A8B.7 | *ebp-1* |
| T05G5.6 | *ech-6* |
| T19E10.1 | *ect-2* |
| R05D11.8 | *edc-3* |
| EEED8.3 | *EEED8.3* |
| Y48C3A.17 | *efl-2* |
| ZK328.2 | *eft-1* |
| F55A8.1 | *egl-18* |
| C51E3.7 | *egl-3* |
| R07A4.1 | *egl-36* |
| F55A8.2 | *egl-4* |
| R53.3 | *egl-43* |
| C27D11.1 | *egl-45* |
| ZK1248.3 | *ehs-1* |
| B0511.10 | *eif-3.E* |
| C47B2.5 | *eif-6* |
| F22D6.6 | *ekl-1* |
| Y105E8A.17 | *ekl-4* |
| F38A6.2 | *elp-1* |
| C33D3.1 | *elt-2* |
| F10B5.6 | *emb-27* |
| K10D2.6 | *emb-8* |
| Y111B2A.11 | *epc-1* |
| ZC434.5 | *ers-2* |
| T01D1.2 | *etr-1* |
| C42D8.4 | *ets-5* |
| F22B5.1 | *evl-20* |
| C43E11.8 | *exoc-7* |
| Y105E8B.2 | *exoc-8* |
| F01D5.1 | *F01D5.1* |
| F01D5.2 | *F01D5.2* |
| F01D5.3 | *F01D5.3* |
| F01D5.5 | *F01D5.5* |
| F01D5.7 | *F01D5.7* |
| F01G10.6 | *F01G10.6* |
| F01G4.6 | *F01G4.6* |
| F02D10.6 | *F02D10.6* |
| F02E9.5 | *F02E9.5* |
| F08G12.1 | *F08G12.1* |
| F08G2.7 | *F08G2.7* |
| F08G5.3 | *F08G5.3* |
| F09C12.6 | *F09C12.6* |
| F09D1.1 | *F09D1.1* |
| F09E10.6 | *F09E10.6* |
| F09E5.11 | *F09E5.11* |
| F09F7.5 | *F09F7.5* |
| F10D11.2 | *F10D11.2* |
| F10D11.5 | *F10D11.5* |
| F10E7.5 | *F10E7.5* |
| F10E7.6 | *F10E7.6* |
| F10F2.4 | *F10F2.4* |
| F10G8.2 | *F10G8.2* |
| F11E6.3 | *F11E6.3* |
| F11F1.1 | *F11F1.1* |
| F11F1.2 | *F11F1.2* |
| F11F1.5 | *F11F1.5* |
| F11F1.6 | *F11F1.6* |
| F11G11.4 | *F11G11.4* |
| F12A10.1 | *F12A10.1* |
| F12A10.5 | *F12A10.5* |
| F12F6.7 | *F12F6.7* |
| F13B6.1 | *F13B6.1* |
| F13C5.2 | *F13C5.2* |
| F13G3.3 | *F13G3.3* |
| F13H10.1 | *F13H10.1* |
| F13H8.9 | *F13H8.9* |
| F14B4.2 | *F14B4.2* |
| F14B4.3 | *F14B4.3* |
| F14B6.5 | *F14B6.5* |
| F14D2.14 | *F14D2.14* |
| F14E5.2 | *F14E5.2* |
| F14F7.5 | *F14F7.5* |
| F15C11.2 | *F15C11.2* |
| F15D3.4 | *F15D3.4* |
| F15D3.8 | *F15D3.8* |
| F15H9.1 | *F15H9.1* |
| F16C3.1 | *F16C3.1* |
| F16G10.13 | *F16G10.13* |
| F16H6.5 | *F16H6.5* |
| F17B5.1 | *F17B5.1* |
| F17C8.6 | *F17C8.6* |
| F17E9.5 | *F17E9.5* |
| F18A11.2 | *F18A11.2* |
| F18A11.6 | *F18A11.6* |
| F18A12.1 | *F18A12.1* |
| F18A12.3 | *F18A12.3* |
| F18A12.5 | *F18A12.5* |
| F18A12.8 | *F18A12.8* |
| F18C12.3 | *F18C12.3* |
| F19B6.3 | *F19B6.3* |
| F19C7.3 | *F19C7.3* |
| F20A1.6 | *F20A1.6* |
| F20C5.4 | *F20C5.4* |
| F20D1.9 | *F20D1.9* |
| F20D6.10 | *F20D6.10* |
| F20H11.1 | *F20H11.1* |
| F21D5.7 | *F21D5.7* |
| F21D5.8 | *F21D5.8* |
| F21D9.4 | *F21D9.4* |
| F21F3.1 | *F21F3.1* |
| F21H7.2 | *F21H7.2* |
| F21H7.3 | *F21H7.3* |
| F22B7.1 | *F22B7.1* |
| F22E5.12 | *F22E5.12* |
| F22E5.9 | *F22E5.9* |
| F22F7.1 | *F22F7.1* |
| F23B12.5 | *F23B12.5* |
| F23C8.6 | *F23C8.6* |
| F23F1.5 | *F23F1.5* |
| F23F12.3 | *F23F12.3* |
| F23F12.7 | *F23F12.7* |
| F23F12.8 | *F23F12.8* |
| F23H12.3 | *F23H12.3* |
| F25B4.6 | *F25B4.6* |
| F25B5.3 | *F25B5.3* |
| F25E5.3 | *F25E5.3* |
| F25G6.7 | *F25G6.7* |
| F25H2.6 | *F25H2.6* |
| F25H5.5 | *F25H5.5* |
| F25H5.7 | *F25H5.7* |
| F25H9.7 | *F25H9.7* |
| F26A1.13 | *F26A1.13* |
| F26A1.6 | *F26A1.6* |
| F26A1.8 | *F26A1.8* |
| F26A3.1 | *F26A3.1* |
| F26A3.7 | *F26A3.7* |
| F26E4.4 | *F26E4.4* |
| F26E4.5 | *F26E4.5* |
| F26F4.6 | *F26F4.6* |
| F26G1.5 | *F26G1.5* |
| F26G1.6 | *F26G1.6* |
| F26H11.3 | *F26H11.3* |
| F26H11.4 | *F26H11.4* |
| F26H9.8 | *F26H9.8* |
| F27C1.1 | *F27C1.1* |
| F27C1.6 | *F27C1.6* |
| F27C8.2 | *F27C8.2* |
| F27D4.1 | *F27D4.1* |
| F27D4.2 | *F27D4.2* |
| F27D4.4 | *F27D4.4* |
| F28A10.7 | *F28A10.7* |
| F28C6.5 | *F28C6.5* |
| F28H1.4 | *F28H1.4* |
| F28H7.7 | *F28H7.7* |
| F29B9.1 | *F29B9.1* |
| F29B9.10 | *F29B9.10* |
| F29B9.7 | *F29B9.7* |
| F29C12.2 | *F29C12.2* |
| F29D10.1 | *F29D10.1* |
| F30F8.4 | *F30F8.4* |
| F31C3.2 | *F31C3.2* |
| F31D5.2 | *F31D5.2* |
| F32A11.1 | *F32A11.1* |
| F32A11.3 | *F32A11.3* |
| F32A7.5 | *F32A7.5* |
| F32B4.4 | *F32B4.4* |
| F32D1.7 | *F32D1.7* |
| F32D8.1 | *F32D8.1* |
| F32D8.10 | *F32D8.10* |
| F32D8.5 | *F32D8.5* |
| F32H2.7 | *F32H2.7* |
| F33D11.1 | *F33D11.1* |
| F33H2.2 | *F33H2.2* |
| F35B12.4 | *F35B12.4* |
| F35C5.1 | *F35C5.1* |
| F35C5.3 | *F35C5.3* |
| F35E2.5 | *F35E2.5* |
| F35F11.1 | *F35F11.1* |
| F35H12.4 | *F35H12.4* |
| F35H8.2 | *F35H8.2* |
| F36A2.11 | *F36A2.11* |
| F36A2.7 | *F36A2.7* |
| F36H12.1 | *F36H12.1* |
| F36H12.14 | *F36H12.14* |
| F36H12.2 | *F36H12.2* |
| F36H5.4 | *F36H5.4* |
| F37A4.3 | *F37A4.3* |
| F37A4.6 | *F37A4.6* |
| F37A8.1 | *F37A8.1* |
| F37C12.7 | *F37C12.7* |
| F37C4.3 | *F37C4.3* |
| F37C4.4 | *F37C4.4* |
| F37C4.6 | *F37C4.6* |
| F38A1.9 | *F38A1.9* |
| F38B2.4 | *F38B2.4* |
| F38E11.5 | *F38E11.5* |
| F38H4.5 | *F38H4.5* |
| F39B2.5 | *F39B2.5* |
| F39B2.8 | *F39B2.8* |
| F40A3.6 | *F40A3.6* |
| F40F12.7 | *F40F12.7* |
| F40G12.11 | *F40G12.11* |
| F40G12.5 | *F40G12.5* |
| F40G9.9 | *F40G9.9* |
| F40H3.1 | *F40H3.1* |
| F41C3.11 | *F41C3.11* |
| F41C3.4 | *F41C3.4* |
| F41D3.4 | *F41D3.4* |
| F41D3.5 | *F41D3.5* |
| F41E6.11 | *F41E6.11* |
| F41G3.1 | *F41G3.1* |
| F42A10.5 | *F42A10.5* |
| F42A10.7 | *F42A10.7* |
| F42A9.3 | *F42A9.3* |
| F42F12.3 | *F42F12.3* |
| F42G8.10 | *F42G8.10* |
| F42G8.5 | *F42G8.5* |
| F42H10.4 | *F42H10.4* |
| F42H10.5 | *F42H10.5* |
| F42H10.6 | *F42H10.6* |
| F43G6.7 | *F43G6.7* |
| F43G6.8 | *F43G6.8* |
| F43G9.1 | *F43G9.1* |
| F43G9.2 | *F43G9.2* |
| F43G9.4 | *F43G9.4* |
| F43H9.3 | *F43H9.3* |
| F44D12.1 | *F44D12.1* |
| F44D12.6 | *F44D12.6* |
| F44E2.6 | *F44E2.6* |
| F44E2.7 | *F44E2.7* |
| F44E2.9 | *F44E2.9* |
| F44E7.5 | *F44E7.5* |
| F44F1.6 | *F44F1.6* |
| F44F4.1 | *F44F4.1* |
| F44F4.10 | *F44F4.10* |
| F44G4.3 | *F44G4.3* |
| F45D11.1 | *F45D11.1* |
| F45D11.16 | *F45D11.16* |
| F45E1.3 | *F45E1.3* |
| F45E12.5 | *F45E12.5* |
| F45G2.7 | *F45G2.7* |
| F45G2.8 | *F45G2.8* |
| F45H10.3 | *F45H10.3* |
| F46A8.9 | *F46A8.9* |
| F46A9.1 | *F46A9.1* |
| F46C5.7 | *F46C5.7* |
| F46C5.9 | *F46C5.9* |
| F46C8.3 | *F46C8.3* |
| F46F11.1 | *F46F11.1* |
| F46F11.9 | *F46F11.9* |
| F46F5.11 | *F46F5.11* |
| F46F5.6 | *F46F5.6* |
| F46F5.7 | *F46F5.7* |
| F46F5.9 | *F46F5.9* |
| F47B3.7 | *F47B3.7* |
| F47B8.2 | *F47B8.2* |
| F47G4.2 | *F47G4.2* |
| F47G4.4 | *F47G4.4* |
| F47G4.5 | *F47G4.5* |
| F47G6.3 | *F47G6.3* |
| F47G9.4 | *F47G9.4* |
| F48C1.5 | *F48C1.5* |
| F48C1.6 | *F48C1.6* |
| F48E8.4 | *F48E8.4* |
| F48F7.6 | *F48F7.6* |
| F49C12.2 | *F49C12.2* |
| F49C12.4 | *F49C12.4* |
| F52B11.2 | *F52B11.2* |
| F52B5.2 | *F52B5.2* |
| F52F12.7 | *F52F12.7* |
| F52H2.6 | *F52H2.6* |
| F52H2.7 | *F52H2.7* |
| F53A3.1 | *F53A3.1* |
| F53A9.8 | *F53A9.8* |
| F53B1.3 | *F53B1.3* |
| F53C3.3 | *F53C3.3* |
| F53C3.4 | *F53C3.4* |
| F53E10.6 | *F53E10.6* |
| F53F10.1 | *F53F10.1* |
| F53F10.2 | *F53F10.2* |
| F53F4.10 | *F53F4.10* |
| F53F4.3 | *F53F4.3* |
| F53H1.3 | *F53H1.3* |
| F53H4.5 | *F53H4.5* |
| F54A3.1 | *F54A3.1* |
| F54A5.2 | *F54A5.2* |
| F54C4.4 | *F54C4.4* |
| F54D12.4 | *F54D12.4* |
| F54D12.5 | *F54D12.5* |
| F54D5.12 | *F54D5.12* |
| F54D5.15 | *F54D5.15* |
| F54D5.4 | *F54D5.4* |
| F54D7.2 | *F54D7.2* |
| F54E2.2 | *F54E2.2* |
| F54F11.1 | *F54F11.1* |
| F54F2.9 | *F54F2.9* |
| F54F3.4 | *F54F3.4* |
| F54G2.1 | *F54G2.1* |
| F54H12.6 | *F54H12.6* |
| F54H5.2 | *F54H5.2* |
| F55A11.8 | *F55A11.8* |
| F55A12.2 | *F55A12.2* |
| F55A12.5 | *F55A12.5* |
| F55A4.5 | *F55A4.5* |
| F55B11.2 | *F55B11.2* |
| F55B12.4 | *F55B12.4* |
| F55C5.2 | *F55C5.2* |
| F55C5.8 | *F55C5.8* |
| F55D12.2 | *F55D12.2* |
| F55D12.5 | *F55D12.5* |
| F55F10.1 | *F55F10.1* |
| F55F3.3 | *F55F3.3* |
| F55F8.3 | *F55F8.3* |
| F55F8.9 | *F55F8.9* |
| F55G1.5 | *F55G1.5* |
| F55G1.7 | *F55G1.7* |
| F55G11.1 | *F55G11.1* |
| F55G11.2 | *F55G11.2* |
| F56B3.6 | *F56B3.6* |
| F56B3.8 | *F56B3.8* |
| F56C11.6 | *F56C11.6* |
| F56D2.6 | *F56D2.6* |
| F56D3.1 | *F56D3.1* |
| F56F11.1 | *F56F11.1* |
| F56F11.2 | *F56F11.2* |
| F56F3.4 | *F56F3.4* |
| F56F4.3 | *F56F4.3* |
| F56G4.4 | *F56G4.4* |
| F56G4.6 | *F56G4.6* |
| F57A8.4 | *F57A8.4* |
| F57C9.1 | *F57C9.1* |
| F57C9.7 | *F57C9.7* |
| F58D5.7 | *F58D5.7* |
| F58E1.11 | *F58E1.11* |
| F58E1.12 | *F58E1.12* |
| F58E6.1 | *F58E6.1* |
| F58F6.6 | *F58F6.6* |
| F58F9.1 | *F58F9.1* |
| F58F9.4 | *F58F9.4* |
| F58F9.6 | *F58F9.6* |
| F58G1.3 | *F58G1.3* |
| F58G1.7 | *F58G1.7* |
| F58H1.2 | *F58H1.2* |
| F58H10.1 | *F58H10.1* |
| F59A2.3 | *F59A2.3* |
| F59A3.1 | *F59A3.1* |
| F59A3.3 | *F59A3.3* |
| F59A6.4 | *F59A6.4* |
| F59A6.5 | *F59A6.5* |
| F59B10.3 | *F59B10.3* |
| F59B10.6 | *F59B10.6* |
| F59C6.2 | *F59C6.2* |
| F59E12.6 | *F59E12.6* |
| F59E12.9 | *F59E12.9* |
| F59H5.1 | *F59H5.1* |
| F59H6.5 | *F59H6.5* |
| F02A9.2 | *far-1* |
| W06D12.3 | *fat-5* |
| K07A3.1 | *fbp-1* |
| F08A8.7 | *fbxa-101* |
| Y59A8B.11 | *fbxa-106* |
| Y113G7B.7 | *fbxa-114* |
| F59A1.8 | *fbxa-129* |
| C08E3.8 | *fbxa-165* |
| C31C9.3 | *fbxa-167* |
| C17B7.11 | *fbxa-65* |
| T12B5.11 | *fbxa-67* |
| F54B8.3 | *fbxa-69* |
| C08F11.5 | *fbxa-98* |
| Y63D3A.2 | *fbxb-100* |
| F08D12.11 | *fbxb-111* |
| F58E1.9 | *fbxb-19* |
| Y56A3A.10 | *fbxb-22* |
| M01D1.9 | *fbxb-40* |
| M01D1.8 | *fbxb-41* |
| Y51H7BR.1 | *fbxb-42* |
| F36H5.5 | *fbxb-53* |
| Y40B1B.3 | *fbxb-66* |
| F09C3.5 | *fbxb-69* |
| ZK909.5 | *fbxb-70* |
| C46H11.11 | *fhod-1* |
| Y18D10A.19 | *fkb-2* |
| C50F2.6 | *fkb-5* |
| F40H3.4 | *fkh-8* |
| F02D10.5 | *flr-1* |
| K01B6.1 | *fozi-1* |
| F59G1.7 | *frh-1* |
| T04C9.6 | *frm-2* |
| C24A11.8 | *frm-4* |
| T08B2.9 | *frs-1* |
| C54F6.14 | *ftn-1* |
| F59E12.13 | *fut-3* |
| F37B12.2 | *gcs-1* |
| T01A4.1 | *gcy-28* |
| T04D3.4 | *gcy-35* |
| C49H3.1 | *gcy-8* |
| Y57G11C.10 | *gdi-1* |
| F32H2.1 | *gei-11* |
| T17H7.4 | *gei-16* |
| W10D5.3 | *gei-17* |
| W07B3.2 | *gei-4* |
| F56A11.1 | *gex-2* |
| F09C12.1 | *ggr-3* |
| C45G3.3 | *gip-2* |
| F14F3.2 | *git-1* |
| T02E1.3 | *gla-3* |
| F52A8.4 | *glb-18* |
| C06E4.7 | *glb-2* |
| C23H5.2 | *glb-7* |
| T23G11.3 | *gld-1* |
| ZC308.1 | *gld-2* |
| K03H1.1 | *gln-2* |
| Y105C5B.28 | *gln-3* |
| C12D12.2 | *glt-1* |
| Y53C12A.2 | *glt-5* |
| C54C8.11 | *gly-15* |
| Y47D3A.23 | *gly-9* |
| Y75B8A.17 | *gmn-1* |
| T23G11.2 | *gna-2* |
| T01H8.5 | *gon-2* |
| K02A4.2 | *gpc-1* |
| K10B3.8 | *gpd-2* |
| K10B3.7 | *gpd-3* |
| T01B10.2 | *grd-14* |
| ZK512.9 | *grl-11* |
| Y47D7A.5 | *grl-5* |
| K10C2.5 | *grl-6* |
| T02E9.2 | *grl-7* |
| ZC395.6 | *gro-1* |
| R06A10.2 | *gsa-1* |
| K06A4.3 | *gsnl-1* |
| Y48E1B.10 | *gst-20* |
| Y53F4B.31 | *gst-28* |
| C02A12.1 | *gst-33* |
| Y53F4B.33 | *gst-39* |
| F56B3.10 | *gst-40* |
| F13A7.10 | *gst-44* |
| F11G11.1 | *gst-8* |
| ZK1320.1 | *gstk-1* |
| C29E4.7 | *gsto-1* |
| Y46G5A.31 | *gsy-1* |
| H01G02.2 | *H01G02.2* |
| H04M03.11 | *H04M03.11* |
| H06I04.6 | *H06I04.6* |
| H08M01.1 | *H08M01.1* |
| H10E21.4 | *H10E21.4* |
| H12D21.2 | *H12D21.2* |
| H13N06.4 | *H13N06.4* |
| H17B01.1 | *H17B01.1* |
| H20E11.2 | *H20E11.2* |
| H20J04.1 | *H20J04.1* |
| H25P06.1 | *H25P06.1* |
| H27M09.3 | *H27M09.3* |
| H27M09.5 | *H27M09.5* |
| H28O16.2 | *H28O16.2* |
| H32K16.1 | *H32K16.1* |
| H35B03.2 | *H35B03.2* |
| Y50E8A.16 | *haf-7* |
| F40E10.1 | *hch-1* |
| T03F1.9 | *hcp-4* |
| Y51H1A.5 | *hda-6* |
| F21C3.3 | *hint-1* |
| T10C6.14 | *his-1* |
| F17E9.10 | *his-32* |
| F17E9.13 | *his-33* |
| T10C6.11 | *his-4* |
| F45F2.3 | *his-5* |
| F54E12.1 | *his-55* |
| H02I12.6 | *his-66* |
| T23D8.5 | *his-67* |
| T23D8.6 | *his-68* |
| E03A3.4 | *his-70* |
| F45E1.6 | *his-71* |
| Y49E10.6 | *his-72* |
| W05B10.1 | *his-74* |
| F58A4.7 | *hlh-11* |
| T05G5.2 | *hlh-4* |
| T20B12.8 | *hmg-4* |
| M01F1.5 | *hmit-1.3* |
| T21C12.2 | *hpd-1* |
| W02D3.11 | *hrpf-1* |
| T11G6.1 | *hrs-1* |
| F43E2.8 | *hsp-4* |
| C37H5.8 | *hsp-6* |
| C34F6.4 | *hst-2* |
| K05B2.3 | *ifa-4* |
| F54C9.1 | *iff-2* |
| C45G7.2 | *ilys-2* |
| F22A3.6 | *ilys-5* |
| C33H5.11 | *imp-3* |
| C17C3.4 | *ins-11* |
| F08G2.6 | *ins-37* |
| ZK75.1 | *ins-4* |
| ZK84.6 | *ins-6* |
| Y8G1A.2 | *inx-13* |
| F07A5.1 | *inx-14* |
| C41C4.4 | *ire-1* |
| C25A1.7 | *irs-2* |
| F37A4.8 | *isw-1* |
| Y116A8C.36 | *itsn-1* |
| JC8.11 | *JC8.11* |
| JC8.5 | *JC8.5* |
| Y48B6A.11 | *jmjd-2* |
| K01A11.2 | *K01A11.2* |
| K01A6.4 | *K01A6.4* |
| K01C8.6 | *K01C8.6* |
| K01D12.5 | *K01D12.5* |
| K01G5.8 | *K01G5.8* |
| K01H12.2 | *K01H12.2* |
| K01H12.4 | *K01H12.4* |
| K02A2.1 | *K02A2.1* |
| K02B12.3 | *K02B12.3* |
| K02B12.7 | *K02B12.7* |
| K02D10.4 | *K02D10.4* |
| K02E10.6 | *K02E10.6* |
| K02E7.2 | *K02E7.2* |
| K02F2.2 | *K02F2.2* |
| K02F3.10 | *K02F3.10* |
| K02F3.2 | *K02F3.2* |
| K02F3.9 | *K02F3.9* |
| K02F6.9 | *K02F6.9* |
| K03B8.6 | *K03B8.6* |
| K03H1.8 | *K03H1.8* |
| K04A8.3 | *K04A8.3* |
| K04C2.5 | *K04C2.5* |
| K04F10.1 | *K04F10.1* |
| K04F10.3 | *K04F10.3* |
| K04G7.1 | *K04G7.1* |
| K05C4.11 | *K05C4.11* |
| K05C4.2 | *K05C4.2* |
| K05F1.10 | *K05F1.10* |
| K06A1.1 | *K06A1.1* |
| K06A1.2 | *K06A1.2* |
| K06A4.2 | *K06A4.2* |
| K06A5.3 | *K06A5.3* |
| K06A5.6 | *K06A5.6* |
| K06A5.8 | *K06A5.8* |
| K06H7.8 | *K06H7.8* |
| K07A1.9 | *K07A1.9* |
| K07A12.1 | *K07A12.1* |
| K07A12.2 | *K07A12.2* |
| K07A3.3 | *K07A3.3* |
| K07C11.4 | *K07C11.4* |
| K07C5.2 | *K07C5.2* |
| K07C5.3 | *K07C5.3* |
| K07D4.6 | *K07D4.6* |
| K07E8.6 | *K07E8.6* |
| K07H8.8 | *K07H8.8* |
| K08C9.2 | *K08C9.2* |
| K08C9.5 | *K08C9.5* |
| K08D12.4 | *K08D12.4* |
| K08E3.5 | *K08E3.5* |
| K08F8.5 | *K08F8.5* |
| K09B11.10 | *K09B11.10* |
| K09E10.2 | *K09E10.2* |
| K09F6.7 | *K09F6.7* |
| K09G1.1 | *K09G1.1* |
| K10B4.3 | *K10B4.3* |
| K10C3.5 | *K10C3.5* |
| K10D3.4 | *K10D3.4* |
| K10D3.5 | *K10D3.5* |
| K10D6.2 | *K10D6.2* |
| K10H10.6 | *K10H10.6* |
| K11B4.1 | *K11B4.1* |
| K11D9.3 | *K11D9.3* |
| K11H12.9 | *K11H12.9* |
| K11H3.3 | *K11H3.3* |
| K12B6.4 | *K12B6.4* |
| K12H4.4 | *K12H4.4* |
| K12H6.4 | *K12H6.4* |
| K12H6.6 | *K12H6.6* |
| F22D6.1 | *kin-14* |
| T17E9.1 | *kin-18* |
| F46F2.2 | *kin-20* |
| C30F8.4 | *kin-32* |
| Y43F4B.6 | *klp-19* |
| T09A5.2 | *klp-3* |
| T02G5.9 | *krs-1* |
| F58D5.4 | *ksr-2* |
| ZK945.1 | *lact-2* |
| M05D6.4 | *lact-4* |
| C40H5.4 | *lact-7* |
| Y6B3B.10 | *lagr-1* |
| T12F5.5 | *larp-2* |
| W02D3.5 | *lbp-6* |
| F52H3.7 | *lec-2* |
| ZK892.1 | *lec-3* |
| C44F1.3 | *lec-4* |
| R07B1.2 | *lec-7* |
| R07B1.10 | *lec-8* |
| F26D11.11 | *let-413* |
| C01G8.9 | *let-526* |
| ZK792.6 | *let-60* |
| C29E6.1 | *let-653* |
| H19M22.2 | *let-805* |
| C46H11.4 | *lfe-2* |
| ZC8.4 | *lfi-1* |
| B0491.4 | *lgc-20* |
| F58H7.3 | *lgc-30* |
| T27A1.4 | *lgc-34* |
| W10G11.16 | *lgc-45* |
| F12B6.3 | *lgc-51* |
| Y113G7A.5 | *lgc-55* |
| Y57G11C.2 | *lgc-7* |
| K09C8.4 | *lge-1* |
| B0336.8 | *lgg-3* |
| ZK678.1 | *lin-15A* |
| Y71F9B.5 | *lin-17* |
| F18A1.2 | *lin-26* |
| T14F9.5 | *lin-32* |
| E01A2.3 | *lin-44* |
| F14E5.5 | *lips-10* |
| C09E8.2 | *lips-7* |
| R74.1 | *lrs-1* |
| ZK524.3 | *lrs-2* |
| T04H1.6 | *lrx-1* |
| F40F8.9 | *lsm-1* |
| Y62E10A.12 | *lsm-3* |
| M01B12.4 | *M01B12.4* |
| M01B12.5 | *M01B12.5* |
| M01E11.1 | *M01E11.1* |
| M01E11.3 | *M01E11.3* |
| M01E5.2 | *M01E5.2* |
| M01E5.6 | *M01E5.6* |
| M01F1.8 | *M01F1.8* |
| M02B7.2 | *M02B7.2* |
| M02G9.1 | *M02G9.1* |
| M03B6.4 | *M03B6.4* |
| M03C11.8 | *M03C11.8* |
| M03D4.4 | *M03D4.4* |
| M04C9.4 | *M04C9.4* |
| M05B5.1 | *M05B5.1* |
| M05D6.2 | *M05D6.2* |
| M195.2 | *M195.2* |
| M57.2 | *M57.2* |
| M7.7 | *M7.7* |
| M70.4 | *M70.4* |
| C18D11.2 | *maa-1* |
| Y48C3A.7 | *mac-1* |
| E04F6.3 | *maoc-1* |
| Y110A7A.17 | *mat-1* |
| C40D2.3 | *math-21* |
| T08E11.2 | *math-39* |
| Y62E10A.8 | *mau-8* |
| T04C10.1 | *mbk-1* |
| Y48G1A.6 | *mbtr-1* |
| Y51H1A.6 | *mcd-1* |
| D2030.5 | *mce-1* |
| F20H11.3 | *mdh-1* |
| T09A5.6 | *mdt-10* |
| R144.9 | *mdt-11* |
| Y71H2B.6 | *mdt-19* |
| Y62F5A.1 | *mdt-8* |
| ZK154.3 | *mec-7* |
| F57B10.12 | *mei-2* |
| R05D3.11 | *met-2* |
| F09F7.2 | *mlc-3* |
| C56G7.1 | *mlc-4* |
| ZK430.8 | *mlt-7* |
| T06H11.4 | *moc-1* |
| C04H5.6 | *mog-4* |
| T07H6.2 | *mom-1* |
| F52F12.3 | *mom-4* |
| Y53C12A.4 | *mop-25.2* |
| Y75B8A.26 | *mrp-8* |
| R10E9.1 | *msi-1* |
| K05F1.2 | *msp-142* |
| F36H12.7 | *msp-19* |
| F26G1.7 | *msp-3* |
| C33F10.9 | *msp-40* |
| B0414.7 | *mtk-1* |
| Y110A7A.5 | *mtm-1* |
| H28G03.6 | *mtm-5* |
| ZC97.1 | *mtx-2* |
| K04F10.6 | *mut-2* |
| ZK1098.8 | *mut-7* |
| F43G9.7 | *NA* |
| C43E11.6 | *nab-1* |
| F45G2.1 | *nas-1* |
| T11F9.3 | *nas-20* |
| F58A6.4 | *nas-29* |
| F58B4.1 | *nas-31* |
| C26C6.3 | *nas-36* |
| T23H4.3 | *nas-5* |
| 4R79.1 | *nas-6* |
| C07D10.4 | *nas-7* |
| F02E8.6 | *ncr-1* |
| T26A5.3 | *nduf-2.2* |
| F22D6.4 | *nduf-6* |
| T26E3.2 | *ndx-1* |
| Y38A8.1 | *ndx-3* |
| T05A8.4 | *nep-2* |
| T07C4.9 | *nex-2* |
| C37H5.1 | *nex-4* |
| T08D10.1 | *nfya-1* |
| T19A6.2 | *ngp-1* |
| F54G8.4 | *nhl-1* |
| T06C12.6 | *nhr-102* |
| ZK1025.9 | *nhr-113* |
| C47F8.2 | *nhr-165* |
| C54C8.1 | *nhr-169* |
| E02H1.7 | *nhr-19* |
| K06B4.10 | *nhr-199* |
| K06A1.4 | *nhr-22* |
| ZK1025.6 | *nhr-244* |
| H22D14.1 | *nhr-267* |
| K10C3.6 | *nhr-49* |
| C06C6.5 | *nhr-50* |
| R11G11.2 | *nhr-58* |
| F54D1.4 | *nhr-7* |
| C27C7.3 | *nhr-74* |
| H10E21.3 | *nhr-80* |
| C47F8.8 | *nhr-81* |
| W05B5.3 | *nhr-85* |
| K08A2.5 | *nhr-88* |
| M02H5.6 | *nhr-98* |
| B0495.4 | *nhx-2* |
| ZK822.3 | *nhx-9* |
| F37A8.4 | *nlp-10* |
| ZK1320.10 | *nlp-11* |
| F33A8.2 | *nlp-18* |
| F48C11.3 | *nlp-3* |
| C33A12.2 | *nlp-35* |
| E03D2.2 | *nlp-9* |
| F13H8.4 | *nmgp-1* |
| W01B11.3 | *nol-5* |
| ZK1127.1 | *nos-2* |
| ZK328.5 | *npp-10* |
| Y56A3A.17 | *npp-16* |
| Y77E11A.13 | *npp-20* |
| D1007.7 | *nrd-1* |
| F55B12.5 | *nrf-5* |
| F22D6.3 | *nrs-1* |
| F25G6.6 | *nrs-2* |
| Y23H5B.9 | *nspd-8* |
| K01C8.9 | *nst-1* |
| Y56A3A.1 | *ntl-3* |
| F44A6.1 | *nucb-1* |
| F08F8.1 | *numr-2* |
| T01B11.7 | *oat-1* |
| C34H3.2 | *odd-2* |
| C06G8.2 | *opt-1* |
| T05D4.4 | *osm-7* |
| F48E8.5 | *paa-1* |
| Y106G6H.2 | *pab-1* |
| C17E4.5 | *pabp-2* |
| W03G9.6 | *paf-1* |
| C45B11.1 | *pak-2* |
| H39E23.1 | *par-1* |
| C15H11.7 | *pas-1* |
| C36B1.4 | *pas-4* |
| ZK945.2 | *pas-7* |
| T22A3.5 | *pash-1* |
| F54F2.1 | *pat-2* |
| C29F9.7 | *pat-4* |
| K07C11.1 | *pax-1* |
| F27E5.2 | *pax-3* |
| F39H11.5 | *pbs-7* |
| Y47G6A.6 | *pcaf-1* |
| C10F3.5 | *pcm-1* |
| ZK688.6 | *pcp-5* |
| F54D5.1 | *pcs-1* |
| C32E12.2 | *pde-5* |
| H06O01.1 | *pdi-3* |
| T28H11.4 | *pes-1* |
| B0035.4 | *pfd-4* |
| R151.9 | *pfd-5* |
| T05B4.3 | *phat-4* |
| T05B4.11 | *phat-5* |
| T10B10.6 | *phat-6* |
| Y71F9B.7 | *plk-2* |
| K10F12.3 | *pll-1* |
| C44B7.9 | *pmp-2* |
| F56G4.5 | *png-1* |
| Y76A2B.1 | *pod-1* |
| W10C8.2 | *pop-1* |
| Y49E10.3 | *pph-4.2* |
| VF11C1L.1 | *ppk-3* |
| F44C4.5 | *ppt-1* |
| Y110A7A.18 | *ppw-2* |
| F52C9.8 | *pqe-1* |
| F35B3.5 | *pqn-34* |
| C03A7.4 | *pqn-5* |
| T06E4.11 | *pqn-63* |
| T16G1.1 | *pqn-67* |
| T16G1.10 | *pqn-68* |
| T23F1.6 | *pqn-71* |
| W01B11.5 | *pqn-72* |
| Y39E4B.3 | *pqn-83* |
| Y41C4A.5 | *pqn-84* |
| Y43H11AL.3 | *pqn-85* |
| ZK1067.7 | *pqn-95* |
| D2030.6 | *prg-1* |
| W02D9.1 | *pri-2* |
| C06E8.3 | *prk-1* |
| F45H7.4 | *prk-2* |
| ZK381.5 | *prkl-1* |
| C07E3.2 | *pro-2* |
| F22D6.5 | *prpf-4* |
| C37A5.9 | *pry-1* |
| F54E7.1 | *pst-2* |
| D2089.4 | *ptb-1* |
| F59G1.5 | *ptp-2* |
| F56C11.2 | *ptr-11* |
| Y18D10A.7 | *ptr-17* |
| Y39A1B.2 | *ptr-19* |
| Y53F4B.28 | *ptr-20* |
| Y65B4BR.3 | *ptr-21* |
| ZK270.1 | *ptr-23* |
| C41D7.2 | *ptr-3* |
| Y48G1BL.3 | *puf-10* |
| W06B11.2 | *puf-9* |
| W06H3.2 | *pus-1* |
| T14G10.2 | *pxf-1* |
| C28H8.6 | *pxl-1* |
| D2023.2 | *pyc-1* |
| T05C12.10 | *qua-1* |
| R02D3.1 | *R02D3.1* |
| R02D3.3 | *R02D3.3* |
| R02D3.8 | *R02D3.8* |
| R03D7.1 | *R03D7.1* |
| R03E1.2 | *R03E1.2* |
| R03H10.4 | *R03H10.4* |
| R04B5.6 | *R04B5.6* |
| R05A10.2 | *R05A10.2* |
| R05C11.1 | *R05C11.1* |
| R05D7.4 | *R05D7.4* |
| R05D7.5 | *R05D7.5* |
| R05F9.12 | *R05F9.12* |
| R05G6.10 | *R05G6.10* |
| R05G6.5 | *R05G6.5* |
| R05G9.2 | *R05G9.2* |
| R05H10.2 | *R05H10.2* |
| R05H10.3 | *R05H10.3* |
| R05H5.4 | *R05H5.4* |
| R05H5.5 | *R05H5.5* |
| R06A10.4 | *R06A10.4* |
| R06A4.8 | *R06A4.8* |
| R06B9.1 | *R06B9.1* |
| R06C7.5 | *R06C7.5* |
| R06F6.2 | *R06F6.2* |
| R06F6.7 | *R06F6.7* |
| R07B7.10 | *R07B7.10* |
| R07C12.1 | *R07C12.1* |
| R07C3.11 | *R07C3.11* |
| R07C3.4 | *R07C3.4* |
| R07C3.7 | *R07C3.7* |
| R07E5.11 | *R07E5.11* |
| R07G3.5 | *R07G3.5* |
| R07G3.7 | *R07G3.7* |
| R08C7.2 | *R08C7.2* |
| R08C7.4 | *R08C7.4* |
| R08F11.4 | *R08F11.4* |
| R09B3.3 | *R09B3.3* |
| R09D1.11 | *R09D1.11* |
| R09F10.1 | *R09F10.1* |
| R102.1 | *R102.1* |
| R107.2 | *R107.2* |
| R10E11.3 | *R10E11.3* |
| R10E11.6 | *R10E11.6* |
| R10E4.8 | *R10E4.8* |
| R10H1.3 | *R10H1.3* |
| R10H10.6 | *R10H10.6* |
| R119.5 | *R119.5* |
| R11A5.4 | *R11A5.4* |
| R12C12.1 | *R12C12.1* |
| R13.1 | *R13.1* |
| R13.4 | *R13.4* |
| R13A1.5 | *R13A1.5* |
| R13A1.7 | *R13A1.7* |
| R13A5.9 | *R13A5.9* |
| R13F6.10 | *R13F6.10* |
| R13G10.4 | *R13G10.4* |
| R151.7 | *R151.7* |
| R166.2 | *R166.2* |
| R17.2 | *R17.2* |
| R186.1 | *R186.1* |
| R31.2 | *R31.2* |
| R52.5 | *R52.5* |
| R53.4 | *R53.4* |
| R53.7 | *R53.7* |
| R57.1 | *R57.1* |
| C39F7.4 | *rab-1* |
| F53G12.1 | *rab-11.1* |
| Y62E10A.9 | *rab-19* |
| T01B7.3 | *rab-21* |
| Y45F3A.2 | *rab-30* |
| F26H9.6 | *rab-5* |
| F59B2.7 | *rab-6.1* |
| W03C9.3 | *rab-7* |
| D1037.4 | *rab-8* |
| Y53G8AR.3 | *ral-1* |
| K01G5.4 | *ran-1* |
| T22C1.10 | *rbg-2* |
| F54E7.7 | *rcn-1* |
| W06A7.3 | *ret-1* |
| Y53C10A.4 | *rga-2* |
| F28B4.2 | *rgl-1* |
| F56B6.2 | *rgs-7* |
| F54C8.5 | *rheb-1* |
| W07A12.7 | *rhy-1* |
| F12F6.3 | *rib-1* |
| C14C10.3 | *ril-2* |
| Y62E10A.1 | *rla-2* |
| M142.6 | *rle-1* |
| T11F8.3 | *rme-2* |
| C16C10.7 | *rnf-5* |
| Y47G6A.20 | *rnp-6* |
| Y57A10A.11 | *rol-1* |
| F23B2.13 | *rpb-12* |
| F26F4.11 | *rpb-8* |
| Y71F9AL.13 | *rpl-1* |
| F10B5.1 | *rpl-10* |
| F07D10.1 | *rpl-11.2* |
| K11H12.2 | *rpl-15* |
| Y48G8AL.8 | *rpl-17* |
| Y45F10D.12 | *rpl-18* |
| E04A4.8 | *rpl-20* |
| C14B9.7 | *rpl-21* |
| C53H9.1 | *rpl-27* |
| B0513.3 | *rpl-29* |
| W09C5.6 | *rpl-31* |
| C42C1.14 | *rpl-34* |
| F37C12.4 | *rpl-36* |
| C26F1.9 | *rpl-39* |
| B0041.4 | *rpl-4* |
| C09H10.2 | *rpl-41* |
| R151.3 | *rpl-6* |
| F53G12.10 | *rpl-7* |
| R13A5.8 | *rpl-9* |
| K07D4.3 | *rpn-11* |
| C30C11.2 | *rpn-3* |
| F57B9.10 | *rpn-6* |
| R12E2.3 | *rpn-8* |
| D1007.6 | *rps-10* |
| F54E7.2 | *rps-12* |
| C16A3.9 | *rps-13* |
| F37C12.9 | *rps-14* |
| F36A2.6 | *rps-15* |
| T01C3.6 | *rps-16* |
| C49H3.11 | *rps-2* |
| F37C12.11 | *rps-21* |
| F28D1.7 | *rps-23* |
| B0412.4 | *rps-29* |
| C23G10.3 | *rps-3* |
| T05E11.1 | *rps-5* |
| Y71A12B.1 | *rps-6* |
| ZC434.2 | *rps-7* |
| C52E4.4 | *rpt-1* |
| F29G9.5 | *rpt-2* |
| F23F12.6 | *rpt-3* |
| F23F1.8 | *rpt-4* |
| F56H1.4 | *rpt-5* |
| C18H9.7 | *rpy-1* |
| Y47D3A.16 | *rsks-1* |
| Y111B2A.18 | *rsp-3* |
| D2089.1 | *rsp-7* |
| F28D9.1 | *rsr-1* |
| C27H6.2 | *ruvb-1* |
| F15A2.6 | *sad-1* |
| K11D9.2 | *sca-1* |
| F10G7.4 | *scc-1* |
| F18E2.3 | *scc-3* |
| C39E9.2 | *scl-5* |
| C39E9.4 | *scl-6* |
| Y47D9A.2 | *scpl-3* |
| T22H2.5 | *scrm-1* |
| F46A8.10 | *scrm-6* |
| F42A8.2 | *sdhb-1* |
| F57C7.3 | *sdn-1* |
| F12E12.5 | *sdz-12* |
| F45C12.11 | *sdz-18* |
| F53A2.2 | *sdz-20* |
| K07E8.3 | *sdz-24* |
| ZK673.10 | *sdz-37* |
| C55A6.5 | *sdz-8* |
| C33H5.9 | *sec-10* |
| Y113G7A.3 | *sec-23* |
| F12F6.6 | *sec-24.1* |
| T23G7.4 | *sec-5* |
| F35H12.3 | *sel-12* |
| F35G12.3 | *sel-5* |
| W02D7.7 | *sel-9* |
| C47B2.7 | *selb-1* |
| C02D4.2 | *ser-2* |
| K02F2.6 | *ser-3* |
| Y22D7AR.13 | *ser-4* |
| T22A3.4 | *set-18* |
| ZC8.3 | *set-30* |
| C41G7.4 | *set-32* |
| F44A6.2 | *sex-1* |
| H06I04.2 | *sft-1* |
| C47D12.3 | *sfxn-1.4* |
| W10G6.2 | *sgk-1* |
| F54A5.3 | *shc-1* |
| T27F7.2 | *shc-2* |
| C33B4.3 | *shn-1* |
| Y37E11AR.2 | *siah-1* |
| F39H2.2 | *sig-7* |
| F02E9.4 | *sin-3* |
| F43D9.4 | *sip-1* |
| K08H2.1 | *skr-21* |
| M02A10.3 | *sli-1* |
| ZK370.2 | *sma-2* |
| Y37E11AM.1 | *smgl-2* |
| C41G7.1 | *smn-1* |
| K12C11.2 | *smo-1* |
| Y49F6B.4 | *smu-2* |
| D1014.3 | *snap-1* |
| T10H9.4 | *snb-1* |
| Y46G5A.30 | *snf-5* |
| T08A9.3 | *sng-1* |
| Y38C1BA.2 | *snn-1* |
| C52E4.3 | *snr-4* |
| W06D4.5 | *snx-3* |
| Y71F9B.10 | *sop-3* |
| T28F12.3 | *sos-1* |
| K08A8.2 | *sox-2* |
| F32H2.3 | *spd-2* |
| C06A8.5 | *spdl-1* |
| F47G6.4 | *spe-15* |
| K01A11.4 | *spe-41* |
| Y47G6A.10 | *spg-7* |
| F42G8.11 | *sph-1* |
| T05E11.4 | *spo-11* |
| C28C12.7 | *spp-10* |
| F27C8.4 | *spp-18* |
| ZK616.9 | *spp-7* |
| C09H6.1 | *spr-4* |
| F43H9.2 | *sptl-2* |
| F44F4.13 | *sra-11* |
| F35C5.2 | *sra-14* |
| AH6.8 | *sra-4* |
| AH6.10 | *sra-6* |
| AH6.11 | *sra-7* |
| R10H1.2 | *srab-14* |
| F58A6.6 | *srb-16* |
| C27D6.8 | *srb-3* |
| R05H5.6 | *srb-6* |
| T19E7.5 | *srd-5* |
| F13G3.2 | *srd-53* |
| E04F6.1 | *srd-59* |
| C06G8.4 | *srd-7* |
| C13B7.4 | *srd-71* |
| Y39C12A.6 | *sre-19* |
| F36D1.2 | *sre-22* |
| W05H5.7 | *sre-32* |
| W07G1.6 | *sre-43* |
| W07G1.2 | *sre-44* |
| T04A8.2 | *srg-11* |
| Y51A2D.12 | *srg-34* |
| T12A2.9 | *srg-8* |
| T12A2.10 | *srg-9* |
| R52.7 | *srh-195* |
| W02H5.6 | *srh-271* |
| C10G11.3 | *srh-51* |
| T21B4.5 | *srh-68* |
| T21B4.9 | *srh-70* |
| Y69E1A.6 | *sri-19* |
| F22E5.4 | *sri-47* |
| ZC239.10 | *sri-53* |
| Y45G12C.14 | *srj-19* |
| C05E4.3 | *srp-1* |
| F36D3.3 | *srr-3* |
| K11D12.3 | *srr-4* |
| C13D9.1 | *srr-6* |
| Y62E10A.4 | *srsx-25* |
| Y41E3.12 | *srt-47* |
| T27C10.1 | *srt-61* |
| C33A12.14 | *sru-1* |
| C33A12.13 | *sru-2* |
| C33A12.11 | *sru-3* |
| F36D1.3 | *sru-47* |
| Y105C5B.6 | *srv-15* |
| T13A10.12 | *srv-32* |
| F40H7.8 | *srx-105* |
| T24E12.4 | *srx-111* |
| F49C5.2 | *srx-120* |
| Y43B11AL.2 | *srx-50* |
| F19B10.8 | *srx-98* |
| E03H12.6 | *srz-24* |
| C08F11.9 | *srz-29* |
| H12I19.3 | *srz-30* |
| C39B5.1 | *srz-56* |
| B0205.2 | *srz-85* |
| Y51H4A.17 | *sta-1* |
| F54C9.2 | *stc-1* |
| C07E3.1 | *stip-1* |
| Y9C9A.7 | *str-153* |
| Y45G12C.12 | *str-154* |
| Y9C9A.2 | *str-164* |
| T08B6.6 | *str-166* |
| Y9C9A.4 | *str-169* |
| Y17G9A.7 | *str-174* |
| T10H9.6 | *str-180* |
| F32A7.7 | *str-245* |
| C05E4.13 | *str-262* |
| F28C6.6 | *suf-1* |
| W01B11.2 | *sulp-6* |
| F53C11.8 | *swan-1* |
| ZK1067.6 | *sym-2* |
| C54H2.1 | *sym-3* |
| F36F2.4 | *syn-13* |
| T01A4.2 | *T01A4.2* |
| T01C8.5 | *T01C8.5* |
| T01D1.3 | *T01D1.3* |
| T01D1.4 | *T01D1.4* |
| T01E8.5 | *T01E8.5* |
| T01E8.6 | *T01E8.6* |
| T01G1.2 | *T01G1.2* |
| T01G9.2 | *T01G9.2* |
| T01H8.2 | *T01H8.2* |
| T03D8.2 | *T03D8.2* |
| T03D8.6 | *T03D8.6* |
| T03F1.6 | *T03F1.6* |
| T03G11.4 | *T03G11.4* |
| T04A11.5 | *T04A11.5* |
| T04A8.11 | *T04A8.11* |
| T04A8.5 | *T04A8.5* |
| T04B8.5 | *T04B8.5* |
| T04D1.2 | *T04D1.2* |
| T05A1.3 | *T05A1.3* |
| T05B4.10 | *T05B4.10* |
| T05B4.8 | *T05B4.8* |
| T05C1.3 | *T05C1.3* |
| T05C1.4 | *T05C1.4* |
| T05C12.1 | *T05C12.1* |
| T05E11.2 | *T05E11.2* |
| T05E11.7 | *T05E11.7* |
| T05E7.1 | *T05E7.1* |
| T05E7.4 | *T05E7.4* |
| T05F1.8 | *T05F1.8* |
| T05G5.4 | *T05G5.4* |
| T05H4.4 | *T05H4.4* |
| T05H4.5 | *T05H4.5* |
| T05H4.6 | *T05H4.6* |
| T05H4.7 | *T05H4.7* |
| T06C10.3 | *T06C10.3* |
| T06D8.2 | *T06D8.2* |
| T06D8.5 | *T06D8.5* |
| T06E4.8 | *T06E4.8* |
| T06E4.9 | *T06E4.9* |
| T06G6.3 | *T06G6.3* |
| T07D10.2 | *T07D10.2* |
| T07D3.4 | *T07D3.4* |
| T07D3.9 | *T07D3.9* |
| T07E3.1 | *T07E3.1* |
| T07E3.4 | *T07E3.4* |
| T07F8.4 | *T07F8.4* |
| T08B2.5 | *T08B2.5* |
| T08B2.8 | *T08B2.8* |
| T08E11.8 | *T08E11.8* |
| T08G11.2 | *T08G11.2* |
| T09A5.12 | *T09A5.12* |
| T09A5.7 | *T09A5.7* |
| T09B4.8 | *T09B4.8* |
| T09E11.10 | *T09E11.10* |
| T09E11.5 | *T09E11.5* |
| T09E11.7 | *T09E11.7* |
| T09F3.1 | *T09F3.1* |
| T10E9.9 | *T10E9.9* |
| T10F2.4 | *T10F2.4* |
| T10G3.2 | *T10G3.2* |
| T11B7.2 | *T11B7.2* |
| T11G6.8 | *T11G6.8* |
| T12A2.1 | *T12A2.1* |
| T12A2.6 | *T12A2.6* |
| T12D8.4 | *T12D8.4* |
| T13A10.1 | *T13A10.1* |
| T13F2.2 | *T13F2.2* |
| T13H5.1 | *T13H5.1* |
| T13H5.5 | *T13H5.5* |
| T13H5.6 | *T13H5.6* |
| T14B4.1 | *T14B4.1* |
| T14B4.2 | *T14B4.2* |
| T14D7.1 | *T14D7.1* |
| T15D6.10 | *T15D6.10* |
| T15H9.5 | *T15H9.5* |
| T16G1.5 | *T16G1.5* |
| T16G1.7 | *T16G1.7* |
| T16G12.3 | *T16G12.3* |
| T17A3.10 | *T17A3.10* |
| T17H7.7 | *T17H7.7* |
| T19A5.3 | *T19A5.3* |
| T19B10.9 | *T19B10.9* |
| T19B4.1 | *T19B4.1* |
| T19C3.7 | *T19C3.7* |
| T19D2.3 | *T19D2.3* |
| T19H5.4 | *T19H5.4* |
| T20D3.5 | *T20D3.5* |
| T20F5.4 | *T20F5.4* |
| T20G5.8 | *T20G5.8* |
| T21B10.3 | *T21B10.3* |
| T21B6.2 | *T21B6.2* |
| T21D12.11 | *T21D12.11* |
| T21D12.12 | *T21D12.12* |
| T21G5.4 | *T21G5.4* |
| T22B11.2 | *T22B11.2* |
| T22B11.5 | *T22B11.5* |
| T22C1.8 | *T22C1.8* |
| T22C8.1 | *T22C8.1* |
| T22C8.7 | *T22C8.7* |
| T23B12.3 | *T23B12.3* |
| T23B3.1 | *T23B3.1* |
| T23D8.3 | *T23D8.3* |
| T23F11.1 | *T23F11.1* |
| T23F11.4 | *T23F11.4* |
| T23G11.4 | *T23G11.4* |
| T23G11.6 | *T23G11.6* |
| T23G11.7 | *T23G11.7* |
| T23G4.2 | *T23G4.2* |
| T23G5.6 | *T23G5.6* |
| T23H2.4 | *T23H2.4* |
| T24E12.5 | *T24E12.5* |
| T24F1.2 | *T24F1.2* |
| T25B9.1 | *T25B9.1* |
| T25D3.2 | *T25D3.2* |
| T25D3.4 | *T25D3.4* |
| T26A5.4 | *T26A5.4* |
| T26E3.8 | *T26E3.8* |
| T26G10.1 | *T26G10.1* |
| T27A3.5 | *T27A3.5* |
| T27A3.6 | *T27A3.6* |
| T27A3.7 | *T27A3.7* |
| T27D12.1 | *T27D12.1* |
| T27F2.4 | *T27F2.4* |
| T27F6.1 | *T27F6.1* |
| T27F7.3 | *T27F7.3* |
| T28A11.2 | *T28A11.2* |
| T28D9.1 | *T28D9.1* |
| T28F2.4 | *T28F2.4* |
| T28H10.3 | *T28H10.3* |
| T28H11.7 | *T28H11.7* |
| C31C9.1 | *tag-10* |
| C01B7.4 | *tag-117* |
| F08F1.7 | *tag-123* |
| T11F9.2 | *tag-140* |
| C30H6.2 | *tag-141* |
| M01E11.7 | *tag-163* |
| F44G4.4 | *tag-169* |
| F27D4.5 | *tag-173* |
| D2013.10 | *tag-175* |
| T14G12.3 | *tag-18* |
| W02D3.6 | *tag-194* |
| K02F2.3 | *tag-203* |
| T18H9.7 | *tag-232* |
| F46G11.3 | *tag-257* |
| M04G12.1 | *tag-260* |
| Y54H5A.3 | *tag-262* |
| C38C6.6 | *tag-297* |
| R10E4.2 | *tag-310* |
| T01B11.4 | *tag-316* |
| F17C11.8 | *tag-318* |
| C33H5.10 | *tag-322* |
| C38D4.5 | *tag-325* |
| Y119C1B.8 | *tag-332* |
| C42C1.5 | *tag-335* |
| C01F6.6 | *tag-60* |
| F25D1.1 | *tag-93* |
| F26G5.9 | *tam-1* |
| Y49E10.11 | *tat-1* |
| T24H7.5 | *tat-4* |
| C02F4.2 | *tax-6* |
| C47B2.3 | *tba-2* |
| B0272.1 | *tbb-4* |
| T04H1.9 | *tbb-6* |
| F21H11.3 | *tbx-2* |
| Y47D3A.10 | *tbx-34* |
| ZK829.5 | *tbx-36* |
| Y38C1AA.4 | *tcl-2* |
| Y63D3A.5 | *tfg-1* |
| C08D8.2 | *tmd-2* |
| W03F8.1 | *tni-4* |
| T08B1.2 | *tnt-4* |
| M01A10.2 | *tom-1* |
| B0545.1 | *tpa-1* |
| F42G9.8 | *tpst-2* |
| C23H3.7 | *tre-5* |
| DY3.4 | *trt-1* |
| ZK637.10 | *trxr-2* |
| F55C5.5 | *tsfm-1* |
| C09G12.9 | *tsg-101* |
| B0563.2 | *tsp-11* |
| Y39B6A.6 | *tsp-13* |
| F53B2.2 | *tsp-4* |
| T23D8.2 | *tsp-7* |
| D2013.9 | *ttll-12* |
| Y39E4A.2 | *ttm-1* |
| K03H1.4 | *ttr-2* |
| R13A5.3 | *ttr-32* |
| C04G2.1 | *ttr-39* |
| T14G10.4 | *ttr-54* |
| C33D12.3 | *twk-26* |
| W06D12.2 | *twk-42* |
| F22B7.7 | *twk-7* |
| C35B1.1 | *ubc-1* |
| F49E12.4 | *ubc-24* |
| H06I04.4 | *ubl-1* |
| F25B5.4 | *ubq-1* |
| Y94H6A.9 | *ubxn-2* |
| F48A11.5 | *ubxn-3* |
| F26H9.7 | *uev-3* |
| C10H11.5 | *ugt-27* |
| C10H11.4 | *ugt-28* |
| C07A9.6 | *ugt-60* |
| C07G3.9 | *ugt-64* |
| ZK370.7 | *ugtp-1* |
| C32E8.10 | *unc-11* |
| F11C3.2 | *unc-122* |
| H14N18.1 | *unc-23* |
| ZK897.1 | *unc-31* |
| F26C11.2 | *unc-4* |
| B0350.2 | *unc-44* |
| Y60A3A.1 | *unc-51* |
| C38C3.5 | *unc-60* |
| Y50E8A.4 | *unc-61* |
| F55C7.7 | *unc-73* |
| C01G10.11 | *unc-76* |
| C09D1.1 | *unc-89* |
| Y105E8A.6 | *unc-95* |
| Y56A3A.29 | *ung-1* |
| F56B6.4 | *uvt-5* |
| T22D2.1 | *vab-19* |
| K12F2.2 | *vab-8* |
| VC5.2 | *VC5.2* |
| T17A3.1 | *ver-1* |
| F44F1.7 | *vet-6* |
| Y38F2AL.3 | *vha-11* |
| F20B6.2 | *vha-12* |
| C30F8.2 | *vha-16* |
| Y38F2AL.4 | *vha-3* |
| T01H3.1 | *vha-4* |
| VW02B12L.1 | *vha-6* |
| C17H12.14 | *vha-8* |
| ZK970.4 | *vha-9* |
| F56D12.5 | *vig-1* |
| C10H11.1 | *viln-1* |
| B0025.1 | *vps-34* |
| VW02B12L.2 | *VW02B12L.2* |
| W01A11.1 | *W01A11.1* |
| W01A11.2 | *W01A11.2* |
| W01A8.4 | *W01A8.4* |
| W01B6.2 | *W01B6.2* |
| W01C8.5 | *W01C8.5* |
| W01C9.1 | *W01C9.1* |
| W01D2.1 | *W01D2.1* |
| W01D2.5 | *W01D2.5* |
| W02A11.3 | *W02A11.3* |
| W02B12.10 | *W02B12.10* |
| W02B12.11 | *W02B12.11* |
| W02B12.9 | *W02B12.9* |
| W02B8.3 | *W02B8.3* |
| W02D3.4 | *W02D3.4* |
| W02F12.2 | *W02F12.2* |
| W02F12.5 | *W02F12.5* |
| W02G9.4 | *W02G9.4* |
| W03A5.4 | *W03A5.4* |
| W03D8.2 | *W03D8.2* |
| W03F8.3 | *W03F8.3* |
| W03F8.4 | *W03F8.4* |
| W03F9.4 | *W03F9.4* |
| W03G1.2 | *W03G1.2* |
| W04A8.1 | *W04A8.1* |
| W04A8.5 | *W04A8.5* |
| W04C9.3 | *W04C9.3* |
| W04C9.5 | *W04C9.5* |
| W04G5.1 | *W04G5.1* |
| W04H10.1 | *W04H10.1* |
| W05B10.2 | *W05B10.2* |
| W05F2.4 | *W05F2.4* |
| W05G11.6 | *W05G11.6* |
| W05H12.2 | *W05H12.2* |
| W06E11.4 | *W06E11.4* |
| W06H8.2 | *W06H8.2* |
| W07A12.4 | *W07A12.4* |
| W07A12.6 | *W07A12.6* |
| W07B8.3 | *W07B8.3* |
| W07G1.1 | *W07G1.1* |
| W08E12.3 | *W08E12.3* |
| W08E12.5 | *W08E12.5* |
| W09C3.7 | *W09C3.7* |
| W09C5.7 | *W09C5.7* |
| W09C5.8 | *W09C5.8* |
| W09G10.3 | *W09G10.3* |
| D2030.9 | *wdr-23* |
| R144.4 | *wip-1* |
| F18C5.2 | *wrn-1* |
| ZK1290.12 | *wrt-1* |
| T20F10.1 | *wts-1* |
| R06C1.3 | *wve-1* |
| Y65B4BR.4 | *wwp-1* |
| F40F9.1 | *xbx-6* |
| K07G5.2 | *xpa-1* |
| Y48B6A.3 | *xrn-2* |
| Y102A5C.2 | *Y102A5C.2* |
| Y105C5A.1 | *Y105C5A.1* |
| Y105C5B.7 | *Y105C5B.7* |
| Y106G6A.1 | *Y106G6A.1* |
| Y106G6D.1 | *Y106G6D.1* |
| Y106G6D.2 | *Y106G6D.2* |
| Y106G6D.3 | *Y106G6D.3* |
| Y106G6D.7 | *Y106G6D.7* |
| Y106G6E.1 | *Y106G6E.1* |
| Y106G6H.15 | *Y106G6H.15* |
| Y106G6H.4 | *Y106G6H.4* |
| Y106G6H.5 | *Y106G6H.5* |
| Y106G6H.6 | *Y106G6H.6* |
| Y110A2AL.1 | *Y110A2AL.1* |
| Y110A2AL.12 | *Y110A2AL.12* |
| Y110A2AL.7 | *Y110A2AL.7* |
| Y110A7A.11 | *Y110A7A.11* |
| Y110A7A.4 | *Y110A7A.4* |
| Y110A7A.6 | *Y110A7A.6* |
| Y110A7A.8 | *Y110A7A.8* |
| Y113G7B.11 | *Y113G7B.11* |
| Y116A8C.11 | *Y116A8C.11* |
| Y116A8C.23 | *Y116A8C.23* |
| Y116A8C.29 | *Y116A8C.29* |
| Y116A8C.33 | *Y116A8C.33* |
| Y11D7A.15 | *Y11D7A.15* |
| Y11D7A.6 | *Y11D7A.6* |
| Y11D7A.9 | *Y11D7A.9* |
| Y14H12A.1 | *Y14H12A.1* |
| Y17G9B.4 | *Y17G9B.4* |
| Y18D10A.23 | *Y18D10A.23* |
| Y19D10B.4 | *Y19D10B.4* |
| Y23H5B.4 | *Y23H5B.4* |
| Y23H5B.5 | *Y23H5B.5* |
| Y23H5B.6 | *Y23H5B.6* |
| Y24F12A.2 | *Y24F12A.2* |
| Y25C1A.13 | *Y25C1A.13* |
| Y25C1A.2 | *Y25C1A.2* |
| Y25C1A.7 | *Y25C1A.7* |
| Y26D4A.2 | *Y26D4A.2* |
| Y27F2A.5 | *Y27F2A.5* |
| Y32H12A.7 | *Y32H12A.7* |
| Y34D9A.1 | *Y34D9A.1* |
| Y34D9A.2 | *Y34D9A.2* |
| Y34D9A.3 | *Y34D9A.3* |
| Y37D8A.18 | *Y37D8A.18* |
| Y37D8A.2 | *Y37D8A.2* |
| Y37D8A.21 | *Y37D8A.21* |
| Y37D8A.22 | *Y37D8A.22* |
| Y37E11AM.3 | *Y37E11AM.3* |
| Y37E11B.6 | *Y37E11B.6* |
| Y37H9A.1 | *Y37H9A.1* |
| Y37H9A.3 | *Y37H9A.3* |
| Y38B5A.1 | *Y38B5A.1* |
| Y38C1AA.7 | *Y38C1AA.7* |
| Y38C1AB.1 | *Y38C1AB.1* |
| Y38C1AB.6 | *Y38C1AB.6* |
| Y38C1BA.1 | *Y38C1BA.1* |
| Y38E10A.17 | *Y38E10A.17* |
| Y38E10A.24 | *Y38E10A.24* |
| Y38H6A.3 | *Y38H6A.3* |
| Y38H6C.20 | *Y38H6C.20* |
| Y39A1A.12 | *Y39A1A.12* |
| Y39A1A.18 | *Y39A1A.18* |
| Y39A1A.8 | *Y39A1A.8* |
| Y39B6A.42 | *Y39B6A.42* |
| Y39E4B.10 | *Y39E4B.10* |
| Y39F10A.3 | *Y39F10A.3* |
| Y39G8B.5 | *Y39G8B.5* |
| Y39G8B.7 | *Y39G8B.7* |
| Y39H10A.2 | *Y39H10A.2* |
| Y40D12A.1 | *Y40D12A.1* |
| Y40H7A.4 | *Y40H7A.4* |
| Y41C4A.1 | *Y41C4A.1* |
| Y41E3.6 | *Y41E3.6* |
| Y43D4A.6 | *Y43D4A.6* |
| Y43F8B.1 | *Y43F8B.1* |
| Y43F8B.9 | *Y43F8B.9* |
| Y43F8C.4 | *Y43F8C.4* |
| Y43H11AL.2 | *Y43H11AL.2* |
| Y44A6D.2 | *Y44A6D.2* |
| Y44A6D.3 | *Y44A6D.3* |
| Y45F10D.4 | *Y45F10D.4* |
| Y45F3A.1 | *Y45F3A.1* |
| Y45G12C.1 | *Y45G12C.1* |
| Y46B2A.1 | *Y46B2A.1* |
| Y46G5A.12 | *Y46G5A.12* |
| Y46G5A.13 | *Y46G5A.13* |
| Y46G5A.20 | *Y46G5A.20* |
| Y46G5A.22 | *Y46G5A.22* |
| Y46H3A.4 | *Y46H3A.4* |
| Y47D3B.1 | *Y47D3B.1* |
| Y47D7A.12 | *Y47D7A.12* |
| Y47D9A.1 | *Y47D9A.1* |
| Y47D9A.4 | *Y47D9A.4* |
| Y47G6A.12 | *Y47G6A.12* |
| Y47G6A.19 | *Y47G6A.19* |
| Y47G6A.22 | *Y47G6A.22* |
| Y47H10A.4 | *Y47H10A.4* |
| Y47H9C.1 | *Y47H9C.1* |
| Y47H9C.2 | *Y47H9C.2* |
| Y47H9C.7 | *Y47H9C.7* |
| Y47H9C.8 | *Y47H9C.8* |
| Y48A6C.4 | *Y48A6C.4* |
| Y48B6A.6 | *Y48B6A.6* |
| Y48C3A.10 | *Y48C3A.10* |
| Y48C3A.12 | *Y48C3A.12* |
| Y48C3A.16 | *Y48C3A.16* |
| Y48C3A.4 | *Y48C3A.4* |
| Y48E1B.3 | *Y48E1B.3* |
| Y48E1B.5 | *Y48E1B.5* |
| Y48E1C.1 | *Y48E1C.1* |
| Y48G10A.3 | *Y48G10A.3* |
| Y48G1A.4 | *Y48G1A.4* |
| Y48G1C.1 | *Y48G1C.1* |
| Y48G1C.8 | *Y48G1C.8* |
| Y48G9A.9 | *Y48G9A.9* |
| Y49E10.16 | *Y49E10.16* |
| Y4C6B.1 | *Y4C6B.1* |
| Y4C6B.2 | *Y4C6B.2* |
| Y4C6B.3 | *Y4C6B.3* |
| Y4C6B.6 | *Y4C6B.6* |
| Y50C1A.1 | *Y50C1A.1* |
| Y50D4B.6 | *Y50D4B.6* |
| Y51H4A.14 | *Y51H4A.14* |
| Y51H7BR.4 | *Y51H7BR.4* |
| Y51H7C.13 | *Y51H7C.13* |
| Y52B11A.10 | *Y52B11A.10* |
| Y52B11A.3 | *Y52B11A.3* |
| Y52B11A.9 | *Y52B11A.9* |
| Y53C12A.6 | *Y53C12A.6* |
| Y53C12B.2 | *Y53C12B.2* |
| Y53C12B.6 | *Y53C12B.6* |
| Y53F4B.1 | *Y53F4B.1* |
| Y53F4B.11 | *Y53F4B.11* |
| Y53F4B.12 | *Y53F4B.12* |
| Y53F4B.13 | *Y53F4B.13* |
| Y53F4B.18 | *Y53F4B.18* |
| Y53F4B.4 | *Y53F4B.4* |
| Y53F4B.8 | *Y53F4B.8* |
| Y53G8AL.2 | *Y53G8AL.2* |
| Y53G8AR.1 | *Y53G8AR.1* |
| Y53H1B.1 | *Y53H1B.1* |
| Y54E10A.6 | *Y54E10A.6* |
| Y54E10BR.2 | *Y54E10BR.2* |
| Y54E10BR.8 | *Y54E10BR.8* |
| Y54E2A.10 | *Y54E2A.10* |
| Y54E2A.2 | *Y54E2A.2* |
| Y54E5A.5 | *Y54E5A.5* |
| Y54E5A.7 | *Y54E5A.7* |
| Y54G11A.7 | *Y54G11A.7* |
| Y54G2A.15 | *Y54G2A.15* |
| Y54G2A.26 | *Y54G2A.26* |
| Y54H5A.4 | *Y54H5A.4* |
| Y55F3AM.10 | *Y55F3AM.10* |
| Y55F3AM.14 | *Y55F3AM.14* |
| Y55F3AM.3 | *Y55F3AM.3* |
| Y55F3BL.2 | *Y55F3BL.2* |
| Y55F3BR.6 | *Y55F3BR.6* |
| Y56A3A.12 | *Y56A3A.12* |
| Y56A3A.19 | *Y56A3A.19* |
| Y56A3A.7 | *Y56A3A.7* |
| Y57A10A.13 | *Y57A10A.13* |
| Y57A10A.15 | *Y57A10A.15* |
| Y57A10A.24 | *Y57A10A.24* |
| Y57A10A.27 | *Y57A10A.27* |
| Y57A10A.4 | *Y57A10A.4* |
| Y57G11B.1 | *Y57G11B.1* |
| Y57G11C.18 | *Y57G11C.18* |
| Y57G7A.10 | *Y57G7A.10* |
| Y58A7A.3 | *Y58A7A.3* |
| Y59A8B.10 | *Y59A8B.10* |
| Y59H11AM.2 | *Y59H11AM.2* |
| Y59H11AM.4 | *Y59H11AM.4* |
| Y61A9LA.1 | *Y61A9LA.1* |
| Y61A9LA.3 | *Y61A9LA.3* |
| Y62E10A.13 | *Y62E10A.13* |
| Y64G10A.2 | *Y64G10A.2* |
| Y65B4A.6 | *Y65B4A.6* |
| Y65B4A.7 | *Y65B4A.7* |
| Y65B4BL.3 | *Y65B4BL.3* |
| Y65B4BR.1 | *Y65B4BR.1* |
| Y65B4BR.8 | *Y65B4BR.8* |
| Y66A7A.5 | *Y66A7A.5* |
| Y66H1A.4 | *Y66H1A.4* |
| Y67A10A.8 | *Y67A10A.8* |
| Y67D8B.2 | *Y67D8B.2* |
| Y69E1A.4 | *Y69E1A.4* |
| Y6B3B.5 | *Y6B3B.5* |
| Y6D1A.1 | *Y6D1A.1* |
| Y71F9AL.10 | *Y71F9AL.10* |
| Y71F9AL.17 | *Y71F9AL.17* |
| Y71F9AL.6 | *Y71F9AL.6* |
| Y71F9AL.8 | *Y71F9AL.8* |
| Y71F9B.8 | *Y71F9B.8* |
| Y71F9B.9 | *Y71F9B.9* |
| Y71H10A.1 | *Y71H10A.1* |
| Y71H2B.2 | *Y71H2B.2* |
| Y73F4A.1 | *Y73F4A.1* |
| Y73F8A.10 | *Y73F8A.10* |
| Y73F8A.12 | *Y73F8A.12* |
| Y73F8A.22 | *Y73F8A.22* |
| Y73F8A.25 | *Y73F8A.25* |
| Y73F8A.27 | *Y73F8A.27* |
| Y73F8A.5 | *Y73F8A.5* |
| Y74C9A.4 | *Y74C9A.4* |
| Y75B8A.14 | *Y75B8A.14* |
| Y75B8A.19 | *Y75B8A.19* |
| Y75B8A.29 | *Y75B8A.29* |
| Y75B8A.4 | *Y75B8A.4* |
| Y75B8A.8 | *Y75B8A.8* |
| Y77E11A.5 | *Y77E11A.5* |
| Y81G3A.3 | *Y81G3A.3* |
| Y87G2A.2 | *Y87G2A.2* |
| Y8A9A.3 | *Y8A9A.3* |
| Y95B8A.2 | *Y95B8A.2* |
| Y95D11A.1 | *Y95D11A.1* |
| Y97E10AR.2 | *Y97E10AR.2* |
| Y9C2UA.1 | *Y9C2UA.1* |
| Y9C2UA.2 | *Y9C2UA.2* |
| Y9C9A.1 | *Y9C9A.1* |
| K08F11.4 | *yrs-1* |
| ZC123.4 | *ZC123.4* |
| ZC155.5 | *ZC155.5* |
| ZC204.14 | *ZC204.14* |
| ZC239.15 | *ZC239.15* |
| ZC239.4 | *ZC239.4* |
| ZC334.4 | *ZC334.4* |
| ZC434.9 | *ZC434.9* |
| ZC455.1 | *ZC455.1* |
| ZC487.1 | *ZC487.1* |
| ZC581.9 | *ZC581.9* |
| F59B2.6 | *zif-1* |
| C34D1.5 | *zip-5* |
| ZK1053.2 | *ZK1053.2* |
| ZK1053.3 | *ZK1053.3* |
| ZK1098.2 | *ZK1098.2* |
| ZK1098.5 | *ZK1098.5* |
| ZK112.5 | *ZK112.5* |
| ZK1127.12 | *ZK1127.12* |
| ZK1128.2 | *ZK1128.2* |
| ZK1128.7 | *ZK1128.7* |
| ZK121.2 | *ZK121.2* |
| ZK1225.1 | *ZK1225.1* |
| ZK1225.2 | *ZK1225.2* |
| ZK1225.4 | *ZK1225.4* |
| ZK1225.5 | *ZK1225.5* |
| ZK1248.1 | *ZK1248.1* |
| ZK1290.7 | *ZK1290.7* |
| ZK1307.1 | *ZK1307.1* |
| ZK1320.8 | *ZK1320.8* |
| ZK1320.9 | *ZK1320.9* |
| ZK1321.4 | *ZK1321.4* |
| ZK177.8 | *ZK177.8* |
| ZK180.4 | *ZK180.4* |
| ZK218.5 | *ZK218.5* |
| ZK228.3 | *ZK228.3* |
| ZK265.7 | *ZK265.7* |
| ZK353.3 | *ZK353.3* |
| ZK354.3 | *ZK354.3* |
| ZK430.7 | *ZK430.7* |
| ZK484.1 | *ZK484.1* |
| ZK484.7 | *ZK484.7* |
| ZK507.6 | *ZK507.6* |
| ZK546.2 | *ZK546.2* |
| ZK596.3 | *ZK596.3* |
| ZK6.7 | *ZK6.7* |
| ZK616.2 | *ZK616.2* |
| ZK616.6 | *ZK616.6* |
| ZK632.3 | *ZK632.3* |
| ZK632.4 | *ZK632.4* |
| ZK643.1 | *ZK643.1* |
| ZK666.4 | *ZK666.4* |
| ZK669.4 | *ZK669.4* |
| ZK673.5 | *ZK673.5* |
| ZK682.5 | *ZK682.5* |
| ZK686.3 | *ZK686.3* |
| ZK792.4 | *ZK792.4* |
| ZK84.1 | *ZK84.1* |
| ZK849.1 | *ZK849.1* |
| ZK856.8 | *ZK856.8* |
| ZK858.5 | *ZK858.5* |
| ZK930.1 | *ZK930.1* |
| ZK930.4 | *ZK930.4* |
| ZK938.1 | *ZK938.1* |
| ZK970.1 | *ZK970.1* |
| ZK970.7 | *ZK970.7* |
| ZK973.11 | *ZK973.11* |
| W06H12.1 | *ztf-6* |
| ZK546.1 | *zyg-12* |
| F42G4.3 | *zyx-1* |
