# Supplementary material for: Large-scale functional RNAi screen in C. elegans identifies genes that regulate the dysfunction of mutant polyglutamine neurons
Source: BMC Genomics. 2012 Mar 13;13:91. doi: 10.1186/1471-2164-13-91 (PMC3331833; doi:10.1186/1471-2164-13-91)
Supplement: Additional file 3 — Table S2. List of the 3823 genes that either showed no effect or modified 128Q-neuron dysfunction when knocked-down by RNAi in the primary screen. [file 1471-2164-13-91-S3.DOC]

**Supplementary Table 2.** List of the 3823 genes that either showed no effect or modified 128Q-neuron dysfunction when knocked-down by RNAi in the primary screen.

Genes are ranked by decreasing S-score values. S means suppression of 128Q-neuron dysfunction when knocked-down by RNAi, whereas E means enhancement of 128Q-neuron dysfunction. NE means no effect on 128Q-neuron dysfunction. The maximally-achievable score is 4.55 (100% response to touch) and the smallest score is -1 (complete loss of touch response).

| **Gene ID** | **Gene name** | **S score** | **Effect** |
| --- | --- | --- | --- |
| Y74C9A.2 | *nlp-40* | 3.359 | S |
| C04E7.2 | *sor-3* | 2.208 | S |
| F26E4.8 | *tba-1* | 2.012 | S |
| Y79H2A.1 | *brp-1* | 1.999 | S |
| T07A9.5 | *eri-1* | 1.957 | S |
| K07C5.4 | *K07C5.4* | 1.948 | S |
| C32D5.9 | *lgg-1* | 1.937 | S |
| C27B7.8 | *rap-1* | 1.933 | S |
| Y66A7A.6 | *gly-8* | 1.888 | S |
| C17E4.3 | *C17E4.3* | 1.876 | S |
| ZK856.12 | *ZK856.12* | 1.821 | S |
| F55F8.2 | *F55F8.2* | 1.818 | S |
| T21C12.1 | *unc-49* | 1.789 | S |
| F40F12.4 | *F40F12.4* | 1.706 | S |
| Y111B2A.22 | *ssl-1* | 1.701 | S |
| F41F3.3 | *F41F3.3* | 1.683 | S |
| K08F8.4 | *pah-1* | 1.665 | S |
| C13G5.2 | *C13G5.2* | 1.627 | S |
| VW02B12L.3 | *ebp-2* | 1.611 | S |
| K08E7.1 | *K08E7.1* | 1.595 | S |
| Y54G2A.16 | *Y54G2A.16* | 1.563 | S |
| Y39B6A.2 | *pph-5* | 1.548 | S |
| F23H12.2 | *F23H12.2* | 1.544 | S |
| F56H11.1 | *fbl-1* | 1.515 | S |
| Y102A5C.1 | *fbxa-206* | 1.508 | S |
| Y102E9.3 | *Y102E9.3* | 1.5 | S |
| C37A2.7 | *C37A2.7* | 1.481 | S |
| Y40B10A.6 | *Y40B10A.6* | 1.48 | S |
| C31H5.4 | *C31H5.4* | 1.472 | S |
| M01F1.1 | *gly-14* | 1.451 | S |
| C34E10.7 | *cnd-1* | 1.449 | S |
| K10F12.4 | *gsto-3* | 1.425 | S |
| W02D3.1 | *W02D3.1* | 1.411 | S |
| Y40B10A.2 | *Y40B10A.2* | 1.382 | S |
| F10E9.2 | *F10E9.2* | 1.373 | S |
| F42H10.7 | *F42H10.7* | 1.363 | S |
| C28A5.1 | *C28A5.1* | 1.362 | S |
| C50D2.2 | *C50D2.2* | 1.35 | S |
| Y105C5A.10 | *Y105C5A.10* | 1.347 | S |
| Y57A10A.28 | *Y57A10A.28* | 1.345 | S |
| T28D9.10 | *snr-3* | 1.345 | S |
| T19B4.3 | *T19B4.3* | 1.336 | S |
| C17H12.8 | *C17H12.8* | 1.321 | S |
| Y38E10A.3 | *Y38E10A.3* | 1.32 | S |
| B0035.6 | *B0035.6* | 1.314 | S |
| K07H8.2 | *K07H8.2* | 1.314 | S |
| H14E04.2 | *H14E04.2* | 1.309 | S |
| F39C12.2 | *add-1* | 1.305 | S |
| ZC21.6 | *ZC21.6* | 1.303 | S |
| T22C8.5 | *sptf-2* | 1.301 | S |
| T12B5.2 | *fbxa-54* | 1.297 | S |
| F35G12.5 | *F35G12.5* | 1.297 | S |
| F45D3.5 | *sel-1* | 1.283 | S |
| R02F2.8 | *R02F2.8* | 1.282 | S |
| F53A3.3 | *rps-22* | 1.275 | S |
| C29F9.5 | *C29F9.5* | 1.256 | S |
| H21P03.1 | *mbf-1* | 1.252 | S |
| B0252.6 | *B0252.6* | 1.25 | S |
| F54C8.3 | *emb-30* | 1.242 | S |
| R02F2.1 | *R02F2.1* | 1.24 | S |
| Y57G11C.23 | *Y57G11C.23* | 1.233 | S |
| ZK430.3 | *sod-5* | 1.232 | S |
| F29G9.1 | *F29G9.1* | 1.223 | S |
| Y51B9A.6 | *Y51B9A.6* | 1.22 | S |
| W07E6.2 | *W07E6.2* | 1.217 | S |
| F22B7.9 | *F22B7.9* | 1.216 | S |
| T13F2.7 | *sna-2* | 1.213 | S |
| Y39E4B.4 | *tsp-3* | 1.206 | S |
| ZK1290.6 | *rnh-1.1* | 1.203 | S |
| C34B2.4 | *C34B2.4* | 1.199 | S |
| Y69F12A.1 | *Y69F12A.1* | 1.195 | S |
| F47C10.2 | *btb-21* | 1.195 | S |
| F10E9.7 | *F10E9.7* | 1.195 | S |
| T02C12.1 | *hum-5* | 1.193 | S |
| F52C9.5 | *F52C9.5* | 1.189 | S |
| F52B5.6 | *rpl-25.2* | 1.189 | S |
| T07D1.2 | *T07D1.2* | 1.183 | S |
| B0218.3 | *pmk-1* | 1.177 | S |
| R09B3.2 | *R09B3.2* | 1.17 | S |
| H35N09.1 | *H35N09.1* | 1.169 | S |
| W02D9.2 | *W02D9.2* | 1.162 | S |
| ZK829.6 | *tgt-1* | 1.159 | S |
| F27B3.6 | *F27B3.6* | 1.135 | S |
| B0281.5 | *B0281.5* | 1.131 | S |
| C16C10.10 | *glod-4* | 1.127 | S |
| ZK550.6 | *ZK550.6* | 1.126 | S |
| T13F2.6 | *T13F2.6* | 1.116 | S |
| F20H11.5 | *F20H11.5* | 1.116 | S |
| C53D6.2 | *unc-129* | 1.115 | S |
| Y59A8A.3 | *Y59A8A.3* | 1.112 | S |
| F07H5.8 | *F07H5.8* | 1.108 | S |
| Y47D3A.27 | *teg-1* | 1.102 | S |
| F36H12.13 | *F36H12.13* | 1.098 | S |
| ZK637.8 | *unc-32* | 1.092 | S |
| C08E3.4 | *fbxa-161* | 1.086 | S |
| C10C5.5 | *C10C5.5* | 1.079 | S |
| K08C7.4 | *K08C7.4* | 1.075 | S |
| Y39A3CL.3 | *Y39A3CL.3* | 1.072 | S |
| Y48B6A.8 | *ace-3* | 1.069 | S |
| F54D10.7 | *F54D10.7* | 1.064 | S |
| K01G5.5 | *K01G5.5* | 1.064 | S |
| T26C5.1 | *gst-13* | 1.063 | S |
| K02B9.4 | *elt-3* | 1.054 | S |
| W06E11.1 | *W06E11.1* | 1.054 | S |
| C36B1.1 | *cle-1* | 1.047 | S |
| K08D10.3 | *rnp-3* | 1.046 | S |
| F28C12.5 | *sra-21* | 1.046 | S |
| B0361.7 | *B0361.7* | 1.04 | S |
| R52.8 | *math-36* | 1.039 | S |
| W06A11.1 | *W06A11.1* | 1.036 | S |
| T12F5.1 | *T12F5.1* | 1.031 | S |
| F58A4.8 | *tbg-1* | 1.029 | S |
| Y51H7C.2 | *scl-17* | 1.029 | S |
| Y51A2D.11 | *ttr-26* | 1.026 | S |
| C10H11.3 | *ugt-25* | 1.024 | S |
| C03C11.2 | *fog-3* | 1.02 | S |
| F58H7.5 | *F58H7.5* | 1.01 | S |
| ZK1127.3 | *ZK1127.3* | 1.008 | S |
| T28F4.3 | *T28F4.3* | 1.007 | S |
| ZK84.2 | *ZK84.2* | 1.006 | S |
| F25B4.4 | *F25B4.4* | 0.998 | S |
| F45G2.9 | *F45G2.9* | 0.997 | S |
| Y18D10A.5 | *gsk-3* | 0.993 | S |
| W02D9.5 | *W02D9.5* | 0.99 | S |
| Y39E4B.9 | *bre-2* | 0.986 | S |
| F54D5.9 | *F54D5.9* | 0.983 | S |
| F27C1.4 | *F27C1.4* | 0.977 | S |
| Y57G11B.5 | *Y57G11B.5* | 0.975 | S |
| F38A6.1 | *pha-4* | 0.974 | S |
| C56A3.5 | *C56A3.5* | 0.971 | S |
| ZK1098.4 | *ZK1098.4* | 0.964 | S |
| C03B8.3 | *C03B8.3* | 0.959 | S |
| F37E3.3 | *F37E3.3* | 0.954 | S |
| Y39A1A.3 | *Y39A1A.3* | 0.952 | S |
| ZK512.8 | *ZK512.8* | 0.952 | S |
| Y41G9A.4 | *Y41G9A.4* | 0.947 | S |
| F10E9.4 | *F10E9.4* | 0.945 | S |
| C08F8.5 | *fbxb-9* | 0.943 | S |
| F57C9.4 | *F57C9.4* | 0.942 | S |
| Y57G11C.33 | *Y57G11C.33* | 0.939 | S |
| C56C10.9 | *C56C10.9* | 0.935 | S |
| K03F8.2 | *acr-5* | 0.933 | S |
| M110.3 | *M110.3* | 0.933 | S |
| K02D10.5 | *K02D10.5* | 0.93 | S |
| ZK1290.11 | *ZK1290.11* | 0.923 | S |
| C39B5.7 | *fbxa-12* | 0.919 | S |
| C25A1.11 | *aha-1* | 0.919 | S |
| T04A11.4 | *T04A11.4* | 0.914 | S |
| K02E11.5 | *K02E11.5* | 0.914 | S |
| Y40B1B.8 | *Y40B1B.8* | 0.91 | S |
| F58E1.6 | *nhx-6* | 0.902 | S |
| K03D10.1 | *kal-1* | 0.901 | S |
| C34F11.6 | *msp-49* | 0.9 | S |
| T23B12.6 | *T23B12.6* | 0.9 | S |
| F19H6.1 | *nekl-3* | 0.9 | S |
| R12E2.14 | *R12E2.14* | 0.898 | S |
| F53A2.9 | *F53A2.9* | 0.895 | S |
| ZK652.2 | *tomm-7* | 0.893 | S |
| C25A8.1 | *C25A8.1* | 0.89 | S |
| C54D10.3 | *C54D10.3* | 0.89 | S |
| T19C3.8 | *fem-2* | 0.888 | S |
| B0304.1 | *B0304.1* | 0.888 | S |
| C25A11.4 | *ajm-1* | 0.88 | S |
| R03E9.1 | *mdl-1* | 0.88 | S |
| K01G5.2 | *hpl-2* | 0.876 | S |
| F14F7.2 | *cyp-13A11* | 0.873 | S |
| Y116F11B.12 | *gly-4* | 0.873 | S |
| T05H10.8 | *T05H10.8* | 0.872 | S |
| F43C9.4 | *mig-13* | 0.871 | S |
| ZC395.10 | *ZC395.10* | 0.868 | S |
| F22D3.1 | *ceh-38* | 0.867 | S |
| C01G5.8 | *C01G5.8* | 0.867 | S |
| T03F6.1 | *qdpr-1* | 0.857 | S |
| F42G10.1 | *F42G10.1* | 0.856 | S |
| C48B4.11 | *C48B4.11* | 0.854 | S |
| Y37D8A.19 | *Y37D8A.19* | 0.851 | S |
| C56C10.8 | *icd-1* | 0.842 | S |
| F38H4.8 | *ech-2* | 0.841 | S |
| ZK39.3 | *clec-94* | 0.84 | S |
| F25B5.5 | *F25B5.5* | 0.837 | S |
| Y39E4B.12 | *gly-5* | 0.836 | S |
| T19B10.11 | *mxl-1* | 0.832 | S |
| ZC15.8 | *pqn-94* | 0.831 | S |
| T10H9.3 | *T10H9.3* | 0.83 | S |
| F09G8.6 | *col-91* | 0.827 | S |
| C34F11.8 | *C34F11.8* | 0.823 | S |
| W03D2.6 | *W03D2.6* | 0.817 | S |
| F17E5.1 | *lin-2* | 0.815 | S |
| F53B7.3 | *F53B7.3* | 0.812 | S |
| B0412.2 | *daf-7* | 0.806 | S |
| F13G3.4 | *dylt-1* | 0.804 | S |
| B0393.2 | *rbg-3* | 0.8 | S |
| F08B6.2 | *gpc-2* | 0.8 | S |
| F53C3.5 | *F53C3.5* | 0.797 | S |
| C45B11.3 | *dhs-18* | 0.795 | S |
| Y57E12AL.5 | *mdt-6* | 0.791 | S |
| C24H11.1 | *C24H11.1* | 0.787 | S |
| Y17G7B.2 | *ash-2* | 0.787 | S |
| F36A4.5 | *F36A4.5* | 0.784 | S |
| B0547.1 | *csn-5* | 0.783 | S |
| C35D10.10 | *C35D10.10* | 0.781 | S |
| C37A2.4 | *cye-1* | 0.78 | S |
| F53A2.5 | *dro-1* | 0.772 | S |
| C56C10.10 | *C56C10.10* | 0.77 | S |
| Y40B10A.1 | *lbp-9* | 0.767 | S |
| F56G4.3 | *pes-2.2* | 0.762 | S |
| T20B5.2 | *T20B5.2* | 0.761 | S |
| VT23B5.2 | *VT23B5.2* | 0.758 | S |
| Y34D9A.8 | *Y34D9A.8* | 0.757 | S |
| C16C8.14 | *C16C8.14* | 0.756 | S |
| T20B5.3 | *oga-1* | 0.744 | S |
| Y47D3B.7 | *sbp-1* | 0.743 | S |
| F57A10.3 | *haf-3* | 0.74 | S |
| Y71D11A.4 | *Y71D11A.4* | 0.74 | S |
| F22B5.4 | *F22B5.4* | 0.739 | S |
| T05E7.3 | *T05E7.3* | 0.739 | S |
| Y22F5A.3 | *ric-4* | 0.736 | S |
| T20B6.1 | *T20B6.1* | 0.736 | S |
| Y75B12B.5 | *cyn-3* | 0.73 | S |
| Y94H6A.5 | *Y94H6A.5* | 0.726 | S |
| R01H2.6 | *ubc-18* | 0.725 | S |
| T23H2.5 | *rab-10* | 0.724 | S |
| D2045.6 | *cul-1* | 0.724 | S |
| Y49E10.1 | *rpt-6* | 0.724 | S |
| T02H6.1 | *T02H6.1* | 0.724 | S |
| T02C12.3 | *T02C12.3* | 0.72 | S |
| T04D1.3 | *unc-57* | 0.715 | S |
| F48C1.4 | *F48C1.4* | 0.713 | S |
| Y53H1B.2 | *Y53H1B.2* | 0.713 | S |
| H14A12.4 | *mls-1* | 0.713 | S |
| C36B1.11 | *C36B1.11* | 0.712 | S |
| W09D6.4 | *W09D6.4* | 0.712 | S |
| F37C12.12 | *mec-14* | 0.712 | S |
| C30D11.1 | *unc-103* | 0.702 | S |
| B0464.4 | *bre-3* | 0.7 | S |
| R02F2.7 | *R02F2.7* | 0.699 | S |
| Y39E4B.11 | *Y39E4B.11* | 0.697 | S |
| F56A8.7 | *unc-64* | 0.696 | S |
| D1054.3 | *D1054.3* | 0.694 | S |
| B0524.2 | *B0524.2* | 0.692 | S |
| K07D4.7 | *tag-218* | 0.692 | S |
| F57B9.5 | *byn-1* | 0.691 | S |
| W09D10.3 | *W09D10.3* | 0.69 | S |
| F28H1.3 | *ars-2* | 0.689 | S |
| Y105C5A.8 | *Y105C5A.8* | 0.689 | S |
| ZK1236.1 | *ZK1236.1* | 0.688 | S |
| F41B5.4 | *cyp-33C3* | 0.686 | S |
| C07A9.4 | *ncx-6* | 0.683 | S |
| C10A4.8 | *mnm-2* | 0.681 | S |
| T16A9.1 | *T16A9.1* | 0.68 | S |
| C09G1.1 | *pqn-11* | 0.676 | S |
| C40H1.3 | *C40H1.3* | 0.674 | S |
| F55A11.1 | *F55A11.1* | 0.673 | S |
| ZK353.6 | *lap-1* | 0.672 | S |
| T24B8.5 | *T24B8.5* | 0.672 | S |
| T22F3.4 | *rpl-11.1* | 0.671 | S |
| H04M03.1 | *H04M03.1* | 0.671 | S |
| Y56A3A.31 | *Y56A3A.31* | 0.669 | S |
| C36E8.4 | *C36E8.4* | 0.668 | S |
| C39B5.9 | *fbxa-47* | 0.666 | S |
| F10E9.6 | *mig-10* | 0.666 | S |
| C35B1.2 | *C35B1.2* | 0.666 | S |
| F11A10.1 | *lex-1* | 0.665 | S |
| F53E10.4 | *F53E10.4* | 0.664 | S |
| F11C1.2 | *F11C1.2* | 0.661 | S |
| C01F1.5 | *C01F1.5* | 0.659 | S |
| T03F1.11 | *T03F1.11* | 0.659 | S |
| T23G5.3 | *T23G5.3* | 0.657 | S |
| R09E12.6 | *R09E12.6* | 0.653 | S |
| Y65B4BR.5 | *Y65B4BR.5* | 0.652 | S |
| C08F8.2 | *C08F8.2* | 0.652 | S |
| Y37D8A.11 | *Y37D8A.11* | 0.652 | S |
| T05H10.3 | *T05H10.3* | 0.649 | S |
| F21F3.2 | *F21F3.2* | 0.646 | S |
| Y106G6H.9 | *Y106G6H.9* | 0.646 | S |
| F58E2.4 | *F58E2.4* | 0.642 | S |
| Y40H7A.5 | *srd-23* | 0.641 | S |
| Y46H3D.1 | *Y46H3D.1* | 0.639 | S |
| W05B2.1 | *col-94* | 0.638 | S |
| R144.1 | *klp-6* | 0.638 | S |
| C10G11.5 | *pnk-1* | 0.637 | S |
| R07E5.3 | *R07E5.3* | 0.636 | S |
| C53B4.7 | *bre-1* | 0.636 | S |
| Y53G8B.1 | *Y53G8B.1* | 0.635 | S |
| C02B8.4 | *hlh-8* | 0.633 | S |
| C03B8.1 | *C03B8.1* | 0.63 | S |
| F25F8.2 | *glc-2* | 0.629 | S |
| C39D10.7 | *C39D10.7* | 0.628 | S |
| C18C4.3 | *ugt-48* | 0.625 | S |
| Y75B8A.9 | *gly-11* | 0.623 | S |
| EEED8.5 | *mog-5* | 0.62 | S |
| T26C5.3 | *T26C5.3* | 0.62 | S |
| ZK546.4 | *ZK546.4* | 0.62 | S |
| T23B7.1 | *nspd-4* | 0.619 | S |
| C26E6.8 | *ula-1* | 0.619 | S |
| Y47D3A.28 | *Y47D3A.28* | 0.619 | S |
| ZK652.5 | *ceh-23* | 0.618 | S |
| F59B1.8 | *F59B1.8* | 0.614 | S |
| T19D12.9 | *T19D12.9* | 0.614 | S |
| ZK384.2 | *scl-20* | 0.613 | S |
| Y49F6C.1 | *bath-8* | 0.612 | S |
| C39B5.12 | *srd-65* | 0.611 | S |
| Y47D3B.2 | *nlp-21* | 0.606 | S |
| M110.1 | *col-76* | 0.606 | S |
| D1054.10 | *D1054.10* | 0.602 | S |
| Y56A3A.15 | *fbxb-24* | 0.602 | S |
| T05A6.1 | *cki-1* | 0.594 | S |
| M18.8 | *M18.8* | 0.593 | S |
| F26F4.1 | *cee-1* | 0.59 | S |
| C06C6.9 | *C06C6.9* | 0.588 | S |
| Y57A10B.5 | *sre-41* | 0.588 | S |
| F35B12.6 | *tag-290* | 0.587 | S |
| ZK337.2 | *ZK337.2* | 0.586 | S |
| Y58A7A.1 | *Y58A7A.1* | 0.586 | S |
| Y34D9A.6 | *glrx-10* | 0.585 | S |
| Y54E10BL.5 | *nduf-5* | 0.581 | S |
| T01B7.6 | *T01B7.6* | 0.581 | S |
| ZK507.1 | *ZK507.1* | 0.579 | S |
| F57B9.8 | *F57B9.8* | 0.579 | S |
| F09G8.2 | *tag-198* | 0.579 | S |
| F37A8.2 | *F37A8.2* | 0.579 | S |
| Y113G7A.9 | *dcs-1* | 0.579 | S |
| K04D7.5 | *gon-4* | 0.577 | S |
| T04A6.2 | *T04A6.2* | 0.576 | S |
| T25G3.1 | *T25G3.1* | 0.575 | S |
| R74.8 | *R74.8* | 0.575 | S |
| ZK131.7 | *his-13* | 0.573 | S |
| Y47D3A.6 | *tra-1* | 0.572 | S |
| ZK512.7 | *ZK512.7* | 0.572 | S |
| C02D5.2 | *C02D5.2* | 0.572 | S |
| ZK1127.10 | *ZK1127.10* | 0.57 | S |
| Y41E3.5 | *Y41E3.5* | 0.569 | S |
| F29B9.6 | *ubc-9* | 0.567 | S |
| R08D7.3 | *eif-3.D* | 0.565 | S |
| C08B6.9 | *aos-1* | 0.564 | S |
| T17H7.1 | *T17H7.1* | 0.563 | S |
| F22F1.1 | *hil-3* | 0.562 | S |
| R11A5.6 | *R11A5.6* | 0.562 | S |
| T27F2.1 | *skp-1* | 0.562 | S |
| Y75B8A.34 | *Y75B8A.34* | 0.562 | S |
| T20H9.3 | *fbxa-71* | 0.562 | S |
| F17C11.4 | *F17C11.4* | 0.56 | S |
| F54G8.2 | *dgk-3* | 0.559 | S |
| Y38F1A.9 | *oig-2* | 0.559 | S |
| F55C7.2 | *F55C7.2* | 0.554 | S |
| T28D6.3 | *T28D6.3* | 0.553 | S |
| R05F9.1 | *R05F9.1* | 0.553 | S |
| K01C8.3 | *tdc-1* | 0.552 | S |
| B0035.8 | *his-48* | 0.552 | S |
| R05D3.8 | *R05D3.8* | 0.551 | S |
| T07E3.2 | *T07E3.2* | 0.551 | S |
| F38A5.10 | *nspb-4* | 0.55 | S |
| F28C6.10 | *F28C6.10* | 0.549 | S |
| F19B6.2 | *ufd-1* | 0.549 | S |
| C36E8.5 | *tbb-2* | 0.549 | S |
| T22G5.2 | *lbp-7* | 0.549 | S |
| C50F4.6 | *C50F4.6* | 0.548 | S |
| M01E11.2 | *M01E11.2* | 0.547 | S |
| K09E4.4 | *K09E4.4* | 0.545 | S |
| F02H6.3 | *F02H6.3* | 0.545 | S |
| F10F2.5 | *clec-154* | 0.545 | S |
| T07C4.5 | *ttr-15* | 0.544 | S |
| F49C5.4 | *F49C5.4* | 0.543 | S |
| W01H2.3 | *rab-37* | 0.543 | S |
| ZK673.3 | *ZK673.3* | 0.541 | S |
| F53H4.4 | *F53H4.4* | 0.541 | S |
| C09G9.7 | *C09G9.7* | 0.541 | S |
| ZK673.7 | *tnc-2* | 0.541 | S |
| F54C9.7 | *F54C9.7* | 0.541 | S |
| M88.3 | *M88.3* | 0.54 | S |
| C51E3.6 | *C51E3.6* | 0.539 | S |
| B0348.6 | *ife-3* | 0.537 | S |
| F25H2.12 | *F25H2.12* | 0.535 | S |
| K01A2.5 | *K01A2.5* | 0.534 | S |
| ZK512.5 | *ZK512.5* | 0.534 | S |
| W02B3.2 | *grk-2* | 0.534 | S |
| Y42G9A.6 | *wht-7* | 0.534 | S |
| C10C5.4 | *C10C5.4* | 0.533 | S |
| C06B3.4 | *stdh-1* | 0.533 | S |
| F14F3.1 | *vab-3* | 0.533 | S |
| F36F2.1 | *F36F2.1* | 0.532 | S |
| K11H3.5 | *K11H3.5* | 0.53 | S |
| R13A5.6 | *ttr-8* | 0.53 | S |
| ZK520.4 | *cul-2* | 0.529 | S |
| C46E10.7 | *srh-99* | 0.529 | S |
| ZK1251.6 | *msp-76* | 0.529 | S |
| F23B12.6 | *tag-114* | 0.529 | S |
| C04F12.5 | *C04F12.5* | 0.528 | S |
| F26E4.2 | *F26E4.2* | 0.527 | S |
| T08G2.3 | *T08G2.3* | 0.525 | S |
| LLC1.3 | *LLC1.3* | 0.523 | S |
| T26A5.8 | *T26A5.8* | 0.52 | S |
| F15E6.3 | *F15E6.3* | 0.519 | S |
| F25B5.6 | *F25B5.6* | 0.517 | S |
| F33D11.10 | *F33D11.10* | 0.517 | S |
| ZC395.8 | *ztf-8* | 0.516 | S |
| F11G11.2 | *gst-7* | 0.515 | S |
| C44B11.1 | *C44B11.1* | 0.515 | S |
| C17G10.5 | *lys-8* | 0.513 | S |
| C39B5.3 | *fbxa-62* | 0.512 | S |
| K08E4.2 | *K08E4.2* | 0.511 | S |
| Y37D8A.16 | *Y37D8A.16* | 0.51 | S |
| T23G7.2 | *T23G7.2* | 0.509 | S |
| C25D7.3 | *sdc-3* | 0.507 | S |
| F42G9.7 | *snt-2* | 0.507 | S |
| Y38F1A.10 | *max-2* | 0.505 | S |
| T02G5.7 | *T02G5.7* | 0.503 | S |
| F45G2.10 | *F45G2.10* | 0.502 | S |
| F59B2.8 | *F59B2.8* | 0.502 | S |
| F25F2.1 | *F25F2.1* | 0.501 | S |
| F37C12.1 | *F37C12.1* | 0.501 | S |
| K08E3.8 | *mdt-29* | 0.501 | S |
| Y51A2B.1 | *Y51A2B.1* | 0.5 | S |
| F08B12.4 | *F08B12.4* | 0.5 | S |
| F49E12.2 | *dod-23* | 0.497 | S |
| C08F11.12 | *C08F11.12* | 0.496 | S |
| R13A5.10 | *R13A5.10* | 0.496 | S |
| C47D2.2 | *cdd-1* | 0.496 | S |
| R13A5.4 | *lgc-12* | 0.496 | S |
| K04A8.5 | *K04A8.5* | 0.495 | S |
| F54C8.2 | *cpar-1* | 0.495 | S |
| Y50D7A.11 | *Y50D7A.11* | 0.493 | S |
| T28A8.1 | *twk-40* | 0.493 | S |
| F42A6.7 | *hrp-1* | 0.491 | S |
| F17C8.2 | *col-89* | 0.49 | S |
| F39B2.10 | *dnj-12* | 0.49 | S |
| K03A1.5 | *sur-5* | 0.489 | S |
| Y42H9AR.3 | *rabs-5* | 0.489 | S |
| Y38E10A.14 | *Y38E10A.14* | 0.487 | S |
| F54C9.6 | *F54C9.6* | 0.487 | S |
| T19C3.5 | *T19C3.5* | 0.487 | S |
| F48E8.6 | *F48E8.6* | 0.486 | S |
| F40F4.3 | *lbp-1* | 0.482 | S |
| K04C2.3 | *K04C2.3* | 0.482 | S |
| R09B5.8 | *cnc-3* | 0.481 | S |
| F25H2.2 | *F25H2.2* | 0.48 | NE |
| B0336.11 | *B0336.11* | 0.479 | NE |
| ZK652.11 | *cuc-1* | 0.478 | NE |
| T14F9.3 | *hex-1* | 0.477 | NE |
| F11A5.12 | *stdh-2* | 0.477 | NE |
| T16H12.2 | *T16H12.2* | 0.477 | NE |
| F28D1.5 | *thn-2* | 0.476 | NE |
| F40F8.1 | *F40F8.1* | 0.476 | NE |
| T28B4.4 | *T28B4.4* | 0.476 | NE |
| F54E7.5 | *sdz-21* | 0.476 | NE |
| R08E5.4 | *R08E5.4* | 0.476 | NE |
| C05D10.4 | *C05D10.4* | 0.475 | NE |
| C05D11.5 | *C05D11.5* | 0.474 | NE |
| C24F3.2 | *C24F3.2* | 0.474 | NE |
| T20H4.2 | *T20H4.2* | 0.473 | NE |
| ZK643.2 | *ZK643.2* | 0.473 | NE |
| T21C12.4 | *T21C12.4* | 0.473 | NE |
| R09D1.3 | *R09D1.3* | 0.471 | NE |
| Y51A2D.9 | *ttr-24* | 0.469 | NE |
| F56C9.3 | *F56C9.3* | 0.469 | NE |
| R05F9.10 | *sgt-1* | 0.468 | NE |
| Y44E3A.2 | *ace-2* | 0.467 | NE |
| W08E3.1 | *snr-2* | 0.466 | NE |
| W03A5.3 | *grl-22* | 0.466 | NE |
| K03H1.11 | *K03H1.11* | 0.466 | NE |
| T05G5.8 | *T05G5.8* | 0.465 | NE |
| Y71D11A.3 | *Y71D11A.3* | 0.465 | NE |
| B0334.1 | *B0334.1* | 0.463 | NE |
| F25D1.3 | *F25D1.3* | 0.463 | NE |
| F40E10.4 | *slt-1* | 0.462 | NE |
| R148.4 | *R148.4* | 0.462 | NE |
| H38K22.2 | *dcn-1* | 0.462 | NE |
| B0286.3 | *B0286.3* | 0.46 | NE |
| C52E2.2 | *C52E2.2* | 0.46 | NE |
| R06F6.11 | *tag-209* | 0.46 | NE |
| C34F11.1 | *C34F11.1* | 0.458 | NE |
| R06A4.9 | *R06A4.9* | 0.458 | NE |
| F32A5.4 | *F32A5.4* | 0.457 | NE |
| F37B1.1 | *gst-24* | 0.455 | NE |
| K10B2.4 | *K10B2.4* | 0.455 | NE |
| B0285.9 | *B0285.9* | 0.453 | NE |
| T20G5.10 | *T20G5.10* | 0.453 | NE |
| B0304.4 | *B0304.4* | 0.453 | NE |
| C50B8.1 | *C50B8.1* | 0.452 | NE |
| F45C12.2 | *F45C12.2* | 0.452 | NE |
| ZK688.2 | *ZK688.2* | 0.452 | NE |
| T09A5.9 | *T09A5.9* | 0.451 | NE |
| Y48A6B.7 | *Y48A6B.7* | 0.45 | NE |
| Y39E4A.3 | *Y39E4A.3* | 0.449 | NE |
| F53C3.6 | *F53C3.6* | 0.449 | NE |
| D2007.2 | *D2007.2* | 0.446 | NE |
| ZK112.4 | *ZK112.4* | 0.446 | NE |
| F20H11.4 | *F20H11.4* | 0.446 | NE |
| T12D8.5 | *T12D8.5* | 0.445 | NE |
| T28B8.2 | *ins-18* | 0.444 | NE |
| D1037.3 | *ftn-2* | 0.443 | NE |
| F53F1.4 | *F53F1.4* | 0.443 | NE |
| F54C8.1 | *F54C8.1* | 0.443 | NE |
| B0213.5 | *nlp-30* | 0.442 | NE |
| T08B2.11 | *T08B2.11* | 0.442 | NE |
| R01H2.2 | *R01H2.2* | 0.442 | NE |
| F07F6.1 | *F07F6.1* | 0.441 | NE |
| F52H3.5 | *F52H3.5* | 0.439 | NE |
| Y41E3.7 | *Y41E3.7* | 0.438 | NE |
| R06C1.4 | *R06C1.4* | 0.438 | NE |
| ZK1236.2 | *cec-1* | 0.438 | NE |
| Y71H2B.3 | *ppfr-3* | 0.438 | NE |
| F42A10.8 | *nas-28* | 0.437 | NE |
| B0478.1 | *jnk-1* | 0.436 | NE |
| Y39A1A.24 | *Y39A1A.24* | 0.433 | NE |
| C44B9.2 | *C44B9.2* | 0.433 | NE |
| M02D8.4 | *M02D8.4* | 0.432 | NE |
| ZK675.4 | *ZK675.4* | 0.432 | NE |
| K08F11.5 | *K08F11.5* | 0.431 | NE |
| C23G10.7 | *C23G10.7* | 0.431 | NE |
| F48E8.1 | *lon-1* | 0.43 | NE |
| Y18D10A.20 | *pfn-1* | 0.43 | NE |
| F27C8.6 | *F27C8.6* | 0.43 | NE |
| B0213.4 | *nlp-29* | 0.429 | NE |
| ZC239.16 | *ZC239.16* | 0.428 | NE |
| T12G3.1 | *T12G3.1* | 0.428 | NE |
| C01G6.5 | *C01G6.5* | 0.427 | NE |
| F32D8.6 | *emo-1* | 0.427 | NE |
| H41C03.1 | *H41C03.1* | 0.427 | NE |
| Y71F9AL.1 | *Y71F9AL.1* | 0.427 | NE |
| ZK1128.3 | *ZK1128.3* | 0.426 | NE |
| F37C4.5 | *F37C4.5* | 0.425 | NE |
| T09B4.5 | *T09B4.5* | 0.423 | NE |
| F47D12.5 | *F47D12.5* | 0.423 | NE |
| C48B4.4 | *ced-7* | 0.423 | NE |
| T04B8.2 | *T04B8.2* | 0.422 | NE |
| C14F5.1 | *dct-1* | 0.422 | NE |
| Y43F8C.9 | *Y43F8C.9* | 0.421 | NE |
| F26E4.1 | *sur-6* | 0.421 | NE |
| F44D12.8 | *F44D12.8* | 0.421 | NE |
| C01B12.8 | *C01B12.8* | 0.421 | NE |
| F46F11.4 | *ubl-5* | 0.421 | NE |
| T06G6.9 | *pfd-3* | 0.42 | NE |
| Y39A1A.7 | *Y39A1A.7* | 0.42 | NE |
| C44E4.6 | *acbp-1* | 0.42 | NE |
| B0304.2 | *B0304.2* | 0.419 | NE |
| K04F10.7 | *K04F10.7* | 0.419 | NE |
| ZC395.7 | *hap-1* | 0.419 | NE |
| C26B2.1 | *C26B2.1* | 0.414 | NE |
| K07A1.12 | *lin-53* | 0.414 | NE |
| F55C12.1 | *F55C12.1* | 0.413 | NE |
| C06E8.4 | *C06E8.4* | 0.413 | NE |
| T19B4.5 | *T19B4.5* | 0.413 | NE |
| D1054.15 | *tag-135* | 0.413 | NE |
| F46B6.7 | *ztf-7* | 0.413 | NE |
| T06D4.1 | *T06D4.1* | 0.412 | NE |
| C03E10.6 | *clec-222* | 0.412 | NE |
| K08F4.1 | *K08F4.1* | 0.411 | NE |
| F55G1.15 | *F55G1.15* | 0.411 | NE |
| B0457.2 | *B0457.2* | 0.411 | NE |
| Y55F3C.6 | *Y55F3C.6* | 0.411 | NE |
| F34D10.4 | *F34D10.4* | 0.41 | NE |
| C27A7.7 | *srj-4* | 0.41 | NE |
| Y48A6B.8 | *Y48A6B.8* | 0.409 | NE |
| ZC204.2 | *ZC204.2* | 0.408 | NE |
| Y71F9B.4 | *snr-7* | 0.407 | NE |
| F17A9.3 | *F17A9.3* | 0.407 | NE |
| C18E9.4 | *C18E9.4* | 0.407 | NE |
| F59A7.2 | *F59A7.2* | 0.407 | NE |
| T26E3.7 | *T26E3.7* | 0.407 | NE |
| T02G5.6 | *msh-4* | 0.407 | NE |
| Y47H9C.12 | *Y47H9C.12* | 0.406 | NE |
| K08A2.4 | *K08A2.4* | 0.405 | NE |
| Y57G11C.14 | *Y57G11C.14* | 0.403 | NE |
| C05D2.4 | *bas-1* | 0.403 | NE |
| ZK525.1 | *flp-15* | 0.403 | NE |
| C02D5.1 | *C02D5.1* | 0.403 | NE |
| T12B5.4 | *fbxa-11* | 0.403 | NE |
| C34B2.2 | *kbp-5* | 0.401 | NE |
| ZK1307.9 | *ZK1307.9* | 0.401 | NE |
| Y43C5A.2 | *Y43C5A.2* | 0.398 | NE |
| R07E5.2 | *prdx-3* | 0.397 | NE |
| D2096.2 | *praf-3* | 0.395 | NE |
| C47G2.2 | *unc-130* | 0.394 | NE |
| Y49E10.21 | *Y49E10.21* | 0.394 | NE |
| F07F6.5 | *dct-5* | 0.393 | NE |
| C38D4.9 | *C38D4.9* | 0.392 | NE |
| Y71F9AM.5 | *nxt-1* | 0.391 | NE |
| Y55D9A.1 | *efa-6* | 0.39 | NE |
| C38C6.2 | *atgp-2* | 0.389 | NE |
| R10E4.7 | *R10E4.7* | 0.389 | NE |
| T04G9.4 | *T04G9.4* | 0.388 | NE |
| D1022.4 | *D1022.4* | 0.387 | NE |
| K04H4.5 | *K04H4.5* | 0.385 | NE |
| R07B1.1 | *vab-15* | 0.384 | NE |
| Y79H2A.11 | *zyg-8* | 0.384 | NE |
| C18D4.8 | *C18D4.8* | 0.382 | NE |
| T19C3.4 | *T19C3.4* | 0.382 | NE |
| F45G2.6 | *trf-1* | 0.381 | NE |
| M176.3 | *M176.3* | 0.381 | NE |
| R07E5.7 | *R07E5.7* | 0.381 | NE |
| C01F1.3 | *C01F1.3* | 0.38 | NE |
| ZK892.4 | *ZK892.4* | 0.38 | NE |
| Y48G8AR.3 | *Y48G8AR.3* | 0.379 | NE |
| B0336.7 | *B0336.7* | 0.378 | NE |
| F59F4.2 | *F59F4.2* | 0.378 | NE |
| W09C5.2 | *unc-59* | 0.378 | NE |
| R11E3.8 | *dpf-5* | 0.378 | NE |
| C56G2.6 | *let-767* | 0.378 | NE |
| C05D11.12 | *let-721* | 0.378 | NE |
| C24H11.8 | *twk-39* | 0.378 | NE |
| K04C2.4 | *brd-1* | 0.378 | NE |
| F57H12.7 | *mec-17* | 0.377 | NE |
| T12B5.10 | *fbxa-60* | 0.377 | NE |
| C49D10.2 | *nhr-166* | 0.377 | NE |
| C08B11.2 | *hda-2* | 0.376 | NE |
| Y49E10.20 | *Y49E10.20* | 0.376 | NE |
| C14F11.1 | *C14F11.1* | 0.375 | NE |
| C50C3.7 | *C50C3.7* | 0.374 | NE |
| R17.3 | *R17.3* | 0.374 | NE |
| Y56A3A.18 | *Y56A3A.18* | 0.374 | NE |
| R01H10.5 | *R01H10.5* | 0.374 | NE |
| Y54G9A.3 | *kqt-3* | 0.374 | NE |
| ZK370.5 | *pdhk-2* | 0.374 | NE |
| F43G9.5 | *F43G9.5* | 0.373 | NE |
| T09B4.1 | *T09B4.1* | 0.371 | NE |
| ZK593.6 | *lgg-2* | 0.371 | NE |
| Y105C5B.13 | *skr-10* | 0.37 | NE |
| K05C4.7 | *K05C4.7* | 0.37 | NE |
| T17A3.7 | *fbxb-84* | 0.37 | NE |
| C29F9.13 | *nhr-280* | 0.369 | NE |
| D2045.7 | *D2045.7* | 0.368 | NE |
| F37B1.7 | *gst-18* | 0.368 | NE |
| T21C9.8 | *ttr-23* | 0.367 | NE |
| T24B8.2 | *T24B8.2* | 0.367 | NE |
| T20H4.1 | *osm-10* | 0.366 | NE |
| M04B2.4 | *M04B2.4* | 0.366 | NE |
| ZC21.8 | *ZC21.8* | 0.366 | NE |
| R12B2.5 | *mdt-15* | 0.362 | NE |
| K02B12.2 | *K02B12.2* | 0.362 | NE |
| F53C11.4 | *F53C11.4* | 0.362 | NE |
| Y55B1AR.1 | *lec-6* | 0.362 | NE |
| W06F12.3 | *W06F12.3* | 0.362 | NE |
| C18A3.1 | *C18A3.1* | 0.361 | NE |
| Y110A2AL.9 | *Y110A2AL.9* | 0.361 | NE |
| K03H1.12 | *K03H1.12* | 0.361 | NE |
| R11.1 | *R11.1* | 0.36 | NE |
| C32D5.8 | *C32D5.8* | 0.36 | NE |
| F53A2.3 | *F53A2.3* | 0.36 | NE |
| ZK632.8 | *arl-5* | 0.358 | NE |
| B0303.7 | *B0303.7* | 0.358 | NE |
| F57C2.4 | *F57C2.4* | 0.357 | NE |
| Y42G9A.2 | *Y42G9A.2* | 0.356 | NE |
| F08C6.3 | *tag-197* | 0.355 | NE |
| Y106G6H.7 | *sec-8* | 0.354 | NE |
| C05B5.3 | *pqn-8* | 0.354 | NE |
| Y66A7A.4 | *Y66A7A.4* | 0.354 | NE |
| C16A3.10 | *C16A3.10* | 0.354 | NE |
| F55B12.1 | *ceh-24* | 0.352 | NE |
| Y43C5B.2 | *Y43C5B.2* | 0.351 | NE |
| Y54G2A.31 | *ubc-13* | 0.351 | NE |
| T02E9.5 | *T02E9.5* | 0.351 | NE |
| C09G5.7 | *C09G5.7* | 0.35 | NE |
| C17D12.5 | *C17D12.5* | 0.35 | NE |
| C26E6.7 | *C26E6.7* | 0.347 | NE |
| W04C9.2 | *W04C9.2* | 0.347 | NE |
| K01C8.2 | *K01C8.2* | 0.347 | NE |
| F57A10.2 | *F57A10.2* | 0.346 | NE |
| T21B10.7 | *cct-2* | 0.346 | NE |
| F43C1.2 | *mpk-1* | 0.346 | NE |
| F09B12.3 | *F09B12.3* | 0.345 | NE |
| R09H10.3 | *R09H10.3* | 0.345 | NE |
| C40C9.5 | *nlg-1* | 0.344 | NE |
| C13B9.3 | *C13B9.3* | 0.344 | NE |
| C08F11.14 | *C08F11.14* | 0.342 | NE |
| F47G9.1 | *F47G9.1* | 0.341 | NE |
| ZK1128.8 | *vps-29* | 0.341 | NE |
| LLC1.2 | *LLC1.2* | 0.34 | NE |
| Y75B8A.35 | *zip-1* | 0.339 | NE |
| M01D7.5 | *nlp-12* | 0.339 | NE |
| C34G6.3 | *C34G6.3* | 0.339 | NE |
| F35G12.10 | *asb-1* | 0.339 | NE |
| C43E11.9 | *C43E11.9* | 0.339 | NE |
| F42C5.7 | *grl-4* | 0.337 | NE |
| ZK484.5 | *ZK484.5* | 0.337 | NE |
| B0280.4 | *B0280.4* | 0.337 | NE |
| F01F1.14 | *F01F1.14* | 0.337 | NE |
| F23B2.3 | *F23B2.3* | 0.336 | NE |
| E04F6.13 | *srd-58* | 0.336 | NE |
| B0336.6 | *B0336.6* | 0.336 | NE |
| C46F4.2 | *acs-17* | 0.335 | NE |
| C28A5.3 | *nex-3* | 0.335 | NE |
| Y41C4A.10 | *elb-1* | 0.335 | NE |
| R05D3.3 | *R05D3.3* | 0.334 | NE |
| C46C2.1 | *wnk-1* | 0.334 | NE |
| C35D10.2 | *C35D10.2* | 0.333 | NE |
| C54F6.13 | *nhx-3* | 0.333 | NE |
| ZK757.3 | *tag-76* | 0.332 | NE |
| Y53G8AM.7 | *Y53G8AM.7* | 0.332 | NE |
| F54D10.1 | *skr-15* | 0.331 | NE |
| H20J04.8 | *sap-1* | 0.33 | NE |
| T13F2.1 | *fat-4* | 0.328 | NE |
| F56D5.5 | *F56D5.5* | 0.328 | NE |
| C08E3.3 | *bath-33* | 0.328 | NE |
| C09F12.1 | *clc-1* | 0.327 | NE |
| F56F3.1 | *pqn-45* | 0.327 | NE |
| Y59E9AR.4 | *thn-5* | 0.325 | NE |
| R11D1.8 | *rpl-28* | 0.325 | NE |
| F54C8.6 | *F54C8.6* | 0.325 | NE |
| C15C8.7 | *C15C8.7* | 0.325 | NE |
| ZK1058.3 | *ZK1058.3* | 0.324 | NE |
| F42C5.8 | *rps-8* | 0.324 | NE |
| C17H12.1 | *dyci-1* | 0.323 | NE |
| F14D7.8 | *F14D7.8* | 0.323 | NE |
| M01D1.7 | *fbxb-39* | 0.323 | NE |
| Y54E10A.2 | *cogc-1* | 0.322 | NE |
| T06D8.7 | *T06D8.7* | 0.322 | NE |
| C05C8.3 | *fkb-3* | 0.321 | NE |
| Y55F3AM.13 | *Y55F3AM.13* | 0.321 | NE |
| T13B5.5 | *lips-11* | 0.32 | NE |
| T07C4.1 | *T07C4.1* | 0.32 | NE |
| K01C8.1 | *K01C8.1* | 0.32 | NE |
| F12E12.3 | *F12E12.3* | 0.32 | NE |
| F40G9.3 | *ubc-20* | 0.318 | NE |
| C38D4.4 | *C38D4.4* | 0.318 | NE |
| K07D4.2 | *K07D4.2* | 0.318 | NE |
| F44F4.2 | *egg-3* | 0.318 | NE |
| C16A3.4 | *C16A3.4* | 0.318 | NE |
| D2096.8 | *D2096.8* | 0.317 | NE |
| F25H2.5 | *F25H2.5* | 0.317 | NE |
| C28H8.9 | *C28H8.9* | 0.317 | NE |
| Y106G6H.8 | *Y106G6H.8* | 0.316 | NE |
| Y53C10A.2 | *Y53C10A.2* | 0.316 | NE |
| C06A8.6 | *C06A8.6* | 0.316 | NE |
| C14B1.4 | *tag-125* | 0.315 | NE |
| B0024.9 | *trx-2* | 0.315 | NE |
| C28H8.5 | *C28H8.5* | 0.314 | NE |
| T23B5.1 | *T23B5.1* | 0.314 | NE |
| F21C10.10 | *F21C10.10* | 0.313 | NE |
| F33D4.1 | *nhr-8* | 0.313 | NE |
| F22E5.3 | *gcy-21* | 0.313 | NE |
| PAR2.1 | *mtss-1* | 0.312 | NE |
| B0035.12 | *B0035.12* | 0.312 | NE |
| Y54E10BR.4 | *Y54E10BR.4* | 0.311 | NE |
| T05A7.2 | *clec-140* | 0.31 | NE |
| B0432.6 | *B0432.6* | 0.31 | NE |
| Y51B9A.3 | *Y51B9A.3* | 0.31 | NE |
| C26B9.5 | *C26B9.5* | 0.309 | NE |
| M18.2 | *dlc-2* | 0.308 | NE |
| F25H9.6 | *F25H9.6* | 0.308 | NE |
| B0303.3 | *B0303.3* | 0.308 | NE |
| W05F2.2 | *W05F2.2* | 0.308 | NE |
| K10D2.3 | *cid-1* | 0.308 | NE |
| C48D1.3 | *cho-1* | 0.307 | NE |
| F43D9.5 | *taf-11.3* | 0.306 | NE |
| T12D8.8 | *T12D8.8* | 0.306 | NE |
| C48E7.2 | *C48E7.2* | 0.306 | NE |
| ZK484.4 | *ZK484.4* | 0.305 | NE |
| C01B4.7 | *C01B4.7* | 0.305 | NE |
| M04D8.2 | *ins-22* | 0.304 | NE |
| C05D11.11 | *mel-32* | 0.304 | NE |
| Y70G10A.2 | *Y70G10A.2* | 0.304 | NE |
| ZK1128.5 | *tag-246* | 0.303 | NE |
| F30A10.7 | *F30A10.7* | 0.303 | NE |
| C52D10.11 | *flp-17* | 0.302 | NE |
| M28.6 | *lact-3* | 0.302 | NE |
| T01G9.6 | *kin-10* | 0.3 | NE |
| C35D10.7 | *C35D10.7* | 0.299 | NE |
| F26E4.9 | *cco-1* | 0.299 | NE |
| C07B5.5 | *nuc-1* | 0.298 | NE |
| Y119D3B.15 | *dss-1* | 0.298 | NE |
| T23B5.3 | *T23B5.3* | 0.298 | NE |
| W05H9.1 | *W05H9.1* | 0.297 | NE |
| F38E11.2 | *hsp-12.6* | 0.297 | NE |
| K07F5.11 | *ssq-1* | 0.296 | NE |
| Y66A7A.2 | *Y66A7A.2* | 0.296 | NE |
| T10F2.2 | *T10F2.2* | 0.296 | NE |
| C05C8.6 | *C05C8.6* | 0.293 | NE |
| C14B9.1 | *hsp-12.2* | 0.293 | NE |
| F25H5.4 | *eft-2* | 0.292 | NE |
| Y39G8B.2 | *Y39G8B.2* | 0.292 | NE |
| R05D11.6 | *R05D11.6* | 0.292 | NE |
| F38E11.3 | *cpin-1* | 0.291 | NE |
| Y48A6B.6 | *Y48A6B.6* | 0.291 | NE |
| C49C3.8 | *C49C3.8* | 0.29 | NE |
| F32B6.2 | *F32B6.2* | 0.29 | NE |
| H04M03.2 | *nspb-6* | 0.29 | NE |
| AC3.7 | *ugt-1* | 0.289 | NE |
| W02F12.3 | *W02F12.3* | 0.289 | NE |
| C06E8.5 | *C06E8.5* | 0.289 | NE |
| F55G1.8 | *plk-3* | 0.289 | NE |
| Y56A3A.3 | *mif-1* | 0.289 | NE |
| 3R5.1 | *3R5.1* | 0.289 | NE |
| C44F1.5 | *acy-3* | 0.289 | NE |
| Y57A10A.9 | *tag-276* | 0.289 | NE |
| ZK1025.5 | *ZK1025.5* | 0.289 | NE |
| K09H9.3 | *col-49* | 0.288 | NE |
| K02F6.1 | *K02F6.1* | 0.287 | NE |
| F13D12.8 | *F13D12.8* | 0.287 | NE |
| C47E12.7 | *C47E12.7* | 0.286 | NE |
| T14G10.8 | *T14G10.8* | 0.285 | NE |
| T11G6.4 | *T11G6.4* | 0.285 | NE |
| Y41E3.1 | *Y41E3.1* | 0.285 | NE |
| Y57G7A.6 | *Y57G7A.6* | 0.285 | NE |
| Y71H2AL.1 | *Y71H2AL.1* | 0.285 | NE |
| B0218.1 | *B0218.1* | 0.284 | NE |
| B0280.9 | *B0280.9* | 0.284 | NE |
| Y22D7AR.6 | *Y22D7AR.6* | 0.282 | NE |
| F32D8.7 | *F32D8.7* | 0.281 | NE |
| K11H12.6 | *K11H12.6* | 0.281 | NE |
| C08E8.4 | *C08E8.4* | 0.28 | NE |
| C34E10.8 | *C34E10.8* | 0.28 | NE |
| F31C3.6 | *F31C3.6* | 0.279 | NE |
| F37B1.4 | *gst-15* | 0.278 | NE |
| C47E12.4 | *pyp-1* | 0.278 | NE |
| ZK354.7 | *ZK354.7* | 0.278 | NE |
| C06E1.6 | *fipr-16* | 0.277 | NE |
| W02B3.7 | *W02B3.7* | 0.277 | NE |
| T05A12.2 | *tre-2* | 0.277 | NE |
| H19N07.4 | *H19N07.4* | 0.277 | NE |
| B0554.3 | *B0554.3* | 0.277 | NE |
| F26A3.6 | *del-3* | 0.276 | NE |
| C30G12.7 | *puf-8* | 0.276 | NE |
| F15A4.3 | *sre-37* | 0.276 | NE |
| F18A11.3 | *F18A11.3* | 0.276 | NE |
| C08E3.7 | *fbxa-164* | 0.276 | NE |
| T12B5.8 | *fbxa-59* | 0.275 | NE |
| F22F4.2 | *inx-3* | 0.275 | NE |
| F45D11.13 | *fbxb-30* | 0.274 | NE |
| C46F11.3 | *C46F11.3* | 0.274 | NE |
| K12H6.1 | *nhr-119* | 0.274 | NE |
| T19C3.6 | *T19C3.6* | 0.274 | NE |
| F45H10.4 | *drr-1* | 0.274 | NE |
| F59E12.5 | *npl-4.2* | 0.274 | NE |
| K03H1.3 | *ttr-3* | 0.274 | NE |
| T12B5.12 | *fbxa-70* | 0.274 | NE |
| C39E9.5 | *scl-7* | 0.273 | NE |
| C15F1.8 | *C15F1.8* | 0.273 | NE |
| Y39G10AL.3 | *cdk-7* | 0.272 | NE |
| C08F11.11 | *C08F11.11* | 0.272 | NE |
| T09A5.5 | *T09A5.5* | 0.272 | NE |
| C07A9.7 | *set-3* | 0.271 | NE |
| W03A3.1 | *ceh-10* | 0.271 | NE |
| F48E8.7 | *skpt-1* | 0.271 | NE |
| C23H3.1 | *egl-26* | 0.271 | NE |
| K09E4.2 | *K09E4.2* | 0.271 | NE |
| T26E3.6 | *T26E3.6* | 0.27 | NE |
| F44B9.4 | *cit-1.1* | 0.27 | NE |
| E02H9.5 | *E02H9.5* | 0.27 | NE |
| C26E6.9 | *set-2* | 0.27 | NE |
| F56C4.1 | *F56C4.1* | 0.269 | NE |
| F23F12.4 | *sdz-15* | 0.269 | NE |
| H14A12.2 | *fum-1* | 0.269 | NE |
| W05H7.3 | *sedl-1* | 0.268 | NE |
| C44H4.6 | *C44H4.6* | 0.268 | NE |
| C54E4.2 | *C54E4.2* | 0.267 | NE |
| F28H7.3 | *F28H7.3* | 0.267 | NE |
| K10D2.4 | *K10D2.4* | 0.267 | NE |
| H10D18.2 | *scl-12* | 0.266 | NE |
| F25B4.9 | *clec-1* | 0.266 | NE |
| ZK652.1 | *snr-5* | 0.266 | NE |
| Y116A8C.26 | *Y116A8C.26* | 0.265 | NE |
| F08F1.8 | *tth-1* | 0.265 | NE |
| F35G2.3 | *F35G2.3* | 0.265 | NE |
| C08F1.6 | *C08F1.6* | 0.264 | NE |
| Y51H7C.7 | *Y51H7C.7* | 0.264 | NE |
| K10D2.5 | *K10D2.5* | 0.264 | NE |
| Y41E3.3 | *Y41E3.3* | 0.264 | NE |
| T12B5.1 | *fbxa-51* | 0.262 | NE |
| Y69E1A.8 | *Y69E1A.8* | 0.262 | NE |
| F08H9.2 | *F08H9.2* | 0.261 | NE |
| D2007.4 | *D2007.4* | 0.26 | NE |
| F32A5.3 | *F32A5.3* | 0.259 | NE |
| F13G3.10 | *F13G3.10* | 0.259 | NE |
| Y62E10A.6 | *Y62E10A.6* | 0.258 | NE |
| B0432.10 | *B0432.10* | 0.256 | NE |
| F53F1.5 | *F53F1.5* | 0.256 | NE |
| R12H7.2 | *asp-4* | 0.255 | NE |
| ZK792.3 | *inx-9* | 0.255 | NE |
| Y46G5A.8 | *Y46G5A.8* | 0.255 | NE |
| ZC482.1 | *gab-1* | 0.254 | NE |
| F53F8.3 | *F53F8.3* | 0.254 | NE |
| F15H10.3 | *apc-10* | 0.253 | NE |
| F58G1.1 | *F58G1.1* | 0.253 | NE |
| F46C5.1 | *F46C5.1* | 0.252 | NE |
| K10B4.4 | *K10B4.4* | 0.252 | NE |
| B0035.16 | *B0035.16* | 0.252 | NE |
| C01A2.5 | *C01A2.5* | 0.251 | NE |
| Y38H6C.1 | *dct-16* | 0.249 | NE |
| T22D1.10 | *ruvb-2* | 0.248 | NE |
| T02G5.12 | *T02G5.12* | 0.248 | NE |
| R05F9.5 | *gst-9* | 0.247 | NE |
| Y38F1A.5 | *cyd-1* | 0.246 | NE |
| Y54G11A.3 | *Y54G11A.3* | 0.246 | NE |
| F19H6.4 | *F19H6.4* | 0.246 | NE |
| F37A4.1 | *F37A4.1* | 0.246 | NE |
| F54G8.3 | *ina-1* | 0.246 | NE |
| C06G4.4 | *C06G4.4* | 0.246 | NE |
| F01F1.4 | *rabn-5* | 0.245 | NE |
| C27C7.1 | *C27C7.1* | 0.244 | NE |
| F29D11.2 | *F29D11.2* | 0.244 | NE |
| F59E12.10 | *ddl-1* | 0.243 | NE |
| C18E9.10 | *C18E9.10* | 0.243 | NE |
| Y11D7A.4 | *rab-28* | 0.242 | NE |
| C47D12.6 | *trs-1* | 0.242 | NE |
| T21C9.4 | *T21C9.4* | 0.241 | NE |
| F15H10.2 | *col-13* | 0.241 | NE |
| C25A1.6 | *C25A1.6* | 0.24 | NE |
| Y47G6A.13 | *Y47G6A.13* | 0.239 | NE |
| B0361.5 | *psd-1* | 0.239 | NE |
| ZK512.4 | *ZK512.4* | 0.238 | NE |
| F46C8.2 | *col-174* | 0.238 | NE |
| F54D5.3 | *F54D5.3* | 0.238 | NE |
| F35G12.1 | *F35G12.1* | 0.238 | NE |
| ZK112.1 | *pcp-1* | 0.238 | NE |
| C33F10.11 | *C33F10.11* | 0.238 | NE |
| ZK1307.8 | *ZK1307.8* | 0.237 | NE |
| T24H10.2 | *T24H10.2* | 0.237 | NE |
| R04D3.3 | *R04D3.3* | 0.237 | NE |
| K08A2.2 | *K08A2.2* | 0.236 | NE |
| Y55B1BR.4 | *Y55B1BR.4* | 0.236 | NE |
| F23F1.3 | *F23F1.3* | 0.236 | NE |
| T22G5.5 | *sptl-3* | 0.235 | NE |
| F09G2.8 | *F09G2.8* | 0.235 | NE |
| F32H5.3 | *F32H5.3* | 0.235 | NE |
| F26E4.12 | *F26E4.12* | 0.234 | NE |
| K02B7.1 | *K02B7.1* | 0.234 | NE |
| Y47D3A.11 | *wht-8* | 0.234 | NE |
| ZC155.2 | *ZC155.2* | 0.234 | NE |
| F52C6.8 | *bath-4* | 0.232 | NE |
| ZK909.4 | *ces-2* | 0.232 | NE |
| ZK546.15 | *try-1* | 0.231 | NE |
| F58E1.2 | *fbxb-25* | 0.231 | NE |
| Y47D3B.9 | *Y47D3B.9* | 0.231 | NE |
| F41H10.10 | *htp-1* | 0.23 | NE |
| Y39A1A.20 | *Y39A1A.20* | 0.23 | NE |
| C23H3.9 | *C23H3.9* | 0.229 | NE |
| K05F1.7 | *msp-63* | 0.229 | NE |
| ZK593.5 | *dnc-1* | 0.229 | NE |
| C05C12.4 | *C05C12.4* | 0.229 | NE |
| W05B2.5 | *col-93* | 0.229 | NE |
| F57G8.3 | *srh-167* | 0.227 | NE |
| D1046.2 | *D1046.2* | 0.227 | NE |
| C26B2.8 | *C26B2.8* | 0.227 | NE |
| C09F9.3 | *C09F9.3* | 0.227 | NE |
| F17E9.3 | *F17E9.3* | 0.226 | NE |
| Y38E10A.19 | *nhr-235* | 0.226 | NE |
| F26H11.5 | *exl-1* | 0.225 | NE |
| R106.2 | *R106.2* | 0.225 | NE |
| F40F8.4 | *F40F8.4* | 0.225 | NE |
| F13E9.7 | *F13E9.7* | 0.224 | NE |
| T15B12.1 | *T15B12.1* | 0.224 | NE |
| C09B9.6 | *msp-55* | 0.224 | NE |
| F28A10.6 | *F28A10.6* | 0.224 | NE |
| T28D6.6 | *T28D6.6* | 0.224 | NE |
| W06E11.5 | *tag-266* | 0.224 | NE |
| Y39H10A.7 | *chk-1* | 0.224 | NE |
| Y57A10A.23 | *Y57A10A.23* | 0.223 | NE |
| D2062.6 | *D2062.6* | 0.223 | NE |
| F46F5.15 | *F46F5.15* | 0.223 | NE |
| Y76B12C.4 | *Y76B12C.4* | 0.222 | NE |
| K08H2.6 | *hpl-1* | 0.221 | NE |
| Y46G5A.23 | *Y46G5A.23* | 0.22 | NE |
| F37A8.5 | *F37A8.5* | 0.22 | NE |
| B0496.7 | *valv-1* | 0.219 | NE |
| Y116A8C.30 | *Y116A8C.30* | 0.219 | NE |
| K01G5.6 | *rib-2* | 0.219 | NE |
| ZK669.3 | *ZK669.3* | 0.219 | NE |
| C44C11.1 | *ras-1* | 0.218 | NE |
| C43E11.7 | *ndx-7* | 0.217 | NE |
| F54C8.7 | *F54C8.7* | 0.216 | NE |
| Y70G10A.3 | *Y70G10A.3* | 0.215 | NE |
| R13F6.8 | *clec-158* | 0.214 | NE |
| F23H11.7 | *F23H11.7* | 0.214 | NE |
| T13A10.13 | *srv-33* | 0.214 | NE |
| R05H10.5 | *R05H10.5* | 0.214 | NE |
| M05D6.6 | *M05D6.6* | 0.213 | NE |
| D2013.8 | *scp-1* | 0.213 | NE |
| C50E10.8 | *sre-56* | 0.212 | NE |
| T21B10.2 | *enol-1* | 0.212 | NE |
| C44B7.6 | *C44B7.6* | 0.212 | NE |
| B0416.4 | *B0416.4* | 0.212 | NE |
| F44F4.3 | *F44F4.3* | 0.212 | NE |
| T13A10.5 | *nlp-16* | 0.212 | NE |
| ZK1010.2 | *ZK1010.2* | 0.212 | NE |
| F54H12.4 | *F54H12.4* | 0.212 | NE |
| Y45F10C.3 | *fbxa-215* | 0.211 | NE |
| H18N23.2 | *H18N23.2* | 0.211 | NE |
| M195.3 | *amt-3* | 0.211 | NE |
| C25E10.8 | *C25E10.8* | 0.211 | NE |
| T01C3.2 | *T01C3.2* | 0.211 | NE |
| Y34D9A.7 | *Y34D9A.7* | 0.21 | NE |
| F58D5.1 | *hrp-2* | 0.209 | NE |
| F32E10.1 | *nol-10* | 0.209 | NE |
| ZK354.6 | *ZK354.6* | 0.209 | NE |
| Y71F9B.1 | *Y71F9B.1* | 0.208 | NE |
| C03C10.5 | *C03C10.5* | 0.208 | NE |
| Y71H2B.4 | *Y71H2B.4* | 0.207 | NE |
| W08D2.1 | *egl-20* | 0.207 | NE |
| F59H6.8 | *bath-21* | 0.206 | NE |
| Y44A6C.2 | *Y44A6C.2* | 0.206 | NE |
| Y105C5B.5 | *Y105C5B.5* | 0.206 | NE |
| R11A8.2 | *R11A8.2* | 0.205 | NE |
| C06E1.4 | *glr-1* | 0.205 | NE |
| K09B11.4 | *K09B11.4* | 0.203 | NE |
| D1081.7 | *D1081.7* | 0.203 | NE |
| H10E21.5 | *H10E21.5* | 0.202 | NE |
| T20H9.2 | *fbxa-43* | 0.202 | NE |
| C56G2.3 | *C56G2.3* | 0.202 | NE |
| F09G2.9 | *F09G2.9* | 0.201 | NE |
| F48C1.2 | *fbxa-145* | 0.201 | NE |
| T07A9.6 | *daf-18* | 0.201 | NE |
| T07E3.5 | *brc-2* | 0.201 | NE |
| K11D2.2 | *asah-1* | 0.201 | NE |
| K06H7.7 | *K06H7.7* | 0.201 | NE |
| K10F12.5 | *K10F12.5* | 0.2 | NE |
| H31G24.1 | *H31G24.1* | 0.2 | NE |
| ZK353.2 | *ZK353.2* | 0.2 | NE |
| F08D12.2 | *F08D12.2* | 0.2 | NE |
| Y106G6D.4 | *Y106G6D.4* | 0.2 | NE |
| C02F5.8 | *tsp-1* | 0.199 | NE |
| Y39F10A.1 | *Y39F10A.1* | 0.199 | NE |
| F11A6.1 | *kpc-1* | 0.198 | NE |
| Y71H2AM.23 | *tufm-1* | 0.198 | NE |
| F37A4.5 | *F37A4.5* | 0.198 | NE |
| R74.3 | *xbp-1* | 0.198 | NE |
| R09B3.1 | *exo-3* | 0.198 | NE |
| W02B12.1 | *W02B12.1* | 0.197 | NE |
| T15H9.3 | *hlh-6* | 0.197 | NE |
| C54G4.5 | *C54G4.5* | 0.197 | NE |
| W03F11.6 | *afd-1* | 0.196 | NE |
| F33A8.5 | *sdhd-1* | 0.196 | NE |
| C49H3.12 | *C49H3.12* | 0.196 | NE |
| C28H8.2 | *C28H8.2* | 0.196 | NE |
| T14B4.5 | *T14B4.5* | 0.196 | NE |
| T28D6.9 | *pen-2* | 0.196 | NE |
| T10D4.4 | *ins-31* | 0.195 | NE |
| R107.1 | *nac-2* | 0.195 | NE |
| T05C12.8 | *T05C12.8* | 0.195 | NE |
| Y39A1A.15 | *cnt-2* | 0.194 | NE |
| F10B5.4 | *tub-1* | 0.194 | NE |
| C16A11.7 | *C16A11.7* | 0.193 | NE |
| F54F3.3 | *F54F3.3* | 0.193 | NE |
| F46C5.8 | *rer-1* | 0.193 | NE |
| C15F1.7 | *sod-1* | 0.193 | NE |
| R09B5.9 | *cnc-4* | 0.193 | NE |
| F40H6.1 | *F40H6.1* | 0.193 | NE |
| T04A8.15 | *T04A8.15* | 0.192 | NE |
| C48E7.9 | *twk-37* | 0.192 | NE |
| Y54E2A.4 | *Y54E2A.4* | 0.191 | NE |
| F46A9.5 | *skr-1* | 0.191 | NE |
| K04H4.6 | *crn-6* | 0.19 | NE |
| F49E12.9 | *F49E12.9* | 0.19 | NE |
| F45F2.4 | *his-7* | 0.189 | NE |
| C28H8.4 | *C28H8.4* | 0.189 | NE |
| F56F12.1 | *F56F12.1* | 0.189 | NE |
| R02F2.6 | *R02F2.6* | 0.189 | NE |
| ZK525.2 | *aqp-11* | 0.188 | NE |
| ZC482.5 | *lgc-37* | 0.188 | NE |
| C24H11.5 | *C24H11.5* | 0.188 | NE |
| K06A1.3 | *K06A1.3* | 0.188 | NE |
| H12I19.4 | *H12I19.4* | 0.188 | NE |
| C06E2.5 | *C06E2.5* | 0.187 | NE |
| C09E8.3 | *mlt-10* | 0.187 | NE |
| R12E2.13 | *R12E2.13* | 0.187 | NE |
| M7.5 | *atg-7* | 0.187 | NE |
| F55C5.7 | *F55C5.7* | 0.187 | NE |
| C29F9.2 | *C29F9.2* | 0.187 | NE |
| F20D1.1 | *F20D1.1* | 0.187 | NE |
| F44F4.4 | *ptr-8* | 0.186 | NE |
| C38D4.7 | *C38D4.7* | 0.186 | NE |
| F57F5.1 | *F57F5.1* | 0.185 | NE |
| C17E7.4 | *C17E7.4* | 0.185 | NE |
| R13F6.1 | *kbp-1* | 0.185 | NE |
| C01G12.3 | *C01G12.3* | 0.184 | NE |
| H06I04.1 | *H06I04.1* | 0.184 | NE |
| T20B12.2 | *tbp-1* | 0.184 | NE |
| F46F6.2 | *F46F6.2* | 0.184 | NE |
| C14B1.8 | *C14B1.8* | 0.184 | NE |
| T22D1.4 | *T22D1.4* | 0.184 | NE |
| C50F2.10 | *abf-2* | 0.183 | NE |
| C44B9.4 | *athp-1* | 0.183 | NE |
| F21D5.4 | *F21D5.4* | 0.182 | NE |
| Y56A3A.22 | *Y56A3A.22* | 0.182 | NE |
| B0280.10 | *B0280.10* | 0.181 | NE |
| F43D9.2 | *rab-33* | 0.181 | NE |
| F45E1.7 | *sdpn-1* | 0.181 | NE |
| C32H11.12 | *dod-24* | 0.181 | NE |
| T07A5.3 | *T07A5.3* | 0.18 | NE |
| C18A3.5 | *C18A3.5* | 0.18 | NE |
| Y110A2AR.1 | *Y110A2AR.1* | 0.18 | NE |
| Y75B12B.6 | *plc-2* | 0.179 | NE |
| R13A1.9 | *R13A1.9* | 0.179 | NE |
| F54E7.6 | *F54E7.6* | 0.178 | NE |
| Y111B2A.8 | *Y111B2A.8* | 0.178 | NE |
| F44E5.1 | *F44E5.1* | 0.177 | NE |
| Y105C5B.11 | *Y105C5B.11* | 0.177 | NE |
| C55A6.9 | *C55A6.9* | 0.177 | NE |
| C56E6.3 | *C56E6.3* | 0.177 | NE |
| Y42H9AR.2 | *Y42H9AR.2* | 0.177 | NE |
| C34D4.8 | *str-48* | 0.177 | NE |
| K10G6.1 | *lin-31* | 0.177 | NE |
| Y19D10B.6 | *Y19D10B.6* | 0.176 | NE |
| B0410.2 | *vang-1* | 0.176 | NE |
| ZK287.1 | *ZK287.1* | 0.176 | NE |
| Y54E5A.8 | *Y54E5A.8* | 0.175 | NE |
| Y53F4B.19 | *Y53F4B.19* | 0.175 | NE |
| B0303.14 | *B0303.14* | 0.175 | NE |
| B0284.4 | *B0284.4* | 0.175 | NE |
| F16H9.1 | *rgs-2* | 0.174 | NE |
| C40H1.5 | *ttr-5* | 0.174 | NE |
| F49H12.3 | *F49H12.3* | 0.174 | NE |
| T22H6.2 | *T22H6.2* | 0.173 | NE |
| C30C11.4 | *C30C11.4* | 0.172 | NE |
| T20D4.5 | *T20D4.5* | 0.172 | NE |
| F15A4.6 | *F15A4.6* | 0.172 | NE |
| ZK1010.8 | *ZK1010.8* | 0.172 | NE |
| F54G8.1 | *F54G8.1* | 0.171 | NE |
| F02E9.7 | *F02E9.7* | 0.17 | NE |
| ZK632.13 | *lin-52* | 0.17 | NE |
| C39B5.11 | *srd-67* | 0.17 | NE |
| F11E6.4 | *F11E6.4* | 0.169 | NE |
| T04C9.4 | *mlp-1* | 0.169 | NE |
| M18.6 | *M18.6* | 0.169 | NE |
| F22B5.5 | *F22B5.5* | 0.169 | NE |
| F35C5.4 | *F35C5.4* | 0.168 | NE |
| W07B8.1 | *W07B8.1* | 0.168 | NE |
| T24H10.3 | *dnj-23* | 0.167 | NE |
| C23G10.1 | *C23G10.1* | 0.167 | NE |
| T13C2.2 | *T13C2.2* | 0.167 | NE |
| R11G1.3 | *gst-11* | 0.167 | NE |
| Y18H1A.6 | *pif-1* | 0.167 | NE |
| R05D3.6 | *R05D3.6* | 0.167 | NE |
| Y45F3A.4 | *Y45F3A.4* | 0.167 | NE |
| C32F10.2 | *lin-35* | 0.166 | NE |
| C10H11.7 | *C10H11.7* | 0.166 | NE |
| ZK632.10 | *ZK632.10* | 0.166 | NE |
| D2023.7 | *col-158* | 0.165 | NE |
| ZK177.3 | *ZK177.3* | 0.165 | NE |
| C32B5.5 | *C32B5.5* | 0.165 | NE |
| D1005.2 | *D1005.2* | 0.165 | NE |
| C33H5.14 | *ntp-1* | 0.164 | NE |
| W06F12.2 | *W06F12.2* | 0.164 | NE |
| R08D7.5 | *R08D7.5* | 0.164 | NE |
| ZC53.7 | *rgs-9* | 0.164 | NE |
| C13F10.6 | *C13F10.6* | 0.164 | NE |
| T05H4.12 | *atp-4* | 0.164 | NE |
| K02E2.6 | *K02E2.6* | 0.163 | NE |
| C47D12.7 | *kel-1* | 0.163 | NE |
| F35D2.3 | *F35D2.3* | 0.162 | NE |
| F42G2.5 | *F42G2.5* | 0.161 | NE |
| F22B7.13 | *gpr-1* | 0.161 | NE |
| ZK1127.9 | *ZK1127.9* | 0.161 | NE |
| C34E10.3 | *gop-1* | 0.161 | NE |
| M176.2 | *M176.2* | 0.161 | NE |
| W09G10.6 | *clec-125* | 0.161 | NE |
| B0495.2 | *B0495.2* | 0.161 | NE |
| C24H12.6 | *C24H12.6* | 0.161 | NE |
| M176.6 | *kin-15* | 0.161 | NE |
| C50C3.9 | *unc-36* | 0.161 | NE |
| H02I12.7 | *his-65* | 0.16 | NE |
| C04B4.2 | *C04B4.2* | 0.158 | NE |
| F36A4.1 | *F36A4.1* | 0.158 | NE |
| K07H8.3 | *K07H8.3* | 0.158 | NE |
| Y46G5A.19 | *Y46G5A.19* | 0.158 | NE |
| JC8.8 | *ttr-51* | 0.158 | NE |
| Y51H4A.18 | *Y51H4A.18* | 0.158 | NE |
| C54G4.9 | *C54G4.9* | 0.158 | NE |
| F52C9.6 | *F52C9.6* | 0.157 | NE |
| C06E1.5 | *fip-3* | 0.157 | NE |
| C47A4.5 | *C47A4.5* | 0.157 | NE |
| F21D5.2 | *F21D5.2* | 0.157 | NE |
| F32D1.2 | *F32D1.2* | 0.157 | NE |
| C34D4.4 | *C34D4.4* | 0.156 | NE |
| F49E11.1 | *mbk-2* | 0.156 | NE |
| F32B6.6 | *msp-77* | 0.155 | NE |
| C43F9.7 | *C43F9.7* | 0.155 | NE |
| F35D11.10 | *clec-139* | 0.155 | NE |
| F09F7.7 | *F09F7.7* | 0.155 | NE |
| B0213.6 | *nlp-31* | 0.154 | NE |
| C07G1.3 | *pct-1* | 0.154 | NE |
| F36A4.4 | *F36A4.4* | 0.154 | NE |
| F13B12.1 | *F13B12.1* | 0.154 | NE |
| K04D7.1 | *rack-1* | 0.154 | NE |
| B0286.5 | *B0286.5* | 0.154 | NE |
| C47G2.4 | *C47G2.4* | 0.154 | NE |
| C04G6.3 | *pld-1* | 0.153 | NE |
| K06H7.2 | *K06H7.2* | 0.153 | NE |
| C38C10.3 | *C38C10.3* | 0.153 | NE |
| F11A5.10 | *glc-1* | 0.153 | NE |
| F40H6.4 | *tbx-11* | 0.153 | NE |
| H06I04.7 | *fipr-29* | 0.153 | NE |
| F10F2.1 | *sel-2* | 0.153 | NE |
| T07C4.3 | *T07C4.3* | 0.153 | NE |
| F57A8.1 | *F57A8.1* | 0.153 | NE |
| K09B3.1 | *K09B3.1* | 0.152 | NE |
| C46C2.5 | *C46C2.5* | 0.152 | NE |
| F13D12.5 | *F13D12.5* | 0.152 | NE |
| Y5F2A.4 | *Y5F2A.4* | 0.151 | NE |
| ZK632.1 | *mcm-6* | 0.151 | NE |
| C06A8.4 | *skr-17* | 0.151 | NE |
| ZK742.5 | *lbp-4* | 0.151 | NE |
| F35C12.2 | *ncx-4* | 0.15 | NE |
| R166.3 | *R166.3* | 0.149 | NE |
| C10C6.3 | *C10C6.3* | 0.148 | NE |
| F52E4.1 | *pccb-1* | 0.147 | NE |
| F53A3.7 | *F53A3.7* | 0.147 | NE |
| T20B5.1 | *apa-2* | 0.147 | NE |
| C14A4.9 | *C14A4.9* | 0.147 | NE |
| T18D3.1 | *T18D3.1* | 0.146 | NE |
| Y39A3CL.5 | *clp-4* | 0.146 | NE |
| F33D4.4 | *F33D4.4* | 0.146 | NE |
| T10C6.5 | *T10C6.5* | 0.146 | NE |
| F35G12.4 | *F35G12.4* | 0.146 | NE |
| C01A2.4 | *C01A2.4* | 0.146 | NE |
| Y69E1A.2 | *Y69E1A.2* | 0.146 | NE |
| ZK512.2 | *ZK512.2* | 0.146 | NE |
| H20J04.3 | *H20J04.3* | 0.145 | NE |
| C05C10.4 | *C05C10.4* | 0.145 | NE |
| F49C12.14 | *F49C12.14* | 0.145 | NE |
| M28.5 | *M28.5* | 0.145 | NE |
| C18E3.5 | *C18E3.5* | 0.145 | NE |
| B0334.7 | *B0334.7* | 0.145 | NE |
| B0336.4 | *rgs-5* | 0.145 | NE |
| R03C1.1 | *R03C1.1* | 0.144 | NE |
| F53G12.11 | *F53G12.11* | 0.144 | NE |
| W09C5.4 | *ins-33* | 0.144 | NE |
| R01H2.1 | *R01H2.1* | 0.144 | NE |
| AC3.5 | *AC3.5* | 0.143 | NE |
| C07A12.4 | *pdi-2* | 0.143 | NE |
| C28A5.2 | *C28A5.2* | 0.143 | NE |
| EEED8.13 | *EEED8.13* | 0.142 | NE |
| M88.4 | *M88.4* | 0.142 | NE |
| K08F9.2 | *tag-216* | 0.142 | NE |
| R119.7 | *rnp-8* | 0.142 | NE |
| F52A8.3 | *F52A8.3* | 0.141 | NE |
| W01C9.4 | *W01C9.4* | 0.141 | NE |
| T08E11.1 | *T08E11.1* | 0.141 | NE |
| ZK849.2 | *ZK849.2* | 0.141 | NE |
| C06E1.9 | *C06E1.9* | 0.141 | NE |
| Y38F1A.4 | *Y38F1A.4* | 0.14 | NE |
| E03A3.3 | *his-69* | 0.14 | NE |
| F44G4.5 | *F44G4.5* | 0.14 | NE |
| Y56A3A.30 | *Y56A3A.30* | 0.14 | NE |
| ZC482.6 | *srw-10* | 0.14 | NE |
| ZK328.7 | *ZK328.7* | 0.139 | NE |
| F33H1.5 | *srd-1* | 0.139 | NE |
| C41H7.3 | *C41H7.3* | 0.139 | NE |
| K10G6.2 | *dos-2* | 0.139 | NE |
| T24E12.11 | *T24E12.11* | 0.139 | NE |
| R07C3.9 | *R07C3.9* | 0.138 | NE |
| M02B1.2 | *M02B1.2* | 0.138 | NE |
| B0213.2 | *nlp-27* | 0.138 | NE |
| C01F6.4 | *fem-3* | 0.138 | NE |
| B0546.4 | *B0546.4* | 0.138 | NE |
| W01B6.9 | *ndc-80* | 0.137 | NE |
| ZK546.14 | *ZK546.14* | 0.137 | NE |
| T01B7.8 | *T01B7.8* | 0.137 | NE |
| W03D8.3 | *W03D8.3* | 0.136 | NE |
| Y116F11B.1 | *daf-28* | 0.135 | NE |
| Y17G7B.17 | *Y17G7B.17* | 0.135 | NE |
| M142.4 | *vab-7* | 0.134 | NE |
| F58G1.2 | *F58G1.2* | 0.134 | NE |
| Y39A1A.21 | *Y39A1A.21* | 0.134 | NE |
| F42H11.2 | *lem-3* | 0.133 | NE |
| F56D2.4 | *uev-2* | 0.133 | NE |
| F40G9.11 | *mxl-2* | 0.132 | NE |
| M88.5 | *M88.5* | 0.132 | NE |
| K01G5.9 | *K01G5.9* | 0.132 | NE |
| R09B5.3 | *cnc-2* | 0.132 | NE |
| ZK262.5 | *ZK262.5* | 0.132 | NE |
| F35E8.3 | *F35E8.3* | 0.131 | NE |
| T04F8.8 | *T04F8.8* | 0.131 | NE |
| C55B6.2 | *dnj-7* | 0.131 | NE |
| F11C7.3 | *vap-1* | 0.131 | NE |
| F45F2.11 | *F45F2.11* | 0.13 | NE |
| F53H8.2 | *arr-1* | 0.13 | NE |
| C29E4.1 | *col-90* | 0.13 | NE |
| H14E04.1 | *H14E04.1* | 0.13 | NE |
| T07D4.2 | *T07D4.2* | 0.129 | NE |
| T04A8.7 | *T04A8.7* | 0.129 | NE |
| Y40H7A.11 | *Y40H7A.11* | 0.129 | NE |
| T19C3.9 | *ttr-7* | 0.129 | NE |
| T20B12.7 | *T20B12.7* | 0.129 | NE |
| D2007.1 | *D2007.1* | 0.129 | NE |
| H20J04.6 | *H20J04.6* | 0.128 | NE |
| F35D2.4 | *F35D2.4* | 0.128 | NE |
| F59B8.1 | *F59B8.1* | 0.128 | NE |
| F58A4.6 | *F58A4.6* | 0.128 | NE |
| F14H12.1 | *col-165* | 0.127 | NE |
| F12F6.8 | *F12F6.8* | 0.127 | NE |
| C29F9.12 | *C29F9.12* | 0.127 | NE |
| DH11.2 | *DH11.2* | 0.127 | NE |
| C36F7.1 | *irx-1* | 0.126 | NE |
| F01D5.6 | *F01D5.6* | 0.126 | NE |
| Y54F10AL.2 | *smg-6* | 0.126 | NE |
| F29C4.2 | *F29C4.2* | 0.125 | NE |
| Y57G7A.9 | *glb-31* | 0.125 | NE |
| C29F9.1 | *C29F9.1* | 0.125 | NE |
| R01H2.4 | *R01H2.4* | 0.125 | NE |
| F14D12.2 | *unc-97* | 0.125 | NE |
| R09D1.5 | *R09D1.5* | 0.124 | NE |
| Y18D10A.17 | *car-1* | 0.123 | NE |
| T01B7.5 | *T01B7.5* | 0.123 | NE |
| C46E10.5 | *C46E10.5* | 0.123 | NE |
| F36H1.4 | *lin-3* | 0.123 | NE |
| F56D2.2 | *F56D2.2* | 0.123 | NE |
| C24D10.5 | *C24D10.5* | 0.122 | NE |
| T02G5.2 | *T02G5.2* | 0.122 | NE |
| K11G12.5 | *K11G12.5* | 0.122 | NE |
| F56E10.4 | *rps-27* | 0.122 | NE |
| Y53G8B.3 | *Y53G8B.3* | 0.122 | NE |
| T07C12.7 | *ttr-46* | 0.121 | NE |
| C16A11.4 | *C16A11.4* | 0.121 | NE |
| C01F6.5 | *aly-1* | 0.12 | NE |
| ZK177.6 | *fzy-1* | 0.12 | NE |
| F22B8.7 | *F22B8.7* | 0.12 | NE |
| C25A1.8 | *clec-87* | 0.12 | NE |
| C13C12.1 | *cal-1* | 0.12 | NE |
| F47C12.6 | *F47C12.6* | 0.119 | NE |
| C15H9.7 | *C15H9.7* | 0.119 | NE |
| C07E3.6 | *C07E3.6* | 0.119 | NE |
| B0523.3 | *pgl-2* | 0.119 | NE |
| C34F11.4 | *msp-50* | 0.119 | NE |
| T28D9.9 | *T28D9.9* | 0.118 | NE |
| F02A9.3 | *far-2* | 0.118 | NE |
| T01B7.7 | *rol-6* | 0.117 | NE |
| R06F6.8 | *R06F6.8* | 0.117 | NE |
| F10F2.3 | *lips-3* | 0.117 | NE |
| T01H3.2 | *T01H3.2* | 0.117 | NE |
| K04C1.5 | *K04C1.5* | 0.117 | NE |
| C27H6.8 | *C27H6.8* | 0.117 | NE |
| F56C9.1 | *gsp-2* | 0.117 | NE |
| D2096.5 | *D2096.5* | 0.117 | NE |
| C24G6.1 | *syp-2* | 0.117 | NE |
| T22B7.1 | *egl-13* | 0.117 | NE |
| K07A1.3 | *K07A1.3* | 0.117 | NE |
| M18.3 | *M18.3* | 0.116 | NE |
| E02H9.3 | *E02H9.3* | 0.116 | NE |
| C23G10.4 | *rpn-2* | 0.116 | NE |
| C47G2.1 | *cut-1* | 0.115 | NE |
| H12C20.2 | *pms-2* | 0.115 | NE |
| T10B9.8 | *cyp-13A1* | 0.115 | NE |
| W09D10.4 | *W09D10.4* | 0.114 | NE |
| C49G7.4 | *phat-3* | 0.114 | NE |
| F47D12.6 | *F47D12.6* | 0.114 | NE |
| ZC302.2 | *ZC302.2* | 0.113 | NE |
| F55B11.4 | *F55B11.4* | 0.113 | NE |
| F59B2.3 | *F59B2.3* | 0.113 | NE |
| C17F4.3 | *C17F4.3* | 0.112 | NE |
| C40H1.4 | *elo-4* | 0.112 | NE |
| Y69A2AL.1 | *grd-16* | 0.112 | NE |
| Y51H7BR.2 | *fbxb-43* | 0.111 | NE |
| ZC204.15 | *srw-97* | 0.11 | NE |
| F32B4.1 | *F32B4.1* | 0.11 | NE |
| F08A10.1 | *F08A10.1* | 0.109 | NE |
| T24H10.4 | *T24H10.4* | 0.109 | NE |
| F25H2.9 | *pas-5* | 0.108 | NE |
| JC8.6 | *lin-54* | 0.108 | NE |
| B0491.2 | *sqt-1* | 0.107 | NE |
| F40E3.5 | *F40E3.5* | 0.107 | NE |
| Y110A2AL.6 | *Y110A2AL.6* | 0.106 | NE |
| K02F3.1 | *tag-51* | 0.106 | NE |
| F31E8.3 | *tab-1* | 0.106 | NE |
| M88.6 | *pan-1* | 0.105 | NE |
| K02B7.3 | *K02B7.3* | 0.104 | NE |
| C47D12.2 | *C47D12.2* | 0.104 | NE |
| F47F2.1 | *F47F2.1* | 0.103 | NE |
| F58E1.14 | *fbxb-47* | 0.102 | NE |
| C06A8.8 | *C06A8.8* | 0.102 | NE |
| Y53F4B.7 | *Y53F4B.7* | 0.102 | NE |
| F26G1.4 | *ttm-2* | 0.102 | NE |
| ZK1053.4 | *ZK1053.4* | 0.102 | NE |
| F17C11.7 | *F17C11.7* | 0.101 | NE |
| T04D3.1 | *T04D3.1* | 0.1 | NE |
| D2005.3 | *D2005.3* | 0.099 | NE |
| C30A5.4 | *C30A5.4* | 0.099 | NE |
| Y54E5A.4 | *npp-4* | 0.099 | NE |
| C49A1.5 | *C49A1.5* | 0.099 | NE |
| Y54G2A.19 | *Y54G2A.19* | 0.099 | NE |
| C24B9.9 | *dod-3* | 0.098 | NE |
| K08E7.3 | *let-99* | 0.098 | NE |
| C18H9.6 | *C18H9.6* | 0.097 | NE |
| ZC168.4 | *cyb-1* | 0.097 | NE |
| C14B9.3 | *C14B9.3* | 0.097 | NE |
| W04G3.5 | *W04G3.5* | 0.097 | NE |
| C01G8.2 | *cln-3.2* | 0.097 | NE |
| R02D5.1 | *R02D5.1* | 0.097 | NE |
| EEED8.12 | *EEED8.12* | 0.097 | NE |
| F40H7.2 | *srx-108* | 0.097 | NE |
| H38K22.3 | *tag-131* | 0.097 | NE |
| F42G9.5 | *alh-11* | 0.096 | NE |
| Y74C9A.3 | *Y74C9A.3* | 0.096 | NE |
| T20G5.7 | *dod-6* | 0.094 | NE |
| C13G3.3 | *pptr-2* | 0.094 | NE |
| Y73F8A.14 | *Y73F8A.14* | 0.094 | NE |
| F13D11.2 | *hbl-1* | 0.094 | NE |
| C34E10.9 | *C34E10.9* | 0.094 | NE |
| R11H6.5 | *R11H6.5* | 0.093 | NE |
| F14E5.1 | *F14E5.1* | 0.093 | NE |
| W10G11.9 | *srh-60* | 0.093 | NE |
| Y73C8B.4 | *lag-2* | 0.092 | NE |
| Y39A1B.1 | *clec-163* | 0.092 | NE |
| T03D8.7 | *T03D8.7* | 0.092 | NE |
| K02B2.5 | *rps-25* | 0.092 | NE |
| C01G8.1 | *C01G8.1* | 0.092 | NE |
| F22B7.4 | *fip-1* | 0.092 | NE |
| F28D1.2 | *F28D1.2* | 0.091 | NE |
| C35D10.6 | *C35D10.6* | 0.091 | NE |
| F07H5.7 | *F07H5.7* | 0.091 | NE |
| H14A12.3 | *H14A12.3* | 0.091 | NE |
| R04F11.5 | *R04F11.5* | 0.09 | NE |
| R07G3.8 | *R07G3.8* | 0.09 | NE |
| C28H8.7 | *C28H8.7* | 0.09 | NE |
| F36A4.10 | *col-34* | 0.09 | NE |
| F39B2.7 | *F39B2.7* | 0.09 | NE |
| C14F11.5 | *hsp-43* | 0.09 | NE |
| F17C8.4 | *ras-2* | 0.089 | NE |
| F53C11.5 | *F53C11.5* | 0.089 | NE |
| Y77E11A.6 | *Y77E11A.6* | 0.089 | NE |
| F36H5.2 | *math-27* | 0.088 | NE |
| C24H12.5 | *C24H12.5* | 0.088 | NE |
| C06B8.8 | *rpl-38* | 0.088 | NE |
| F01D4.5 | *F01D4.5* | 0.087 | NE |
| Y87G2A.9 | *ubc-14* | 0.087 | NE |
| R12E2.4 | *inx-17* | 0.087 | NE |
| C29F3.2 | *wrt-8* | 0.086 | NE |
| Y48B6A.14 | *hmg-1.1* | 0.086 | NE |
| D1054.2 | *pas-2* | 0.086 | NE |
| Y92C3A.1 | *Y92C3A.1* | 0.086 | NE |
| F43G6.9 | *patr-1* | 0.086 | NE |
| ZK632.6 | *cnx-1* | 0.086 | NE |
| C44B7.8 | *pmp-1* | 0.085 | NE |
| C35D10.11 | *C35D10.11* | 0.085 | NE |
| C33A12.10 | *sru-4* | 0.085 | NE |
| BE10.2 | *BE10.2* | 0.085 | NE |
| R05F9.9 | *R05F9.9* | 0.085 | NE |
| W10G11.2 | *W10G11.2* | 0.085 | NE |
| F01D5.9 | *cyp-37A1* | 0.084 | NE |
| R06A4.7 | *mes-2* | 0.084 | NE |
| Y48C3A.14 | *Y48C3A.14* | 0.084 | NE |
| ZC376.5 | *trm-1* | 0.084 | NE |
| F56D2.7 | *ced-6* | 0.084 | NE |
| F42G4.6 | *F42G4.6* | 0.084 | NE |
| C35D6.4 | *C35D6.4* | 0.084 | NE |
| K11E8.1 | *unc-43* | 0.084 | NE |
| F26H9.4 | *F26H9.4* | 0.084 | NE |
| F54D12.3 | *aat-7* | 0.084 | NE |
| B0432.11 | *B0432.11* | 0.083 | NE |
| R07B7.2 | *R07B7.2* | 0.083 | NE |
| ZK1320.2 | *ZK1320.2* | 0.083 | NE |
| F02E9.1 | *F02E9.1* | 0.083 | NE |
| F25D7.1 | *cup-2* | 0.083 | NE |
| C04F12.10 | *fce-1* | 0.083 | NE |
| F07A11.2 | *F07A11.2* | 0.083 | NE |
| C55B7.5 | *uri-1* | 0.082 | NE |
| F28H7.2 | *F28H7.2* | 0.082 | NE |
| F14F7.1 | *col-98* | 0.082 | NE |
| Y49A3A.2 | *vha-13* | 0.082 | NE |
| F46F5.14 | *F46F5.14* | 0.082 | NE |
| T27D12.3 | *clec-144* | 0.082 | NE |
| F52C6.4 | *F52C6.4* | 0.081 | NE |
| T14D7.3 | *tag-212* | 0.081 | NE |
| F37B1.3 | *gst-14* | 0.081 | NE |
| C07H4.1 | *C07H4.1* | 0.081 | NE |
| C43F9.6 | *C43F9.6* | 0.08 | NE |
| Y43C5A.3 | *Y43C5A.3* | 0.08 | NE |
| F22D6.14 | *F22D6.14* | 0.08 | NE |
| K09E4.1 | *K09E4.1* | 0.08 | NE |
| T21C9.13 | *T21C9.13* | 0.079 | NE |
| C18E3.9 | *C18E3.9* | 0.079 | NE |
| Y48E1B.13 | *csp-1* | 0.079 | NE |
| C08F8.3 | *C08F8.3* | 0.079 | NE |
| C12D8.11 | *rop-1* | 0.079 | NE |
| C28D4.3 | *gln-6* | 0.079 | NE |
| ZC477.5 | *ZC477.5* | 0.078 | NE |
| ZK856.10 | *ZK856.10* | 0.078 | NE |
| K08F4.3 | *K08F4.3* | 0.078 | NE |
| W08A12.2 | *W08A12.2* | 0.078 | NE |
| ZK1127.2 | *ZK1127.2* | 0.078 | NE |
| B0464.6 | *B0464.6* | 0.077 | NE |
| C01F6.8 | *icl-1* | 0.077 | NE |
| F14B4.1 | *F14B4.1* | 0.077 | NE |
| C28H8.8 | *C28H8.8* | 0.076 | NE |
| C32E8.4 | *C32E8.4* | 0.076 | NE |
| F07E5.7 | *F07E5.7* | 0.076 | NE |
| F38B2.1 | *ifa-1* | 0.076 | NE |
| ZC204.10 | *fbxb-16* | 0.076 | NE |
| ZK945.4 | *ZK945.4* | 0.076 | NE |
| C34F6.8 | *C34F6.8* | 0.075 | NE |
| T27C10.3 | *mop-25.3* | 0.075 | NE |
| Y39A3B.1 | *Y39A3B.1* | 0.074 | NE |
| Y76B12C.1 | *Y76B12C.1* | 0.074 | NE |
| K07H8.10 | *K07H8.10* | 0.074 | NE |
| F40F12.5 | *cyld-1* | 0.074 | NE |
| F59B2.2 | *F59B2.2* | 0.074 | NE |
| F41E6.5 | *F41E6.5* | 0.074 | NE |
| C50F7.10 | *C50F7.10* | 0.073 | NE |
| C04F12.3 | *ikb-1* | 0.073 | NE |
| F15A4.9 | *F15A4.9* | 0.073 | NE |
| F34D10.3 | *F34D10.3* | 0.073 | NE |
| F14B6.1 | *sdz-14* | 0.073 | NE |
| T10C6.8 | *T10C6.8* | 0.073 | NE |
| R148.3 | *R148.3* | 0.073 | NE |
| F49E8.7 | *F49E8.7* | 0.072 | NE |
| F29A7.6 | *F29A7.6* | 0.072 | NE |
| F13H8.1 | *F13H8.1* | 0.072 | NE |
| E02C12.5 | *gpa-3* | 0.071 | NE |
| M05D6.7 | *gbh-2* | 0.071 | NE |
| R10E4.9 | *R10E4.9* | 0.071 | NE |
| K03E6.6 | *pfn-3* | 0.07 | NE |
| W01G7.3 | *rpb-11* | 0.07 | NE |
| R12C12.7 | *R12C12.7* | 0.07 | NE |
| C52D10.8 | *skr-13* | 0.07 | NE |
| ZK354.4 | *msp-113* | 0.07 | NE |
| F43C11.1 | *F43C11.1* | 0.069 | NE |
| F35C11.3 | *F35C11.3* | 0.069 | NE |
| ZK632.5 | *ZK632.5* | 0.069 | NE |
| D1054.6 | *D1054.6* | 0.069 | NE |
| Y55B1AR.4 | *Y55B1AR.4* | 0.068 | NE |
| R07G3.2 | *lips-17* | 0.068 | NE |
| EEED8.7 | *rsp-4* | 0.068 | NE |
| C18E9.8 | *C18E9.8* | 0.067 | NE |
| E04F6.4 | *E04F6.4* | 0.067 | NE |
| F47B8.11 | *sss-2* | 0.067 | NE |
| F13D12.2 | *ldh-1* | 0.067 | NE |
| W09D6.5 | *W09D6.5* | 0.067 | NE |
| M04D8.3 | *ins-23* | 0.066 | NE |
| T01H3.5 | *T01H3.5* | 0.066 | NE |
| Y57A10A.32 | *Y57A10A.32* | 0.066 | NE |
| F37A4.7 | *rbf-1* | 0.066 | NE |
| F21D5.5 | *F21D5.5* | 0.066 | NE |
| Y51H4A.4 | *Y51H4A.4* | 0.065 | NE |
| K08D12.2 | *K08D12.2* | 0.065 | NE |
| F08B4.3 | *F08B4.3* | 0.064 | NE |
| C34D4.13 | *C34D4.13* | 0.064 | NE |
| Y47D3A.17 | *obr-1* | 0.064 | NE |
| C01F1.4 | *C01F1.4* | 0.063 | NE |
| T04H1.2 | *T04H1.2* | 0.063 | NE |
| F15A4.2 | *F15A4.2* | 0.063 | NE |
| M04B2.3 | *gfl-1* | 0.063 | NE |
| F46A9.4 | *skr-2* | 0.062 | NE |
| T24B8.1 | *rpl-32* | 0.062 | NE |
| Y49F6C.3 | *bath-9* | 0.062 | NE |
| F21H11.2 | *sax-2* | 0.062 | NE |
| M176.7 | *kin-16* | 0.062 | NE |
| R01H2.5 | *ger-1* | 0.061 | NE |
| B0205.8 | *B0205.8* | 0.061 | NE |
| F30A10.5 | *stl-1* | 0.061 | NE |
| Y57A10B.1 | *Y57A10B.1* | 0.061 | NE |
| T04C9.1 | *T04C9.1* | 0.06 | NE |
| F11G11.8 | *nspd-5* | 0.059 | NE |
| W01B6.4 | *W01B6.4* | 0.059 | NE |
| F37B1.6 | *gst-17* | 0.058 | NE |
| B0414.3 | *hil-5* | 0.057 | NE |
| T27F6.5 | *prs-2* | 0.057 | NE |
| C04G6.1 | *mpk-2* | 0.057 | NE |
| F56D1.6 | *cex-1* | 0.057 | NE |
| D1014.5 | *D1014.5* | 0.057 | NE |
| C48E7.1 | *C48E7.1* | 0.056 | NE |
| ZK1248.6 | *msp-64* | 0.056 | NE |
| H25P19.1 | *H25P19.1* | 0.056 | NE |
| F58A6.1 | *F58A6.1* | 0.056 | NE |
| W09B12.1 | *ace-1* | 0.056 | NE |
| C50E3.13 | *C50E3.13* | 0.055 | NE |
| ZC47.10 | *fbxa-34* | 0.055 | NE |
| F37A4.2 | *F37A4.2* | 0.055 | NE |
| VC5.3 | *npa-1* | 0.055 | NE |
| F59A1.4 | *str-89* | 0.055 | NE |
| ZK795.3 | *ZK795.3* | 0.055 | NE |
| Y39E4B.5 | *Y39E4B.5* | 0.055 | NE |
| Y46G5A.5 | *pisy-1* | 0.055 | NE |
| T08B2.12 | *T08B2.12* | 0.055 | NE |
| F01F1.10 | *eng-1* | 0.054 | NE |
| T27A1.1 | *T27A1.1* | 0.054 | NE |
| C17G10.1 | *C17G10.1* | 0.054 | NE |
| Y48A6B.1 | *Y48A6B.1* | 0.054 | NE |
| H36L18.2 | *H36L18.2* | 0.054 | NE |
| R11F4.1 | *R11F4.1* | 0.054 | NE |
| C29F9.10 | *fbxa-57* | 0.054 | NE |
| F11H8.1 | *rfl-1* | 0.054 | NE |
| B0285.1 | *B0285.1* | 0.054 | NE |
| T27E4.3 | *hsp-16.48* | 0.053 | NE |
| F26F12.4 | *F26F12.4* | 0.053 | NE |
| C15F1.5 | *C15F1.5* | 0.053 | NE |
| R166.5 | *mnk-1* | 0.053 | NE |
| F32H2.2 | *mdt-31* | 0.052 | NE |
| C23G10.11 | *C23G10.11* | 0.052 | NE |
| Y39C12A.9 | *Y39C12A.9* | 0.052 | NE |
| F55A4.1 | *F55A4.1* | 0.052 | NE |
| D2085.5 | *D2085.5* | 0.051 | NE |
| Y39A3B.5 | *ckr-2* | 0.051 | NE |
| F46C8.5 | *ceh-14* | 0.051 | NE |
| F10B5.5 | *pch-2* | 0.051 | NE |
| Y4C6B.5 | *Y4C6B.5* | 0.05 | NE |
| F22B5.10 | *F22B5.10* | 0.05 | NE |
| F22E5.6 | *F22E5.6* | 0.05 | NE |
| C16C8.4 | *C16C8.4* | 0.05 | NE |
| R11D1.1 | *R11D1.1* | 0.05 | NE |
| T28C6.5 | *T28C6.5* | 0.05 | NE |
| C32B5.10 | *C32B5.10* | 0.05 | NE |
| JC8.7 | *JC8.7* | 0.05 | NE |
| F23F1.7 | *F23F1.7* | 0.05 | NE |
| Y51B9A.4 | *Y51B9A.4* | 0.05 | NE |
| K05G3.3 | *cah-3* | 0.049 | NE |
| D2096.10 | *D2096.10* | 0.049 | NE |
| R07E5.6 | *R07E5.6* | 0.049 | NE |
| T07D3.7 | *alg-2* | 0.049 | NE |
| R06F6.1 | *cdl-1* | 0.049 | NE |
| C02F5.6 | *C02F5.6* | 0.049 | NE |
| ZK430.2 | *tag-231* | 0.048 | NE |
| F57F5.5 | *pkc-1* | 0.048 | NE |
| E02C12.8 | *E02C12.8* | 0.048 | NE |
| ZK1248.11 | *ZK1248.11* | 0.047 | NE |
| Y27F2A.6 | *Y27F2A.6* | 0.047 | NE |
| Y47D7A.1 | *skr-7* | 0.047 | NE |
| F45D11.6 | *F45D11.6* | 0.046 | NE |
| F14D7.2 | *F14D7.2* | 0.046 | NE |
| C13F10.7 | *C13F10.7* | 0.046 | NE |
| F39B2.6 | *rps-26* | 0.045 | NE |
| B0511.8 | *tag-264* | 0.045 | NE |
| F10G8.3 | *npp-17* | 0.044 | NE |
| B0280.5 | *B0280.5* | 0.044 | NE |
| F56C9.8 | *F56C9.8* | 0.044 | NE |
| C34C12.3 | *pph-6* | 0.044 | NE |
| F35H8.5 | *exc-7* | 0.044 | NE |
| C09H10.6 | *nasp-1* | 0.044 | NE |
| B0035.11 | *B0035.11* | 0.044 | NE |
| K12H4.3 | *K12H4.3* | 0.044 | NE |
| D2085.6 | *D2085.6* | 0.044 | NE |
| K10G9.1 | *K10G9.1* | 0.044 | NE |
| Y113G7A.11 | *ssu-1* | 0.043 | NE |
| F13D12.3 | *F13D12.3* | 0.043 | NE |
| C49C3.7 | *C49C3.7* | 0.043 | NE |
| F45E4.9 | *hmg-5* | 0.042 | NE |
| C10G11.10 | *C10G11.10* | 0.042 | NE |
| ZK1251.2 | *ins-7* | 0.042 | NE |
| B0432.3 | *B0432.3* | 0.042 | NE |
| C01G12.7 | *C01G12.7* | 0.042 | NE |
| C08B11.4 | *nrf-6* | 0.042 | NE |
| Y54E5B.3 | *let-49* | 0.041 | NE |
| C32B5.6 | *C32B5.6* | 0.041 | NE |
| M01F1.7 | *M01F1.7* | 0.04 | NE |
| C05B5.4 | *C05B5.4* | 0.04 | NE |
| T14A8.1 | *ric-3* | 0.04 | NE |
| W03G9.2 | *W03G9.2* | 0.039 | NE |
| T05A7.3 | *T05A7.3* | 0.038 | NE |
| F25B5.2 | *F25B5.2* | 0.038 | NE |
| Y52E8A.3 | *Y52E8A.3* | 0.038 | NE |
| W09H1.5 | *W09H1.5* | 0.038 | NE |
| R74.4 | *dnj-16* | 0.038 | NE |
| W03G11.1 | *col-181* | 0.037 | NE |
| C08E3.6 | *fbxa-163* | 0.036 | NE |
| F10G7.11 | *ttr-41* | 0.036 | NE |
| F15H10.1 | *col-12* | 0.036 | NE |
| Y39A3A.1 | *Y39A3A.1* | 0.036 | NE |
| T13F2.8 | *cav-1* | 0.036 | NE |
| F57C9.5 | *htp-3* | 0.036 | NE |
| D1081.2 | *unc-120* | 0.035 | NE |
| Y105E8B.1 | *lev-11* | 0.035 | NE |
| T12B5.6 | *fbxa-55* | 0.035 | NE |
| C34E10.2 | *gop-2* | 0.035 | NE |
| Y66D12A.2 | *Y66D12A.2* | 0.034 | NE |
| T05H10.4 | *T05H10.4* | 0.034 | NE |
| F55A11.3 | *hrd-1* | 0.034 | NE |
| F54C9.9 | *F54C9.9* | 0.033 | NE |
| Y57A10B.6 | *Y57A10B.6* | 0.033 | NE |
| R08D7.1 | *R08D7.1* | 0.033 | NE |
| F10D11.1 | *sod-2* | 0.033 | NE |
| F45C12.13 | *fbxb-48* | 0.033 | NE |
| F12A10.4 | *F12A10.4* | 0.033 | NE |
| Y48E1B.12 | *csc-1* | 0.032 | NE |
| F42A6.9 | *elks-1* | 0.032 | NE |
| R09E10.8 | *R09E10.8* | 0.031 | NE |
| F46F11.6 | *F46F11.6* | 0.031 | NE |
| F19B10.1 | *F19B10.1* | 0.031 | NE |
| C06E4.2 | *C06E4.2* | 0.031 | NE |
| F40F8.8 | *F40F8.8* | 0.031 | NE |
| T25B6.2 | *T25B6.2* | 0.031 | NE |
| F55F8.5 | *tag-345* | 0.03 | NE |
| T07G12.6 | *zim-1* | 0.03 | NE |
| T05H10.6 | *T05H10.6* | 0.029 | NE |
| K09A9.3 | *ent-2* | 0.029 | NE |
| M01E11.6 | *klp-15* | 0.029 | NE |
| ZK673.1 | *ZK673.1* | 0.028 | NE |
| M02B7.5 | *M02B7.5* | 0.028 | NE |
| F42F12.4 | *F42F12.4* | 0.028 | NE |
| ZK637.14 | *ZK637.14* | 0.028 | NE |
| F40G9.6 | *F40G9.6* | 0.027 | NE |
| C23H3.2 | *C23H3.2* | 0.027 | NE |
| C05C8.1 | *C05C8.1* | 0.027 | NE |
| W02A2.8 | *W02A2.8* | 0.027 | NE |
| T20H4.3 | *prs-1* | 0.027 | NE |
| F49B2.5 | *src-2* | 0.027 | NE |
| F43G9.10 | *F43G9.10* | 0.026 | NE |
| F38C2.1 | *F38C2.1* | 0.026 | NE |
| Y57G7A.1 | *Y57G7A.1* | 0.025 | NE |
| E02H9.8 | *nhr-121* | 0.025 | NE |
| K09H9.6 | *lpd-6* | 0.024 | NE |
| F35C5.11 | *F35C5.11* | 0.023 | NE |
| F08G2.4 | *F08G2.4* | 0.023 | NE |
| F59B10.4 | *F59B10.4* | 0.023 | NE |
| F26B1.2 | *F26B1.2* | 0.022 | NE |
| F57B10.4 | *F57B10.4* | 0.022 | NE |
| F35C8.3 | *jkk-1* | 0.022 | NE |
| F14D2.2 | *F14D2.2* | 0.022 | NE |
| D2062.4 | *D2062.4* | 0.022 | NE |
| T16A9.4 | *T16A9.4* | 0.022 | NE |
| C27B7.1 | *spr-2* | 0.022 | NE |
| ZC15.3 | *ZC15.3* | 0.022 | NE |
| F58G11.3 | *F58G11.3* | 0.022 | NE |
| Y32F6B.3 | *crp-1* | 0.022 | NE |
| Y53F4B.32 | *gst-29* | 0.022 | NE |
| ZK1010.9 | *snf-7* | 0.022 | NE |
| F53C3.2 | *fbxb-103* | 0.021 | NE |
| ZK1290.3 | *rol-8* | 0.02 | NE |
| F45E4.2 | *plp-1* | 0.02 | NE |
| ZK945.6 | *ZK945.6* | 0.02 | NE |
| B0273.2 | *B0273.2* | 0.02 | NE |
| T15H9.4 | *T15H9.4* | 0.02 | NE |
| Y75B8A.33 | *Y75B8A.33* | 0.02 | NE |
| C06E1.7 | *C06E1.7* | 0.02 | NE |
| Y47D3B.11 | *Y47D3B.11* | 0.02 | NE |
| C32B5.4 | *C32B5.4* | 0.019 | NE |
| C09E8.1 | *C09E8.1* | 0.019 | NE |
| W01B6.7 | *col-2* | 0.019 | NE |
| C48D5.2 | *ptp-1* | 0.019 | NE |
| F54A3.5 | *F54A3.5* | 0.018 | NE |
| C49D10.6 | *nhr-75* | 0.018 | NE |
| E01G4.3 | *E01G4.3* | 0.018 | NE |
| F09F7.6 | *F09F7.6* | 0.018 | NE |
| C29F5.2 | *sdz-3* | 0.018 | NE |
| W09G12.9 | *W09G12.9* | 0.018 | NE |
| W07E11.3 | *flp-2* | 0.018 | NE |
| C14A4.6 | *C14A4.6* | 0.017 | NE |
| R06B9.5 | *R06B9.5* | 0.017 | NE |
| F56C9.7 | *F56C9.7* | 0.017 | NE |
| C27B7.5 | *C27B7.5* | 0.017 | NE |
| K07A12.6 | *hot-5* | 0.016 | NE |
| Y40D12A.2 | *Y40D12A.2* | 0.016 | NE |
| F56F3.2 | *ndg-4* | 0.016 | NE |
| C02B10.2 | *C02B10.2* | 0.016 | NE |
| H13N06.3 | *gob-1* | 0.016 | NE |
| Y17G7B.15 | *cnt-1* | 0.016 | NE |
| Y37E11AL.7 | *map-1* | 0.016 | NE |
| T21G5.3 | *glh-1* | 0.016 | NE |
| C39H7.1 | *C39H7.1* | 0.016 | NE |
| C01H6.5 | *nhr-23* | 0.016 | NE |
| F48E3.1 | *gly-12* | 0.016 | NE |
| Y106G6H.14 | *Y106G6H.14* | 0.015 | NE |
| ZK470.5 | *nck-1* | 0.015 | NE |
| F07E5.2 | *fbxb-35* | 0.015 | NE |
| F46E10.2 | *F46E10.2* | 0.015 | NE |
| F54H5.4 | *mua-1* | 0.015 | NE |
| ZC239.9 | *sri-48* | 0.014 | NE |
| F44B9.9 | *F44B9.9* | 0.013 | NE |
| C54C6.1 | *rpl-37* | 0.013 | NE |
| ZK1098.3 | *ZK1098.3* | 0.013 | NE |
| T10B11.2 | *T10B11.2* | 0.013 | NE |
| F58H1.7 | *F58H1.7* | 0.013 | NE |
| F33D11.11 | *vpr-1* | 0.013 | NE |
| Y51H7C.6 | *cogc-4* | 0.013 | NE |
| ZK75.2 | *ins-2* | 0.012 | NE |
| F16B4.4 | *F16B4.4* | 0.012 | NE |
| T16D1.2 | *pho-4* | 0.012 | NE |
| F35H8.7 | *wee-1.1* | 0.012 | NE |
| F59A6.3 | *F59A6.3* | 0.011 | NE |
| F41A4.1 | *F41A4.1* | 0.011 | NE |
| F28C6.8 | *F28C6.8* | 0.011 | NE |
| Y67A10A.6 | *ssu-2* | 0.01 | NE |
| C07H6.2 | *C07H6.2* | 0.01 | NE |
| VW02B12L.4 | *adbp-1* | 0.01 | NE |
| Y57A10C.1 | *Y57A10C.1* | 0.01 | NE |
| R08C7.7 | *str-185* | 0.01 | NE |
| F32B6.3 | *F32B6.3* | 0.01 | NE |
| ZK418.5 | *ZK418.5* | 0.01 | NE |
| ZK354.2 | *ZK354.2* | 0.009 | NE |
| F19H8.2 | *F19H8.2* | 0.009 | NE |
| H27M09.1 | *H27M09.1* | 0.009 | NE |
| Y57A10C.7 | *dct-12* | 0.009 | NE |
| F32A11.6 | *moe-3* | 0.008 | NE |
| Y47D3A.26 | *smc-3* | 0.007 | NE |
| K12H4.5 | *K12H4.5* | 0.007 | NE |
| F12F6.5 | *srgp-1* | 0.007 | NE |
| F52H3.2 | *F52H3.2* | 0.007 | NE |
| W02A2.7 | *mex-5* | 0.007 | NE |
| C34B2.8 | *C34B2.8* | 0.007 | NE |
| Y39A3A.2 | *Y39A3A.2* | 0.006 | NE |
| F57C9.2 | *clec-90* | 0.006 | NE |
| C16C10.3 | *C16C10.3* | 0.006 | NE |
| W03C9.2 | *W03C9.2* | 0.006 | NE |
| ZK524.1 | *spe-4* | 0.006 | NE |
| K12H6.7 | *K12H6.7* | 0.006 | NE |
| F18A1.8 | *F18A1.8* | 0.005 | NE |
| F43D9.1 | *F43D9.1* | 0.005 | NE |
| C34B2.7 | *sdha-2* | 0.004 | NE |
| C06A1.6 | *C06A1.6* | 0.004 | NE |
| R10A10.2 | *rbx-2* | 0.004 | NE |
| Y56A3A.13 | *nft-1* | 0.004 | NE |
| T14B4.4 | *tsp-10* | 0.004 | NE |
| C37A5.7 | *C37A5.7* | 0.004 | NE |
| Y47G6A.25 | *Y47G6A.25* | 0.004 | NE |
| F15D4.2 | *F15D4.2* | 0.003 | NE |
| F33H2.1 | *dog-1* | 0.003 | NE |
| ZK742.2 | *ZK742.2* | 0.003 | NE |
| EEED8.11 | *clec-141* | 0.002 | NE |
| C09G5.4 | *col-39* | 0.002 | NE |
| VW06B3R.1 | *ucr-2.1* | 0.002 | NE |
| K08E3.7 | *pdr-1* | 0.001 | NE |
| F54A5.1 | *F54A5.1* | 0.001 | NE |
| C02C6.3 | *C02C6.3* | 0.001 | NE |
| W06D4.1 | *hgo-1* | 0 | NE |
| T09B4.2 | *T09B4.2* | 0 | NE |
| R74.7 | *R74.7* | 0 | NE |
| K07A1.8 | *ile-1* | 0 | NE |
| Y45F10B.3 | *Y45F10B.3* | -0.001 | NE |
| Y39A1A.16 | *Y39A1A.16* | -0.001 | NE |
| F44F4.5 | *sra-10* | -0.002 | NE |
| C26C6.5 | *dcp-66* | -0.002 | NE |
| Y105C5B.20 | *Y105C5B.20* | -0.002 | NE |
| K09D9.2 | *cyp-35A3* | -0.003 | NE |
| C37A2.3 | *C37A2.3* | -0.003 | NE |
| T12C9.6 | *nhr-16* | -0.003 | NE |
| C44E12.3 | *twk-17* | -0.003 | NE |
| C31H5.6 | *C31H5.6* | -0.003 | NE |
| D2062.7 | *D2062.7* | -0.003 | NE |
| F52A8.2 | *gpb-2* | -0.003 | NE |
| ZK945.8 | *ZK945.8* | -0.003 | NE |
| F33H1.3 | *F33H1.3* | -0.003 | NE |
| ZC434.8 | *ZC434.8* | -0.003 | NE |
| Y110A2AM.1 | *Y110A2AM.1* | -0.004 | NE |
| K02D10.1 | *K02D10.1* | -0.004 | NE |
| C33H5.12 | *rsp-6* | -0.004 | NE |
| C36C9.1 | *C36C9.1* | -0.004 | NE |
| T12B5.3 | *fbxa-10* | -0.004 | NE |
| F44E2.3 | *F44E2.3* | -0.004 | NE |
| C14B9.6 | *gei-8* | -0.004 | NE |
| T28F4.5 | *T28F4.5* | -0.005 | NE |
| T24F1.5 | *T24F1.5* | -0.005 | NE |
| Y57A10A.25 | *Y57A10A.25* | -0.005 | NE |
| ZK1307.2 | *ZK1307.2* | -0.005 | NE |
| T19B10.6 | *T19B10.6* | -0.005 | NE |
| F14D2.4 | *bath-29* | -0.005 | NE |
| ZK637.3 | *tag-256* | -0.006 | NE |
| T26E3.3 | *par-6* | -0.006 | NE |
| F45H7.2 | *gei-1* | -0.007 | NE |
| T12E12.1 | *T12E12.1* | -0.007 | NE |
| F28C10.3 | *F28C10.3* | -0.007 | NE |
| E01A2.8 | *E01A2.8* | -0.007 | NE |
| F25B4.1 | *F25B4.1* | -0.008 | NE |
| ZK1320.6 | *arc-1* | -0.008 | NE |
| F30A10.3 | *F30A10.3* | -0.008 | NE |
| T03F7.7 | *T03F7.7* | -0.008 | NE |
| B0361.10 | *B0361.10* | -0.008 | NE |
| K07A12.5 | *K07A12.5* | -0.008 | NE |
| F01F1.6 | *alh-9* | -0.009 | NE |
| C49A1.4 | *eya-1* | -0.009 | NE |
| T23G7.5 | *pir-1* | -0.01 | NE |
| R05G6.9 | *R05G6.9* | -0.01 | NE |
| F54C4.2 | *spt-4* | -0.01 | NE |
| R10E12.2 | *R10E12.2* | -0.01 | NE |
| F26F4.10 | *rrt-1* | -0.01 | NE |
| W09G10.1 | *col-72* | -0.01 | NE |
| ZK770.3 | *inx-12* | -0.01 | NE |
| F55G11.5 | *dod-22* | -0.011 | NE |
| M03F8.2 | *pst-1* | -0.011 | NE |
| F42G2.6 | *F42G2.6* | -0.011 | NE |
| F47F6.3 | *F47F6.3* | -0.011 | NE |
| R74.5 | *asd-1* | -0.012 | NE |
| C29F5.7 | *glb-10* | -0.012 | NE |
| Y75B8A.30 | *pph-4.1* | -0.012 | NE |
| C49C3.1 | *snf-9* | -0.012 | NE |
| ZK418.7 | *ZK418.7* | -0.012 | NE |
| ZK1058.1 | *mmcm-1* | -0.012 | NE |
| H16D19.1 | *clec-13* | -0.013 | NE |
| F36D1.5 | *F36D1.5* | -0.013 | NE |
| W06E11.2 | *tag-267* | -0.013 | NE |
| E01A2.7 | *E01A2.7* | -0.013 | NE |
| F45B8.4 | *pag-3* | -0.013 | NE |
| Y49E10.2 | *glrx-5* | -0.013 | NE |
| Y73B6A.3 | *Y73B6A.3* | -0.014 | NE |
| B0454.1 | *lin-8* | -0.015 | NE |
| C45E5.1 | *C45E5.1* | -0.015 | NE |
| C01B12.3 | *C01B12.3* | -0.015 | NE |
| K07E8.7 | *K07E8.7* | -0.015 | NE |
| C34D4.11 | *grsp-3* | -0.015 | NE |
| Y46H3A.3 | *hsp-16.2* | -0.015 | NE |
| F08F3.3 | *rhr-1* | -0.016 | NE |
| F14B6.2 | *F14B6.2* | -0.016 | NE |
| W09C2.1 | *elt-1* | -0.017 | NE |
| T08E11.4 | *math-41* | -0.017 | NE |
| Y111B2A.15 | *tpst-1* | -0.017 | NE |
| E02H1.6 | *E02H1.6* | -0.017 | NE |
| K07H8.9 | *K07H8.9* | -0.017 | NE |
| C34E11.1 | *rsd-3* | -0.017 | NE |
| R10D12.14 | *R10D12.14* | -0.017 | NE |
| C16C10.4 | *C16C10.4* | -0.017 | NE |
| W01G7.1 | *daf-5* | -0.017 | NE |
| C16B8.1 | *lin-18* | -0.018 | NE |
| F52A8.5 | *F52A8.5* | -0.018 | NE |
| Y16B4A.1 | *unc-3* | -0.018 | NE |
| T26A8.4 | *T26A8.4* | -0.019 | NE |
| ZK337.5 | *mtd-1* | -0.019 | NE |
| Y43F8C.8 | *Y43F8C.8* | -0.019 | NE |
| F21H12.5 | *fbf-2* | -0.019 | NE |
| B0238.11 | *B0238.11* | -0.019 | NE |
| F30H5.2 | *nlp-32* | -0.02 | NE |
| F52C12.1 | *F52C12.1* | -0.021 | NE |
| Y45G12C.7 | *srd-73* | -0.021 | NE |
| Y37E11B.1 | *Y37E11B.1* | -0.021 | NE |
| R08D7.2 | *R08D7.2* | -0.021 | NE |
| Y17G7B.3 | *Y17G7B.3* | -0.021 | NE |
| C50F7.4 | *C50F7.4* | -0.021 | NE |
| ZK154.7 | *adm-4* | -0.021 | NE |
| Y49F6C.8 | *Y49F6C.8* | -0.021 | NE |
| ZK287.8 | *her-1* | -0.021 | NE |
| F56F11.3 | *klf-1* | -0.022 | NE |
| ZC395.2 | *clk-1* | -0.022 | NE |
| K02F3.4 | *zip-2* | -0.023 | NE |
| C24A3.4 | *C24A3.4* | -0.023 | NE |
| F32A7.3 | *eva-1* | -0.023 | NE |
| R144.6 | *R144.6* | -0.024 | NE |
| T12D8.2 | *drr-2* | -0.024 | NE |
| R10E11.4 | *sqv-3* | -0.024 | NE |
| Y64G10A.1 | *Y64G10A.1* | -0.024 | NE |
| F59F5.2 | *F59F5.2* | -0.024 | NE |
| T04G9.5 | *trap-2* | -0.024 | NE |
| T25B9.9 | *T25B9.9* | -0.026 | NE |
| Y39E4B.1 | *abce-1* | -0.026 | NE |
| Y53F4B.2 | *elo-9* | -0.027 | NE |
| Y48C3A.3 | *Y48C3A.3* | -0.027 | NE |
| C07G1.2 | *C07G1.2* | -0.027 | NE |
| Y77E11A.9 | *clec-171* | -0.027 | NE |
| C28C12.2 | *C28C12.2* | -0.028 | NE |
| F49E8.3 | *pam-1* | -0.028 | NE |
| W01F3.2 | *W01F3.2* | -0.028 | NE |
| K04B12.3 | *K04B12.3* | -0.028 | NE |
| M28.8 | *M28.8* | -0.028 | NE |
| F15A4.10 | *F15A4.10* | -0.029 | NE |
| F54D8.3 | *alh-1* | -0.029 | NE |
| C47B2.1 | *fbxa-140* | -0.029 | NE |
| C48B6.3 | *C48B6.3* | -0.029 | NE |
| F56A3.4 | *spd-5* | -0.029 | NE |
| C16C4.4 | *math-14* | -0.029 | NE |
| C08B6.8 | *C08B6.8* | -0.029 | NE |
| F47D12.1 | *gar-2* | -0.03 | NE |
| F40F4.1 | *fbxb-71* | -0.03 | NE |
| F59C12.3 | *F59C12.3* | -0.03 | NE |
| T09E8.2 | *him-17* | -0.03 | NE |
| H14E04.5 | *cic-1* | -0.03 | NE |
| C07A9.2 | *C07A9.2* | -0.03 | NE |
| C49H3.6 | *C49H3.6* | -0.03 | NE |
| F44E2.8 | *F44E2.8* | -0.03 | NE |
| C15F1.2 | *C15F1.2* | -0.03 | NE |
| R05D3.2 | *R05D3.2* | -0.03 | NE |
| C25A11.2 | *C25A11.2* | -0.03 | NE |
| W03A5.5 | *W03A5.5* | -0.031 | NE |
| C06E7.6 | *spe-27* | -0.031 | NE |
| F08G5.1 | *F08G5.1* | -0.031 | NE |
| Y51H7C.3 | *Y51H7C.3* | -0.031 | NE |
| ZC204.9 | *fbxb-20* | -0.031 | NE |
| Y46G5A.18 | *Y46G5A.18* | -0.032 | NE |
| C54E4.1 | *C54E4.1* | -0.032 | NE |
| Y22D7AL.10 | *Y22D7AL.10* | -0.033 | NE |
| B0303.15 | *B0303.15* | -0.033 | NE |
| C04F12.9 | *rnh-1.3* | -0.034 | NE |
| Y113G7B.16 | *Y113G7B.16* | -0.034 | NE |
| ZK643.5 | *ZK643.5* | -0.035 | NE |
| H02I12.8 | *cyp-31A2* | -0.035 | NE |
| F16H11.3 | *F16H11.3* | -0.035 | NE |
| M03D4.1 | *zen-4* | -0.036 | NE |
| Y53F4B.35 | *gst-31* | -0.036 | NE |
| M7.9 | *M7.9* | -0.036 | NE |
| T22C1.5 | *T22C1.5* | -0.036 | NE |
| PDB1.1 | *PDB1.1* | -0.036 | NE |
| F34D6.3 | *sup-9* | -0.036 | NE |
| VF39H2L.1 | *VF39H2L.1* | -0.036 | NE |
| Y54E2A.5 | *Y54E2A.5* | -0.036 | NE |
| W03D2.5 | *wrt-5* | -0.036 | NE |
| W10D5.1 | *mef-2* | -0.037 | NE |
| C28C12.5 | *spp-8* | -0.037 | NE |
| W08E3.3 | *tag-210* | -0.037 | NE |
| C44E4.3 | *C44E4.3* | -0.037 | NE |
| R04F11.2 | *R04F11.2* | -0.037 | NE |
| Y87G2A.7 | *Y87G2A.7* | -0.038 | NE |
| K07C11.2 | *air-1* | -0.038 | NE |
| T05D4.1 | *T05D4.1* | -0.038 | NE |
| ZK1248.13 | *ZK1248.13* | -0.038 | NE |
| T15H9.6 | *T15H9.6* | -0.038 | NE |
| ZK909.6 | *ZK909.6* | -0.038 | NE |
| C04F12.8 | *C04F12.8* | -0.038 | NE |
| C07D10.3 | *sre-3* | -0.038 | NE |
| C16C8.19 | *C16C8.19* | -0.039 | NE |
| C10H11.6 | *ugt-26* | -0.039 | NE |
| ZK381.4 | *pgl-1* | -0.039 | NE |
| D2092.2 | *ppfr-2* | -0.039 | NE |
| C06A12.5 | *lact-6* | -0.04 | NE |
| ZK1290.1 | *ZK1290.1* | -0.04 | NE |
| C02E7.7 | *C02E7.7* | -0.041 | NE |
| ZK666.6 | *clec-60* | -0.041 | NE |
| Y47G6A.2 | *inx-22* | -0.041 | NE |
| W10G11.11 | *clec-134* | -0.041 | NE |
| F09F7.8 | *nspb-12* | -0.041 | NE |
| Y54E2A.7 | *Y54E2A.7* | -0.042 | NE |
| Y34B4A.9 | *Y34B4A.9* | -0.042 | NE |
| R09D1.10 | *R09D1.10* | -0.042 | NE |
| M03A8.4 | *gei-15* | -0.042 | NE |
| Y57A10A.5 | *Y57A10A.5* | -0.042 | NE |
| ZK1025.2 | *ZK1025.2* | -0.042 | NE |
| T25E4.1 | *T25E4.1* | -0.042 | NE |
| F36D4.2 | *F36D4.2* | -0.042 | NE |
| F42A8.3 | *F42A8.3* | -0.042 | NE |
| F02E9.3 | *F02E9.3* | -0.043 | NE |
| F37H8.3 | *F37H8.3* | -0.043 | NE |
| C31A11.5 | *C31A11.5* | -0.043 | NE |
| C38D4.8 | *arl-6* | -0.043 | NE |
| F55C5.4 | *F55C5.4* | -0.043 | NE |
| T02E1.4 | *T02E1.4* | -0.043 | NE |
| F46G10.6 | *mxl-3* | -0.043 | NE |
| T05E11.3 | *T05E11.3* | -0.044 | NE |
| ZK593.7 | *lsm-7* | -0.044 | NE |
| M04D8.7 | *M04D8.7* | -0.045 | NE |
| C02E7.6 | *C02E7.6* | -0.045 | NE |
| B0280.7 | *B0280.7* | -0.045 | NE |
| W03B1.2 | *W03B1.2* | -0.045 | NE |
| F17E9.4 | *F17E9.4* | -0.046 | NE |
| F17E9.7 | *lgc-5* | -0.046 | NE |
| Y17G7B.12 | *Y17G7B.12* | -0.047 | NE |
| Y43F4B.4 | *npp-18* | -0.047 | NE |
| R05A10.4 | *R05A10.4* | -0.048 | NE |
| Y92C3B.1 | *kbp-4* | -0.048 | NE |
| CC4.3 | *smu-1* | -0.049 | NE |
| ZK637.4 | *ZK637.4* | -0.049 | NE |
| C55B7.3 | *C55B7.3* | -0.049 | NE |
| C40D2.2 | *math-20* | -0.05 | NE |
| Y46D2A.1 | *Y46D2A.1* | -0.05 | NE |
| K09F6.8 | *K09F6.8* | -0.05 | NE |
| F40F12.2 | *F40F12.2* | -0.05 | NE |
| ZK829.9 | *ZK829.9* | -0.05 | NE |
| T12B3.1 | *T12B3.1* | -0.051 | NE |
| Y110A2AL.5 | *Y110A2AL.5* | -0.051 | NE |
| K01A2.3 | *K01A2.3* | -0.051 | NE |
| F44B9.6 | *lin-36* | -0.051 | NE |
| F08G2.5 | *F08G2.5* | -0.051 | NE |
| ZK938.4 | *ZK938.4* | -0.051 | NE |
| C54E10.6 | *C54E10.6* | -0.051 | NE |
| Y24D9A.7 | *Y24D9A.7* | -0.051 | NE |
| Y110A7A.12 | *tag-300* | -0.051 | NE |
| F29C12.3 | *F29C12.3* | -0.052 | NE |
| F59C12.2 | *ser-1* | -0.052 | NE |
| T12F5.2 | *T12F5.2* | -0.052 | NE |
| K05F1.1 | *K05F1.1* | -0.052 | NE |
| F52E1.10 | *vha-18* | -0.052 | NE |
| Y73B6A.4 | *smg-7* | -0.052 | NE |
| Y57A10A.10 | *Y57A10A.10* | -0.052 | NE |
| F09G8.3 | *F09G8.3* | -0.053 | NE |
| F37B1.2 | *gst-12* | -0.053 | NE |
| F58F12.3 | *F58F12.3* | -0.053 | NE |
| Y37H9A.6 | *ndx-4* | -0.053 | NE |
| W05F2.3 | *W05F2.3* | -0.054 | NE |
| C26G2.1 | *syg-2* | -0.054 | NE |
| C09F5.2 | *orai-1* | -0.054 | NE |
| R04A9.4 | *ife-2* | -0.055 | NE |
| Y71F9AL.2 | *Y71F9AL.2* | -0.055 | NE |
| Y17G7B.8 | *Y17G7B.8* | -0.055 | NE |
| Y94H6A.2 | *Y94H6A.2* | -0.055 | NE |
| B0334.4 | *B0334.4* | -0.055 | NE |
| T28A8.4 | *T28A8.4* | -0.056 | NE |
| Y57G7A.2 | *Y57G7A.2* | -0.056 | NE |
| Y55B1BR.2 | *Y55B1BR.2* | -0.056 | NE |
| D1022.8 | *cah-2* | -0.056 | NE |
| Y45F10D.1 | *Y45F10D.1* | -0.057 | NE |
| C05G5.4 | *C05G5.4* | -0.057 | NE |
| T20G5.9 | *T20G5.9* | -0.057 | NE |
| C08G5.4 | *snt-6* | -0.057 | NE |
| K07A1.11 | *rba-1* | -0.057 | NE |
| C08C3.1 | *egl-5* | -0.057 | NE |
| C14A4.12 | *C14A4.12* | -0.058 | NE |
| M01H9.3 | *M01H9.3* | -0.058 | NE |
| ZK617.2 | *lips-6* | -0.058 | NE |
| K10E9.2 | *K10E9.2* | -0.058 | NE |
| F57B10.3 | *F57B10.3* | -0.058 | NE |
| C50D2.5 | *C50D2.5* | -0.058 | NE |
| ZC204.12 | *ZC204.12* | -0.059 | NE |
| B0454.5 | *B0454.5* | -0.059 | NE |
| C15H7.3 | *C15H7.3* | -0.059 | NE |
| F49D11.8 | *cpn-4* | -0.059 | NE |
| T23F4.2 | *T23F4.2* | -0.059 | NE |
| W02A11.4 | *uba-2* | -0.059 | NE |
| T05G5.3 | *cdk-1* | -0.059 | NE |
| F42G9.6 | *F42G9.6* | -0.059 | NE |
| W06D4.4 | *W06D4.4* | -0.059 | NE |
| F21A9.1 | *F21A9.1* | -0.059 | NE |
| K02F6.8 | *K02F6.8* | -0.059 | NE |
| C04F1.3 | *lim-7* | -0.06 | NE |
| F35D11.1 | *F35D11.1* | -0.06 | NE |
| F10D2.11 | *ugt-41* | -0.06 | NE |
| ZK418.1 | *nhr-9* | -0.061 | NE |
| B0545.3 | *scl-23* | -0.061 | NE |
| K02E7.10 | *K02E7.10* | -0.061 | NE |
| B0280.3 | *B0280.3* | -0.061 | NE |
| W01C9.5 | *glb-27* | -0.062 | NE |
| Y62H9A.6 | *Y62H9A.6* | -0.062 | NE |
| Y51B9A.9 | *Y51B9A.9* | -0.062 | NE |
| Y54G2A.18 | *Y54G2A.18* | -0.063 | NE |
| C09B8.6 | *hsp-25* | -0.063 | NE |
| R11A5.2 | *nud-2* | -0.063 | NE |
| F08A8.5 | *F08A8.5* | -0.063 | NE |
| C46F11.2 | *C46F11.2* | -0.063 | NE |
| D2013.5 | *eat-3* | -0.063 | NE |
| F45E12.1 | *scpl-2* | -0.063 | NE |
| T23G4.4 | *T23G4.4* | -0.064 | NE |
| C09H6.3 | *mau-2* | -0.064 | NE |
| T02H6.9 | *T02H6.9* | -0.065 | NE |
| C41C4.3 | *C41C4.3* | -0.065 | NE |
| Y17G7B.14 | *Y17G7B.14* | -0.065 | NE |
| K05F1.3 | *K05F1.3* | -0.065 | NE |
| C43E11.4 | *tufm-2* | -0.066 | NE |
| R11G10.1 | *R11G10.1* | -0.066 | NE |
| Y46G5A.14 | *Y46G5A.14* | -0.066 | NE |
| C50C3.8 | *bath-42* | -0.066 | NE |
| T24F1.1 | *raga-1* | -0.066 | NE |
| K08D10.8 | *scrm-5* | -0.066 | NE |
| Y40H4A.1 | *gar-3* | -0.066 | NE |
| T21B4.2 | *col-85* | -0.066 | NE |
| Y48E1C.2 | *Y48E1C.2* | -0.066 | NE |
| T22E7.1 | *T22E7.1* | -0.066 | NE |
| Y55F3BR.1 | *Y55F3BR.1* | -0.067 | NE |
| Y106G6G.1 | *Y106G6G.1* | -0.067 | NE |
| T27E9.7 | *abcf-2* | -0.067 | NE |
| C08F1.3 | *fbxb-13* | -0.067 | NE |
| ZK520.2 | *sid-2* | -0.067 | NE |
| C43D7.8 | *C43D7.8* | -0.067 | NE |
| CC8.2 | *CC8.2* | -0.067 | NE |
| R52.3 | *math-35* | -0.067 | NE |
| C45G9.7 | *C45G9.7* | -0.067 | NE |
| R53.6 | *R53.6* | -0.067 | NE |
| C23H3.3 | *C23H3.3* | -0.068 | NE |
| ZK596.1 | *ZK596.1* | -0.068 | NE |
| R05G6.4 | *R05G6.4* | -0.068 | NE |
| D2062.5 | *D2062.5* | -0.068 | NE |
| B0041.6 | *ptps-1* | -0.069 | NE |
| R12C12.9 | *R12C12.9* | -0.069 | NE |
| R08D7.6 | *pde-2* | -0.069 | NE |
| F52H3.1 | *let-268* | -0.069 | NE |
| ZK1225.6 | *ssp-31* | -0.069 | NE |
| F42A10.6 | *F42A10.6* | -0.069 | NE |
| F20H11.6 | *F20H11.6* | -0.069 | NE |
| K01A2.10 | *K01A2.10* | -0.07 | NE |
| F38C2.5 | *F38C2.5* | -0.07 | NE |
| T26F2.1 | *T26F2.1* | -0.07 | NE |
| C17G1.7 | *C17G1.7* | -0.07 | NE |
| K01H12.1 | *K01H12.1* | -0.07 | NE |
| C55C3.4 | *C55C3.4* | -0.07 | NE |
| C17A2.1 | *nhr-257* | -0.07 | NE |
| F53F10.4 | *unc-108* | -0.07 | NE |
| R05D11.4 | *R05D11.4* | -0.07 | NE |
| T09A5.11 | *T09A5.11* | -0.07 | NE |
| F57C2.6 | *spat-1* | -0.071 | NE |
| F26A1.1 | *F26A1.1* | -0.071 | NE |
| T26C12.2 | *T26C12.2* | -0.071 | NE |
| C06E7.4 | *C06E7.4* | -0.072 | NE |
| T27F6.6 | *T27F6.6* | -0.072 | NE |
| C09H10.8 | *glb-4* | -0.073 | NE |
| K04F10.2 | *K04F10.2* | -0.073 | NE |
| Y44E3A.3 | *Y44E3A.3* | -0.073 | NE |
| Y37D8A.15 | *flp-14* | -0.073 | NE |
| Y45F10A.2 | *puf-3* | -0.073 | NE |
| F53A2.4 | *nud-1* | -0.073 | NE |
| Y48A6B.13 | *spat-2* | -0.073 | NE |
| W03B1.3 | *W03B1.3* | -0.074 | NE |
| Y38F2AL.5 | *nhr-236* | -0.074 | NE |
| F54D8.2 | *tag-174* | -0.074 | NE |
| C26B2.2 | *C26B2.2* | -0.074 | NE |
| C33F10.4 | *C33F10.4* | -0.074 | NE |
| F52D10.3 | *ftt-2* | -0.075 | NE |
| T19D2.2 | *prl-1* | -0.075 | NE |
| W02B12.2 | *rsp-2* | -0.075 | NE |
| F53A2.7 | *F53A2.7* | -0.075 | NE |
| F27C8.1 | *aat-1* | -0.076 | NE |
| Y116A8C.17 | *dct-13* | -0.076 | NE |
| F13D12.6 | *F13D12.6* | -0.076 | NE |
| ZK970.3 | *mdt-22* | -0.076 | NE |
| B0205.6 | *B0205.6* | -0.076 | NE |
| F40A3.3 | *F40A3.3* | -0.076 | NE |
| F15D3.7 | *F15D3.7* | -0.077 | NE |
| D1025.1 | *D1025.1* | -0.077 | NE |
| D1022.1 | *ubc-6* | -0.077 | NE |
| Y65B4BL.5 | *Y65B4BL.5* | -0.077 | NE |
| F14D2.11 | *F14D2.11* | -0.077 | NE |
| Y113G7B.23 | *psa-1* | -0.078 | NE |
| F13E6.1 | *F13E6.1* | -0.078 | NE |
| M7.2 | *klc-1* | -0.078 | NE |
| F56F11.4 | *F56F11.4* | -0.078 | NE |
| ZK637.2 | *ZK637.2* | -0.078 | NE |
| C55B7.9 | *mdt-18* | -0.078 | NE |
| C16E9.4 | *inx-1* | -0.078 | NE |
| B0228.5 | *trx-1* | -0.079 | NE |
| T01C3.9 | *T01C3.9* | -0.079 | NE |
| F28D1.3 | *thn-1* | -0.079 | NE |
| F22E5.2 | *F22E5.2* | -0.079 | NE |
| D2096.4 | *sqv-1* | -0.079 | NE |
| AH6.2 | *sfxn-1.1* | -0.08 | NE |
| Y47D3A.15 | *aakb-2* | -0.08 | NE |
| C31A11.8 | *C31A11.8* | -0.081 | NE |
| ZK353.8 | *ubxn-4* | -0.081 | NE |
| F42G9.9 | *ptl-1* | -0.082 | NE |
| C31H1.5 | *C31H1.5* | -0.082 | NE |
| C16C8.11 | *C16C8.11* | -0.082 | NE |
| T28C6.1 | *grsp-2* | -0.082 | NE |
| F45C12.5 | *fbxb-11* | -0.083 | NE |
| K05F6.9 | *fbxb-46* | -0.083 | NE |
| ZK652.3 | *tag-277* | -0.083 | NE |
| F39H11.1 | *F39H11.1* | -0.083 | NE |
| Y57A10A.8 | *Y57A10A.8* | -0.083 | NE |
| C09G4.5 | *mes-6* | -0.083 | NE |
| T13C5.4 | *T13C5.4* | -0.083 | NE |
| R13A5.5 | *ceh-13* | -0.084 | NE |
| T24C2.2 | *T24C2.2* | -0.084 | NE |
| C32E8.8 | *ptr-2* | -0.084 | NE |
| C52D10.3 | *C52D10.3* | -0.084 | NE |
| ZC513.6 | *oma-2* | -0.084 | NE |
| F35C8.7 | *chtl-1* | -0.084 | NE |
| C01F6.1 | *C01F6.1* | -0.085 | NE |
| F20C5.3 | *F20C5.3* | -0.085 | NE |
| T23G7.3 | *T23G7.3* | -0.085 | NE |
| T27F6.7 | *T27F6.7* | -0.086 | NE |
| C08A9.1 | *sod-3* | -0.086 | NE |
| F08G12.4 | *vhl-1* | -0.086 | NE |
| W01G7.4 | *W01G7.4* | -0.086 | NE |
| T28D9.2 | *rsp-5* | -0.086 | NE |
| C08H9.12 | *C08H9.12* | -0.087 | NE |
| T08G11.5 | *unc-29* | -0.087 | NE |
| F11G11.7 | *dnj-9* | -0.087 | NE |
| Y46G5A.11 | *Y46G5A.11* | -0.088 | NE |
| F10G7.5 | *F10G7.5* | -0.088 | NE |
| B0432.5 | *cat-2* | -0.088 | NE |
| Y71H9A.3 | *sto-4* | -0.088 | NE |
| C18E3.6 | *cas-2* | -0.088 | NE |
| F55G1.3 | *his-62* | -0.088 | NE |
| Y106G6H.13 | *Y106G6H.13* | -0.089 | NE |
| F28A10.5 | *F28A10.5* | -0.089 | NE |
| ZK546.11 | *gst-30* | -0.089 | NE |
| R05H5.3 | *R05H5.3* | -0.09 | NE |
| K11H12.7 | *K11H12.7* | -0.09 | NE |
| F10E7.9 | *F10E7.9* | -0.09 | NE |
| B0432.12 | *clec-117* | -0.09 | NE |
| D1007.5 | *D1007.5* | -0.091 | NE |
| T23B12.7 | *dnj-22* | -0.091 | NE |
| F37B1.8 | *gst-19* | -0.091 | NE |
| ZK1127.5 | *ZK1127.5* | -0.092 | NE |
| F56D5.1 | *col-121* | -0.092 | NE |
| H06H21.3 | *H06H21.3* | -0.092 | NE |
| F39B2.3 | *F39B2.3* | -0.092 | NE |
| W08E3.2 | *W08E3.2* | -0.092 | NE |
| F35E12.7 | *dct-17* | -0.092 | NE |
| Y49A3A.5 | *cyn-1* | -0.092 | NE |
| B0302.3 | *B0302.3* | -0.092 | NE |
| W08E12.8 | *W08E12.8* | -0.092 | NE |
| Y43F4A.1 | *Y43F4A.1* | -0.092 | NE |
| C18F10.8 | *srg-7* | -0.094 | NE |
| K01A2.8 | *mps-2* | -0.094 | NE |
| F53C3.13 | *F53C3.13* | -0.095 | NE |
| D2013.6 | *D2013.6* | -0.095 | NE |
| DY3.1 | *tin-13* | -0.095 | NE |
| T05H4.15 | *T05H4.15* | -0.095 | NE |
| ZK355.2 | *ZK355.2* | -0.095 | NE |
| C27H5.1 | *pdl-1* | -0.095 | NE |
| C18H9.1 | *C18H9.1* | -0.095 | NE |
| Y110A2AR.3 | *Y110A2AR.3* | -0.095 | NE |
| K05F1.8 | *K05F1.8* | -0.095 | NE |
| EGAP1.3 | *zmp-1* | -0.095 | NE |
| Y39E4A.1 | *Y39E4A.1* | -0.096 | NE |
| T23G5.5 | *dat-1* | -0.096 | NE |
| C56G7.3 | *C56G7.3* | -0.096 | NE |
| F32E10.5 | *F32E10.5* | -0.097 | NE |
| K10D2.2 | *pup-2* | -0.098 | NE |
| C17H12.13 | *C17H12.13* | -0.098 | NE |
| F42H10.3 | *F42H10.3* | -0.098 | NE |
| B0281.6 | *B0281.6* | -0.098 | NE |
| ZK1290.5 | *ZK1290.5* | -0.098 | NE |
| C35D10.13 | *C35D10.13* | -0.098 | NE |
| ZK1320.5 | *ZK1320.5* | -0.098 | NE |
| Y54E10BR.5 | *Y54E10BR.5* | -0.098 | NE |
| T07G12.11 | *zim-3* | -0.099 | NE |
| T20B12.3 | *T20B12.3* | -0.1 | NE |
| C18F10.5 | *srg-2* | -0.1 | NE |
| F26A1.2 | *fkh-5* | -0.1 | NE |
| C18D11.1 | *C18D11.1* | -0.101 | NE |
| T13B5.8 | *sut-1* | -0.101 | NE |
| F40F8.10 | *rps-9* | -0.101 | NE |
| T10E9.2 | *T10E9.2* | -0.101 | NE |
| Y92C3B.2 | *uaf-1* | -0.102 | NE |
| T20H4.4 | *adr-2* | -0.102 | NE |
| Y43F8A.3 | *Y43F8A.3* | -0.102 | NE |
| C24A3.6 | *twk-18* | -0.102 | NE |
| F38E9.5 | *F38E9.5* | -0.102 | NE |
| C07G2.1 | *cpg-1* | -0.102 | NE |
| F36H5.8 | *F36H5.8* | -0.103 | NE |
| Y17G7B.10 | *Y17G7B.10* | -0.103 | NE |
| T19B10.2 | *T19B10.2* | -0.103 | NE |
| Y48G9A.8 | *ppk-2* | -0.103 | NE |
| ZK892.3 | *ZK892.3* | -0.104 | NE |
| F35G12.12 | *F35G12.12* | -0.104 | NE |
| R03D7.5 | *R03D7.5* | -0.104 | NE |
| C50E10.4 | *sop-2* | -0.104 | NE |
| Y105C5A.13 | *Y105C5A.13* | -0.104 | NE |
| C46F11.4 | *C46F11.4* | -0.105 | NE |
| C52E2.3 | *C52E2.3* | -0.105 | NE |
| R01E6.5 | *R01E6.5* | -0.105 | NE |
| F46A9.6 | *mec-8* | -0.105 | NE |
| ZC395.5 | *ZC395.5* | -0.106 | NE |
| M176.8 | *M176.8* | -0.106 | NE |
| C08F11.3 | *C08F11.3* | -0.106 | NE |
| W02B3.4 | *W02B3.4* | -0.106 | NE |
| F54D8.1 | *dpy-17* | -0.106 | NE |
| F15E6.4 | *F15E6.4* | -0.106 | NE |
| Y32H12A.2 | *Y32H12A.2* | -0.106 | NE |
| K02F3.11 | *rnp-5* | -0.107 | NE |
| F56A8.6 | *cpf-2* | -0.107 | NE |
| Y57A10C.9 | *Y57A10C.9* | -0.107 | NE |
| Y37A1B.1 | *lst-3* | -0.108 | NE |
| ZK381.2 | *ZK381.2* | -0.108 | NE |
| C17H1.7 | *C17H1.7* | -0.109 | NE |
| Y51A2D.5 | *hmit-1.2* | -0.109 | NE |
| F13G3.9 | *mif-3* | -0.109 | NE |
| F56A6.1 | *sago-2* | -0.109 | NE |
| K08B12.1 | *K08B12.1* | -0.109 | NE |
| Y46C8AR.1 | *clec-76* | -0.11 | NE |
| F10C1.7 | *ifb-2* | -0.11 | NE |
| F46C5.2 | *F46C5.2* | -0.11 | NE |
| T23G5.1 | *rnr-1* | -0.11 | NE |
| Y39G8B.1 | *Y39G8B.1* | -0.111 | NE |
| ZK669.5 | *ZK669.5* | -0.111 | NE |
| Y19D10A.4 | *Y19D10A.4* | -0.111 | NE |
| F01G12.1 | *F01G12.1* | -0.111 | NE |
| F43E2.7 | *F43E2.7* | -0.111 | NE |
| F58E1.3 | *fbxb-26* | -0.112 | NE |
| K01C8.7 | *K01C8.7* | -0.112 | NE |
| M01E5.5 | *top-1* | -0.112 | NE |
| T20G5.6 | *unc-47* | -0.112 | NE |
| T05C12.6 | *mig-5* | -0.112 | NE |
| Y25C1A.6 | *Y25C1A.6* | -0.112 | NE |
| F35C5.7 | *clec-64* | -0.112 | NE |
| F57A10.4 | *F57A10.4* | -0.112 | NE |
| T23G7.1 | *dpl-1* | -0.112 | NE |
| W08F4.3 | *W08F4.3* | -0.112 | NE |
| T22C8.6 | *T22C8.6* | -0.112 | NE |
| Y6B3B.8 | *Y6B3B.8* | -0.112 | NE |
| F58A4.10 | *ubc-7* | -0.113 | NE |
| M04F3.4 | *M04F3.4* | -0.113 | NE |
| R09B5.6 | *hacd-1* | -0.113 | NE |
| Y116A8C.20 | *Y116A8C.20* | -0.113 | NE |
| K04H4.2 | *K04H4.2* | -0.113 | NE |
| F35C5.12 | *F35C5.12* | -0.113 | NE |
| JC8.12 | *JC8.12* | -0.113 | NE |
| C13B7.1 | *srt-1* | -0.114 | NE |
| B0285.4 | *B0285.4* | -0.114 | NE |
| F58G1.4 | *dct-18* | -0.114 | NE |
| D2024.7 | *gstk-2* | -0.114 | NE |
| C09G4.3 | *cks-1* | -0.114 | NE |
| DY3.2 | *lmn-1* | -0.114 | NE |
| Y32H12A.6 | *Y32H12A.6* | -0.114 | NE |
| H02I12.5 | *H02I12.5* | -0.114 | NE |
| ZK1058.4 | *ccdc-47* | -0.115 | NE |
| Y47G6A.14 | *Y47G6A.14* | -0.115 | NE |
| C04F12.4 | *rpl-14* | -0.115 | NE |
| C01H6.7 | *tag-298* | -0.115 | NE |
| F53F1.2 | *F53F1.2* | -0.115 | NE |
| C06C3.3 | *C06C3.3* | -0.116 | NE |
| F47D12.3 | *F47D12.3* | -0.116 | NE |
| K03D10.3 | *K03D10.3* | -0.116 | NE |
| C48B4.6 | *C48B4.6* | -0.116 | NE |
| F35C5.6 | *clec-63* | -0.116 | NE |
| Y37D8A.23 | *unc-25* | -0.117 | NE |
| ZC410.3 | *ZC410.3* | -0.117 | NE |
| F37H8.2 | *F37H8.2* | -0.117 | NE |
| F59H6.11 | *bath-5* | -0.118 | NE |
| T10E9.3 | *T10E9.3* | -0.118 | NE |
| Y57G11C.19 | *Y57G11C.19* | -0.118 | NE |
| C17G10.2 | *C17G10.2* | -0.119 | NE |
| F47G6.1 | *dyb-1* | -0.12 | NE |
| W10G6.1 | *W10G6.1* | -0.12 | NE |
| T14B4.6 | *dpy-2* | -0.12 | NE |
| C07E3.3 | *C07E3.3* | -0.12 | NE |
| Y48B6A.5 | *Y48B6A.5* | -0.12 | NE |
| K02F6.2 | *K02F6.2* | -0.12 | NE |
| Y110A7A.10 | *aap-1* | -0.12 | NE |
| Y54E2A.8 | *Y54E2A.8* | -0.12 | NE |
| K08B4.1 | *lag-1* | -0.12 | NE |
| C18A3.6 | *rab-3* | -0.121 | NE |
| C47F8.1 | *C47F8.1* | -0.121 | NE |
| C06C3.1 | *mel-11* | -0.121 | NE |
| F47D12.7 | *F47D12.7* | -0.121 | NE |
| F19C7.6 | *F19C7.6* | -0.121 | NE |
| C54C8.3 | *C54C8.3* | -0.121 | NE |
| T05D4.3 | *T05D4.3* | -0.122 | NE |
| C18A3.2 | *C18A3.2* | -0.122 | NE |
| F59A3.9 | *pup-3* | -0.122 | NE |
| M05D6.5 | *M05D6.5* | -0.122 | NE |
| C43G2.2 | *C43G2.2* | -0.123 | NE |
| F18A12.6 | *F18A12.6* | -0.123 | NE |
| F13E9.9 | *F13E9.9* | -0.123 | NE |
| C24H11.3 | *tbx-38* | -0.123 | NE |
| C41C4.8 | *cdc-48.2* | -0.124 | NE |
| T21B4.7 | *srh-69* | -0.124 | NE |
| Y53C12B.3 | *nos-3* | -0.124 | NE |
| F32H2.9 | *tba-6* | -0.125 | NE |
| R10D12.12 | *R10D12.12* | -0.125 | NE |
| W09G3.2 | *W09G3.2* | -0.125 | NE |
| C27C7.7 | *C27C7.7* | -0.125 | NE |
| C06B3.8 | *gcy-32* | -0.125 | NE |
| F26A3.4 | *F26A3.4* | -0.125 | NE |
| C27D6.3 | *C27D6.3* | -0.126 | NE |
| K10B4.5 | *srd-3* | -0.126 | NE |
| T26C5.2 | *T26C5.2* | -0.126 | NE |
| F53C3.1 | *F53C3.1* | -0.126 | NE |
| T27A1.5 | *T27A1.5* | -0.126 | NE |
| K10H10.1 | *K10H10.1* | -0.126 | NE |
| R153.1 | *pde-4* | -0.126 | NE |
| C14B9.4 | *plk-1* | -0.126 | NE |
| Y57G11C.32 | *Y57G11C.32* | -0.127 | NE |
| ZK484.8 | *nspd-1* | -0.127 | NE |
| C16D2.1 | *C16D2.1* | -0.127 | NE |
| Y54E10A.12 | *Y54E10A.12* | -0.128 | NE |
| T10B11.9 | *T10B11.9* | -0.128 | NE |
| ZK1010.7 | *col-97* | -0.128 | NE |
| C44H4.3 | *sym-1* | -0.128 | NE |
| C08G5.5 | *C08G5.5* | -0.128 | NE |
| Y49E10.4 | *Y49E10.4* | -0.129 | NE |
| C41D11.7 | *C41D11.7* | -0.129 | NE |
| W10G11.17 | *W10G11.17* | -0.129 | NE |
| Y45G12B.2 | *Y45G12B.2* | -0.13 | NE |
| AC7.2 | *soc-2* | -0.13 | NE |
| F49B2.2 | *fbxb-67* | -0.13 | NE |
| C32H11.1 | *C32H11.1* | -0.13 | NE |
| Y47H9C.5 | *dnj-27* | -0.13 | NE |
| T11F1.1 | *srw-60* | -0.13 | NE |
| F42G9.1 | *F42G9.1* | -0.13 | NE |
| D1022.3 | *D1022.3* | -0.13 | NE |
| Y106G6G.4 | *Y106G6G.4* | -0.13 | NE |
| VF36H2L.1 | *aph-1* | -0.131 | NE |
| F59B8.2 | *F59B8.2* | -0.131 | NE |
| Y37E11AR.6 | *vab-2* | -0.131 | NE |
| C50B6.2 | *nasp-2* | -0.132 | NE |
| C18D4.6 | *C18D4.6* | -0.132 | NE |
| C30H6.7 | *C30H6.7* | -0.132 | NE |
| F29D10.2 | *F29D10.2* | -0.133 | NE |
| F11A1.3 | *daf-12* | -0.133 | NE |
| T02H6.11 | *T02H6.11* | -0.133 | NE |
| T01A4.3 | *T01A4.3* | -0.133 | NE |
| K03B4.7 | *cpg-8* | -0.133 | NE |
| C46A5.5 | *C46A5.5* | -0.133 | NE |
| K01G5.7 | *tbb-1* | -0.134 | NE |
| F58D5.3 | *F58D5.3* | -0.134 | NE |
| W06D4.3 | *W06D4.3* | -0.134 | NE |
| B0414.5 | *cpb-3* | -0.135 | NE |
| C49F5.6 | *C49F5.6* | -0.135 | NE |
| F54C4.1 | *F54C4.1* | -0.135 | NE |
| C24H11.4 | *srd-74* | -0.135 | NE |
| C14A4.5 | *crn-5* | -0.135 | NE |
| C09H5.6 | *str-131* | -0.135 | NE |
| Y47G6A.1 | *inx-21* | -0.136 | NE |
| C34G6.2 | *tyr-4* | -0.136 | NE |
| R12H7.1 | *unc-9* | -0.136 | NE |
| ZC449.1 | *ZC449.1* | -0.136 | NE |
| F12E12.2 | *F12E12.2* | -0.136 | NE |
| R10H10.2 | *spe-26* | -0.136 | NE |
| K03H1.7 | *K03H1.7* | -0.136 | NE |
| C08H9.7 | *C08H9.7* | -0.136 | NE |
| C06E4.3 | *C06E4.3* | -0.136 | NE |
| K12H6.12 | *K12H6.12* | -0.136 | NE |
| F33G12.3 | *F33G12.3* | -0.136 | NE |
| F58F6.4 | *rfc-2* | -0.136 | NE |
| T11G6.7 | *T11G6.7* | -0.137 | NE |
| C44F1.2 | *C44F1.2* | -0.137 | NE |
| C32E8.2 | *rpl-13* | -0.137 | NE |
| K01C8.5 | *gei-14* | -0.137 | NE |
| C01G8.5 | *erm-1* | -0.138 | NE |
| W03D8.8 | *W03D8.8* | -0.138 | NE |
| F14E5.4 | *F14E5.4* | -0.138 | NE |
| C04F5.1 | *sid-1* | -0.138 | NE |
| E02H9.1 | *E02H9.1* | -0.138 | NE |
| B0244.2 | *ida-1* | -0.138 | NE |
| K07E8.5 | *K07E8.5* | -0.139 | NE |
| ZK177.4 | *ZK177.4* | -0.139 | NE |
| ZC317.7 | *ZC317.7* | -0.139 | NE |
| F32A5.5 | *aqp-1* | -0.139 | NE |
| F46F11.5 | *vha-10* | -0.139 | NE |
| C24G7.1 | *C24G7.1* | -0.14 | NE |
| F52F12.4 | *lsl-1* | -0.14 | NE |
| Y66H1B.4 | *spl-1* | -0.14 | NE |
| M153.1 | *M153.1* | -0.14 | NE |
| F43G6.5 | *F43G6.5* | -0.14 | NE |
| C46E10.1 | *C46E10.1* | -0.14 | NE |
| Y62E10A.14 | *Y62E10A.14* | -0.141 | NE |
| F43E2.4 | *haf-2* | -0.141 | NE |
| ZC334.2 | *ins-30* | -0.141 | NE |
| T08H4.1 | *rhgf-2* | -0.141 | NE |
| ZC334.1 | *ins-26* | -0.141 | NE |
| T28F3.6 | *ifta-2* | -0.141 | NE |
| Y37E11AL.3 | *Y37E11AL.3* | -0.141 | NE |
| C17D12.1 | *C17D12.1* | -0.142 | NE |
| C01B10.3 | *C01B10.3* | -0.143 | NE |
| F46H6.1 | *rhi-1* | -0.143 | NE |
| Y59A8A.1 | *csn-1* | -0.143 | NE |
| R07C3.6 | *R07C3.6* | -0.143 | NE |
| F01G10.1 | *F01G10.1* | -0.143 | NE |
| ZC101.2 | *unc-52* | -0.143 | NE |
| Y42G9A.1 | *Y42G9A.1* | -0.143 | NE |
| B0303.9 | *B0303.9* | -0.144 | NE |
| T23G4.1 | *tlp-1* | -0.144 | NE |
| C52D10.1 | *C52D10.1* | -0.145 | NE |
| K07C10.1 | *ptr-13* | -0.145 | NE |
| W09H1.6 | *lec-1* | -0.145 | NE |
| C18A3.3 | *C18A3.3* | -0.145 | NE |
| T08G5.5 | *T08G5.5* | -0.145 | NE |
| ZC506.3 | *pssy-1* | -0.146 | NE |
| C30B5.1 | *tag-319* | -0.146 | NE |
| Y106G6D.6 | *Y106G6D.6* | -0.147 | NE |
| Y39E4B.2 | *Y39E4B.2* | -0.147 | NE |
| LLC1.1 | *tra-3* | -0.147 | NE |
| H12D21.6 | *H12D21.6* | -0.147 | NE |
| W08F4.12 | *W08F4.12* | -0.148 | NE |
| F44F4.11 | *tba-4* | -0.148 | NE |
| T24H7.4 | *T24H7.4* | -0.148 | NE |
| F17C8.5 | *twk-6* | -0.148 | NE |
| W09H1.3 | *W09H1.3* | -0.149 | NE |
| F32A11.2 | *hpr-17* | -0.149 | NE |
| ZK418.6 | *ZK418.6* | -0.149 | NE |
| C38C10.2 | *C38C10.2* | -0.15 | NE |
| Y59C2A.2 | *Y59C2A.2* | -0.15 | NE |
| F22D6.12 | *gly-19* | -0.151 | NE |
| C33C12.6 | *srb-14* | -0.151 | NE |
| F37C12.14 | *F37C12.14* | -0.151 | NE |
| T22C8.8 | *vab-9* | -0.151 | NE |
| ZK945.7 | *ZK945.7* | -0.151 | NE |
| R10D12.13 | *R10D12.13* | -0.151 | NE |
| F11G11.5 | *F11G11.5* | -0.152 | NE |
| F58A4.1 | *F58A4.1* | -0.152 | NE |
| T23F4.3 | *T23F4.3* | -0.152 | NE |
| F37H8.5 | *F37H8.5* | -0.152 | NE |
| K12D12.5 | *K12D12.5* | -0.152 | NE |
| Y45F10B.5 | *sru-12* | -0.152 | NE |
| W03B1.6 | *W03B1.6* | -0.152 | NE |
| C44B9.3 | *C44B9.3* | -0.152 | NE |
| T10B9.3 | *cyp-13A6* | -0.152 | NE |
| W05F2.6 | *W05F2.6* | -0.152 | NE |
| C33A12.3 | *C33A12.3* | -0.153 | NE |
| R186.3 | *R186.3* | -0.153 | NE |
| Y71H2B.1 | *Y71H2B.1* | -0.153 | NE |
| CC4.2 | *nlp-15* | -0.153 | NE |
| C18E9.9 | *C18E9.9* | -0.153 | NE |
| C05C12.6 | *C05C12.6* | -0.153 | NE |
| Y48A6B.4 | *fipr-17* | -0.153 | NE |
| W07E6.1 | *nol-1* | -0.153 | NE |
| R07G3.1 | *cdc-42* | -0.154 | NE |
| C05H8.1 | *ckk-1* | -0.154 | NE |
| C45G9.10 | *C45G9.10* | -0.154 | NE |
| F52E10.5 | *ifa-3* | -0.154 | NE |
| F57B10.9 | *F57B10.9* | -0.155 | NE |
| T23B12.4 | *T23B12.4* | -0.155 | NE |
| T09B4.9 | *T09B4.9* | -0.155 | NE |
| C24G6.6 | *C24G6.6* | -0.155 | NE |
| C06A1.5 | *rpb-6* | -0.156 | NE |
| R10H10.4 | *R10H10.4* | -0.156 | NE |
| C50F7.5 | *C50F7.5* | -0.156 | NE |
| C32H11.9 | *C32H11.9* | -0.157 | NE |
| F54C1.5 | *dyf-1* | -0.157 | NE |
| T25G12.4 | *rab-6.2* | -0.157 | NE |
| C35D10.3 | *C35D10.3* | -0.158 | NE |
| C50F7.9 | *C50F7.9* | -0.158 | NE |
| C32H11.4 | *C32H11.4* | -0.158 | NE |
| K08B4.6 | *cpi-1* | -0.158 | NE |
| F17E9.1 | *col-116* | -0.158 | NE |
| C27A12.7 | *C27A12.7* | -0.159 | NE |
| F26D10.3 | *hsp-1* | -0.159 | NE |
| C06C3.5 | *C06C3.5* | -0.159 | NE |
| C07E3.9 | *C07E3.9* | -0.16 | NE |
| H06I04.3 | *H06I04.3* | -0.16 | NE |
| T03D8.3 | *sbt-1* | -0.16 | NE |
| C46C11.1 | *C46C11.1* | -0.16 | NE |
| B0491.7 | *B0491.7* | -0.16 | NE |
| F19C7.1 | *F19C7.1* | -0.161 | NE |
| F08G5.6 | *F08G5.6* | -0.161 | NE |
| W04G3.3 | *lpr-4* | -0.161 | NE |
| E03D2.1 | *nlp-13* | -0.162 | NE |
| F08D12.10 | *sdz-9* | -0.162 | NE |
| ZK1127.11 | *him-14* | -0.162 | NE |
| Y47D3A.21 | *Y47D3A.21* | -0.162 | NE |
| F26A3.5 | *F26A3.5* | -0.163 | NE |
| R13A1.8 | *glb-23* | -0.163 | NE |
| H38K22.1 | *evl-14* | -0.163 | NE |
| Y53F4B.15 | *asc-1* | -0.163 | NE |
| ZK652.6 | *ZK652.6* | -0.163 | NE |
| C53B7.4 | *asg-2* | -0.164 | NE |
| D1069.4 | *D1069.4* | -0.164 | NE |
| EEED8.2 | *EEED8.2* | -0.164 | NE |
| F22B3.7 | *F22B3.7* | -0.164 | NE |
| ZK1225.3 | *ZK1225.3* | -0.164 | NE |
| F15C11.1 | *sem-4* | -0.164 | NE |
| R148.6 | *heh-1* | -0.164 | NE |
| C33H5.6 | *C33H5.6* | -0.164 | NE |
| T16G12.7 | *T16G12.7* | -0.165 | NE |
| C50E3.5 | *C50E3.5* | -0.166 | NE |
| F54D10.5 | *F54D10.5* | -0.166 | NE |
| F42A10.4 | *efk-1* | -0.166 | NE |
| B0365.6 | *clec-41* | -0.166 | NE |
| F56D1.1 | *F56D1.1* | -0.166 | NE |
| Y22D7AL.5 | *hsp-60* | -0.166 | NE |
| Y69E1A.5 | *Y69E1A.5* | -0.167 | NE |
| R07C3.8 | *R07C3.8* | -0.167 | NE |
| D2045.8 | *D2045.8* | -0.167 | NE |
| W07G1.5 | *W07G1.5* | -0.167 | NE |
| F07A11.1 | *F07A11.1* | -0.167 | NE |
| ZK20.4 | *ZK20.4* | -0.167 | NE |
| C53H9.2 | *C53H9.2* | -0.168 | NE |
| F46F5.4 | *F46F5.4* | -0.168 | NE |
| F59E12.12 | *bli-2* | -0.168 | NE |
| ZK809.4 | *ent-1* | -0.169 | NE |
| H20J04.5 | *pfd-2* | -0.169 | NE |
| R07G3.6 | *R07G3.6* | -0.169 | NE |
| C06A5.9 | *rnf-1* | -0.169 | NE |
| D2013.2 | *wdfy-2* | -0.169 | NE |
| C06B3.3 | *cyp-35C1* | -0.17 | NE |
| Y46H3A.2 | *hsp-16.41* | -0.17 | NE |
| T24A11.2 | *xbx-5* | -0.17 | NE |
| C37A2.5 | *pqn-21* | -0.17 | NE |
| K10B2.2 | *K10B2.2* | -0.17 | NE |
| B0464.7 | *baf-1* | -0.171 | NE |
| F13G3.1 | *ztf-2* | -0.171 | NE |
| R13G10.2 | *amx-1* | -0.171 | NE |
| T04G9.1 | *T04G9.1* | -0.171 | NE |
| F23H11.1 | *bra-2* | -0.171 | NE |
| T01E8.4 | *T01E8.4* | -0.172 | NE |
| Y50D4A.1 | *Y50D4A.1* | -0.172 | NE |
| Y39A1A.1 | *Y39A1A.1* | -0.172 | NE |
| T12E12.4 | *drp-1* | -0.172 | NE |
| R11F4.3 | *R11F4.3* | -0.172 | NE |
| C43F9.8 | *efn-2* | -0.172 | NE |
| K06B9.4 | *K06B9.4* | -0.173 | NE |
| F23F1.6 | *F23F1.6* | -0.173 | NE |
| F20D6.4 | *srp-7* | -0.173 | NE |
| M04G12.2 | *cpz-2* | -0.174 | NE |
| R05D11.7 | *R05D11.7* | -0.174 | NE |
| C16A3.5 | *C16A3.5* | -0.174 | NE |
| F35E8.11 | *cdr-1* | -0.175 | NE |
| B0250.2 | *B0250.2* | -0.175 | NE |
| F52E4.4 | *twk-16* | -0.175 | NE |
| C45H4.14 | *C45H4.14* | -0.175 | NE |
| F32A5.2 | *F32A5.2* | -0.175 | NE |
| F26A1.10 | *nspd-9* | -0.175 | NE |
| H41C03.2 | *H41C03.2* | -0.175 | NE |
| T06C10.4 | *flp-10* | -0.176 | NE |
| B0523.1 | *kin-31* | -0.176 | NE |
| T19B10.8 | *T19B10.8* | -0.176 | NE |
| F45D11.9 | *F45D11.9* | -0.176 | NE |
| D2013.1 | *rab-39* | -0.177 | NE |
| F56A3.2 | *F56A3.2* | -0.177 | NE |
| E02H4.6 | *E02H4.6* | -0.177 | NE |
| R03D7.7 | *nos-1* | -0.177 | NE |
| B0303.5 | *B0303.5* | -0.177 | NE |
| F55F3.1 | *aakb-1* | -0.177 | NE |
| C56C10.1 | *vps-33.2* | -0.178 | NE |
| C49C3.9 | *C49C3.9* | -0.178 | NE |
| T27E7.1 | *T27E7.1* | -0.178 | NE |
| T13B5.3 | *T13B5.3* | -0.178 | NE |
| Y73B3A.19 | *Y73B3A.19* | -0.178 | NE |
| K11H12.5 | *K11H12.5* | -0.178 | NE |
| F49C12.6 | *F49C12.6* | -0.178 | NE |
| C48B4.9 | *C48B4.9* | -0.178 | NE |
| C53A5.3 | *hda-1* | -0.178 | NE |
| F07D3.2 | *flp-6* | -0.179 | NE |
| F55G11.8 | *F55G11.8* | -0.179 | NE |
| Y53G8AL.1 | *Y53G8AL.1* | -0.179 | NE |
| F21F3.5 | *unc-38* | -0.18 | NE |
| F07A5.7 | *unc-15* | -0.18 | NE |
| T19B10.7 | *ima-1* | -0.181 | NE |
| F57C2.1 | *btb-20* | -0.181 | NE |
| T03F1.1 | *T03F1.1* | -0.181 | NE |
| C07G1.4 | *wsp-1* | -0.181 | NE |
| T25G3.4 | *T25G3.4* | -0.181 | NE |
| T24A11.1 | *mtm-3* | -0.181 | NE |
| F11G11.12 | *col-73* | -0.182 | NE |
| Y17G7B.6 | *glb-29* | -0.182 | NE |
| C36A4.1 | *cyp-25A1* | -0.182 | NE |
| T20G5.12 | *T20G5.12* | -0.183 | NE |
| F28C6.2 | *F28C6.2* | -0.183 | NE |
| R03A10.4 | *nkat-3* | -0.183 | NE |
| F13H8.6 | *F13H8.6* | -0.184 | NE |
| M01D7.6 | *emr-1* | -0.184 | NE |
| T05A7.4 | *hmg-11* | -0.184 | NE |
| C48B4.12 | *C48B4.12* | -0.184 | NE |
| Y48B6A.1 | *Y48B6A.1* | -0.184 | NE |
| F59A6.2 | *F59A6.2* | -0.185 | NE |
| C27C12.1 | *C27C12.1* | -0.185 | NE |
| Y55F3AM.15 | *csn-4* | -0.185 | NE |
| F23H11.4 | *F23H11.4* | -0.185 | NE |
| ZC317.3 | *glc-3* | -0.185 | NE |
| F59F4.4 | *acl-1* | -0.186 | NE |
| C42D4.11 | *clec-179* | -0.186 | NE |
| T02B5.1 | *T02B5.1* | -0.186 | NE |
| C05D9.1 | *snx-1* | -0.187 | NE |
| F53G2.1 | *F53G2.1* | -0.187 | NE |
| H05L14.1 | *H05L14.1* | -0.187 | NE |
| W08E12.7 | *W08E12.7* | -0.188 | NE |
| T22F3.11 | *T22F3.11* | -0.188 | NE |
| F54C9.11 | *F54C9.11* | -0.188 | NE |
| F10G7.8 | *rpn-5* | -0.188 | NE |
| R05G6.8 | *plc-4* | -0.188 | NE |
| W10C8.1 | *ccb-2* | -0.189 | NE |
| T25D3.3 | *T25D3.3* | -0.189 | NE |
| T24H7.2 | *T24H7.2* | -0.189 | NE |
| ZK849.5 | *ZK849.5* | -0.19 | NE |
| H04D03.1 | *H04D03.1* | -0.19 | NE |
| C14A4.1 | *tag-242* | -0.19 | NE |
| Y48A6B.5 | *exos-1* | -0.19 | NE |
| T10B5.6 | *knl-3* | -0.191 | NE |
| T05H4.14 | *gad-1* | -0.191 | NE |
| ZK270.2 | *frm-1* | -0.191 | NE |
| C31H1.6 | *lntl-1* | -0.192 | NE |
| H34I24.1 | *H34I24.1* | -0.192 | NE |
| Y119C1B.3 | *Y119C1B.3* | -0.192 | NE |
| C45G7.3 | *ilys-3* | -0.192 | NE |
| R06B10.4 | *trp-2* | -0.192 | NE |
| Y113G7A.4 | *ncx-1* | -0.193 | NE |
| K02B2.4 | *inx-7* | -0.194 | NE |
| F31D4.3 | *fkb-6* | -0.194 | NE |
| Y49E10.15 | *snr-6* | -0.195 | NE |
| Y53C12C.1 | *Y53C12C.1* | -0.195 | NE |
| W02D9.6 | *W02D9.6* | -0.195 | NE |
| F53G12.5 | *mex-3* | -0.195 | NE |
| F23H11.5 | *F23H11.5* | -0.195 | NE |
| C47C12.3 | *ref-2* | -0.195 | NE |
| Y48B6A.7 | *ace-4* | -0.195 | NE |
| F42A10.2 | *nfm-1* | -0.196 | NE |
| F15E11.15 | *F15E11.15* | -0.196 | NE |
| C15H11.6 | *nxf-2* | -0.196 | NE |
| F35E12.9 | *F35E12.9* | -0.196 | NE |
| W10D9.5 | *W10D9.5* | -0.196 | NE |
| C06G3.8 | *C06G3.8* | -0.196 | NE |
| C36B1.7 | *C36B1.7* | -0.197 | NE |
| Y48E1B.1 | *ddl-2* | -0.197 | NE |
| ZC434.4 | *ZC434.4* | -0.197 | NE |
| C09G4.1 | *hyl-1* | -0.197 | NE |
| H01A20.1 | *nhr-3* | -0.197 | NE |
| F26A3.2 | *ncbp-2* | -0.197 | NE |
| ZK470.2 | *ZK470.2* | -0.197 | NE |
| B0464.8 | *tag-342* | -0.197 | NE |
| C14B9.2 | *C14B9.2* | -0.197 | NE |
| Y50D7A.3 | *Y50D7A.3* | -0.197 | NE |
| D2089.5 | *gbh-1* | -0.197 | NE |
| C39B5.4 | *fbxa-38* | -0.197 | NE |
| T02C12.2 | *T02C12.2* | -0.197 | NE |
| B0205.10 | *B0205.10* | -0.197 | NE |
| EEED8.8 | *ndx-6* | -0.198 | NE |
| C27A7.4 | *che-11* | -0.198 | NE |
| Y38F1A.8 | *Y38F1A.8* | -0.198 | NE |
| H38K22.5 | *gly-6* | -0.199 | NE |
| T09E8.3 | *T09E8.3* | -0.199 | NE |
| F08G2.8 | *F08G2.8* | -0.2 | NE |
| W06B11.3 | *dct-11* | -0.2 | NE |
| Y48C3A.5 | *Y48C3A.5* | -0.2 | NE |
| T25C12.1 | *lin-14* | -0.2 | NE |
| C17H12.10 | *C17H12.10* | -0.2 | NE |
| W08D2.7 | *mtr-4* | -0.2 | NE |
| ZK550.3 | *ZK550.3* | -0.2 | NE |
| Y53F4B.22 | *Y53F4B.22* | -0.2 | NE |
| T28A8.3 | *T28A8.3* | -0.201 | NE |
| T10E9.1 | *T10E9.1* | -0.201 | NE |
| K07E12.2 | *K07E12.2* | -0.202 | NE |
| F43G9.11 | *ces-1* | -0.202 | NE |
| F53A10.1 | *F53A10.1* | -0.202 | NE |
| M02G9.3 | *M02G9.3* | -0.202 | NE |
| B0496.6 | *B0496.6* | -0.202 | NE |
| F32B6.8 | *F32B6.8* | -0.203 | NE |
| Y57G11C.22 | *Y57G11C.22* | -0.203 | NE |
| C08E3.10 | *fbxa-158* | -0.203 | NE |
| K11D9.1 | *klp-7* | -0.203 | NE |
| K06A5.2 | *K06A5.2* | -0.204 | NE |
| R06B9.3 | *R06B9.3* | -0.205 | NE |
| E02H1.5 | *E02H1.5* | -0.205 | NE |
| R10H10.5 | *gpa-7* | -0.205 | NE |
| C33A12.15 | *ttr-9* | -0.205 | NE |
| R03D7.8 | *R03D7.8* | -0.205 | NE |
| W02D3.9 | *unc-37* | -0.206 | NE |
| F23C8.1 | *F23C8.1* | -0.206 | NE |
| K10H10.5 | *K10H10.5* | -0.206 | NE |
| C35E7.9 | *C35E7.9* | -0.206 | NE |
| ZK1251.3 | *ZK1251.3* | -0.207 | NE |
| ZK809.3 | *ZK809.3* | -0.207 | NE |
| C18D1.3 | *flp-4* | -0.207 | NE |
| C43E11.5 | *C43E11.5* | -0.207 | NE |
| K11H3.1 | *gpdh-2* | -0.208 | NE |
| Y57A10A.16 | *Y57A10A.16* | -0.209 | NE |
| M6.1 | *ifc-2* | -0.209 | NE |
| Y54E10BL.6 | *mek-2* | -0.209 | NE |
| F32B6.4 | *F32B6.4* | -0.209 | NE |
| W02C12.3 | *hlh-30* | -0.21 | NE |
| T21B10.4 | *T21B10.4* | -0.21 | NE |
| R03C1.3 | *cog-1* | -0.21 | NE |
| C30A5.5 | *snb-5* | -0.21 | NE |
| C08H9.5 | *old-1* | -0.211 | NE |
| T09A12.2 | *T09A12.2* | -0.212 | NE |
| ZK75.3 | *ins-3* | -0.212 | NE |
| ZK265.3 | *ZK265.3* | -0.212 | NE |
| F35H8.4 | *F35H8.4* | -0.212 | NE |
| Y61A9LA.7 | *Y61A9LA.7* | -0.212 | NE |
| C32B5.1 | *C32B5.1* | -0.212 | NE |
| Y95B8A.11 | *Y95B8A.11* | -0.212 | NE |
| F35C11.2 | *F35C11.2* | -0.212 | NE |
| Y49F6B.9 | *Y49F6B.9* | -0.212 | NE |
| H04D03.3 | *H04D03.3* | -0.213 | NE |
| F29B9.4 | *psr-1* | -0.213 | NE |
| C16C10.1 | *C16C10.1* | -0.214 | NE |
| F39B2.11 | *mtx-1* | -0.214 | NE |
| Y62E10A.17 | *Y62E10A.17* | -0.214 | NE |
| C46F11.5 | *C46F11.5* | -0.214 | NE |
| F21H7.5 | *F21H7.5* | -0.214 | NE |
| C04F1.2 | *C04F1.2* | -0.215 | NE |
| Y53G8AM.4 | *Y53G8AM.4* | -0.215 | NE |
| Y64G10A.5 | *Y64G10A.5* | -0.215 | NE |
| F22D6.10 | *col-60* | -0.215 | NE |
| C17G10.6 | *C17G10.6* | -0.215 | NE |
| C23G10.10 | *C23G10.10* | -0.215 | NE |
| F53C3.12 | *bcmo-2* | -0.216 | NE |
| Y47H9C.10 | *fbxa-216* | -0.216 | NE |
| F43C11.2 | *F43C11.2* | -0.216 | NE |
| Y54G11B.1 | *Y54G11B.1* | -0.216 | NE |
| K06H7.4 | *grp-1* | -0.216 | NE |
| F22D6.9 | *F22D6.9* | -0.216 | NE |
| F58F6.2 | *col-105* | -0.217 | NE |
| R11E3.3 | *R11E3.3* | -0.217 | NE |
| D2024.1 | *D2024.1* | -0.217 | NE |
| C32B5.16 | *sdz-4* | -0.217 | NE |
| C54E4.4 | *C54E4.4* | -0.218 | NE |
| F10B5.3 | *F10B5.3* | -0.218 | NE |
| ZK637.15 | *ZK637.15* | -0.218 | NE |
| ZC328.3 | *ZC328.3* | -0.218 | NE |
| R09B3.5 | *mag-1* | -0.219 | NE |
| Y62E10A.10 | *Y62E10A.10* | -0.219 | NE |
| M03A8.1 | *dhs-28* | -0.219 | NE |
| M04D8.6 | *xbx-3* | -0.219 | NE |
| C36A4.6 | *cyp-25A4* | -0.219 | NE |
| B0495.7 | *B0495.7* | -0.22 | NE |
| B0285.7 | *B0285.7* | -0.22 | NE |
| B0336.3 | *B0336.3* | -0.22 | NE |
| F10F2.9 | *pqn-29* | -0.22 | NE |
| ZK856.11 | *ZK856.11* | -0.221 | NE |
| R166.1 | *mab-10* | -0.221 | NE |
| C09G12.5 | *C09G12.5* | -0.221 | NE |
| C07E3.10 | *C07E3.10* | -0.221 | NE |
| F26D10.10 | *gln-5* | -0.221 | NE |
| ZC416.8 | *cha-1* | -0.221 | NE |
| F58B3.9 | *ttr-50* | -0.222 | NE |
| B0261.6 | *B0261.6* | -0.222 | NE |
| W04H10.4 | *clec-118* | -0.222 | NE |
| T15D6.12 | *T15D6.12* | -0.223 | NE |
| C39E9.9 | *col-132* | -0.223 | NE |
| T10B11.3 | *ztf-4* | -0.223 | NE |
| T01G9.4 | *npp-2* | -0.223 | NE |
| C36B1.10 | *gska-3* | -0.223 | NE |
| C15H7.4 | *C15H7.4* | -0.224 | NE |
| Y119D3B.17 | *pes-4* | -0.224 | NE |
| F45C12.4 | *btb-9* | -0.224 | NE |
| C36A4.10 | *C36A4.10* | -0.224 | NE |
| K09A9.2 | *rab-14* | -0.224 | NE |
| ZK892.2 | *nlt-1* | -0.225 | NE |
| F54H5.3 | *F54H5.3* | -0.225 | NE |
| C33H5.13 | *C33H5.13* | -0.226 | NE |
| Y81G3A.1 | *Y81G3A.1* | -0.226 | NE |
| K04C2.2 | *K04C2.2* | -0.226 | NE |
| ZK863.4 | *ZK863.4* | -0.226 | NE |
| F54C8.4 | *F54C8.4* | -0.227 | NE |
| H19N07.3 | *H19N07.3* | -0.227 | NE |
| F55A12.4 | *dhs-2* | -0.227 | NE |
| F49E12.7 | *F49E12.7* | -0.227 | NE |
| F17C11.9 | *F17C11.9* | -0.227 | NE |
| Y42G9A.3 | *Y42G9A.3* | -0.228 | NE |
| Y50D7A.4 | *Y50D7A.4* | -0.228 | NE |
| K10B4.6 | *cwn-1* | -0.228 | NE |
| C04G2.2 | *C04G2.2* | -0.228 | NE |
| C04C3.3 | *C04C3.3* | -0.229 | NE |
| F57B9.1 | *F57B9.1* | -0.229 | NE |
| F32B5.2 | *F32B5.2* | -0.23 | NE |
| F47B3.2 | *F47B3.2* | -0.23 | NE |
| ZC168.2 | *ZC168.2* | -0.23 | NE |
| ZK112.2 | *ncl-1* | -0.23 | NE |
| C16C10.12 | *wht-3* | -0.23 | NE |
| C17F4.1 | *clec-124* | -0.23 | NE |
| F18A1.4 | *lir-2* | -0.231 | NE |
| T04B2.6 | *dhs-31* | -0.231 | NE |
| F09E5.1 | *pkc-3* | -0.231 | NE |
| C30B5.3 | *cpb-2* | -0.231 | NE |
| C49D10.3 | *srh-250* | -0.232 | NE |
| M28.7 | *nph-1* | -0.232 | NE |
| C35E7.6 | *C35E7.6* | -0.232 | NE |
| Y71F9B.2 | *Y71F9B.2* | -0.232 | NE |
| F45E4.11 | *F45E4.11* | -0.232 | NE |
| W10D9.4 | *nfyb-1* | -0.232 | NE |
| K02F2.4 | *ulp-5* | -0.232 | NE |
| T28F12.2 | *unc-62* | -0.233 | NE |
| Y4C6B.4 | *Y4C6B.4* | -0.233 | NE |
| ZC196.2 | *ZC196.2* | -0.233 | NE |
| K10D11.2 | *K10D11.2* | -0.234 | NE |
| Y76A2B.5 | *Y76A2B.5* | -0.234 | NE |
| F41D3.1 | *nhr-82* | -0.234 | NE |
| Y46G5A.28 | *Y46G5A.28* | -0.234 | NE |
| Y48B6A.12 | *Y48B6A.12* | -0.234 | NE |
| C33F10.7 | *lact-5* | -0.234 | NE |
| C40A11.7 | *C40A11.7* | -0.235 | NE |
| C56A3.2 | *ttr-44* | -0.235 | NE |
| F15G9.1 | *F15G9.1* | -0.235 | NE |
| C08H9.13 | *C08H9.13* | -0.235 | NE |
| C09D4.5 | *rpl-19* | -0.236 | NE |
| W07G1.7 | *W07G1.7* | -0.237 | NE |
| F08H9.1 | *coh-3* | -0.237 | NE |
| E01G4.1 | *E01G4.1* | -0.238 | NE |
| M04F3.1 | *rpa-2* | -0.238 | NE |
| R01H10.8 | *cnk-1* | -0.238 | NE |
| T13A10.7 | *srv-30* | -0.239 | NE |
| H14E04.3 | *H14E04.3* | -0.24 | NE |
| C32D5.2 | *sma-6* | -0.24 | NE |
| C16C2.4 | *C16C2.4* | -0.24 | NE |
| F36D1.6 | *F36D1.6* | -0.24 | NE |
| Y18D10A.8 | *Y18D10A.8* | -0.241 | NE |
| M01A8.1 | *M01A8.1* | -0.241 | NE |
| T14G10.6 | *tsp-12* | -0.241 | NE |
| W04B5.5 | *W04B5.5* | -0.241 | NE |
| F40F11.3 | *F40F11.3* | -0.242 | NE |
| C50F2.7 | *C50F2.7* | -0.243 | NE |
| F36A4.3 | *F36A4.3* | -0.243 | NE |
| M01D1.2 | *math-34* | -0.243 | NE |
| Y40H7A.10 | *Y40H7A.10* | -0.243 | NE |
| C09H10.10 | *C09H10.10* | -0.244 | NE |
| Y87G2A.10 | *vps-28* | -0.245 | NE |
| C15C6.4 | *C15C6.4* | -0.245 | NE |
| W03G9.5 | *W03G9.5* | -0.245 | NE |
| C54C8.4 | *C54C8.4* | -0.245 | NE |
| ZK550.4 | *ZK550.4* | -0.245 | NE |
| F46E10.10 | *F46E10.10* | -0.245 | NE |
| W05B2.6 | *col-92* | -0.246 | NE |
| C11E4.6 | *C11E4.6* | -0.247 | NE |
| D1086.3 | *D1086.3* | -0.247 | NE |
| T07A9.10 | *T07A9.10* | -0.247 | NE |
| F49F1.5 | *F49F1.5* | -0.248 | NE |
| C26C6.6 | *C26C6.6* | -0.248 | NE |
| Y51H1A.3 | *Y51H1A.3* | -0.249 | NE |
| C14B1.7 | *C14B1.7* | -0.249 | NE |
| Y105C5B.21 | *jac-1* | -0.249 | NE |
| C09C7.1 | *zig-4* | -0.25 | NE |
| F01F1.5 | *dpf-4* | -0.25 | NE |
| ZC410.5 | *ZC410.5* | -0.251 | NE |
| T06D8.8 | *rpn-9* | -0.251 | NE |
| F55A11.2 | *syn-3* | -0.251 | NE |
| W03C9.4 | *lin-29* | -0.251 | NE |
| C10G8.5 | *ncx-2* | -0.252 | NE |
| F10G7.3 | *asf-1* | -0.253 | NE |
| F36F12.7 | *F36F12.7* | -0.253 | NE |
| B0285.8 | *B0285.8* | -0.253 | NE |
| T17E9.2 | *nmt-1* | -0.253 | NE |
| F09B9.2 | *unc-115* | -0.253 | NE |
| T19A5.1 | *T19A5.1* | -0.253 | NE |
| Y50D4A.4 | *Y50D4A.4* | -0.253 | NE |
| B0035.1 | *B0035.1* | -0.254 | NE |
| F32B6.11 | *F32B6.11* | -0.254 | NE |
| C34C6.4 | *C34C6.4* | -0.255 | NE |
| C30F12.6 | *C30F12.6* | -0.255 | NE |
| F42G4.2 | *F42G4.2* | -0.255 | NE |
| E02A10.1 | *E02A10.1* | -0.256 | NE |
| E01H11.1 | *pkc-2* | -0.256 | NE |
| C01G5.5 | *C01G5.5* | -0.256 | NE |
| T09A5.8 | *T09A5.8* | -0.256 | NE |
| F36F12.5 | *clec-207* | -0.257 | NE |
| D2045.9 | *D2045.9* | -0.257 | NE |
| T05C1.1 | *T05C1.1* | -0.257 | NE |
| Y67D8C.5 | *eel-1* | -0.257 | NE |
| F56D6.2 | *clec-67* | -0.259 | NE |
| F33D11.3 | *col-54* | -0.26 | NE |
| F46A9.3 | *twk-29* | -0.26 | NE |
| F08B4.4 | *F08B4.4* | -0.26 | NE |
| F36D1.8 | *F36D1.8* | -0.26 | NE |
| Y56A3A.2 | *Y56A3A.2* | -0.26 | NE |
| B0336.9 | *swp-1* | -0.26 | NE |
| F25H2.11 | *tct-1* | -0.261 | NE |
| T05A6.2 | *cki-2* | -0.261 | NE |
| Y80D3A.10 | *nlp-42* | -0.261 | NE |
| Y48G10A.1 | *Y48G10A.1* | -0.261 | NE |
| F01F1.8 | *cct-6* | -0.261 | NE |
| M110.2 | *twk-3* | -0.261 | NE |
| F25G6.8 | *F25G6.8* | -0.261 | NE |
| T28D6.2 | *tba-7* | -0.262 | NE |
| Y87G2A.11 | *Y87G2A.11* | -0.262 | NE |
| Y40B1B.7 | *Y40B1B.7* | -0.262 | NE |
| Y75B8A.7 | *Y75B8A.7* | -0.262 | NE |
| C07D8.6 | *C07D8.6* | -0.263 | NE |
| F23B2.6 | *aly-2* | -0.264 | NE |
| F57B10.11 | *bag-1* | -0.264 | NE |
| C27F2.8 | *C27F2.8* | -0.265 | NE |
| Y17G9B.1 | *Y17G9B.1* | -0.266 | NE |
| Y94H6A.6 | *ubc-8* | -0.266 | NE |
| F38E1.9 | *F38E1.9* | -0.266 | NE |
| C10G8.7 | *ceh-33* | -0.267 | NE |
| C54G4.8 | *cyc-1* | -0.267 | NE |
| F37C12.2 | *F37C12.2* | -0.268 | NE |
| B0218.6 | *clec-51* | -0.268 | NE |
| F35G2.5 | *F35G2.5* | -0.268 | NE |
| H23N18.4 | *H23N18.4* | -0.268 | NE |
| K07E3.3 | *dao-3* | -0.27 | NE |
| Y48A6C.3 | *Y48A6C.3* | -0.27 | NE |
| T01C3.3 | *T01C3.3* | -0.27 | NE |
| F15D3.2 | *clec-101* | -0.27 | NE |
| F58G6.1 | *amph-1* | -0.27 | NE |
| F18H3.4 | *F18H3.4* | -0.271 | NE |
| F28D9.4 | *F28D9.4* | -0.271 | NE |
| Y53F4B.29 | *gst-26* | -0.271 | NE |
| Y56A3A.21 | *trap-4* | -0.272 | NE |
| F18H3.5 | *cdk-4* | -0.272 | NE |
| F59B2.11 | *F59B2.11* | -0.272 | NE |
| Y48G10A.2 | *Y48G10A.2* | -0.272 | NE |
| F33A8.9 | *col-83* | -0.272 | NE |
| Y38E10A.10 | *lips-16* | -0.273 | NE |
| K09F6.4 | *K09F6.4* | -0.273 | NE |
| W09C3.6 | *gsp-3* | -0.273 | NE |
| R07H5.4 | *sdz-27* | -0.273 | NE |
| C16C2.2 | *eat-16* | -0.274 | NE |
| T05C12.4 | *T05C12.4* | -0.274 | NE |
| F35C12.3 | *F35C12.3* | -0.275 | NE |
| Y27F2A.3 | *sri-40* | -0.275 | NE |
| C32D5.3 | *C32D5.3* | -0.275 | NE |
| F54C9.5 | *rpl-5* | -0.276 | NE |
| C09H10.7 | *C09H10.7* | -0.276 | NE |
| ZC373.5 | *ZC373.5* | -0.276 | NE |
| Y73E7A.1 | *Y73E7A.1* | -0.276 | NE |
| F14H3.6 | *F14H3.6* | -0.276 | NE |
| R155.1 | *R155.1* | -0.276 | NE |
| M03F4.7 | *calu-1* | -0.277 | NE |
| F40F9.9 | *aqp-4* | -0.277 | NE |
| W09D6.6 | *hmt-1* | -0.277 | NE |
| T26A5.5 | *T26A5.5* | -0.277 | NE |
| F53A9.1 | *F53A9.1* | -0.278 | NE |
| F28F8.6 | *atx-3* | -0.278 | NE |
| T24C4.5 | *T24C4.5* | -0.279 | NE |
| M01D1.3 | *btb-11* | -0.279 | NE |
| F33G12.2 | *F33G12.2* | -0.279 | NE |
| C48D5.1 | *nhr-6* | -0.28 | NE |
| F14F7.4 | *F14F7.4* | -0.28 | NE |
| W10G11.3 | *W10G11.3* | -0.28 | NE |
| ZK6.11 | *ZK6.11* | -0.281 | NE |
| Y51H4A.1 | *Y51H4A.1* | -0.282 | NE |
| R12C12.3 | *R12C12.3* | -0.283 | NE |
| Y53F4B.3 | *Y53F4B.3* | -0.283 | NE |
| Y17G9B.7 | *fbxb-77* | -0.284 | NE |
| K09C8.2 | *K09C8.2* | -0.285 | NE |
| VF13D12L.3 | *VF13D12L.3* | -0.286 | NE |
| T04D3.5 | *T04D3.5* | -0.286 | NE |
| Y87G2A.13 | *Y87G2A.13* | -0.286 | NE |
| F53E4.1 | *F53E4.1* | -0.288 | NE |
| F28H1.2 | *cpn-3* | -0.288 | NE |
| F45H11.1 | *sptf-1* | -0.288 | NE |
| M04F3.5 | *M04F3.5* | -0.288 | NE |
| C24A11.9 | *coq-1* | -0.288 | NE |
| R05F9.4 | *R05F9.4* | -0.288 | NE |
| K10C3.2 | *K10C3.2* | -0.288 | NE |
| Y119C1B.6 | *Y119C1B.6* | -0.288 | NE |
| ZC416.1 | *ZC416.1* | -0.288 | NE |
| F15E11.13 | *F15E11.13* | -0.288 | NE |
| M110.7 | *M110.7* | -0.289 | NE |
| C33H5.15 | *sgo-1* | -0.29 | NE |
| C31B8.8 | *C31B8.8* | -0.29 | NE |
| K03H1.6 | *ttr-1* | -0.29 | NE |
| M03C11.1 | *M03C11.1* | -0.291 | NE |
| T27A1.6 | *mab-9* | -0.291 | NE |
| K08D10.7 | *scrm-8* | -0.292 | NE |
| R10E4.3 | *R10E4.3* | -0.292 | NE |
| C37A5.1 | *C37A5.1* | -0.293 | NE |
| Y67A6A.2 | *nhr-62* | -0.293 | NE |
| C44B7.7 | *C44B7.7* | -0.293 | NE |
| K04D7.3 | *gta-1* | -0.294 | NE |
| F10G8.9 | *F10G8.9* | -0.294 | NE |
| C01G6.6 | *tag-165* | -0.294 | NE |
| B0564.9 | *B0564.9* | -0.295 | NE |
| F49E11.10 | *scl-2* | -0.296 | NE |
| R03D7.6 | *gst-5* | -0.296 | NE |
| F56E3.4 | *fax-1* | -0.297 | NE |
| F54D5.7 | *F54D5.7* | -0.298 | NE |
| K08D8.3 | *K08D8.3* | -0.298 | NE |
| C17E4.4 | *C17E4.4* | -0.298 | NE |
| T07C4.10 | *T07C4.10* | -0.299 | NE |
| F56E10.1 | *F56E10.1* | -0.299 | NE |
| C17D12.2 | *unc-75* | -0.299 | NE |
| W09C5.1 | *W09C5.1* | -0.3 | NE |
| C23H3.4 | *sptl-1* | -0.301 | NE |
| F01D4.3 | *F01D4.3* | -0.301 | NE |
| B0464.3 | *nlp-36* | -0.303 | NE |
| Y57A10C.6 | *Y57A10C.6* | -0.303 | NE |
| R02F2.5 | *R02F2.5* | -0.303 | NE |
| ZK1290.2 | *tph-1* | -0.303 | NE |
| F21D12.5 | *F21D12.5* | -0.303 | NE |
| C50F4.7 | *his-37* | -0.304 | NE |
| F53A10.2 | *F53A10.2* | -0.304 | NE |
| T07D10.3 | *T07D10.3* | -0.305 | NE |
| Y77E11A.2 | *Y77E11A.2* | -0.306 | NE |
| Y71G12A.2 | *Y71G12A.2* | -0.306 | NE |
| ZC239.8 | *sri-51* | -0.307 | NE |
| F35D11.9 | *clec-138* | -0.307 | NE |
| T22C1.3 | *T22C1.3* | -0.308 | NE |
| C34F11.2 | *C34F11.2* | -0.308 | NE |
| C30A5.2 | *rfs-1* | -0.308 | NE |
| F43E2.2 | *rpb-4* | -0.308 | NE |
| K10D3.2 | *unc-14* | -0.308 | NE |
| R07H5.2 | *cpt-2* | -0.309 | NE |
| C33F10.12 | *C33F10.12* | -0.309 | NE |
| F07H5.2 | *sgn-1* | -0.31 | NE |
| T24D1.3 | *T24D1.3* | -0.31 | NE |
| T10B11.8 | *T10B11.8* | -0.31 | NE |
| F45C12.7 | *btb-6* | -0.311 | NE |
| F56D12.6 | *F56D12.6* | -0.311 | NE |
| C53B7.3 | *C53B7.3* | -0.311 | NE |
| F01D5.8 | *F01D5.8* | -0.311 | NE |
| K07C11.5 | *tag-225* | -0.311 | NE |
| F01F1.2 | *F01F1.2* | -0.311 | NE |
| Y44E3A.5 | *Y44E3A.5* | -0.312 | NE |
| C46H11.6 | *C46H11.6* | -0.312 | NE |
| T04A8.6 | *T04A8.6* | -0.312 | NE |
| K09C4.5 | *K09C4.5* | -0.313 | NE |
| EEED8.1 | *mel-47* | -0.314 | NE |
| F45E12.3 | *cul-4* | -0.314 | NE |
| R06C7.1 | *R06C7.1* | -0.314 | NE |
| C29H12.1 | *rrt-2* | -0.314 | NE |
| C45G3.1 | *aspm-1* | -0.314 | NE |
| T05H4.13 | *alh-4* | -0.315 | NE |
| Y54E5A.6 | *Y54E5A.6* | -0.316 | NE |
| F58B3.8 | *dsl-5* | -0.316 | NE |
| F30A10.2 | *F30A10.2* | -0.316 | NE |
| F29D10.5 | *F29D10.5* | -0.316 | NE |
| F54B11.3 | *unc-84* | -0.317 | NE |
| W08E12.2 | *W08E12.2* | -0.317 | NE |
| C17G1.4 | *C17G1.4* | -0.317 | NE |
| Y25C1A.1 | *clec-123* | -0.317 | NE |
| C52E4.1 | *cpr-1* | -0.318 | NE |
| T02G5.13 | *mmaa-1* | -0.318 | NE |
| C16C10.9 | *C16C10.9* | -0.318 | NE |
| F26A1.3 | *F26A1.3* | -0.318 | NE |
| F08C6.6 | *apy-1* | -0.318 | NE |
| F52B10.1 | *nmy-1* | -0.319 | NE |
| W06A11.2 | *W06A11.2* | -0.32 | NE |
| F46F5.16 | *F46F5.16* | -0.32 | NE |
| C53D5.4 | *ztf-3* | -0.32 | NE |
| C15A11.2 | *C15A11.2* | -0.32 | NE |
| F36H12.4 | *F36H12.4* | -0.321 | NE |
| F44F1.3 | *F44F1.3* | -0.321 | NE |
| R13A5.11 | *R13A5.11* | -0.322 | NE |
| Y17G9A.1 | *str-173* | -0.322 | NE |
| R08C7.8 | *R08C7.8* | -0.322 | NE |
| C12C8.2 | *C12C8.2* | -0.323 | NE |
| K10C8.3 | *K10C8.3* | -0.323 | NE |
| Y39B6A.20 | *asp-1* | -0.323 | NE |
| C34D4.15 | *col-113* | -0.323 | NE |
| C06A1.3 | *C06A1.3* | -0.324 | NE |
| K07C6.4 | *cyp-35B1* | -0.324 | NE |
| T23B3.2 | *T23B3.2* | -0.325 | NE |
| C08B11.8 | *C08B11.8* | -0.326 | NE |
| C53B4.4 | *C53B4.4* | -0.326 | NE |
| T10H4.11 | *cyp-34A2* | -0.327 | NE |
| F25B5.7 | *psf-1* | -0.327 | NE |
| K03B4.2 | *K03B4.2* | -0.327 | NE |
| F57B1.7 | *F57B1.7* | -0.328 | NE |
| C50D2.1 | *C50D2.1* | -0.328 | NE |
| ZC262.2 | *ZC262.2* | -0.328 | NE |
| DH11.4 | *DH11.4* | -0.328 | NE |
| F11H8.4 | *cyk-1* | -0.328 | NE |
| W09C3.4 | *W09C3.4* | -0.329 | NE |
| K04G7.3 | *ogt-1* | -0.329 | NE |
| Y43C5A.6 | *rad-51* | -0.329 | NE |
| T19H12.2 | *T19H12.2* | -0.329 | NE |
| T05B11.3 | *clic-1* | -0.33 | NE |
| C08H9.11 | *C08H9.11* | -0.33 | NE |
| Y102E9.1 | *odr-4* | -0.331 | NE |
| F22B3.1 | *his-64* | -0.331 | NE |
| Y48G8AL.6 | *smg-2* | -0.331 | NE |
| F23H11.2 | *F23H11.2* | -0.331 | NE |
| T07H3.3 | *math-38* | -0.332 | NE |
| F10E7.10 | *F10E7.10* | -0.332 | NE |
| W09C3.3 | *W09C3.3* | -0.333 | NE |
| ZK265.2 | *col-63* | -0.333 | NE |
| C28A5.6 | *C28A5.6* | -0.334 | NE |
| F45G2.4 | *F45G2.4* | -0.334 | NE |
| F47F6.4 | *F47F6.4* | -0.334 | NE |
| C18A11.1 | *C18A11.1* | -0.336 | NE |
| W05E10.3 | *ceh-32* | -0.336 | NE |
| F59E12.11 | *F59E12.11* | -0.336 | NE |
| F47A4.3 | *rrc-1* | -0.337 | NE |
| T22A3.2 | *hsp-12.1* | -0.338 | NE |
| D1007.8 | *D1007.8* | -0.338 | NE |
| T19D12.4 | *T19D12.4* | -0.339 | NE |
| R07H5.3 | *R07H5.3* | -0.339 | NE |
| C18D1.2 | *C18D1.2* | -0.339 | NE |
| C02B10.4 | *C02B10.4* | -0.34 | NE |
| B0464.5 | *spk-1* | -0.34 | NE |
| F40E3.2 | *F40E3.2* | -0.34 | NE |
| ZK892.7 | *sdz-38* | -0.34 | NE |
| M04C7.1 | *gpa-15* | -0.34 | NE |
| F54E12.3 | *his-56* | -0.341 | NE |
| R02D3.5 | *R02D3.5* | -0.342 | NE |
| Y18D10A.13 | *pad-1* | -0.342 | NE |
| T24H10.7 | *T24H10.7* | -0.342 | NE |
| R07C3.5 | *R07C3.5* | -0.342 | NE |
| C27H5.6 | *C27H5.6* | -0.342 | NE |
| F43C1.4 | *nhr-20* | -0.342 | NE |
| F42G4.5 | *F42G4.5* | -0.342 | NE |
| F59B2.9 | *F59B2.9* | -0.343 | NE |
| C56A3.7 | *cav-2* | -0.343 | NE |
| F52D2.2 | *rgs-8.1* | -0.343 | NE |
| T02G5.8 | *kat-1* | -0.343 | NE |
| B0334.6 | *B0334.6* | -0.343 | NE |
| Y48A6C.5 | *pha-1* | -0.343 | NE |
| F44B9.3 | *cit-1.2* | -0.343 | NE |
| Y79H2A.6 | *arx-3* | -0.344 | NE |
| ZC308.4 | *ZC308.4* | -0.344 | NE |
| T03F1.8 | *T03F1.8* | -0.344 | NE |
| T05F1.9 | *T05F1.9* | -0.344 | NE |
| Y54G11A.6 | *ctl-1* | -0.345 | NE |
| Y17G9B.5 | *Y17G9B.5* | -0.346 | NE |
| ZK1290.10 | *ZK1290.10* | -0.347 | NE |
| Y47D3A.25 | *rab-35* | -0.347 | NE |
| ZK550.5 | *ZK550.5* | -0.348 | NE |
| K01A11.1 | *K01A11.1* | -0.348 | NE |
| DH11.1 | *DH11.1* | -0.349 | NE |
| C08C3.4 | *C08C3.4* | -0.349 | NE |
| T19D12.5 | *T19D12.5* | -0.349 | NE |
| F14B6.4 | *F14B6.4* | -0.35 | NE |
| K07G5.4 | *K07G5.4* | -0.35 | NE |
| C54C8.2 | *C54C8.2* | -0.35 | NE |
| K01A2.2 | *far-7* | -0.352 | NE |
| C05D12.4 | *C05D12.4* | -0.352 | NE |
| D2092.4 | *D2092.4* | -0.352 | NE |
| T23B3.6 | *T23B3.6* | -0.352 | NE |
| F02E11.5 | *scl-15* | -0.352 | NE |
| F54D7.1 | *F54D7.1* | -0.353 | NE |
| F16C3.2 | *F16C3.2* | -0.354 | NE |
| C13B4.2 | *usp-14* | -0.354 | NE |
| T23F6.1 | *T23F6.1* | -0.355 | NE |
| ZK20.5 | *rpn-12* | -0.355 | NE |
| F58A4.4 | *pri-1* | -0.355 | NE |
| C56G2.5 | *C56G2.5* | -0.355 | NE |
| F22D3.6 | *F22D3.6* | -0.355 | NE |
| T13C2.3 | *T13C2.3* | -0.356 | NE |
| M05B5.5 | *hlh-2* | -0.356 | NE |
| H14A12.5 | *H14A12.5* | -0.356 | NE |
| F39G3.7 | *prx-6* | -0.356 | NE |
| C09G9.6 | *oma-1* | -0.358 | NE |
| C56C10.7 | *C56C10.7* | -0.358 | NE |
| B0511.9 | *cdc-26* | -0.358 | NE |
| Y59H11AM.1 | *Y59H11AM.1* | -0.359 | NE |
| R05H5.7 | *R05H5.7* | -0.359 | NE |
| C15H9.6 | *hsp-3* | -0.36 | NE |
| Y47D3B.10 | *dpy-18* | -0.36 | NE |
| F33A8.7 | *F33A8.7* | -0.36 | NE |
| F42A9.8 | *F42A9.8* | -0.361 | NE |
| Y57A10B.7 | *Y57A10B.7* | -0.361 | NE |
| ZK185.1 | *ZK185.1* | -0.361 | NE |
| C52D10.13 | *col-138* | -0.361 | NE |
| Y113G7B.5 | *fog-2* | -0.363 | NE |
| K02F6.7 | *K02F6.7* | -0.363 | NE |
| C27A2.5 | *C27A2.5* | -0.363 | NE |
| Y40C5A.2 | *ocr-4* | -0.364 | NE |
| F25B4.8 | *F25B4.8* | -0.364 | NE |
| F40F11.4 | *F40F11.4* | -0.365 | NE |
| F41E6.2 | *grd-5* | -0.365 | NE |
| R151.2 | *R151.2* | -0.366 | NE |
| C16A3.1 | *C16A3.1* | -0.366 | NE |
| F52C6.11 | *bath-2* | -0.366 | NE |
| F01G4.5 | *F01G4.5* | -0.366 | NE |
| R107.7 | *gst-1* | -0.367 | NE |
| C06G3.10 | *cogc-2* | -0.367 | NE |
| B0047.3 | *bath-24* | -0.367 | NE |
| B0564.1 | *exos-4.1* | -0.367 | NE |
| R160.1 | *dpy-23* | -0.367 | NE |
| F23F1.2 | *F23F1.2* | -0.368 | NE |
| F53A2.8 | *mtm-6* | -0.371 | NE |
| F38H4.9 | *let-92* | -0.372 | NE |
| C36A4.2 | *cyp-25A2* | -0.373 | NE |
| M142.2 | *cut-6* | -0.373 | NE |
| B0280.12 | *B0280.12* | -0.373 | NE |
| C47E12.2 | *C47E12.2* | -0.373 | NE |
| T03F1.3 | *pgk-1* | -0.374 | NE |
| F16D3.5 | *F16D3.5* | -0.374 | NE |
| F26E4.6 | *F26E4.6* | -0.374 | NE |
| C05C10.5 | *C05C10.5* | -0.374 | NE |
| Y39A3B.2 | *lgc-42* | -0.374 | NE |
| Y23H5A.4 | *Y23H5A.4* | -0.376 | NE |
| F41D3.9 | *F41D3.9* | -0.376 | NE |
| C08E3.9 | *fbxa-166* | -0.377 | NE |
| F57F5.4 | *add-2* | -0.377 | NE |
| C14A4.11 | *C14A4.11* | -0.377 | NE |
| C27A2.3 | *ify-1* | -0.379 | NE |
| ZK622.3 | *pmt-1* | -0.379 | NE |
| F38E11.1 | *hsp-12.3* | -0.38 | NE |
| T16H12.3 | *T16H12.3* | -0.38 | NE |
| Y39A3CR.4 | *ddp-1* | -0.381 | NE |
| C06A12.4 | *gcy-27* | -0.381 | NE |
| Y87G2A.1 | *Y87G2A.1* | -0.383 | NE |
| F49F1.1 | *F49F1.1* | -0.384 | NE |
| C23H5.3 | *xbx-4* | -0.384 | NE |
| M110.4 | *ifg-1* | -0.384 | NE |
| R119.2 | *R119.2* | -0.385 | NE |
| C05C10.3 | *C05C10.3* | -0.385 | NE |
| D2023.1 | *D2023.1* | -0.386 | NE |
| T14G8.1 | *chd-3* | -0.387 | NE |
| C25A1.4 | *C25A1.4* | -0.387 | NE |
| Y53F4B.14 | *Y53F4B.14* | -0.387 | NE |
| Y45F10D.2 | *Y45F10D.2* | -0.387 | NE |
| R03D7.4 | *R03D7.4* | -0.388 | NE |
| F31E3.5 | *eft-3* | -0.388 | NE |
| F09G8.7 | *F09G8.7* | -0.388 | NE |
| Y73F8A.1 | *pkd-2* | -0.389 | NE |
| D1025.4 | *nspc-20* | -0.39 | NE |
| F31C3.4 | *F31C3.4* | -0.39 | NE |
| T22H2.4 | *T22H2.4* | -0.39 | NE |
| C14A4.2 | *dap-3* | -0.39 | NE |
| C27H5.7 | *dyf-13* | -0.39 | NE |
| Y106G6H.1 | *Y106G6H.1* | -0.391 | NE |
| C18D11.4 | *rsp-8* | -0.392 | NE |
| T01H3.4 | *T01H3.4* | -0.393 | NE |
| ZC416.6 | *ZC416.6* | -0.394 | NE |
| F37D6.6 | *tag-68* | -0.394 | NE |
| C15F1.3 | *tra-2* | -0.395 | NE |
| Y54E10BL.1 | *Y54E10BL.1* | -0.395 | NE |
| F28D1.1 | *F28D1.1* | -0.396 | NE |
| R09H10.2 | *R09H10.2* | -0.396 | NE |
| Y38C1BA.3 | *col-109* | -0.396 | NE |
| F21D5.6 | *F21D5.6* | -0.396 | NE |
| F08B1.1 | *vhp-1* | -0.398 | NE |
| ZK1025.10 | *nhr-245* | -0.398 | NE |
| T15D6.8 | *T15D6.8* | -0.398 | NE |
| T08B2.10 | *rps-17* | -0.399 | NE |
| Y47G6A.3 | *Y47G6A.3* | -0.399 | NE |
| T28C6.3 | *T28C6.3* | -0.4 | NE |
| B0513.6 | *B0513.6* | -0.4 | NE |
| F53G12.4 | *F53G12.4* | -0.4 | NE |
| F02A9.4 | *F02A9.4* | -0.401 | NE |
| Y65B4BL.2 | *deps-1* | -0.401 | NE |
| T05D4.2 | *T05D4.2* | -0.401 | NE |
| C27H5.4 | *C27H5.4* | -0.402 | NE |
| Y62E10A.15 | *cyp-31A5* | -0.403 | NE |
| VF13D12L.1 | *VF13D12L.1* | -0.403 | NE |
| K06H7.6 | *apc-2* | -0.403 | NE |
| T05D4.5 | *T05D4.5* | -0.403 | NE |
| C40C9.1 | *twk-20* | -0.404 | NE |
| Y48B6A.2 | *rpl-43* | -0.405 | NE |
| Y45F10B.1 | *tsp-5* | -0.405 | NE |
| F48A9.1 | *F48A9.1* | -0.405 | NE |
| T22C1.4 | *T22C1.4* | -0.405 | NE |
| T05A8.3 | *T05A8.3* | -0.406 | NE |
| H23L24.3 | *ttll-11* | -0.408 | NE |
| F44E5.5 | *F44E5.5* | -0.409 | NE |
| T14G8.3 | *T14G8.3* | -0.409 | NE |
| T25F10.2 | *dbl-1* | -0.409 | NE |
| Y38F1A.1 | *Y38F1A.1* | -0.409 | NE |
| T08B2.4 | *T08B2.4* | -0.409 | NE |
| F58A3.1 | *ldb-1* | -0.41 | NE |
| W02B8.2 | *W02B8.2* | -0.411 | NE |
| C02C2.1 | *tyr-1* | -0.411 | NE |
| F35D6.1 | *fem-1* | -0.411 | NE |
| W02D9.7 | *W02D9.7* | -0.412 | NE |
| W10G11.1 | *W10G11.1* | -0.412 | NE |
| C35D10.5 | *C35D10.5* | -0.413 | NE |
| F15A2.2 | *tre-4* | -0.413 | NE |
| Y63D3A.4 | *Y63D3A.4* | -0.413 | NE |
| H41C03.3 | *H41C03.3* | -0.413 | NE |
| F56C9.5 | *acbp-4* | -0.413 | NE |
| T05C12.2 | *acr-14* | -0.414 | NE |
| cTel54X.1 | *fbxa-6* | -0.415 | NE |
| Y48G1A.3 | *Y48G1A.3* | -0.416 | NE |
| T04A8.8 | *T04A8.8* | -0.417 | NE |
| C08H9.2 | *C08H9.2* | -0.417 | NE |
| T10B11.6 | *T10B11.6* | -0.418 | NE |
| Y65B4BL.4 | *Y65B4BL.4* | -0.418 | NE |
| Y26D4A.4 | *clec-107* | -0.42 | NE |
| T20G5.11 | *rde-4* | -0.42 | NE |
| C49C3.6 | *C49C3.6* | -0.421 | NE |
| C25D7.7 | *rap-2* | -0.422 | NE |
| Y75B8A.12 | *osm-12* | -0.422 | NE |
| T11B7.4 | *alp-1* | -0.423 | NE |
| F10B5.2 | *F10B5.2* | -0.423 | NE |
| ZC328.4 | *san-1* | -0.423 | NE |
| W04B5.4 | *W04B5.4* | -0.423 | NE |
| F10E7.1 | *F10E7.1* | -0.424 | NE |
| F08D12.7 | *F08D12.7* | -0.424 | NE |
| Y41C4A.6 | *Y41C4A.6* | -0.425 | NE |
| Y46E12A.1 | *cnc-6* | -0.425 | NE |
| W08D2.4 | *fat-3* | -0.426 | NE |
| C01G6.1 | *aqp-2* | -0.426 | NE |
| ZK20.1 | *ZK20.1* | -0.426 | NE |
| C45G9.9 | *C45G9.9* | -0.427 | NE |
| B0496.1 | *B0496.1* | -0.428 | NE |
| Y53F4B.30 | *gst-27* | -0.428 | NE |
| W09B6.1 | *pod-2* | -0.429 | NE |
| B0379.4 | *scpl-1* | -0.43 | NE |
| C17H1.3 | *C17H1.3* | -0.43 | NE |
| F43G9.12 | *F43G9.12* | -0.431 | NE |
| F47B3.4 | *F47B3.4* | -0.431 | NE |
| F56H6.6 | *F56H6.6* | -0.432 | NE |
| F29C12.5 | *bath-45* | -0.432 | NE |
| F07H5.3 | *F07H5.3* | -0.432 | NE |
| K08C9.4 | *col-65* | -0.432 | NE |
| C09G5.8 | *C09G5.8* | -0.433 | NE |
| W03B1.4 | *srs-1* | -0.433 | NE |
| Y63D3A.7 | *Y63D3A.7* | -0.433 | NE |
| F01F1.12 | *F01F1.12* | -0.433 | NE |
| T10B11.1 | *pcbd-1* | -0.433 | NE |
| T05B9.1 | *T05B9.1* | -0.433 | NE |
| R06C1.2 | *R06C1.2* | -0.434 | NE |
| T22A3.8 | *lam-3* | -0.434 | NE |
| Y18D10A.9 | *Y18D10A.9* | -0.436 | NE |
| C36A4.8 | *brc-1* | -0.436 | NE |
| F10G2.2 | *F10G2.2* | -0.436 | NE |
| C05D11.3 | *tag-170* | -0.437 | NE |
| T09B4.4 | *T09B4.4* | -0.437 | NE |
| F17A9.2 | *F17A9.2* | -0.438 | NE |
| B0041.5 | *B0041.5* | -0.438 | NE |
| T05G5.1 | *T05G5.1* | -0.439 | NE |
| Y102E9.2 | *Y102E9.2* | -0.439 | NE |
| R05F9.6 | *R05F9.6* | -0.44 | NE |
| Y6B3B.9 | *Y6B3B.9* | -0.44 | NE |
| R08C7.1 | *R08C7.1* | -0.441 | NE |
| F44F1.4 | *F44F1.4* | -0.441 | NE |
| B0207.3 | *gpa-14* | -0.441 | NE |
| C54C6.2 | *ben-1* | -0.442 | NE |
| C27H5.5 | *col-36* | -0.443 | NE |
| C24G6.4 | *nhr-47* | -0.443 | NE |
| F57B9.3 | *F57B9.3* | -0.443 | NE |
| T26A5.6 | *T26A5.6* | -0.444 | NE |
| C32D5.6 | *C32D5.6* | -0.445 | NE |
| F40H3.3 | *F40H3.3* | -0.445 | NE |
| C05D11.2 | *vps-16* | -0.446 | NE |
| C23G10.2 | *C23G10.2* | -0.447 | NE |
| T26E3.5 | *T26E3.5* | -0.447 | NE |
| F42H10.2 | *F42H10.2* | -0.448 | NE |
| C47B2.4 | *pbs-2* | -0.449 | NE |
| T23H2.2 | *snt-4* | -0.45 | NE |
| B0218.8 | *clec-52* | -0.45 | NE |
| F56C9.10 | *F56C9.10* | -0.45 | NE |
| K02A6.3 | *K02A6.3* | -0.451 | NE |
| F49C12.5 | *F49C12.5* | -0.451 | NE |
| F21A3.4 | *F21A3.4* | -0.452 | NE |
| Y38H6C.14 | *Y38H6C.14* | -0.453 | NE |
| C27A2.2 | *rpl-22* | -0.453 | NE |
| T22C8.3 | *T22C8.3* | -0.453 | NE |
| T20F10.2 | *T20F10.2* | -0.453 | NE |
| ZK829.1 | *ZK829.1* | -0.454 | NE |
| T05A7.7 | *T05A7.7* | -0.454 | NE |
| BE10.4 | *BE10.4* | -0.454 | NE |
| F32E10.6 | *F32E10.6* | -0.455 | NE |
| K08A2.1 | *K08A2.1* | -0.456 | NE |
| ZK632.12 | *ZK632.12* | -0.457 | NE |
| T02D1.7 | *T02D1.7* | -0.457 | NE |
| W03F11.1 | *W03F11.1* | -0.457 | NE |
| ZK973.6 | *anc-1* | -0.458 | NE |
| Y48A6B.3 | *Y48A6B.3* | -0.459 | NE |
| C29F9.4 | *C29F9.4* | -0.459 | NE |
| Y48C3A.2 | *Y48C3A.2* | -0.459 | NE |
| F35C11.1 | *nlp-5* | -0.46 | NE |
| F16B4.5 | *F16B4.5* | -0.46 | NE |
| F49D11.2 | *F49D11.2* | -0.46 | NE |
| Y87G2A.8 | *gpi-1* | -0.461 | NE |
| Y73F8A.32 | *Y73F8A.32* | -0.461 | NE |
| R02F11.1 | *R02F11.1* | -0.463 | NE |
| Y48C3A.11 | *Y48C3A.11* | -0.463 | NE |
| K07A1.13 | *K07A1.13* | -0.464 | NE |
| Y52B11B.1 | *Y52B11B.1* | -0.464 | NE |
| C42D8.8 | *apl-1* | -0.465 | NE |
| C32F10.5 | *hmg-3* | -0.465 | NE |
| F44B9.2 | *F44B9.2* | -0.465 | NE |
| T10B9.1 | *cyp-13A4* | -0.465 | NE |
| C34G6.7 | *stam-1* | -0.465 | NE |
| C29A12.4 | *nrx-1* | -0.466 | NE |
| W03C9.5 | *W03C9.5* | -0.466 | NE |
| T22C1.6 | *T22C1.6* | -0.468 | NE |
| F57B9.7 | *flap-1* | -0.468 | NE |
| F01D5.10 | *F01D5.10* | -0.468 | NE |
| C16C8.5 | *C16C8.5* | -0.469 | NE |
| Y47H10A.5 | *Y47H10A.5* | -0.47 | NE |
| Y46G5A.1 | *Y46G5A.1* | -0.472 | NE |
| F35D2.5 | *syd-1* | -0.472 | NE |
| F08G12.10 | *inx-2* | -0.472 | NE |
| Y47H10A.2 | *Y47H10A.2* | -0.472 | NE |
| Y47H10A.3 | *Y47H10A.3* | -0.472 | NE |
| F47H4.10 | *skr-5* | -0.473 | NE |
| Y57A10A.31 | *Y57A10A.31* | -0.474 | NE |
| F27E11.3 | *cfz-2* | -0.474 | NE |
| F49E11.11 | *scl-3* | -0.476 | NE |
| K07A1.5 | *K07A1.5* | -0.476 | NE |
| F57B10.7 | *tre-1* | -0.478 | NE |
| F38A5.8 | *F38A5.8* | -0.48 | NE |
| R11H6.2 | *R11H6.2* | -0.48 | NE |
| Y41C4A.13 | *Y41C4A.13* | -0.48 | NE |
| C23G10.5 | *C23G10.5* | -0.481 | E |
| F16D3.1 | *tba-5* | -0.481 | E |
| F31E3.2 | *F31E3.2* | -0.481 | E |
| Y87G2A.14 | *ndx-8* | -0.481 | E |
| T20D3.7 | *vps-26* | -0.482 | E |
| K08C7.7 | *K08C7.7* | -0.483 | E |
| Y39A3CL.6 | *pvf-1* | -0.484 | E |
| R12E2.7 | *R12E2.7* | -0.484 | E |
| R53.2 | *R53.2* | -0.485 | E |
| Y73F8A.19 | *tag-64* | -0.486 | E |
| F55B12.3 | *sel-10* | -0.486 | E |
| F57B9.4 | *coq-2* | -0.486 | E |
| ZK265.4 | *ceh-8* | -0.487 | E |
| ZK546.6 | *msp-152* | -0.489 | E |
| F21C3.1 | *twk-1* | -0.489 | E |
| K01A2.1 | *sgcb-1* | -0.49 | E |
| T12D8.9 | *T12D8.9* | -0.49 | E |
| F47C12.10 | *srh-222* | -0.49 | E |
| C46H11.8 | *phat-1* | -0.49 | E |
| Y74C10AR.1 | *eif-3.I* | -0.49 | E |
| E02D9.1 | *E02D9.1* | -0.491 | E |
| F59D12.2 | *F59D12.2* | -0.491 | E |
| T05B9.2 | *T05B9.2* | -0.491 | E |
| R06C7.2 | *R06C7.2* | -0.492 | E |
| F33H2.7 | *set-10* | -0.493 | E |
| Y105E8A.1 | *Y105E8A.1* | -0.493 | E |
| F47G4.7 | *smd-1* | -0.493 | E |
| Y53G8AR.9 | *Y53G8AR.9* | -0.494 | E |
| Y116A8C.37 | *Y116A8C.37* | -0.495 | E |
| K10C3.4 | *K10C3.4* | -0.495 | E |
| F15A4.13 | *fbxb-102* | -0.496 | E |
| Y49E10.22 | *pstk-1* | -0.496 | E |
| F54F7.3 | *F54F7.3* | -0.497 | E |
| C56E6.6 | *C56E6.6* | -0.497 | E |
| C50F2.4 | *C50F2.4* | -0.497 | E |
| C49H3.4 | *C49H3.4* | -0.5 | E |
| C41C4.7 | *ctns-1* | -0.502 | E |
| C48B4.1 | *C48B4.1* | -0.503 | E |
| K09F6.3 | *K09F6.3* | -0.504 | E |
| W01A8.5 | *W01A8.5* | -0.504 | E |
| R06A4.3 | *R06A4.3* | -0.505 | E |
| C54G4.7 | *C54G4.7* | -0.505 | E |
| Y48G9A.7 | *Y48G9A.7* | -0.506 | E |
| W05H5.4 | *srh-27* | -0.507 | E |
| T10B11.5 | *T10B11.5* | -0.507 | E |
| Y49E10.24 | *Y49E10.24* | -0.509 | E |
| F46F11.2 | *cey-2* | -0.51 | E |
| Y106G6H.16 | *Y106G6H.16* | -0.51 | E |
| Y47G7B.2 | *Y47G7B.2* | -0.511 | E |
| M106.5 | *cap-2* | -0.512 | E |
| C44H4.5 | *tap-1* | -0.513 | E |
| M02F4.7 | *clec-265* | -0.514 | E |
| ZK973.8 | *ZK973.8* | -0.514 | E |
| Y53G8AM.5 | *Y53G8AM.5* | -0.515 | E |
| C05D11.7 | *C05D11.7* | -0.516 | E |
| T05G5.10 | *iff-1* | -0.518 | E |
| Y38E10A.22 | *Y38E10A.22* | -0.518 | E |
| Y18D10A.21 | *Y18D10A.21* | -0.519 | E |
| T21C12.3 | *T21C12.3* | -0.52 | E |
| K11G9.6 | *mtl-1* | -0.52 | E |
| F07H5.10 | *F07H5.10* | -0.52 | E |
| F01F1.1 | *F01F1.1* | -0.522 | E |
| ZK1025.3 | *ZK1025.3* | -0.522 | E |
| F36F2.2 | *F36F2.2* | -0.523 | E |
| C44B11.3 | *mec-12* | -0.525 | E |
| F59A2.2 | *F59A2.2* | -0.525 | E |
| C37H5.3 | *C37H5.3* | -0.526 | E |
| Y9C9A.5 | *Y9C9A.5* | -0.526 | E |
| Y48A6B.11 | *rsa-2* | -0.527 | E |
| T10D4.6 | *T10D4.6* | -0.527 | E |
| H08M01.2 | *H08M01.2* | -0.528 | E |
| T10B9.2 | *cyp-13A5* | -0.528 | E |
| Y37A1B.11 | *egl-23* | -0.528 | E |
| C17C3.9 | *C17C3.9* | -0.528 | E |
| R11F4.2 | *R11F4.2* | -0.529 | E |
| M01E5.3 | *M01E5.3* | -0.529 | E |
| T01G9.3 | *T01G9.3* | -0.53 | E |
| W04E12.1 | *fbxa-131* | -0.532 | E |
| W06F12.1 | *lit-1* | -0.532 | E |
| C34C6.5 | *sphk-1* | -0.533 | E |
| F39H2.4 | *syp-3* | -0.533 | E |
| C26D10.2 | *hel-1* | -0.533 | E |
| C32A3.1 | *sel-8* | -0.534 | E |
| Y53G8AR.8 | *Y53G8AR.8* | -0.534 | E |
| DY3.6 | *mfb-1* | -0.535 | E |
| F26H9.3 | *F26H9.3* | -0.536 | E |
| C26B2.6 | *elpc-4* | -0.539 | E |
| T25B9.10 | *T25B9.10* | -0.54 | E |
| T07C4.7 | *mev-1* | -0.54 | E |
| F10C5.1 | *mat-3* | -0.54 | E |
| C07G1.1 | *try-2* | -0.543 | E |
| Y39E4B.6 | *Y39E4B.6* | -0.543 | E |
| B0222.2 | *B0222.2* | -0.545 | E |
| C49H3.3 | *C49H3.3* | -0.546 | E |
| C52B9.1 | *cka-2* | -0.546 | E |
| F54D10.8 | *F54D10.8* | -0.546 | E |
| Y54E10A.3 | *Y54E10A.3* | -0.548 | E |
| Y54E2A.9 | *Y54E2A.9* | -0.549 | E |
| F29G6.2 | *F29G6.2* | -0.549 | E |
| F59A3.4 | *F59A3.4* | -0.549 | E |
| Y38H6C.3 | *dct-14* | -0.551 | E |
| F02H6.2 | *F02H6.2* | -0.551 | E |
| K08F4.5 | *K08F4.5* | -0.553 | E |
| F11D5.1 | *F11D5.1* | -0.554 | E |
| ZK666.7 | *clec-61* | -0.554 | E |
| C41H7.1 | *C41H7.1* | -0.554 | E |
| D2007.5 | *atg-13* | -0.554 | E |
| Y47G6A.8 | *crn-1* | -0.555 | E |
| F53B6.4 | *F53B6.4* | -0.557 | E |
| F37A4.4 | *F37A4.4* | -0.557 | E |
| Y46H3A.6 | *gly-7* | -0.559 | E |
| T21B4.3 | *T21B4.3* | -0.56 | E |
| R05H10.1 | *R05H10.1* | -0.563 | E |
| T21C9.2 | *vps-54* | -0.564 | E |
| R17.1 | *fbxb-85* | -0.564 | E |
| F58B6.2 | *inft-1* | -0.565 | E |
| C09E9.2 | *C09E9.2* | -0.567 | E |
| F49E12.1 | *F49E12.1* | -0.569 | E |
| F02E8.3 | *aps-2* | -0.57 | E |
| F09D12.1 | *grd-10* | -0.57 | E |
| F27E5.1 | *F27E5.1* | -0.57 | E |
| Y53G8AR.5 | *Y53G8AR.5* | -0.57 | E |
| F56D1.4 | *clr-1* | -0.571 | E |
| C17H1.5 | *C17H1.5* | -0.573 | E |
| C12C8.3 | *lin-41* | -0.575 | E |
| F49F1.12 | *F49F1.12* | -0.575 | E |
| Y45G12C.15 | *srj-20* | -0.575 | E |
| K02E7.9 | *btb-10* | -0.577 | E |
| Y9D1A.1 | *Y9D1A.1* | -0.578 | E |
| Y38A8.3 | *ulp-2* | -0.58 | E |
| C15C6.2 | *C15C6.2* | -0.58 | E |
| F02A9.1 | *F02A9.1* | -0.582 | E |
| Y56A3A.20 | *ccf-1* | -0.583 | E |
| Y48G9A.6 | *Y48G9A.6* | -0.583 | E |
| C32D5.5 | *set-4* | -0.583 | E |
| T23D8.1 | *mom-5* | -0.583 | E |
| B0491.1 | *B0491.1* | -0.585 | E |
| B0393.3 | *B0393.3* | -0.585 | E |
| C50D2.8 | *C50D2.8* | -0.586 | E |
| C30B5.7 | *C30B5.7* | -0.586 | E |
| F56H11.3 | *elo-7* | -0.586 | E |
| M199.1 | *srt-44* | -0.587 | E |
| F52C9.7 | *F52C9.7* | -0.587 | E |
| F56H6.5 | *gmd-2* | -0.587 | E |
| C42C1.1 | *sre-14* | -0.588 | E |
| F23C8.8 | *F23C8.8* | -0.588 | E |
| C17D12.3 | *C17D12.3* | -0.589 | E |
| K02D7.5 | *K02D7.5* | -0.589 | E |
| T07A9.9 | *T07A9.9* | -0.589 | E |
| C41D11.1 | *C41D11.1* | -0.589 | E |
| T28F2.3 | *cah-6* | -0.589 | E |
| C25G6.2 | *tsp-9* | -0.59 | E |
| E04F6.7 | *dhs-7* | -0.591 | E |
| F35C11.6 | *F35C11.6* | -0.591 | E |
| ZK1248.5 | *ZK1248.5* | -0.591 | E |
| Y17G7B.11 | *Y17G7B.11* | -0.592 | E |
| C18E9.2 | *C18E9.2* | -0.592 | E |
| F20D12.5 | *exc-9* | -0.593 | E |
| ZK593.3 | *ZK593.3* | -0.594 | E |
| R05G9.3 | *R05G9.3* | -0.594 | E |
| R53.8 | *R53.8* | -0.595 | E |
| C46F9.3 | *math-24* | -0.596 | E |
| T20D3.1 | *clec-183* | -0.598 | E |
| F55C5.3 | *twk-24* | -0.599 | E |
| Y11D7A.12 | *flh-1* | -0.6 | E |
| ZK1127.4 | *ZK1127.4* | -0.601 | E |
| B0035.14 | *dnj-1* | -0.604 | E |
| F46H5.3 | *F46H5.3* | -0.604 | E |
| F55G1.12 | *F55G1.12* | -0.605 | E |
| Y17G7B.19 | *Y17G7B.19* | -0.605 | E |
| F08D12.8 | *fbxb-105* | -0.605 | E |
| F56F11.5 | *F56F11.5* | -0.605 | E |
| F31E8.2 | *snt-1* | -0.607 | E |
| W09H1.1 | *W09H1.1* | -0.61 | E |
| Y38E10A.18 | *nhr-234* | -0.611 | E |
| F59E11.5 | *F59E11.5* | -0.613 | E |
| C04G6.4 | *C04G6.4* | -0.614 | E |
| R12E2.15 | *R12E2.15* | -0.614 | E |
| T17A3.4 | *fbxb-82* | -0.616 | E |
| C30B5.4 | *C30B5.4* | -0.617 | E |
| M01E5.4 | *M01E5.4* | -0.617 | E |
| F56H11.4 | *elo-1* | -0.618 | E |
| F23C8.3 | *F23C8.3* | -0.618 | E |
| C50B8.4 | *C50B8.4* | -0.619 | E |
| F36D1.1 | *F36D1.1* | -0.619 | E |
| Y48G9A.3 | *Y48G9A.3* | -0.619 | E |
| ZC239.7 | *gcy-15* | -0.62 | E |
| E04D5.4 | *E04D5.4* | -0.622 | E |
| F56E3.3 | *klp-4* | -0.622 | E |
| C44H9.8 | *C44H9.8* | -0.622 | E |
| Y48A6B.2 | *Y48A6B.2* | -0.622 | E |
| C13B9.1 | *C13B9.1* | -0.623 | E |
| C18C4.10 | *klc-2* | -0.623 | E |
| C16A3.2 | *C16A3.2* | -0.624 | E |
| F35E2.2 | *F35E2.2* | -0.624 | E |
| C27F2.4 | *C27F2.4* | -0.629 | E |
| R01H2.3 | *egg-2* | -0.63 | E |
| M01G12.12 | *rrf-2* | -0.631 | E |
| F10E7.4 | *spon-1* | -0.631 | E |
| Y54G9A.7 | *Y54G9A.7* | -0.632 | E |
| F41E7.4 | *fip-5* | -0.632 | E |
| Y55F3C.5 | *clec-164* | -0.633 | E |
| T16H12.1 | *T16H12.1* | -0.638 | E |
| C44B12.2 | *ost-1* | -0.638 | E |
| F22B3.4 | *F22B3.4* | -0.64 | E |
| F36A4.2 | *F36A4.2* | -0.64 | E |
| F52H3.3 | *bath-38* | -0.644 | E |
| F53G2.4 | *pqn-42* | -0.648 | E |
| F56C9.6 | *F56C9.6* | -0.648 | E |
| F11G11.10 | *col-17* | -0.649 | E |
| F15A4.11 | *tag-281* | -0.649 | E |
| F57G9.1 | *sre-31* | -0.65 | E |
| Y48E1B.8 | *Y48E1B.8* | -0.651 | E |
| Y40B1B.5 | *Y40B1B.5* | -0.654 | E |
| F23C8.7 | *F23C8.7* | -0.654 | E |
| C17F4.2 | *C17F4.2* | -0.655 | E |
| F47G6.2 | *F47G6.2* | -0.657 | E |
| T03F1.7 | *T03F1.7* | -0.658 | E |
| F59A3.8 | *F59A3.8* | -0.658 | E |
| C09F5.1 | *C09F5.1* | -0.659 | E |
| K07H8.1 | *K07H8.1* | -0.66 | E |
| C50F4.8 | *C50F4.8* | -0.66 | E |
| R52.1 | *sdz-28* | -0.663 | E |
| F14F11.1 | *F14F11.1* | -0.663 | E |
| F13H6.3 | *F13H6.3* | -0.663 | E |
| C38C10.4 | *gpr-2* | -0.663 | E |
| R166.4 | *pro-1* | -0.664 | E |
| T01D3.2 | *T01D3.2* | -0.664 | E |
| B0403.2 | *ubc-17* | -0.664 | E |
| W05B2.7 | *W05B2.7* | -0.664 | E |
| F58A6.5 | *F58A6.5* | -0.667 | E |
| Y54G9A.4 | *Y54G9A.4* | -0.668 | E |
| F45E10.2 | *F45E10.2* | -0.669 | E |
| ZK418.8 | *ZK418.8* | -0.669 | E |
| ZK1010.4 | *ZK1010.4* | -0.669 | E |
| Y57G11C.24 | *eps-8* | -0.67 | E |
| C29E4.5 | *tag-250* | -0.67 | E |
| C07F11.1 | *tol-1* | -0.671 | E |
| F57C2.5 | *F57C2.5* | -0.672 | E |
| ZK616.1 | *ZK616.1* | -0.676 | E |
| F56C11.3 | *F56C11.3* | -0.677 | E |
| F43E2.3 | *insc-1* | -0.678 | E |
| F46C3.1 | *pek-1* | -0.679 | E |
| F26F2.6 | *clec-263* | -0.679 | E |
| K04G7.4 | *nuo-4* | -0.683 | E |
| F32B6.1 | *nhr-4* | -0.684 | E |
| C15H11.9 | *C15H11.9* | -0.685 | E |
| F22G12.2 | *F22G12.2* | -0.686 | E |
| F31E8.4 | *F31E8.4* | -0.69 | E |
| W05H12.1 | *W05H12.1* | -0.692 | E |
| R74.6 | *R74.6* | -0.692 | E |
| ZK512.6 | *eat-4* | -0.695 | E |
| T16G12.4 | *T16G12.4* | -0.695 | E |
| C36E8.3 | *pxd-1* | -0.695 | E |
| C54C8.9 | *nlp-39* | -0.696 | E |
| ZK616.3 | *ZK616.3* | -0.7 | E |
| R13F6.5 | *R13F6.5* | -0.701 | E |
| Y47D3A.2 | *fbxa-128* | -0.702 | E |
| ZK616.7 | *ZK616.7* | -0.703 | E |
| Y54G11A.8 | *ddl-3* | -0.705 | E |
| T22E5.5 | *mup-2* | -0.707 | E |
| F23B12.8 | *bmk-1* | -0.707 | E |
| Y52B11C.1 | *Y52B11C.1* | -0.711 | E |
| ZK353.9 | *ZK353.9* | -0.711 | E |
| B0207.12 | *B0207.12* | -0.713 | E |
| ZK637.5 | *asna-1* | -0.713 | E |
| C09G5.6 | *bli-1* | -0.714 | E |
| C03C10.3 | *rnr-2* | -0.717 | E |
| T21B4.8 | *srh-61* | -0.721 | E |
| F02H6.6 | *F02H6.6* | -0.723 | E |
| K07A9.2 | *cmk-1* | -0.724 | E |
| C46A5.3 | *col-14* | -0.724 | E |
| T15D6.11 | *T15D6.11* | -0.725 | E |
| K02A11.3 | *K02A11.3* | -0.726 | E |
| K12H6.3 | *fut-4* | -0.727 | E |
| Y38C9A.2 | *cgp-1* | -0.733 | E |
| Y34D9B.1 | *mig-1* | -0.734 | E |
| F52H3.6 | *F52H3.6* | -0.734 | E |
| C33H5.8 | *C33H5.8* | -0.739 | E |
| M01A10.5 | *M01A10.5* | -0.739 | E |
| T05A1.5 | *T05A1.5* | -0.74 | E |
| C34E10.4 | *wrs-2* | -0.744 | E |
| F42A10.3 | *F42A10.3* | -0.745 | E |
| F13B10.1 | *tir-1* | -0.746 | E |
| C16C10.2 | *C16C10.2* | -0.746 | E |
| C26D10.5 | *eff-1* | -0.747 | E |
| T24C4.2 | *T24C4.2* | -0.749 | E |
| Y73F4A.3 | *Y73F4A.3* | -0.75 | E |
| T22F3.3 | *T22F3.3* | -0.75 | E |
| Y65B4BR.6 | *grl-16* | -0.75 | E |
| F09C3.4 | *fbxa-103* | -0.751 | E |
| D1081.5 | *D1081.5* | -0.753 | E |
| C48B4.10 | *C48B4.10* | -0.758 | E |
| C04F1.1 | *C04F1.1* | -0.758 | E |
| ZK678.5 | *wrt-4* | -0.759 | E |
| C01B12.5 | *C01B12.5* | -0.761 | E |
| B0228.8 | *B0228.8* | -0.764 | E |
| C34F11.5 | *C34F11.5* | -0.768 | E |
| T19C4.6 | *gpa-1* | -0.769 | E |
| F28D9.2 | *sri-5* | -0.769 | E |
| T24H7.3 | *T24H7.3* | -0.771 | E |
| B0334.11 | *B0334.11* | -0.773 | E |
| Y50D7A.7 | *ads-1* | -0.776 | E |
| F46A9.2 | *F46A9.2* | -0.776 | E |
| K12D12.3 | *col-84* | -0.78 | E |
| F16A11.1 | *F16A11.1* | -0.786 | E |
| Y56A3A.14 | *sdz-33* | -0.791 | E |
| F33E2.5 | *F33E2.5* | -0.793 | E |
| B0207.11 | *B0207.11* | -0.795 | E |
| R06F6.4 | *set-14* | -0.803 | E |
| F54F2.5 | *ztf-1* | -0.803 | E |
| T02H6.5 | *T02H6.5* | -0.805 | E |
| Y55F3AM.11 | *Y55F3AM.11* | -0.807 | E |
| F26H9.1 | *prom-1* | -0.813 | E |
| R52.2 | *R52.2* | -0.814 | E |
| R12B2.2 | *R12B2.2* | -0.816 | E |
| T06A4.3 | *T06A4.3* | -0.822 | E |
| K08C7.6 | *K08C7.6* | -0.826 | E |
| F56D5.10 | *srxa-2* | -0.827 | E |
| E02H1.3 | *tag-124* | -0.828 | E |
| C06A5.8 | *C06A5.8* | -0.828 | E |
| C53B4.6 | *C53B4.6* | -0.829 | E |
| T10D4.7 | *T10D4.7* | -0.829 | E |
| E02H1.4 | *pme-2* | -0.832 | E |
| Y73F8A.6 | *ccg-1* | -0.832 | E |
| Y39A1C.1 | *Y39A1C.1* | -0.838 | E |
| C47E12.11 | *C47E12.11* | -0.841 | E |
| ZK688.8 | *gly-3* | -0.842 | E |
| R01H10.4 | *R01H10.4* | -0.844 | E |
| D1054.14 | *D1054.14* | -0.845 | E |
| W01B11.4 | *W01B11.4* | -0.861 | E |
| K11D2.3 | *unc-101* | -0.87 | E |
| M110.5 | *dab-1* | -0.895 | E |
| Y71F9B.14 | *Y71F9B.14* | -0.91 | E |
| R05A10.5 | *R05A10.5* | -0.914 | E |

.
