# Supplementary material for: Large-scale functional RNAi screen in C. elegans identifies genes that regulate the dysfunction of mutant polyglutamine neurons
Source: BMC Genomics. 2012 Mar 13;13:91. doi: 10.1186/1471-2164-13-91 (PMC3331833; doi:10.1186/1471-2164-13-91)
Supplement: Additional file 4 — Table S3. List of the 662 genes that modified 128Q-neuron dysfunction when knocked-down by RNAi in the secondary screen. [file 1471-2164-13-91-S4.DOC]

**Supplementary Table 3.** List of the 662 genes that modify 128Q-neuron dysfunction when knocked-down by RNAi.

Genes are ranked by decreasing S-score values. S means suppression of 128Q-neuron dysfunction when knocked-down by RNAi, whereas E means enhancement of 128Q-neuron dysfunction. The maximally-achievable score is 4.55 (100% response to touch) and the smallest score is -1 (complete loss of touch response). Conservation in humans (best orthologs) is also indicated as inferred from InParanoid clusters.

| **Gene ID** | **Gene name** | **S score** | **Effect** | **Conserved in homo sapiens** | **Human best ortholog** |
| --- | --- | --- | --- | --- | --- |
| Y74C9A.2 | *nlp-40* | 3.6464 | S | NO |  |
| C13G5.2 | *C13G5.2* | 2.2048 | S | NO |  |
| F56H11.1 | *fbl-1* | 2.0035 | S | YES | FBLN1 |
| C17E4.3 | *C17E4.3* | 1.9818 | S | NO |  |
| K07C5.4 | *K07C5.4* | 1.9792 | S | YES | NOP56 |
| W02D3.1 | *W02D3.1* | 1.9314 | S | NO |  |
| ZK856.12 | *ZK856.12* | 1.9098 | S | NO |  |
| F41F3.3 | *F41F3.3* | 1.8568 | S | NO |  |
| T21C12.1 | *unc-49* | 1.8566 | S | NO |  |
| C04E7.2 | *sor-3* | 1.8500 | S | NO |  |
| F23H12.2 | *F23H12.2* | 1.8441 | S | YES | TOMM20 |
| F58A4.8 | *tbg-1* | 1.8322 | S | YES | TUBG1 |
| F39C12.2 | *add-1* | 1.8149 | S | YES | ADD1 |
| K10F12.4 | *gsto-3* | 1.7657 | S | NO |  |
| F38A6.1 | *pha-4* | 1.7250 | S | NO |  |
| Y102E9.3 | *Y102E9.3* | 1.6921 | S | NO |  |
| C27B7.8 | *rap-1* | 1.6578 | S | YES | RAP1B |
| T07D1.2 | *T07D1.2* | 1.6368 | S | YES | RMND5A |
| Y18D10A.5 | *gsk-3* | 1.6322 | S | YES | GSK3B |
| F40F12.4 | *F40F12.4* | 1.6317 | S | NO |  |
| C31H5.4 | *C31H5.4* | 1.6063 | S | NO |  |
| Y111B2A.22 | *ssl-1* | 1.5936 | S | YES | SRCAP |
| Y102A5C.1 | *fbxa-206* | 1.5784 | S | NO |  |
| Y79H2A.1 | *brp-1* | 1.5781 | S | NO |  |
| T13F2.7 | *sna-2* | 1.5774 | S | NO |  |
| Y66A7A.6 | *gly-8* | 1.5745 | S | NO |  |
| F55F8.2 | *F55F8.2* | 1.5460 | S | YES | DDX24 |
| T28D9.10 | *snr-3* | 1.5043 | S | YES | SNRPD1 |
| F26E4.8 | *tba-1* | 1.5004 | S | NO |  |
| Y49F6B.1 | *cyh-1* | 1.4720 | S | YES | CCNH |
| C32D5.9 | *lgg-1* | 1.4583 | S | YES | GABARAP |
| B0218.3 | *pmk-1* | 1.4563 | S | YES | MAPK14 |
| K08F8.4 | *pah-1* | 1.4514 | S | YES | PAH |
| C50D2.2 | *C50D2.2* | 1.4283 | S | YES | SLC7A4 |
| K10B3.8 | *gpd-2* | 1.4252 | S | YES | GAPDH |
| ZC204.2 | *ZC204.2* | 1.4184 | S | NO |  |
| T02C12.1 | *hum-5* | 1.4144 | S | YES | MYO1D |
| F22B7.9 | *F22B7.9* | 1.4134 | S | NO |  |
| F40F8.9 | *lsm-1* | 1.4093 | S | YES | LSM1 |
| C37A2.7 | *C37A2.7* | 1.4092 | S | YES | RPLP2 |
| M01F1.1 | *gly-14* | 1.4092 | S | YES | MGAT1 |
| H17B01.1 | *H17B01.1* | 1.3921 | S | YES | SLC2A3 |
| Y69F12A.1 | *Y69F12A.1* | 1.3719 | S | NO |  |
| T19B4.3 | *T19B4.3* | 1.3694 | S | YES | APRT |
| Y40B10A.6 | *Y40B10A.6* | 1.3504 | S | YES | COMTD1 |
| Y51B9A.6 | *Y51B9A.6* | 1.3501 | S | NO |  |
| C29F9.5 | *C29F9.5* | 1.3462 | S | NO |  |
| Y54G2A.16 | *Y54G2A.16* | 1.3420 | S | NO |  |
| Y39B6A.2 | *pph-5* | 1.3245 | S | YES | PPP5C |
| F35G12.5 | *F35G12.5* | 1.3217 | S | NO |  |
| ZK430.3 | *sod-5* | 1.3216 | S | YES | SOD1 |
| C34E10.7 | *cnd-1* | 1.3209 | S | YES | NEUROD4 |
| H21P03.1 | *mbf-1* | 1.3208 | S | YES | EDF1 |
| Y57G11C.23 | *Y57G11C.23* | 1.3132 | S | NO |  |
| K02E11.5 | *K02E11.5* | 1.3067 | S | NO |  |
| R52.8 | *math-36* | 1.2996 | S | NO |  |
| F25F8.2 | *glc-2* | 1.2938 | S | NO |  |
| R02F2.1 | *R02F2.1* | 1.2672 | S | YES | CCNI |
| Y47D3A.23 | *gly-9* | 1.2629 | S | NO |  |
| Y116F11B.12 | *gly-4* | 1.2609 | S | YES | GALNT2 |
| Y37D8A.19 | *Y37D8A.19* | 1.2519 | S | NO |  |
| W07E6.2 | *W07E6.2* | 1.2509 | S | YES | NLE1 |
| R03E9.1 | *mdl-1* | 1.2436 | S | NO |  |
| Y48B6A.8 | *ace-3* | 1.2430 | S | NO |  |
| ZK652.2 | *tomm-7* | 1.2418 | S | NO |  |
| K08E3.5 | *K08E3.5* | 1.2405 | S | YES | UGP2 |
| Y51A2D.11 | *ttr-26* | 1.2363 | S | NO |  |
| ZK829.6 | *tgt-1* | 1.2304 | S | YES | QTRT1 |
| F25B5.5 | *F25B5.5* | 1.2301 | S | YES | CDK5RAP1 |
| K01G5.2 | *hpl-2* | 1.2156 | S | NO |  |
| C28A5.1 | *C28A5.1* | 1.2140 | S | NO |  |
| C54D10.3 | *C54D10.3* | 1.2120 | S | NO |  |
| C08E3.4 | *fbxa-161* | 1.1950 | S | NO |  |
| R02F2.8 | *R02F2.8* | 1.1912 | S | NO |  |
| C53D6.2 | *unc-129* | 1.1884 | S | YES | GDF10 |
| F14F7.2 | *cyp-13A11* | 1.1837 | S | YES | CYP3A5 |
| K08E7.1 | *K08E7.1* | 1.1800 | S | NO |  |
| F52C9.5 | *F52C9.5* | 1.1798 | S | NO |  |
| B0035.6 | *B0035.6* | 1.1764 | S | NO |  |
| C16C10.10 | *glod-4* | 1.1685 | S | YES | GLOD4 |
| F10E9.7 | *F10E9.7* | 1.1658 | S | NO |  |
| C03B8.3 | *C03B8.3* | 1.1513 | S | NO |  |
| C34B2.4 | *C34B2.4* | 1.1458 | S | YES | LMO4 |
| C17H12.8 | *C17H12.8* | 1.1446 | S | NO |  |
| F09G8.6 | *col-91* | 1.1416 | S | YES | SFTPD |
| K01G5.5 | *K01G5.5* | 1.1408 | S | YES | ENSP00000377168 |
| B0361.7 | *B0361.7* | 1.1376 | S | YES | ACP2 |
| ZK1290.6 | *rnh-1.1* | 1.1355 | S | NO |  |
| F47C10.2 | *btb-21* | 1.1335 | S | NO |  |
| F53E10.4 | *F53E10.4* | 1.1300 | S | NO |  |
| Y51H7C.2 | *scl-17* | 1.1277 | S | YES | CRISP3 |
| Y105C5A.10 | *Y105C5A.10* | 1.1273 | S | NO |  |
| T03F6.1 | *qdpr-1* | 1.1246 | S | YES | QDPR |
| F38H4.8 | *ech-2* | 1.1164 | S | YES | ECHDC3 |
| C56C10.9 | *C56C10.9* | 1.1129 | S | YES | SDF4 |
| B0524.2 | *B0524.2* | 1.1117 | S | YES | PNPLA4 |
| Y39E4B.9 | *bre-2* | 1.1111 | S | NO |  |
| C36B1.11 | *C36B1.11* | 1.1104 | S | NO |  |
| F58H7.5 | *F58H7.5* | 1.1020 | S | NO |  |
| C10H11.3 | *ugt-25* | 1.0924 | S | YES | UGT3A2 |
| H35N09.1 | *H35N09.1* | 1.0905 | S | NO |  |
| F27C1.4 | *F27C1.4* | 1.0783 | S | NO |  |
| Y39E4B.4 | *tsp-3* | 1.0777 | S | NO |  |
| F56A8.7 | *unc-64* | 1.0637 | S | YES | STX1A |
| Y59A8A.3 | *Y59A8A.3* | 1.0536 | S | NO |  |
| K07H8.2 | *K07H8.2* | 1.0514 | S | YES | SLC41A1 |
| F59H5.3 | *bath-12* | 1.0504 | S | NO |  |
| W02D9.5 | *W02D9.5* | 1.0478 | S | NO |  |
| F20H11.5 | *F20H11.5* | 1.0388 | S | NO |  |
| C25D7.3 | *sdc-3* | 1.0353 | S | NO |  |
| F54D5.9 | *F54D5.9* | 1.0297 | S | NO |  |
| T05E7.3 | *T05E7.3* | 1.0286 | S | YES | ENSP00000367223 |
| F10E9.4 | *F10E9.4* | 1.0245 | S | NO |  |
| C25A8.1 | *C25A8.1* | 1.0233 | S | NO |  |
| ZC395.10 | *ZC395.10* | 1.0206 | S | YES | PTGES3 |
| F54D10.7 | *F54D10.7* | 1.0192 | S | NO |  |
| W06E11.1 | *W06E11.1* | 1.0153 | S | YES | POLR3E |
| C56A3.5 | *C56A3.5* | 1.0131 | S | NO |  |
| ZC15.8 | *pqn-94* | 1.0101 | S | NO |  |
| F36F2.1 | *F36F2.1* | 1.0076 | S | NO |  |
| C08F8.5 | *fbxb-9* | 1.0063 | S | NO |  |
| Y40B10A.2 | *Y40B10A.2* | 1.0054 | S | YES | COMTD1 |
| F52B5.6 | *rpl-25.2* | 1.0051 | S | YES | RPL23A |
| H14E04.2 | *H14E04.2* | 1.0020 | S | NO |  |
| F10E9.2 | *F10E9.2* | 1.0017 | S | NO |  |
| F13G3.4 | *dylt-1* | 0.9991 | S | YES | DYNLT1 |
| VW02B12L.3 | *ebp-2* | 0.9942 | S | NO |  |
| F28C12.5 | *sra-21* | 0.9925 | S | NO |  |
| K03A1.5 | *sur-5* | 0.9870 | S | YES | AACS |
| T12B5.2 | *fbxa-54* | 0.9802 | S | NO |  |
| Y51A2D.9 | *ttr-24* | 0.9798 | S | NO |  |
| C39B5.7 | *fbxa-12* | 0.9796 | S | NO |  |
| D1037.3 | *ftn-2* | 0.9793 | S | YES | FTH1 |
| C03C11.2 | *fog-3* | 0.9729 | S | YES | BTG1 |
| F43C9.4 | *mig-13* | 0.9687 | S | NO |  |
| F53A3.3 | *rps-22* | 0.9624 | S | YES | RPS15A |
| Y47D3A.6 | *tra-1* | 0.9530 | S | NO |  |
| Y47D3B.2 | *nlp-21* | 0.9504 | S | NO |  |
| W02D9.2 | *W02D9.2* | 0.9469 | S | YES | ENSP00000363774 |
| T24B8.5 | *T24B8.5* | 0.9465 | S | NO |  |
| H10D18.2 | *scl-12* | 0.9443 | S | YES | CRISP3 |
| B0304.1 | *B0304.1* | 0.9436 | S | YES | MYOG |
| C06C6.9 | *C06C6.9* | 0.9436 | S | NO |  |
| T23G7.2 | *T23G7.2* | 0.9396 | S | NO |  |
| T07A9.5 | *eri-1* | 0.9368 | S | NO |  |
| C36B1.1 | *cle-1* | 0.9297 | S | YES | COL15A1 |
| F43C1.2 | *mpk-1* | 0.9257 | S | YES | MAPK1 |
| F29G9.1 | *F29G9.1* | 0.9220 | S | NO |  |
| T10H9.3 | *T10H9.3* | 0.9194 | S | NO |  |
| VT23B5.2 | *VT23B5.2* | 0.9187 | S | NO |  |
| F11C1.2 | *F11C1.2* | 0.9104 | S | NO |  |
| F40F8.1 | *F40F8.1* | 0.9020 | S | YES | CMPK1 |
| Y43F8C.9 | *Y43F8C.9* | 0.9018 | S | NO |  |
| K11H3.5 | *K11H3.5* | 0.8999 | S | NO |  |
| T05H10.3 | *T05H10.3* | 0.8894 | S | NO |  |
| Y38E10A.3 | *Y38E10A.3* | 0.8838 | S | NO |  |
| T28D6.3 | *T28D6.3* | 0.8802 | S | NO |  |
| C50F4.6 | *C50F4.6* | 0.8798 | S | NO |  |
| W06A11.1 | *W06A11.1* | 0.8777 | S | NO |  |
| F57C9.4 | *F57C9.4* | 0.8745 | S | NO |  |
| D2045.6 | *cul-1* | 0.8734 | S | YES | CUL1 |
| C46E10.7 | *srh-99* | 0.8707 | S | NO |  |
| Y49F6C.1 | *bath-8* | 0.8698 | S | NO |  |
| F48C1.4 | *F48C1.4* | 0.8686 | S | NO |  |
| F42H10.7 | *F42H10.7* | 0.8612 | S | YES | DGCR14 |
| F57H12.7 | *mec-17* | 0.8612 | S | NO |  |
| T02C12.3 | *T02C12.3* | 0.8599 | S | YES | GTF3C5 |
| C53B4.7 | *bre-1* | 0.8545 | S | YES | GMDS |
| Y40B1B.8 | *Y40B1B.8* | 0.8543 | S | YES | ENSP00000296626 |
| B0035.8 | *his-48* | 0.8466 | S | YES | HIST1H2BL |
| C37A2.4 | *cye-1* | 0.8416 | S | YES | CCNE1 |
| T26C5.1 | *gst-13* | 0.8408 | S | NO |  |
| C34B2.2 | *kbp-5* | 0.8394 | S | NO |  |
| T23B12.6 | *T23B12.6* | 0.8348 | S | YES | KCTD3 |
| C25A1.11 | *aha-1* | 0.8332 | S | YES | ARNTL |
| B0252.6 | *B0252.6* | 0.8301 | S | NO |  |
| C35D10.10 | *C35D10.10* | 0.8294 | S | YES | ENSP00000367563 |
| F55C7.2 | *F55C7.2* | 0.8284 | S | NO |  |
| W09D10.3 | *W09D10.3* | 0.8248 | S | YES | SLC25A10 |
| R09B3.2 | *R09B3.2* | 0.8182 | S | YES | ENSP00000314624 |
| F42G10.1 | *F42G10.1* | 0.8177 | S | NO |  |
| C09G1.1 | *pqn-11* | 0.8167 | S | NO |  |
| C45B11.3 | *dhs-18* | 0.8167 | S | YES | HSDL2 |
| F53C3.5 | *F53C3.5* | 0.8044 | S | NO |  |
| C25A11.4 | *ajm-1* | 0.8033 | S | NO |  |
| Y39A1A.3 | *Y39A1A.3* | 0.8017 | S | YES | SSSCA1 |
| K03D10.1 | *kal-1* | 0.8015 | S | YES | KAL1 |
| F41B5.4 | *cyp-33C3* | 0.8012 | S | YES | CYP2C8 |
| T22C8.5 | *sptf-2* | 0.7980 | S | YES | KLF9 |
| W06E11.4 | *W06E11.4* | 0.7968 | S | YES | SBDS |
| K08C7.4 | *K08C7.4* | 0.7913 | S | NO |  |
| T12F5.1 | *T12F5.1* | 0.7905 | S | NO |  |
| Y41G9A.4 | *Y41G9A.4* | 0.7895 | S | NO |  |
| B0281.5 | *B0281.5* | 0.7889 | S | YES | KCNRG |
| C51E3.6 | *C51E3.6* | 0.7818 | S | YES | ENSP00000368628 |
| C17G10.5 | *lys-8* | 0.7785 | S | NO |  |
| ZK637.8 | *unc-32* | 0.7767 | S | YES | ATP6V0A1 |
| F36H12.13 | *F36H12.13* | 0.7748 | S | NO |  |
| D2007.2 | *D2007.2* | 0.7738 | S | NO |  |
| R12E2.14 | *R12E2.14* | 0.7729 | S | NO |  |
| C01G5.8 | *C01G5.8* | 0.7592 | S | YES | FAN1 |
| R06A4.9 | *R06A4.9* | 0.7538 | S | YES | WDR33 |
| Y49E10.1 | *rpt-6* | 0.7525 | S | YES | PSMC5 |
| C10C5.4 | *C10C5.4* | 0.7494 | S | YES | ACY1 |
| F17C11.4 | *F17C11.4* | 0.7490 | S | NO |  |
| T04D1.3 | *unc-57* | 0.7455 | S | YES | SH3GL1 |
| Y42H9AR.3 | *rabs-5* | 0.7454 | S | YES | ZFYVE20 |
| Y57G11B.5 | *Y57G11B.5* | 0.7379 | S | NO |  |
| F32A5.4 | *F32A5.4* | 0.7349 | S | NO |  |
| C05D11.5 | *C05D11.5* | 0.7299 | S | YES | HYI |
| ZK673.7 | *tnc-2* | 0.7287 | S | NO |  |
| C03B8.1 | *C03B8.1* | 0.7279 | S | NO |  |
| T19C3.8 | *fem-2* | 0.7230 | S | YES | PPM1F |
| ZK512.7 | *ZK512.7* | 0.7211 | S | NO |  |
| ZK384.2 | *scl-20* | 0.7193 | S | YES | CRISP3 |
| B0412.2 | *daf-7* | 0.7185 | S | YES | GDF11 |
| F08B6.2 | *gpc-2* | 0.7111 | S | YES | GNG13 |
| F35B12.6 | *tag-290* | 0.7081 | S | YES | TFPI2 |
| F58E1.6 | *nhx-6* | 0.7078 | S | NO |  |
| T20B5.3 | *oga-1* | 0.7031 | S | YES | MGEA5 |
| ZK652.5 | *ceh-23* | 0.7003 | S | NO |  |
| T22G5.2 | *lbp-7* | 0.6974 | S | YES | FABP3 |
| W05B2.1 | *col-94* | 0.6963 | S | YES | SFTPD |
| R05F9.10 | *sgt-1* | 0.6960 | S | YES | SGTA |
| T20B5.2 | *T20B5.2* | 0.6916 | S | NO |  |
| Y22D7AR.6 | *Y22D7AR.6* | 0.6881 | S | NO |  |
| F54C8.2 | *cpar-1* | 0.6842 | S | NO |  |
| T05H10.8 | *T05H10.8* | 0.6803 | S | NO |  |
| C56C10.10 | *C56C10.10* | 0.6789 | S | YES | AIP |
| F53A2.5 | *dro-1* | 0.6777 | S | YES | DR1 |
| F44D12.8 | *F44D12.8* | 0.6712 | S | NO |  |
| T21C9.8 | *ttr-23* | 0.6689 | S | NO |  |
| Y47D3B.7 | *sbp-1* | 0.6631 | S | YES | SREBF2 |
| F22D3.1 | *ceh-38* | 0.6578 | S | NO |  |
| C10C5.5 | *C10C5.5* | 0.6457 | S | YES | ACY1 |
| F17C8.2 | *col-89* | 0.6430 | S | YES | SFTPD |
| T08G2.3 | *T08G2.3* | 0.6412 | S | YES | ACADM |
| F07F6.5 | *dct-5* | 0.6400 | S | NO |  |
| W03D2.6 | *W03D2.6* | 0.6400 | S | NO |  |
| Y57A10A.28 | *Y57A10A.28* | 0.6393 | S | YES | TMEM38B |
| Y53H1B.2 | *Y53H1B.2* | 0.6387 | S | NO |  |
| K02B9.4 | *elt-3* | 0.6376 | S | NO |  |
| T27F2.1 | *skp-1* | 0.6368 | S | YES | SNW1 |
| F02H6.3 | *F02H6.3* | 0.6327 | S | NO |  |
| ZK337.2 | *ZK337.2* | 0.6312 | S | YES | ZSCAN5B |
| B0348.6 | *ife-3* | 0.6309 | S | YES | ENSP00000378376 |
| ZK1251.6 | *msp-76* | 0.6309 | S | NO |  |
| F15E6.3 | *F15E6.3* | 0.6302 | S | NO |  |
| K02D10.5 | *K02D10.5* | 0.6302 | S | YES | SNAP29 |
| LLC1.2 | *LLC1.2* | 0.6275 | S | NO |  |
| K08F4.1 | *K08F4.1* | 0.6272 | S | YES | CHTF18 |
| F42G9.7 | *snt-2* | 0.6259 | S | NO |  |
| C34F11.8 | *C34F11.8* | 0.6254 | S | NO |  |
| T22F3.4 | *rpl-11.1* | 0.6223 | S | YES | RPL11 |
| K04A8.5 | *K04A8.5* | 0.6114 | S | YES | LIPF |
| F14F3.1 | *vab-3* | 0.6063 | S | YES | PAX6 |
| F31C3.6 | *F31C3.6* | 0.6023 | S | NO |  |
| F11A5.12 | *stdh-2* | 0.5896 | S | YES | HSD17B12 |
| Y38F1A.10 | *max-2* | 0.5891 | S | NO |  |
| F58E2.4 | *F58E2.4* | 0.5880 | S | NO |  |
| T09B4.5 | *T09B4.5* | 0.5878 | S | NO |  |
| T03F1.11 | *T03F1.11* | 0.5850 | S | NO |  |
| R12B2.5 | *mdt-15* | 0.5828 | S | YES | MED15 |
| Y51A2B.1 | *Y51A2B.1* | 0.5825 | S | NO |  |
| C10G11.5 | *pnk-1* | 0.5776 | S | YES | PANK1 |
| F40F4.3 | *lbp-1* | 0.5728 | S | NO |  |
| F22B5.1 | *evl-20* | 0.5714 | S | YES | ARL2 |
| ZK1236.1 | *ZK1236.1* | 0.5712 | S | YES | GUF1 |
| C18A3.1 | *C18A3.1* | 0.5693 | S | NO |  |
| Y106G6H.9 | *Y106G6H.9* | 0.5632 | S | NO |  |
| Y38E10A.14 | *Y38E10A.14* | 0.5628 | S | NO |  |
| ZK520.4 | *cul-2* | 0.5608 | S | YES | CUL2 |
| F59B1.8 | *F59B1.8* | 0.5602 | S | NO |  |
| C07B5.5 | *nuc-1* | 0.5544 | S | NO |  |
| K01A2.5 | *K01A2.5* | 0.5541 | S | YES | BPHL |
| ZK353.6 | *lap-1* | 0.5520 | S | YES | NPEPL1 |
| Y11D7A.4 | *rab-28* | 0.5515 | S | YES | RAB28 |
| C10A4.8 | *mnm-2* | 0.5467 | S | NO |  |
| ZK550.6 | *ZK550.6* | 0.5451 | S | YES | ENSP00000367928 |
| F56G4.3 | *pes-2.2* | 0.5442 | S | NO |  |
| Y39E4A.3 | *Y39E4A.3* | 0.5441 | S | YES | BCKDHA |
| Y71F9AM.5 | *nxt-1* | 0.5414 | S | YES | NXT2 |
| B0547.1 | *csn-5* | 0.5356 | S | YES | COPS5 |
| W05H7.3 | *sedl-1* | 0.5351 | S | YES | TRAPPC2 |
| F28D1.5 | *thn-2* | 0.5328 | S | NO |  |
| Y22F5A.3 | *ric-4* | 0.5322 | S | YES | SNAP25 |
| R07B1.1 | *vab-15* | 0.5296 | S | YES | MSX2 |
| ZK1127.10 | *ZK1127.10* | 0.5296 | S | YES | CTH |
| F11A10.1 | *lex-1* | 0.5296 | S | YES | ATAD2B |
| Y65B4BR.5 | *Y65B4BR.5* | 0.5242 | S | YES | NACA |
| Y106G6H.7 | *sec-8* | 0.5228 | S | YES | EXOC4 |
| F38A5.10 | *nspb-4* | 0.5138 | S | NO |  |
| R07E5.2 | *prdx-3* | 0.5137 | S | YES | PRDX3 |
| W05H9.1 | *W05H9.1* | 0.5137 | S | NO |  |
| F29B9.6 | *ubc-9* | 0.5105 | S | YES | UBE2I |
| C39D10.7 | *C39D10.7* | 0.5092 | S | NO |  |
| ZK512.8 | *ZK512.8* | 0.5049 | S | NO |  |
| F08B12.4 | *F08B12.4* | 0.4985 | S | NO |  |
| Y57E12AL.5 | *mdt-6* | 0.4983 | S | YES | MED6 |
| F17E5.1 | *lin-2* | 0.4941 | S | YES | CASK |
| F22F1.1 | *hil-3* | 0.4910 | S | YES | HIST1H1D |
| F36A4.5 | *F36A4.5* | 0.4906 | S | NO |  |
| T16A9.1 | *T16A9.1* | 0.4890 | S | NO |  |
| F53H4.4 | *F53H4.4* | 0.4872 | S | NO |  |
| D1054.10 | *D1054.10* | 0.4865 | S | NO |  |
| T25G3.1 | *T25G3.1* | 0.4830 | S | YES | KIAA1143 |
| C09G9.7 | *C09G9.7* | 0.4825 | S | NO |  |
| T06D4.1 | *T06D4.1* | 0.4819 | S | NO |  |
| F25B4.4 | *F25B4.4* | 0.4819 | S | NO |  |
| F43G9.5 | *F43G9.5* | 0.4783 | S | YES | NUDT21 |
| B0478.1 | *jnk-1* | 0.4751 | S | YES | MAPK10 |
| ZK131.7 | *his-13* | 0.4751 | S | YES | HIST1H3H |
| M18.2 | *dlc-2* | 0.4741 | S | NO |  |
| K09E4.4 | *K09E4.4* | 0.4730 | S | YES | NAGLU |
| C40C9.5 | *nlg-1* | 0.4712 | S | NO |  |
| T17H7.1 | *T17H7.1* | 0.4692 | S | NO |  |
| F32D8.6 | *emo-1* | 0.4669 | S | YES | SEC61G |
| T23H2.5 | *rab-10* | 0.4610 | S | YES | RAB10 |
| F07H5.8 | *F07H5.8* | 0.4594 | S | NO |  |
| Y17G7B.2 | *ash-2* | 0.4594 | S | YES | ASH2L |
| T21B10.7 | *cct-2* | 0.4592 | S | YES | CCT2 |
| Y54E10A.2 | *cogc-1* | 0.4592 | S | YES | COG1 |
| F53B7.3 | *F53B7.3* | 0.4577 | S | YES | ISY1 |
| K07D4.7 | *tag-218* | 0.4568 | S | YES | ARHGEF26 |
| B0304.2 | *B0304.2* | 0.4565 | S | NO |  |
| ZK39.3 | *clec-94* | 0.4556 | S | NO |  |
| F38E11.2 | *hsp-12.6* | 0.4465 | S | YES | CRYAB |
| T04G9.4 | *T04G9.4* | 0.4465 | S | YES | AASDHPPT |
| Y43C5B.2 | *Y43C5B.2* | 0.4453 | S | NO |  |
| ZK1098.4 | *ZK1098.4* | 0.4442 | S | YES | EIF2B1 |
| F45F2.4 | *his-7* | 0.4426 | S | YES | HIST1H2AC |
| C02B8.4 | *hlh-8* | 0.4388 | S | YES | ASCL4 |
| Y34D9A.7 | *Y34D9A.7* | 0.4365 | S | YES | ZCCHC8 |
| R11.1 | *R11.1* | 0.4355 | S | YES | SLC25A21 |
| C14B1.4 | *tag-125* | 0.4342 | S | YES | WDR5 |
| T26C5.3 | *T26C5.3* | 0.4339 | S | YES | SLC33A1 |
| C01A2.5 | *C01A2.5* | 0.4297 | S | YES | C20orf43 |
| ZK673.3 | *ZK673.3* | 0.4297 | S | NO |  |
| AC3.7 | *ugt-1* | 0.4227 | S | YES | UGT3A2 |
| K02B12.2 | *K02B12.2* | 0.4217 | S | NO |  |
| C05C8.3 | *fkb-3* | 0.4215 | S | NO |  |
| F54D10.1 | *skr-15* | 0.4211 | S | NO |  |
| K08D10.3 | *rnp-3* | 0.4211 | S | YES | SNRPB2 |
| F57A10.2 | *F57A10.2* | 0.4203 | S | NO |  |
| C06B3.4 | *stdh-1* | 0.4202 | S | YES | HSD17B12 |
| T02G5.7 | *T02G5.7* | 0.4193 | S | NO |  |
| T05A6.1 | *cki-1* | 0.4134 | S | NO |  |
| H04M03.1 | *H04M03.1* | 0.4115 | S | NO |  |
| W09D6.4 | *W09D6.4* | 0.4114 | S | NO |  |
| T14F9.3 | *hex-1* | 0.4113 | S | YES | HEXB |
| Y59E9AR.4 | *thn-5* | 0.4106 | S | NO |  |
| F09B12.3 | *F09B12.3* | 0.4070 | S | NO |  |
| R07E5.3 | *R07E5.3* | 0.4056 | S | YES | SMARCB1 |
| F25H2.2 | *F25H2.2* | 0.4016 | S | YES | SNX27 |
| F28C6.10 | *F28C6.10* | 0.3999 | S | NO |  |
| R12H7.2 | *asp-4* | 0.3975 | S | YES | CTSD |
| F40G9.3 | *ubc-20* | 0.3916 | S | YES | UBE2K |
| C27C7.1 | *C27C7.1* | 0.3877 | S | NO |  |
| Y38F1A.5 | *cyd-1* | 0.3876 | S | YES | CCND1 |
| F19B6.2 | *ufd-1* | 0.3875 | S | YES | UFD1L |
| F52H3.5 | *F52H3.5* | 0.3859 | S | YES | TTC36 |
| T13F2.6 | *T13F2.6* | 0.3847 | S | NO |  |
| Y18D10A.20 | *pfn-1* | 0.3800 | S | NO |  |
| Y38H6C.1 | *dct-16* | 0.3761 | S | NO |  |
| M01E11.2 | *M01E11.2* | 0.3725 | S | YES | CTNNBL1 |
| F07F6.1 | *F07F6.1* | 0.3711 | S | NO |  |
| F25H9.6 | *F25H9.6* | 0.3698 | S | YES | PPCDC |
| F15H10.3 | *apc-10* | 0.3625 | S | NO |  |
| M02D8.4 | *M02D8.4* | 0.3625 | S | YES | ASNS |
| F56D5.5 | *F56D5.5* | 0.3613 | S | NO |  |
| W02F12.3 | *W02F12.3* | 0.3607 | S | NO |  |
| F11G11.2 | *gst-7* | 0.3566 | S | NO |  |
| T28B4.4 | *T28B4.4* | 0.3556 | S | NO |  |
| W08E3.1 | *snr-2* | 0.3548 | S | YES | SNRPB |
| F21F3.2 | *F21F3.2* | 0.3534 | S | NO |  |
| T06G6.9 | *pfd-3* | 0.3478 | S | YES | VBP1 |
| F26E4.1 | *sur-6* | 0.3470 | S | YES | PPP2R2A |
| C26B9.5 | *C26B9.5* | 0.3445 | S | YES | ENSP00000366676 |
| K07F5.11 | *ssq-1* | 0.3436 | S | YES | ENSP00000365448 |
| C44H4.6 | *C44H4.6* | 0.3434 | S | NO |  |
| ZK792.3 | *inx-9* | 0.3411 | S | NO |  |
| B0286.3 | *B0286.3* | 0.3375 | S | YES | PAICS |
| C08B6.9 | *aos-1* | 0.3352 | S | YES | SAE1 |
| Y75B12B.5 | *cyn-3* | 0.3302 | S | YES | ENSP00000348240 |
| Y39G10AL.3 | *cdk-7* | 0.3299 | S | YES | CDK7 |
| C47G2.2 | *unc-130* | 0.3275 | S | YES | FOXD4 |
| C01F1.3 | *C01F1.3* | 0.3231 | S | YES | TGDS |
| T28B8.2 | *ins-18* | 0.3214 | S | NO |  |
| C54E4.2 | *C54E4.2* | 0.3207 | S | YES | SPOCK1 |
| ZK652.11 | *cuc-1* | 0.3180 | S | YES | ATOX1 |
| T19B10.11 | *mxl-1* | 0.3178 | S | YES | MAX |
| F48E8.1 | *lon-1* | 0.3174 | S | NO |  |
| Y39A1A.24 | *Y39A1A.24* | 0.3073 | S | NO |  |
| R05F9.1 | *R05F9.1* | 0.3059 | S | YES | BTBD10 |
| F28H1.3 | *ars-2* | 0.3025 | S | YES | AARS |
| C34F11.6 | *msp-49* | 0.2950 | S | NO |  |
| W01H2.3 | *rab-37* | 0.2825 | S | YES | RAB37 |
| Y19D10B.6 | *Y19D10B.6* | 0.2821 | S | NO |  |
| F26A3.6 | *del-3* | 0.2265 | S | NO |  |
| F22D6.12 | *gly-19* | -0.1608 | E | YES | GCNT1 |
| C25A1.4 | *C25A1.4* | -0.1682 | E | YES | MYEF2 |
| C37A5.1 | *C37A5.1* | -0.1682 | E | NO |  |
| T15D6.12 | *T15D6.12* | -0.1682 | E | NO |  |
| F32H2.9 | *tba-6* | -0.2053 | E | NO |  |
| C12C8.2 | *C12C8.2* | -0.2103 | E | NO |  |
| Y106G6H.1 | *Y106G6H.1* | -0.2322 | E | NO |  |
| C24G7.1 | *C24G7.1* | -0.2735 | E | NO |  |
| Y47G6A.1 | *inx-21* | -0.2912 | E | NO |  |
| T23H2.2 | *snt-4* | -0.2956 | E | NO |  |
| C08B11.8 | *C08B11.8* | -0.2965 | E | YES | ALG6 |
| K08D8.3 | *K08D8.3* | -0.3013 | E | NO |  |
| Y18D10A.13 | *pad-1* | -0.3014 | E | YES | DOPEY1 |
| K02A6.3 | *K02A6.3* | -0.3015 | E | NO |  |
| ZK6.11 | *ZK6.11* | -0.3023 | E | NO |  |
| F58A4.4 | *pri-1* | -0.3042 | E | YES | PRIM1 |
| Y87G2A.8 | *gpi-1* | -0.3050 | E | YES | GPI |
| F57B10.11 | *bag-1* | -0.3056 | E | YES | ENSP00000224112 |
| T23B3.2 | *T23B3.2* | -0.3060 | E | NO |  |
| B0035.1 | *B0035.1* | -0.3069 | E | YES | ZNF207 |
| C09G9.6 | *oma-1* | -0.3072 | E | YES | ZFP36L2 |
| F38E11.1 | *hsp-12.3* | -0.3074 | E | YES | CRYAB |
| E02A10.1 | *E02A10.1* | -0.3083 | E | YES | MRPS5 |
| Y47G6A.8 | *crn-1* | -0.3142 | E | YES | FEN1 |
| F10G7.3 | *asf-1* | -0.3166 | E | YES | ASF1B |
| T23F6.1 | *T23F6.1* | -0.3188 | E | NO |  |
| Y41C4A.6 | *Y41C4A.6* | -0.3260 | E | NO |  |
| D1086.3 | *D1086.3* | -0.3263 | E | NO |  |
| C46H11.11 | *fhod-1* | -0.3271 | E | YES | FHOD3 |
| C13B9.1 | *C13B9.1* | -0.3286 | E | NO |  |
| F54D7.1 | *NA* | -0.3301 | E | NO |  |
| T06A4.3 | *T06A4.3* | -0.3305 | E | YES | CPA2 |
| F25H2.11 | *tct-1* | -0.3313 | E | YES | TPT1 |
| F11D5.1 | *F11D5.1* | -0.3316 | E | NO |  |
| C37H5.3 | *C37H5.3* | -0.3349 | E | YES | ABHD4 |
| Y39A3CR.4 | *ddp-1* | -0.3356 | E | YES | TIMM8A |
| ZC416.6 | *ZC416.6* | -0.3368 | E | YES | LTA4H |
| Y75B8A.35 | *zip-1* | -0.3406 | E | NO |  |
| C36E8.3 | *pxd-1* | -0.3426 | E | YES | PLXDC1 |
| C07D8.6 | *C07D8.6* | -0.3428 | E | NO |  |
| C49C3.6 | *C49C3.6* | -0.3469 | E | NO |  |
| C32A3.1 | *sel-8* | -0.3474 | E | NO |  |
| Y67A6A.2 | *nhr-62* | -0.3496 | E | NO |  |
| F56D6.2 | *clec-67* | -0.3510 | E | NO |  |
| W08D2.4 | *fat-3* | -0.3542 | E | YES | FADS1 |
| M106.5 | *cap-2* | -0.3578 | E | YES | CAPZB |
| F23B2.6 | *aly-2* | -0.3583 | E | YES | THOC4 |
| F57B10.9 | *F57B10.9* | -0.3630 | E | YES | SPG20 |
| M110.4 | *ifg-1* | -0.3664 | E | YES | EIF4G3 |
| C52E4.1 | *cpr-1* | -0.3678 | E | NO |  |
| B0218.8 | *clec-52* | -0.3687 | E | YES | COLEC11 |
| F49E12.1 | *F49E12.1* | -0.3692 | E | YES | PXDNL |
| ZC410.5 | *ZC410.5* | -0.3711 | E | NO |  |
| T11B7.4 | *alp-1* | -0.3714 | E | YES | LDB3 |
| B0564.9 | *B0564.9* | -0.3728 | E | NO |  |
| F36F12.5 | *clec-207* | -0.3728 | E | NO |  |
| Y77E11A.2 | *Y77E11A.2* | -0.3752 | E | NO |  |
| C27A2.2 | *rpl-22* | -0.3778 | E | YES | RPL22 |
| F46F11.2 | *cey-2* | -0.3792 | E | YES | YBX1 |
| C01G6.1 | *aqp-2* | -0.3805 | E | YES | AQP3 |
| Y38H6C.3 | *dct-14* | -0.3805 | E | NO |  |
| C28A5.6 | *C28A5.6* | -0.3831 | E | NO |  |
| C05D12.4 | *C05D12.4* | -0.3887 | E | NO |  |
| E02D9.1 | *E02D9.1* | -0.3918 | E | NO |  |
| F56D12.6 | *F56D12.6* | -0.3932 | E | YES | FCHO2 |
| C56E6.6 | *C56E6.6* | -0.3936 | E | YES | TLR3 |
| ZK632.12 | *ZK632.12* | -0.3938 | E | YES | PLEKHF2 |
| F54C9.5 | *rpl-5* | -0.3985 | E | YES | RPL5 |
| Y51H1A.3 | *Y51H1A.3* | -0.3987 | E | YES | NDUFB8 |
| F45G2.4 | *F45G2.4* | -0.4010 | E | YES | COPE |
| C52B9.1 | *cka-2* | -0.4064 | E | YES | CHKB |
| R02F11.1 | *R02F11.1* | -0.4075 | E | NO |  |
| F54B11.3 | *unc-84* | -0.4090 | E | NO |  |
| C09C7.1 | *zig-4* | -0.4097 | E | NO |  |
| D1025.4 | *nspc-20* | -0.4158 | E | NO |  |
| Y87G2A.1 | *Y87G2A.1* | -0.4181 | E | NO |  |
| F35D2.5 | *syd-1* | -0.4195 | E | YES | ARHGAP18 |
| C02B10.4 | *C02B10.4* | -0.4223 | E | NO |  |
| T13C2.3 | *T13C2.3* | -0.4273 | E | NO |  |
| M03F4.7 | *calu-1* | -0.4303 | E | YES | CALU |
| C50F4.7 | *his-37* | -0.4323 | E | YES | HIST2H4B |
| Y56A3A.21 | *trap-4* | -0.4323 | E | YES | SSR4 |
| F57B1.7 | *F57B1.7* | -0.4334 | E | NO |  |
| R07H5.2 | *cpt-2* | -0.4337 | E | YES | CPT2 |
| C15C6.2 | *C15C6.2* | -0.4343 | E | NO |  |
| W09B6.1 | *pod-2* | -0.4349 | E | YES | ACACA |
| Y57A10A.31 | *Y57A10A.31* | -0.4393 | E | NO |  |
| F47A4.3 | *rrc-1* | -0.4394 | E | YES | ARHGAP33 |
| F38A5.8 | *F38A5.8* | -0.4396 | E | NO |  |
| F10E7.4 | *spon-1* | -0.4425 | E | YES | SPON1 |
| T07H3.3 | *math-38* | -0.4450 | E | NO |  |
| K07E3.3 | *dao-3* | -0.4459 | E | YES | MTHFD2 |
| F36A4.2 | *F36A4.2* | -0.4480 | E | NO |  |
| M01E5.3 | *M01E5.3* | -0.4495 | E | NO |  |
| E01G4.1 | *E01G4.1* | -0.4505 | E | NO |  |
| K11D2.3 | *unc-101* | -0.4506 | E | YES | AP1M1 |
| F38H4.9 | *let-92* | -0.4555 | E | YES | PPP2CB |
| F56H11.4 | *elo-1* | -0.4564 | E | NO |  |
| B0041.5 | *B0041.5* | -0.4565 | E | YES | SLC35F5 |
| Y54E10A.12 | *Y54E10A.12* | -0.4633 | E | NO |  |
| F40F9.9 | *aqp-4* | -0.4665 | E | YES | AQP8 |
| Y59H11AM.1 | *Y59H11AM.1* | -0.4681 | E | NO |  |
| F47G6.2 | *F47G6.2* | -0.4696 | E | NO |  |
| F44F1.3 | *F44F1.3* | -0.4705 | E | NO |  |
| Y56A3A.20 | *ccf-1* | -0.4763 | E | YES | CNOT7 |
| F29G6.2 | *F29G6.2* | -0.4770 | E | YES | CCDC149 |
| R02D3.5 | *R02D3.5* | -0.4777 | E | YES | FNTA |
| F49F1.12 | *F49F1.12* | -0.4793 | E | NO |  |
| C49H3.3 | *C49H3.3* | -0.4820 | E | YES | LLPH |
| F45C12.7 | *btb-6* | -0.4829 | E | NO |  |
| ZK666.7 | *clec-61* | -0.4854 | E | NO |  |
| C23H5.3 | *xbx-4* | -0.4882 | E | NO |  |
| C12C8.3 | *lin-41* | -0.4932 | E | YES | TRIM71 |
| B0403.2 | *ubc-17* | -0.4942 | E | NO |  |
| F28D1.1 | *F28D1.1* | -0.4969 | E | YES | ENSP00000372552 |
| T03F1.3 | *pgk-1* | -0.4975 | E | YES | PGK1 |
| F31E3.5 | *eft-3* | -0.4986 | E | YES | EEF1A2 |
| Y45F10B.1 | *tsp-5* | -0.5013 | E | NO |  |
| F46H5.3 | *F46H5.3* | -0.5031 | E | YES | CKB |
| B0496.1 | *B0496.1* | -0.5032 | E | NO |  |
| Y55F3AM.11 | *Y55F3AM.11* | -0.5072 | E | NO |  |
| C53B7.3 | *C53B7.3* | -0.5134 | E | NO |  |
| F16A11.1 | *F16A11.1* | -0.5195 | E | YES | RSPRY1 |
| ZK973.6 | *anc-1* | -0.5206 | E | YES | SYNE1 |
| T26A5.6 | *T26A5.6* | -0.5206 | E | YES | AVL9 |
| Y39E4B.6 | *Y39E4B.6* | -0.5222 | E | NO |  |
| C42C1.1 | *sre-14* | -0.5226 | E | NO |  |
| F44F1.4 | *F44F1.4* | -0.5255 | E | NO |  |
| C15F1.3 | *tra-2* | -0.5267 | E | NO |  |
| T02D1.7 | *T02D1.7* | -0.5306 | E | NO |  |
| C17D12.3 | *C17D12.3* | -0.5306 | E | NO |  |
| F23B12.8 | *bmk-1* | -0.5311 | E | YES | KIF11 |
| T16H12.1 | *T16H12.1* | -0.5348 | E | NO |  |
| ZK20.5 | *rpn-12* | -0.5391 | E | YES | PSMD8 |
| C41C4.7 | *ctns-1* | -0.5399 | E | YES | CTNS |
| K08C7.6 | *K08C7.6* | -0.5428 | E | NO |  |
| Y17G7B.11 | *Y17G7B.11* | -0.5429 | E | YES | ARRDC2 |
| F56E3.3 | *klp-4* | -0.5438 | E | YES | KIF13A |
| B0035.14 | *dnj-1* | -0.5441 | E | YES | DNAJB12 |
| C53B4.6 | *C53B4.6* | -0.5458 | E | NO |  |
| K08D10.7 | *scrm-8* | -0.5499 | E | NO |  |
| C03C10.3 | *rnr-2* | -0.5499 | E | YES | RRM2B |
| C49H3.4 | *C49H3.4* | -0.5523 | E | NO |  |
| K04G7.3 | *ogt-1* | -0.5535 | E | YES | OGT |
| F56H11.3 | *elo-7* | -0.5558 | E | NO |  |
| Y34D9B.1 | *mig-1* | -0.5561 | E | NO |  |
| F20D12.5 | *exc-9* | -0.5589 | E | YES | CRIP1 |
| C09G5.6 | *bli-1* | -0.5609 | E | YES | SFTPD |
| C33H5.8 | *C33H5.8* | -0.5617 | E | YES | RPAP3 |
| R11F4.2 | *R11F4.2* | -0.5641 | E | NO |  |
| ZK593.3 | *ZK593.3* | -0.5641 | E | NO |  |
| F57B10.7 | *tre-1* | -0.5645 | E | YES | TREH |
| M02F4.7 | *clec-265* | -0.5663 | E | NO |  |
| Y62E10A.15 | *cyp-31A5* | -0.5663 | E | NO |  |
| F40F11.4 | *F40F11.4* | -0.5682 | E | NO |  |
| F09E5.13 | *agt-2* | -0.5685 | E | NO |  |
| K09F6.3 | *K09F6.3* | -0.5701 | E | NO |  |
| ZK829.1 | *ZK829.1* | -0.5726 | E | YES | HSD17B14 |
| F57C2.5 | *F57C2.5* | -0.5732 | E | YES | C20orf3 |
| Y57G11C.24 | *eps-8* | -0.5755 | E | YES | EPS8L1 |
| R52.2 | *R52.2* | -0.5756 | E | NO |  |
| T21C12.3 | *T21C12.3* | -0.5835 | E | NO |  |
| T10D4.6 | *T10D4.6* | -0.5838 | E | NO |  |
| T20D3.7 | *vps-26* | -0.5858 | E | YES | VPS26B |
| F58A6.5 | *F58A6.5* | -0.5866 | E | NO |  |
| K07H8.1 | *K07H8.1* | -0.5908 | E | YES | TBCE |
| F14F11.1 | *F14F11.1* | -0.5927 | E | YES | KCNC1 |
| W05H5.4 | *srh-27* | -0.5927 | E | NO |  |
| F41E6.2 | *grd-5* | -0.5931 | E | NO |  |
| M01G12.12 | *rrf-2* | -0.5931 | E | NO |  |
| C05D11.3 | *tag-170* | -0.5954 | E | YES | TXNDC9 |
| T01D3.2 | *T01D3.2* | -0.5975 | E | YES | ENSP00000253050 |
| K09C4.5 | *K09C4.5* | -0.5978 | E | NO |  |
| Y54G9A.4 | *Y54G9A.4* | -0.5995 | E | YES | SLC39A2 |
| T12D8.9 | *T12D8.9* | -0.6007 | E | NO |  |
| C47E12.11 | *C47E12.11* | -0.6009 | E | NO |  |
| T21B4.8 | *srh-61* | -0.6013 | E | NO |  |
| F43E2.3 | *insc-1* | -0.6021 | E | NO |  |
| Y47G7B.2 | *Y47G7B.2* | -0.6036 | E | NO |  |
| F02E8.3 | *aps-2* | -0.6039 | E | YES | AP2S1 |
| F08B1.1 | *vhp-1* | -0.6103 | E | YES | DUSP8 |
| F02A9.1 | *F02A9.1* | -0.6124 | E | NO |  |
| R06F6.4 | *set-14* | -0.6132 | E | YES | SMYD2 |
| F46C3.1 | *pek-1* | -0.6140 | E | YES | EIF2AK3 |
| F54D10.8 | *F54D10.8* | -0.6161 | E | NO |  |
| F53G2.4 | *pqn-42* | -0.6171 | E | NO |  |
| R05F9.6 | *R05F9.6* | -0.6173 | E | YES | PGM1 |
| C34C6.5 | *sphk-1* | -0.6174 | E | YES | SPHK2 |
| F42A9.8 | *F42A9.8* | -0.6187 | E | NO |  |
| F54F7.3 | *F54F7.3* | -0.6202 | E | NO |  |
| Y48A6B.3 | *Y48A6B.3* | -0.6217 | E | YES | NHP2 |
| B0228.8 | *B0228.8* | -0.6235 | E | NO |  |
| T21C9.2 | *vps-54* | -0.6262 | E | YES | VPS54 |
| C35D10.5 | *C35D10.5* | -0.6306 | E | YES | UQCC |
| F43G9.12 | *F43G9.12* | -0.6306 | E | YES | GCFC1 |
| ZK616.3 | *ZK616.3* | -0.6321 | E | YES | CHCHD4 |
| K02E7.9 | *btb-10* | -0.6355 | E | NO |  |
| F33H2.7 | *set-10* | -0.6409 | E | NO |  |
| R01H10.4 | *R01H10.4* | -0.6448 | E | NO |  |
| F26H9.1 | *prom-1* | -0.6452 | E | NO |  |
| R05G9.3 | *R05G9.3* | -0.6452 | E | NO |  |
| C48B4.10 | *C48B4.10* | -0.6557 | E | NO |  |
| F35C11.6 | *F35C11.6* | -0.6604 | E | NO |  |
| B0491.1 | *B0491.1* | -0.6637 | E | YES | PIGM |
| C07G1.1 | *try-2* | -0.6716 | E | YES | PRSS22 |
| C34F11.5 | *C34F11.5* | -0.6718 | E | NO |  |
| C09E9.2 | *C09E9.2* | -0.6729 | E | NO |  |
| C01B12.5 | *C01B12.5* | -0.6744 | E | YES | BEST3 |
| E04F6.7 | *dhs-7* | -0.6757 | E | YES | ENSP00000333406 |
| F22B3.4 | *F22B3.4* | -0.6855 | E | YES | GFPT1 |
| C46A5.3 | *col-14* | -0.6871 | E | YES | MARCO |
| F52H3.3 | *bath-38* | -0.6898 | E | NO |  |
| ZK1248.5 | *ZK1248.5* | -0.6913 | E | NO |  |
| F59E11.5 | *F59E11.5* | -0.6938 | E | NO |  |
| F52H3.6 | *F52H3.6* | -0.6994 | E | NO |  |
| R12E2.15 | *R12E2.15* | -0.7075 | E | NO |  |
| K02F2.3 | *tag-203* | -0.7174 | E | YES | SF3B3 |
| F55G1.12 | *F55G1.12* | -0.7238 | E | NO |  |
| E02H1.4 | *pme-2* | -0.7253 | E | NO |  |
| M199.1 | *srt-44* | -0.7267 | E | NO |  |
| F09D12.1 | *grd-10* | -0.7278 | E | NO |  |
| T22F3.3 | *T22F3.3* | -0.7293 | E | YES | PYGM |
| Y46G5A.1 | *Y46G5A.1* | -0.7295 | E | NO |  |
| F31E8.4 | *F31E8.4* | -0.7306 | E | YES | ENSP00000378213 |
| C50D2.8 | *C50D2.8* | -0.7323 | E | YES | LUC7L3 |
| D1054.14 | *D1054.14* | -0.7343 | E | YES | PRPF38A |
| Y50D7A.7 | *ads-1* | -0.7376 | E | YES | AGPS |
| K12D12.3 | *col-84* | -0.7385 | E | YES | COLQ |
| Y73F8A.6 | *ccg-1* | -0.7432 | E | NO |  |
| Y40B1B.5 | *Y40B1B.5* | -0.7433 | E | NO |  |
| T02H6.5 | *T02H6.5* | -0.7442 | E | NO |  |
| R52.1 | *sdz-28* | -0.7519 | E | NO |  |
| F56D5.10 | *srxa-2* | -0.7521 | E | NO |  |
| ZK688.8 | *gly-3* | -0.7573 | E | YES | ENSP00000376574 |
| W05H12.1 | *W05H12.1* | -0.7597 | E | NO |  |
| W01B11.4 | *W01B11.4* | -0.7753 | E | NO |  |
| Y54G9A.7 | *Y54G9A.7* | -0.7756 | E | NO |  |
| C54C8.9 | *nlp-39* | -0.7806 | E | NO |  |
| Y56A3A.14 | *sdz-33* | -0.7806 | E | NO |  |
| T01G9.3 | *T01G9.3* | -0.7854 | E | NO |  |
| F08D12.8 | *fbxb-105* | -0.7911 | E | NO |  |
| F49E11.11 | *scl-3* | -0.7961 | E | YES | CRISP3 |
| Y54G11A.8 | *ddl-3* | -0.8062 | E | YES | TTC19 |
| F02H6.2 | *F02H6.2* | -0.8137 | E | NO |  |
| K02D7.5 | *K02D7.5* | -0.8137 | E | YES | SLC50A1 |
| R05A10.5 | *R05A10.5* | -0.8137 | E | NO |  |
| W09H1.1 | *W09H1.1* | -0.8149 | E | NO |  |
| M110.5 | *dab-1* | -0.8168 | E | YES | DAB2 |
| F02H6.6 | *F02H6.6* | -0.8169 | E | NO |  |
| R07H5.3 | *R07H5.3* | -0.8169 | E | NO |  |
| T20D3.1 | *clec-183* | -0.8169 | E | NO |  |
| Y73F4A.3 | *Y73F4A.3* | -0.8202 | E | NO |  |
| Y77E11A.13 | *npp-20* | -0.8202 | E | YES | SEC13 |
| C18E9.2 | *C18E9.2* | -0.8256 | E | YES | SEC62 |
| E02H1.3 | *tag-124* | -0.8256 | E | YES | PUS3 |
| R166.4 | *pro-1* | -0.8313 | E | YES | WDR18 |
| F11G11.10 | *col-17* | -0.8359 | E | NO |  |
| E04D5.4 | *E04D5.4* | -0.8377 | E | NO |  |
| T24H7.3 | *T24H7.3* | -0.8460 | E | YES | ENSP00000330180 |
| T19C4.6 | *gpa-1* | -0.8536 | E | NO |  |
| B0334.11 | *B0334.11* | -0.8540 | E | NO |  |
| Y48E1B.8 | *Y48E1B.8* | -0.8540 | E | NO |  |
| T22E5.5 | *mup-2* | -0.9054 | E | NO |  |
