# Supplementary material for: Large-scale functional RNAi screen in C. elegans identifies genes that regulate the dysfunction of mutant polyglutamine neurons
Source: BMC Genomics. 2012 Mar 13;13:91. doi: 10.1186/1471-2164-13-91 (PMC3331833; doi:10.1186/1471-2164-13-91)
Supplement: Additional file 5 — Table S4. List of the 15 genes that modified 19Q-neuron dysfunction when knocked-down by RNAi. [file 1471-2164-13-91-S5.DOC]

**Supplementary Table 4.** List of the 15 genes that modify 19Q-neuron dysfunction when knocked-down by RNAi.

A total of 245 genes confirmed to modify 128Q-neuron dysfunction were tested. S means suppression of 19Q-neuron dysfunction when knocked-down by RNAi, whereas E means enhancement of 19Q-neuron dysfunction. Conservation in humans (best orthologs) is also indicated as inferred from InParanoid clusters.

| **Gene ID** | **Gene name** | **Effect** | **Concise description (WormBase)** | **Human best ortholog** |
| --- | --- | --- | --- | --- |
| F22D6.12 | *gly-19* | E | gly-19 encodes a 2/I N-acetylglucosaminyltransferase; a gly-19::gfp promoter fusion is expressed in the intestine and in the anal sphincter muscle from embryogensis through adulthood. | GCNT1 |
| F33H2.7 | *set-10* | E | set-10 encodes a divergent ortholog of human SMYD1 (OMIM:606846), SMYD2 (OMIM:610663), and SYMD3 (OMIM:608783); SET-10 is paralogous to SET-14, SET-18, and SET-30; SET-10 has no obvious function in individual RNAi assays of vulval development, or in mass RNAi assays. |  |
| F44F1.3 | *F44F1.3* | E | none available |  |
| F52H3.6 | *F52H3.6* | E | none available |  |
| F54D10.8 | *F54D10.8* | E | none available |  |
| F59E11.5 | *F59E11.5* | E | none available |  |
| K09F6.3 | *K09F6.3* | E | none available |  |
| T01G9.3 | *T01G9.3* | E | none available |  |
| T24H7.3 | *T24H7.3* | E | none available | ENSP00000330180 |
| W01B11.4 | *W01B11.4* | E | The gene named W01B11.4 (WBGene00020916) has been superseded or retired. |  |
| Y17G7B.11 | *Y17G7B.11* | E | none available | ARRDC2 |
| Y47G7B.2 | *Y47G7B.2* | E | none available |  |
| Y50D7A.7 | *ads-1* | E | ads-1 encodes an ortholog of human ALKYL-DIHYDROXYACETONEPHOSPHATE SYNTHASE PRECURSOR (AGPS; OMIM:603051) that is required for normal larval development; mutation of human AGPS leads to type 3 rhizomelic chondrodysplasia punctata (OMIM:600121). | AGPS |
| ZK688.8 | *gly-3* | E | gly-3 encodes an N-acetylgalactosaminyltransferase that is functional in vitro. | ENSP00000376574 |
| ZC15.8 | *pqn-94* | S | The protein product of this gene is predicted to contain a glutamine/asparagine (Q/N)-rich ('prion') domain, by the algorithm of Michelitsch and Weissman (as of the WS77 release of WormBase, i.e., in wormpep77). |  |
