# Supplementary material for: Large-scale functional RNAi screen in C. elegans identifies genes that regulate the dysfunction of mutant polyglutamine neurons
Source: BMC Genomics. 2012 Mar 13;13:91. doi: 10.1186/1471-2164-13-91 (PMC3331833; doi:10.1186/1471-2164-13-91)
Supplement: Additional file 6 — Table S5. List of the 15 genes that modified 128Q-neuron dysfunction in the secondary screen and were previously reported to modify polyQ aggregation when knocked-down by RNAi. [file 1471-2164-13-91-S6.DOC]

**Supplementary Table 5.** List of the 15 genes that modified 128Q-neuron dysfunction in the secondary screen and were previously reported to modify the aggregation of 35Q-polypeptides (Q35) when knocked-down by RNAi.

S, suppression of 128Q-neuron dysfunction by RNAi; E, enhancement of 128Q-neuron dysfunction by RNAi.

| **Gene ID** | **Gene name** | **S score** | **Effect** | **Human best ortholog** | **Enhances Q35 aggregation when knocked-down in *C. elegans* (Ref. 40)** | **Suppresses Q35 aggregation when knocked-down in *C. elegans* (Ref. 41)** |
| --- | --- | --- | --- | --- | --- | --- |
| T28D9.10 | *snr-3* | 1.5043 | S | SNRPD1 | Yes | No |
| F26E4.8 | *tba-1* | 1.5004 | S | none | Yes | No |
| Y47D3B.7 | *sbp-1* | 0.6631 | S | SREBF2 | Yes | No |
| T27F2.1 | *skp-1* | 0.6368 | S | SNW1 | Yes | No |
| K02D10.5 | *K02D10.5* | 0.6302 | S | SNAP29 | Yes | No |
| T21B10.7 | *cct-2* | 0.4592 | S | CCT2 | Yes | No |
| W08E3.1 | *snr-2* | 0.3548 | S | SNRPB | Yes | No |
| C01F1.3 | *C01F1.3* | 0.3231 | S | TGDS | Yes | No |
| F28H1.3 | *ars-2* | 0.3025 | S | AARS | Yes | No |
| F54C9.5 | *rpl-5* | -0.3985 | E | RPL5 | Yes | No |
| W09B6.1 | *pod-2* | -0.4349 | E | ACACA | Yes | No |
| K02F2.3 | *tag-203* | -0.7174 | E | SF3B3 | Yes | No |
| C50D2.2 | *C50D2.2* | 1.4283 | S | SLC7A4 | No | Yes |
| B0281.5 | *B0281.5* | 0.7889 | S | KCNRG | No | Yes |
| C03C10.3 | *rnr-2* | -0.5499 | E | RRM2B | No | Yes |
