# Supplementary material for: Large-scale functional RNAi screen in C. elegans identifies genes that regulate the dysfunction of mutant polyglutamine neurons
Source: BMC Genomics. 2012 Mar 13;13:91. doi: 10.1186/1471-2164-13-91 (PMC3331833; doi:10.1186/1471-2164-13-91)
Supplement: Additional file 7 — Table S6. Gene Ontology classification of genes that suppressed 128Q-neuron dysfunction when knocked-down by RNAi. [file 1471-2164-13-91-S7.DOC]

**Supplementary Table 6.** Gene Ontology classification of genes that suppress 128Q-neuron dysfunction when knocked-down by RNAi.

Genes were classified based on their functional annotations in the GO categories ‘Biological Process’, ‘Molecular Function’ and ‘Cellular component’ as indicated in the sub-headings of the table. While *P* < 0.05 was considered significant, *P* values for all of the GO terms is shown.

| **GO ID** | **GO description** | **Genes** | **P value** |
| --- | --- | --- | --- |
| **Biological Process** |  |  |  |
| GO:0007275 | multicellular organismal development | *hlh-1; his-13; aos-1; col-94; rpt-6; snt-2; ugt-1; cct-2; rpl-11.1; r06a4.9; lin-2; sur-6; y37d8a.19; stdh-2; t04g9.4; tra-1; ife-3; ssl-1; pnk-1; inx-9; lon-1; f57c9.4; hpl-2; nspb-4; pah-1; zk673.3; pha-4; nxt-1; tbg-1; t12f5.1; cye-1; ace-3; k02d10.5; w07e6.2; sna-2; ins-18; vab-3; f43g9.5; t02g5.7; w02d9.2; cogc-1; w06e11.1; rpl-25.2; nuc-1; tnc-2; c09g9.7; cpar-1; mdt-6; nhx-6; cnd-1; gsk-3; gpc-2; c17e4.3; tba-1; c37a2.7; lex-1; cul-2; zc395.10; glc-2; r05f9.1; evl-20; t17h7.1; ars-2; f55f8.2; w02d3.1; f25h9.6; csn-5; sec-8; pfn-1; snr-3; apc-10; f48c1.4; f44d12.8; r07e5.3; f10e9.7; cdk-7; stdh-1; lbp-7; c01f1.3; ubc-9; cki-1; t13f2.6; unc-32; gpd-2; daf-7; skp-1; k08e3.5; y65b4br.5; rps-22; rnp-3; f54d5.9; f27c1.4; f53b7.3; ash-2; f10e9.4; w09d10.3; k01g5.5; mdl-1; c01a2.5; unc-130; emo-1; t26c5.3; fog-3; lgg-1; hsp-12.6; mdt-15; cle-1; cyh-1; cyd-1; snr-2; his-7; f40f8.1; fkb-3; t23b12.6; unc-129; k07c5.4; c56c10.10; gly-4; his-48; pfd-3; ufd-1; cul-1; f23h12.2; mpk-1; ajm-1; t10h9.3; sbp-1; fem-2* | 2.71e-10 |
| GO:0032502 | developmental process | *hlh-1; his-13; aos-1; col-94; rpt-6; snt-2; ugt-1; cct-2; rpl-11.1; r06a4.9; lin-2; sur-6; y37d8a.19; stdh-2; t04g9.4; tra-1; ife-3; ssl-1; pnk-1; inx-9; lon-1; f57c9.4; hpl-2; nspb-4; pah-1; zk673.3; pha-4; nxt-1; tbg-1; t12f5.1; cye-1; ace-3; k02d10.5; w07e6.2; sna-2; ins-18; sdc-3; vab-3; f43g9.5; t02g5.7; w02d9.2; cogc-1; w06e11.1; rpl-25.2; nuc-1; tnc-2; c09g9.7; cpar-1; mdt-6; nhx-6; cnd-1; gsk-3; gpc-2; c17e4.3; tba-1; c37a2.7; lex-1; cul-2; zc395.10; glc-2; r05f9.1; evl-20; t17h7.1; ars-2; f55f8.2; w02d3.1; f25h9.6; csn-5; sec-8; pfn-1; snr-3; apc-10; f48c1.4; f44d12.8; r07e5.3; f10e9.7; cdk-7; stdh-1; lbp-7; c01f1.3; ubc-9; unc-32; cki-1; t13f2.6; gpd-2; daf-7; skp-1; k08e3.5; y65b4br.5; rps-22; rnp-3; f54d5.9; f27c1.4; f53b7.3; ash-2; f10e9.4; w09d10.3; k01g5.5; mdl-1; c01a2.5; unc-130; emo-1; t26c5.3; lgg-1; fog-3; hsp-12.6; mdt-15; cle-1; cyh-1; cyd-1; snr-2; asp-4; his-7; f40f8.1; fkb-3; t23b12.6; unc-129; pmk-1; k07c5.4; c56c10.10; gly-4; his-48; pfd-3; ufd-1; cul-1; f23h12.2; mpk-1; t10h9.3; ajm-1; sbp-1; fem-2* | 5.94e-10 |
| GO:0003006 | reproductive developmental process | *cle-1; r07e5.3; cyh-1; cogc-1; cct-2; rpl-11.1; rpl-25.2; cpar-1; mdt-6; ubc-9; unc-32; cki-1; tra-1; skp-1; hpl-2; y65b4br.5; tbg-1; nxt-1; k07c5.4; lex-1; cye-1; his-48; evl-20; pfd-3; sdc-3; emo-1; mpk-1; csn-5; sec-8; fog-3; f48c1.4; fem-2* | 1.15e-05 |
| GO:0009792 | embryonic development ending in birth or egg hatching | *hlh-1; his-13; aos-1; rpt-6; f44d12.8; snt-2; r07e5.3; f10e9.7; cdk-7; cct-2; rpl-11.1; sur-6; t04g9.4; ubc-9; cki-1; unc-32; t13f2.6; tra-1; ife-3; ssl-1; pnk-1; inx-9; lon-1; skp-1; f57c9.4; k08e3.5; nspb-4; pah-1; y65b4br.5; zk673.3; pha-4; rps-22; tbg-1; nxt-1; f54d5.9; f53b7.3; ash-2; t12f5.1; cye-1; k02d10.5; mdl-1; sna-2; c01a2.5; emo-1; f43g9.5; t26c5.3; lgg-1; w02d9.2; mdt-15; cogc-1; cyh-1; w06e11.1; snr-2; rpl-25.2; c09g9.7; cpar-1; mdt-6; his-7; nhx-6; f40f8.1; t23b12.6; gsk-3; gpc-2; c17e4.3; tba-1; k07c5.4; c37a2.7; c56c10.10; lex-1; gly-4; his-48; cul-2; zc395.10; glc-2; r05f9.1; evl-20; t17h7.1; pfd-3; ars-2; cul-1; ufd-1; w02d3.1; f23h12.2; csn-5; mpk-1; pfn-1; ajm-1; snr-3; apc-10; sbp-1; f48c1.4* | 5.1e-05 |
| GO:0022414 | reproductive process | *cle-1; r07e5.3; cyh-1; cogc-1; cct-2; rpl-11.1; rpl-25.2; sur-6; lin-2; cpar-1; c01f1.3; mdt-6; ubc-9; unc-32; cki-1; cnd-1; tra-1; gpc-2; skp-1; tba-1; hpl-2; y65b4br.5; eri-1; nxt-1; tbg-1; k07c5.4; lex-1; cye-1; his-48; evl-20; pfd-3; sdc-3; emo-1; sec-8; mpk-1; csn-5; hlh-8; fog-3; fem-2; f48c1.4* | 5.34e-05 |
| GO:0009790 | embryonic development | *hlh-1; his-13; aos-1; rpt-6; f44d12.8; snt-2; r07e5.3; f10e9.7; cdk-7; cct-2; rpl-11.1; sur-6; t04g9.4; ubc-9; cki-1; unc-32; t13f2.6; tra-1; ife-3; ssl-1; pnk-1; inx-9; lon-1; skp-1; f57c9.4; k08e3.5; nspb-4; pah-1; y65b4br.5; zk673.3; pha-4; rps-22; tbg-1; nxt-1; f54d5.9; f53b7.3; ash-2; t12f5.1; cye-1; k02d10.5; mdl-1; sna-2; c01a2.5; unc-130; emo-1; f43g9.5; t26c5.3; lgg-1; w02d9.2; mdt-15; cogc-1; cyh-1; w06e11.1; snr-2; rpl-25.2; c09g9.7; cpar-1; mdt-6; his-7; nhx-6; f40f8.1; t23b12.6; gsk-3; gpc-2; c17e4.3; tba-1; k07c5.4; c37a2.7; c56c10.10; lex-1; gly-4; his-48; cul-2; zc395.10; glc-2; r05f9.1; evl-20; t17h7.1; pfd-3; ars-2; cul-1; ufd-1; w02d3.1; f23h12.2; mpk-1; csn-5; pfn-1; ajm-1; snr-3; apc-10; sbp-1; f48c1.4* | 0.000112 |
| GO:0007548 | sex differentiation | *tbg-1; nxt-1; k07c5.4; lex-1; cye-1; cle-1; r07e5.3; cyh-1; cogc-1; his-48; cct-2; rpl-11.1; rpl-25.2; evl-20; pfd-3; cpar-1; mdt-6; ubc-9; emo-1; cki-1; unc-32; tra-1; mpk-1; csn-5; sec-8; skp-1; hpl-2; y65b4br.5; f48c1.4* | 0.000112 |
| GO:0016043 | cellular component organization and biogenesis | *hlh-1; his-13; r07e5.3; sedl-1; nuc-1; sur-6; cpar-1; his-7; cnd-1; rab-28; unc-129; gsk-3; ife-3; gpc-2; rap-1; tba-1; hpl-2; y65b4br.5; hil-3; unc-64; tbg-1; cul-2; his-48; dlc-2; evl-20; pfd-3; unc-130; cul-1; sdc-3; rab-10; emo-1; f23h12.2; sec-8; csn-5; pfn-1; rab-37; ajm-1; tomm-7; fbl-1* | 0.00013 |
| GO:0048731 | system development | *hlh-1; cle-1; r07e5.3; cyh-1; cogc-1; cct-2; rpl-11.1; rpl-25.2; sur-6; lin-2; cpar-1; mdt-6; ubc-9; unc-32; cki-1; cnd-1; unc-129; tra-1; skp-1; hpl-2; y65b4br.5; nxt-1; tbg-1; k07c5.4; lex-1; cye-1; his-48; evl-20; pfd-3; unc-130; emo-1; sec-8; mpk-1; csn-5; f48c1.4* | 0.00013 |
| GO:0002119 | larval development (sensu Nematoda) | *hlh-1; his-13; aos-1; col-94; rpt-6; mdt-15; r07e5.3; cyh-1; cogc-1; w06e11.1; cyd-1; cct-2; snr-2; r06a4.9; lin-2; sur-6; y37d8a.19; c01f1.3; tnc-2; his-7; ubc-9; unc-32; gsk-3; daf-7; pnk-1; skp-1; tba-1; k08e3.5; hpl-2; nspb-4; y65b4br.5; pha-4; rps-22; rnp-3; nxt-1; k07c5.4; f27c1.4; f10e9.4; cye-1; w09d10.3; k02d10.5; ace-3; w07e6.2; k01g5.5; sna-2; c01a2.5; ars-2; f55f8.2; cul-1; ufd-1; emo-1; f23h12.2; f25h9.6; mpk-1; t10h9.3; snr-3; lgg-1; sbp-1; f48c1.4* | 0.000278 |
| GO:0002164 | larval development | *hlh-1; his-13; aos-1; col-94; rpt-6; mdt-15; r07e5.3; cyh-1; cogc-1; w06e11.1; cyd-1; cct-2; snr-2; r06a4.9; lin-2; sur-6; y37d8a.19; c01f1.3; tnc-2; his-7; ubc-9; unc-32; gsk-3; daf-7; pnk-1; skp-1; tba-1; k08e3.5; hpl-2; nspb-4; y65b4br.5; pha-4; rps-22; rnp-3; nxt-1; k07c5.4; f27c1.4; f10e9.4; cye-1; w09d10.3; k02d10.5; ace-3; w07e6.2; k01g5.5; sna-2; c01a2.5; ars-2; f55f8.2; cul-1; ufd-1; emo-1; f23h12.2; f25h9.6; mpk-1; t10h9.3; snr-3; lgg-1; sbp-1; f48c1.4* | 0.000278 |
| GO:0009791 | post-embryonic development | *hlh-1; his-13; aos-1; col-94; rpt-6; mdt-15; r07e5.3; cyh-1; cogc-1; w06e11.1; cyd-1; cct-2; snr-2; r06a4.9; lin-2; sur-6; y37d8a.19; c01f1.3; tnc-2; his-7; ubc-9; cki-1; unc-32; gsk-3; daf-7; pnk-1; skp-1; tba-1; k08e3.5; hpl-2; nspb-4; y65b4br.5; pha-4; rps-22; rnp-3; nxt-1; k07c5.4; f27c1.4; f10e9.4; cye-1; w09d10.3; k02d10.5; ace-3; w07e6.2; k01g5.5; sna-2; c01a2.5; evl-20; ars-2; f55f8.2; cul-1; ufd-1; emo-1; f23h12.2; t02g5.7; f25h9.6; mpk-1; t10h9.3; snr-3; lgg-1; sbp-1; f48c1.4* | 0.000351 |
| GO:0065007 | biological regulation | *hlh-1; gsto-3; his-13; aos-1; col-94; snt-2; r07e5.3; f10e9.7; rpl-11.1; r06a4.9; lin-2; sur-6; t04g9.4; ubc-9; unc-32; cki-1; tra-1; daf-7; pnk-1; lon-1; skp-1; k08e3.5; elt-3; hpl-2; y65b4br.5; eri-1; pha-4; rps-22; nlp-40; w09d10.3; cye-1; f10e9.4; ceh-38; ace-3; zc204.2; w07e6.2; k01g5.5; mdl-1; sna-2; mxl-1; c01a2.5; unc-130; ins-18; vab-3; sdc-3; emo-1; fog-3; aha-1; mdt-15; cogc-1; cyh-1; w06e11.1; cyd-1; rpl-25.2; skr-15; vab-15; asp-4; mdt-6; his-7; nhx-6; cnd-1; gsk-3; unc-49; ftn-2; r02f2.1; k07c5.4; c37a2.7; his-48; cul-2; tag-218; evl-20; pfd-3; ars-2; f55f8.2; c29f9.5; cul-1; ufd-1; rab-10; f25h2.2; f23h12.2; ceh-23; f25h9.6; sec-8; t10h9.3; ajm-1; snr-3; hlh-8; apc-10; sbp-1; f48c1.4* | 0.000428 |
| GO:0048513 | organ development | *nxt-1; tbg-1; hlh-1; k07c5.4; lex-1; cye-1; cle-1; r07e5.3; cyh-1; cogc-1; his-48; cct-2; rpl-11.1; rpl-25.2; evl-20; pfd-3; sur-6; lin-2; cpar-1; mdt-6; ubc-9; emo-1; unc-32; cki-1; tra-1; sec-8; csn-5; mpk-1; skp-1; hpl-2; y65b4br.5; f48c1.4* | 0.000515 |
| GO:0051649 | establishment of cellular localization | *unc-64; tbg-1; rab-10; emo-1; rab-28; f23h12.2; gsk-3; gpc-2; rap-1; sec-8; csn-5; cul-2; rab-37; tba-1; tomm-7; sedl-1; y65b4br.5; evl-20; pfd-3* | 0.000677 |
| GO:0051641 | cellular localization | *unc-64; tbg-1; rab-10; emo-1; rab-28; f23h12.2; gsk-3; gpc-2; rap-1; sec-8; csn-5; cul-2; rab-37; tba-1; tomm-7; sedl-1; y65b4br.5; evl-20; pfd-3* | 0.000932 |
| GO:0007049 | cell cycle | *tbg-1; sdc-3; cul-1; cki-1; cye-1; gsk-3; csn-5; cul-2; cyd-1; tba-1; cdk-7; apc-10; r02f2.1; fog-3; eri-1* | 0.00124 |
| GO:0048518 | positive regulation of biological process | *hlh-1; his-13; col-94; snt-2; mdt-15; r07e5.3; cyh-1; cogc-1; w06e11.1; f10e9.7; rpl-11.1; rpl-25.2; skr-15; r06a4.9; lin-2; sur-6; asp-4; his-7; ubc-9; t04g9.4; unc-32; tra-1; gsk-3; daf-7; pnk-1; lon-1; k08e3.5; y65b4br.5; pha-4; rps-22; k07c5.4; c37a2.7; f10e9.4; cye-1; w09d10.3; nlp-40; his-48; w07e6.2; k01g5.5; sna-2; c01a2.5; evl-20; ars-2; pfd-3; f55f8.2; sdc-3; vab-3; ufd-1; rab-10; f25h2.2; f23h12.2; f25h9.6; sec-8; ajm-1; t10h9.3; sbp-1; f48c1.4; aha-1* | 0.00132 |
| GO:0050789 | regulation of biological process | *hlh-1; his-13; aos-1; col-94; snt-2; r07e5.3; f10e9.7; rpl-11.1; r06a4.9; lin-2; sur-6; t04g9.4; ubc-9; unc-32; cki-1; tra-1; daf-7; pnk-1; lon-1; k08e3.5; elt-3; hpl-2; y65b4br.5; eri-1; pha-4; rps-22; nlp-40; w09d10.3; cye-1; f10e9.4; ace-3; ceh-38; zc204.2; w07e6.2; k01g5.5; mdl-1; sna-2; mxl-1; c01a2.5; unc-130; ins-18; vab-3; sdc-3; fog-3; aha-1; mdt-15; cogc-1; cyh-1; w06e11.1; cyd-1; rpl-25.2; skr-15; vab-15; asp-4; mdt-6; his-7; cnd-1; gsk-3; unc-49; r02f2.1; k07c5.4; c37a2.7; cul-2; his-48; tag-218; evl-20; pfd-3; ars-2; f55f8.2; c29f9.5; rab-10; cul-1; ufd-1; f25h2.2; f23h12.2; ceh-23; f25h9.6; sec-8; t10h9.3; ajm-1; hlh-8; apc-10; sbp-1; f48c1.4* | 0.00149 |
| GO:0048856 | anatomical structure development | *hlh-1; his-13; col-94; cle-1; r07e5.3; cyh-1; cogc-1; cct-2; snr-2; rpl-11.1; rpl-25.2; lin-2; sur-6; cpar-1; mdt-6; ubc-9; cnd-1; unc-32; cki-1; unc-129; tra-1; skp-1; hpl-2; y65b4br.5; nxt-1; tbg-1; k07c5.4; lex-1; cye-1; his-48; c01a2.5; evl-20; pfd-3; unc-130; sdc-3; cul-1; vab-3; emo-1; t02g5.7; mpk-1; csn-5; sec-8; sbp-1; f48c1.4* | 0.00524 |
| GO:0040035 | hermaphrodite genitalia development | *tbg-1; nxt-1; k07c5.4; cye-1; cle-1; r07e5.3; cyh-1; cogc-1; his-48; cct-2; rpl-11.1; rpl-25.2; evl-20; pfd-3; cpar-1; ubc-9; unc-32; tra-1; sec-8; mpk-1; skp-1; y65b4br.5; f48c1.4* | 0.00629 |
| GO:0000074 | regulation of progression through cell cycle | *cul-1; apc-10; cki-1; fog-3; cye-1; cul-2* | 0.00629 |
| GO:0022402 | cell cycle process | *tbg-1; sdc-3; cul-1; cki-1; cye-1; gsk-3; csn-5; cul-2; cyd-1; tba-1; apc-10; r02f2.1; fog-3; eri-1* | 0.00629 |
| GO:0048806 | genitalia development | *tbg-1; nxt-1; k07c5.4; cye-1; cle-1; r07e5.3; cyh-1; cogc-1; his-48; cct-2; rpl-11.1; rpl-25.2; evl-20; pfd-3; cpar-1; ubc-9; unc-32; tra-1; sec-8; mpk-1; skp-1; y65b4br.5; f48c1.4* | 0.0079 |
| GO:0051726 | regulation of cell cycle | *cyd-1; cul-1; apc-10; cki-1; fog-3; r02f2.1; cye-1; cul-2* | 0.00915 |
| GO:0048522 | positive regulation of cellular process | *vab-3; unc-32; tra-1; cye-1; sbp-1; daf-7; asp-4* | 0.0122 |
| GO:0000278 | mitotic cell cycle | *tbg-1; sdc-3; cul-1; cye-1; gsk-3; csn-5; cdk-7; tba-1; apc-10* | 0.0166 |
| GO:0048608 | reproductive structure development | *mdt-6; lex-1; emo-1; cki-1; hpl-2; cogc-1; csn-5* | 0.0173 |
| GO:0008406 | gonad development | *mdt-6; lex-1; emo-1; cki-1; hpl-2; cogc-1; csn-5* | 0.0173 |
| GO:0040010 | positive regulation of growth rate | *hlh-1; his-13; mdt-15; cyh-1; cogc-1; w06e11.1; f10e9.7; rpl-11.1; rpl-25.2; skr-15; r06a4.9; his-7; t04g9.4; ubc-9; unc-32; gsk-3; pnk-1; lon-1; k08e3.5; y65b4br.5; pha-4; rps-22; k07c5.4; c37a2.7; nlp-40; w09d10.3; f10e9.4; his-48; w07e6.2; k01g5.5; sna-2; c01a2.5; evl-20; pfd-3; ars-2; f55f8.2; ufd-1; f25h2.2; f23h12.2; f25h9.6; sec-8; t10h9.3; ajm-1; sbp-1; aha-1* | 0.022 |
| GO:0040009 | regulation of growth rate | *hlh-1; his-13; mdt-15; cyh-1; cogc-1; w06e11.1; f10e9.7; rpl-11.1; rpl-25.2; skr-15; r06a4.9; his-7; t04g9.4; ubc-9; unc-32; gsk-3; pnk-1; lon-1; k08e3.5; y65b4br.5; pha-4; rps-22; k07c5.4; c37a2.7; nlp-40; w09d10.3; f10e9.4; his-48; w07e6.2; k01g5.5; sna-2; c01a2.5; evl-20; pfd-3; ars-2; f55f8.2; ufd-1; f25h2.2; f23h12.2; f25h9.6; sec-8; t10h9.3; ajm-1; sbp-1; aha-1* | 0.022 |
| GO:0008340 | determination of adult life span | *stdh-2; hsp-12.6; ins-18; fkb-3; gpd-2; mdt-15; ugt-1; mdl-1; nuc-1; stdh-1; lbp-7* | 0.022 |
| GO:0010259 | multicellular organismal aging | *stdh-2; hsp-12.6; ins-18; fkb-3; gpd-2; mdt-15; ugt-1; mdl-1; nuc-1; stdh-1; lbp-7* | 0.022 |
| GO:0007568 | aging | *stdh-2; hsp-12.6; ins-18; fkb-3; gpd-2; mdt-15; ugt-1; mdl-1; nuc-1; stdh-1; lbp-7* | 0.0242 |
| GO:0042023 | DNA endoreduplication | *lon-1; cye-1* | 0.0242 |
| GO:0045137 | development of primary sexual characteristics | *mdt-6; lex-1; emo-1; cki-1; hpl-2; cogc-1; csn-5* | 0.0242 |
| GO:0009987 | cellular process | *hlh-1; gsto-3; his-13; aos-1; b0286.3; ugt-1; cct-2; rpl-11.1; m02d8.4; k08f4.1; lin-2; sur-6; f42g10.1; pph-5; tra-1; rab-28; ife-3; rap-1; pnk-1; lon-1; c10c5.5; t19b4.3; elt-3; hpl-2; pah-1; hil-3; c34b2.4; pha-4; lap-1; tbg-1; unc-64; cye-1; ace-3; tgt-1; mxl-1; dlc-2; h04m03.1; ins-18; sdc-3; vab-3; tomm-7; f54d10.7; cogc-1; w06e11.1; rpl-25.2; nuc-1; y19d10b.6; tnc-2; cpar-1; mdt-6; cnd-1; cyn-3; gsk-3; gpc-2; tba-1; r02f2.1; c37a2.7; cul-2; tag-218; c18a3.1; evl-20; ars-2; sod-5; f21f3.2; ceh-23; csn-5; sec-8; pfn-1; rab-37; snr-3; zk1098.4; apc-10; fbl-1; r07e5.3; bre-1; cdk-7; sedl-1; c01f1.3; c26b9.5; max-2; ubc-9; unc-32; cki-1; gpd-2; daf-7; y65b4br.5; eri-1; lsm-1; rps-22; w09d10.3; ceh-38; y69f12a.1; gly-14; zc204.2; k01g5.5; mdl-1; c10c5.4; unc-130; emo-1; lgg-1; fog-3; aha-1; jnk-1; zk1127.10; cle-1; cyd-1; snr-2; skr-15; vab-15; asp-4; his-7; qdpr-1; f40f8.1; fkb-3; unc-129; ubc-20; unc-49; ftn-2; y43c5b.2; pmk-1; c44h4.6; his-48; c39d10.7; pfd-3; c29f9.5; rab-10; ufd-1; cul-1; f25h2.2; f23h12.2; mpk-1; ajm-1; hlh-8; sbp-1; bre-2; fem-2* | 0.0242 |
| GO:0044237 | cellular metabolic process | *hlh-1; his-13; b0286.3; r07e5.3; ugt-1; bre-1; cdk-7; cct-2; rpl-11.1; m02d8.4; k08f4.1; lin-2; f42g10.1; c26b9.5; c01f1.3; pph-5; max-2; ubc-9; gpd-2; ife-3; daf-7; pnk-1; lon-1; c10c5.5; t19b4.3; elt-3; hpl-2; pah-1; hil-3; eri-1; pha-4; lsm-1; rps-22; tbg-1; lap-1; cye-1; w09d10.3; ceh-38; ace-3; y69f12a.1; gly-14; zc204.2; tgt-1; k01g5.5; mdl-1; mxl-1; c10c5.4; h04m03.1; unc-130; vab-3; aha-1; f54d10.7; jnk-1; zk1127.10; w06e11.1; snr-2; rpl-25.2; skr-15; nuc-1; vab-15; y19d10b.6; asp-4; cpar-1; his-7; qdpr-1; mdt-6; f40f8.1; cnd-1; fkb-3; gsk-3; cyn-3; ubc-20; tba-1; y43c5b.2; pmk-1; c37a2.7; c44h4.6; his-48; cul-2; c18a3.1; c39d10.7; ars-2; pfd-3; c29f9.5; sod-5; cul-1; ufd-1; f21f3.2; rab-10; ceh-23; mpk-1; snr-3; zk1098.4; hlh-8; bre-2; sbp-1; fem-2* | 0.0278 |
| GO:0002009 | morphogenesis of an epithelium | *cpar-1; col-94; sdc-3; ubc-9; tra-1; cyh-1; cogc-1; sec-8; snr-2; evl-20; pfd-3; f48c1.4; sur-6* | 0.0303 |
| GO:0046907 | intracellular transport | *unc-64; tbg-1; rab-10; emo-1; f23h12.2; rab-28; rap-1; rab-37; tba-1; tomm-7; sedl-1; evl-20* | 0.0303 |
| GO:0043170 | macromolecule metabolic process | *hlh-1; his-13; rpt-6; r07e5.3; ugt-1; bre-1; cdk-7; hex-1; cct-2; rpl-11.1; k08f4.1; lin-2; f42g10.1; c01f1.3; c26b9.5; pph-5; max-2; ubc-9; t04g9.4; gpd-2; ife-3; daf-7; lon-1; c10c5.5; elt-3; hpl-2; hil-3; eri-1; pha-4; lsm-1; rps-22; tbg-1; lap-1; cye-1; w09d10.3; ceh-38; y69f12a.1; gly-14; zc204.2; tgt-1; k01g5.5; mxl-1; c10c5.4; h04m03.1; unc-130; vab-3; sdc-3; aha-1; f54d10.7; jnk-1; snr-2; rpl-25.2; skr-15; nuc-1; vab-15; y19d10b.6; asp-4; cpar-1; his-7; fkb-3; cyn-3; gsk-3; ubc-20; tba-1; y43c5b.2; pmk-1; c37a2.7; c44h4.6; his-48; cul-2; lys-8; c39d10.7; pfd-3; ars-2; c29f9.5; rab-10; cul-1; ufd-1; f21f3.2; ceh-23; mpk-1; snr-3; bre-2; sbp-1; fem-2* | 0.0303 |
| GO:0044238 | primary metabolic process | *hlh-1; his-13; b0286.3; rpt-6; r07e5.3; ugt-1; bre-1; hex-1; cdk-7; cct-2; m02d8.4; rpl-11.1; k08f4.1; lin-2; c01f1.3; f42g10.1; c26b9.5; max-2; pph-5; ubc-9; gpd-2; ife-3; daf-7; lon-1; c10c5.5; t19b4.3; elt-3; hpl-2; pah-1; hil-3; eri-1; pha-4; lsm-1; rps-22; tbg-1; lap-1; cye-1; w09d10.3; ceh-38; ace-3; y69f12a.1; gly-14; zc204.2; tgt-1; k01g5.5; mdl-1; mxl-1; k04a8.5; c10c5.4; h04m03.1; unc-130; vab-3; aha-1; f54d10.7; jnk-1; zk1127.10; w06e11.1; snr-2; rpl-25.2; skr-15; nuc-1; vab-15; y19d10b.6; asp-4; cpar-1; his-7; mdt-6; f40f8.1; cnd-1; fkb-3; gsk-3; cyn-3; ubc-20; tba-1; y43c5b.2; pmk-1; c37a2.7; c44h4.6; his-48; cul-2; b0524.2; c18a3.1; lys-8; c39d10.7; ars-2; pfd-3; c29f9.5; cul-1; ufd-1; f21f3.2; rab-10; ceh-23; mpk-1; snr-3; hlh-8; bre-2; sbp-1; fem-2* | 0.0303 |
| GO:0045927 | positive regulation of growth | *hlh-1; his-13; col-94; snt-2; mdt-15; cyh-1; cogc-1; w06e11.1; f10e9.7; rpl-11.1; rpl-25.2; skr-15; r06a4.9; his-7; t04g9.4; ubc-9; unc-32; gsk-3; pnk-1; lon-1; k08e3.5; y65b4br.5; pha-4; rps-22; k07c5.4; c37a2.7; nlp-40; w09d10.3; f10e9.4; his-48; w07e6.2; sna-2; k01g5.5; c01a2.5; evl-20; ars-2; pfd-3; f55f8.2; sdc-3; ufd-1; rab-10; f25h2.2; f23h12.2; f25h9.6; sec-8; ajm-1; t10h9.3; sbp-1; f48c1.4; aha-1* | 0.0345 |
| GO:0018993 | somatic sex determination | *fog-3; tra-1; fem-2* | 0.0345 |
| GO:0040008 | regulation of growth | *hlh-1; his-13; col-94; snt-2; mdt-15; cyh-1; cogc-1; w06e11.1; f10e9.7; rpl-11.1; rpl-25.2; skr-15; r06a4.9; his-7; t04g9.4; ubc-9; unc-32; gsk-3; pnk-1; lon-1; k08e3.5; y65b4br.5; pha-4; rps-22; k07c5.4; c37a2.7; nlp-40; w09d10.3; f10e9.4; his-48; w07e6.2; sna-2; k01g5.5; c01a2.5; evl-20; ars-2; pfd-3; f55f8.2; sdc-3; cul-1; ufd-1; rab-10; f25h2.2; f23h12.2; f25h9.6; sec-8; ajm-1; t10h9.3; sbp-1; f48c1.4; aha-1* | 0.0393 |
| GO:0019953 | sexual reproduction | *aos-1; k07c5.4; cye-1; nlp-40; cyh-1; w06e11.1; cul-2; k01g5.5; rpl-11.1; tag-125; rpl-25.2; evl-20; pfd-3; f55f8.2; ubc-9; emo-1; f43g9.5; tra-1; gpc-2; f25h9.6; ssl-1; csn-5; pfn-1; tba-1; fog-3; sbp-1; y65b4br.5; rps-22* | 0.0393 |
| GO:0051276 | chromosome organization and biogenesis | *cpar-1; tbg-1; his-13; his-7; sdc-3; r07e5.3; his-48; cul-2; hpl-2; hil-3* | 0.0393 |
| GO:0006139 | nucleobase, nucleoside, nucleotide and nucleic acid metabolic process | *hlh-1; his-13; b0286.3; r07e5.3; bre-1; w06e11.1; cdk-7; snr-2; k08f4.1; skr-15; nuc-1; vab-15; cpar-1; mdt-6; his-7; f40f8.1; cnd-1; daf-7; lon-1; t19b4.3; elt-3; hpl-2; hil-3; eri-1; pha-4; lsm-1; cye-1; ceh-38; his-48; tgt-1; zc204.2; c18a3.1; mdl-1; k01g5.5; mxl-1; ars-2; c29f9.5; unc-130; cul-1; vab-3; rab-10; ceh-23; snr-3; hlh-8; sbp-1; aha-1* | 0.0426 |
| GO:0000819 | sister chromatid segregation | *tbg-1; sdc-3; cul-2* | 0.0477 |
| GO:0008104 | protein localization | *unc-64; unc-130; rab-10; emo-1; f23h12.2; rab-28; rap-1; sec-8; cul-2; rab-37; tomm-7; evl-20* | 0.0519 |
| GO:0006886 | intracellular protein transport | *unc-64; rab-10; emo-1; f23h12.2; rab-28; rap-1; rab-37; tomm-7; evl-20* | 0.0519 |
| GO:0043058 | regulation of backward locomotion | *unc-49; ace-3* | 0.0541 |
| GO:0033036 | macromolecule localization | *unc-64; unc-130; rab-10; emo-1; f23h12.2; rab-28; rap-1; sec-8; cul-2; rab-37; tomm-7; evl-20* | 0.0573 |
| GO:0050794 | regulation of cellular process | *hlh-1; cogc-1; cyd-1; vab-15; asp-4; mdt-6; unc-32; cki-1; cnd-1; tra-1; daf-7; lon-1; elt-3; r02f2.1; hpl-2; pha-4; cye-1; ceh-38; cul-2; zc204.2; tag-218; mdl-1; mxl-1; unc-130; c29f9.5; ins-18; vab-3; cul-1; rab-10; ceh-23; hlh-8; apc-10; fog-3; sbp-1; aha-1* | 0.0573 |
| GO:0022403 | cell cycle phase | *tbg-1; sdc-3; apc-10; fog-3; cye-1; eri-1; cul-2* | 0.0573 |
| GO:0007276 | gamete generation | *aos-1; k07c5.4; cye-1; nlp-40; cyh-1; w06e11.1; cul-2; k01g5.5; rpl-11.1; tag-125; rpl-25.2; evl-20; pfd-3; f55f8.2; ubc-9; emo-1; f43g9.5; tra-1; f25h9.6; ssl-1; csn-5; pfn-1; fog-3; sbp-1; y65b4br.5; rps-22* | 0.0573 |
| GO:0007530 | sex determination | *sdc-3; fog-3; tra-1; fem-2* | 0.0645 |
| GO:0051656 | establishment of organelle localization | *tba-1; gpc-2; gsk-3; y65b4br.5; csn-5; pfd-3; cul-2* | 0.0687 |
| GO:0045944 | positive regulation of transcription from RNA polymerase II promoter | *vab-3; sbp-1; daf-7* | 0.0692 |
| GO:0006400 | tRNA modification | *tgt-1; k01g5.5* | 0.0693 |
| GO:0051640 | organelle localization | *tba-1; gpc-2; gsk-3; y65b4br.5; csn-5; pfd-3; cul-2* | 0.0725 |
| GO:0045893 | positive regulation of transcription, DNA-dependent | *vab-3; sbp-1; daf-7* | 0.0729 |
| GO:0007051 | spindle organization and biogenesis | *tbg-1; tba-1; gsk-3; sur-6; cul-2* | 0.0735 |
| GO:0007626 | locomotory behavior | *hlh-1; col-94; rpt-6; mdt-15; cyh-1; cogc-1; w06e11.1; cyd-1; r06a4.9; vab-15; sur-6; c01f1.3; his-7; t04g9.4; ubc-9; unc-32; cki-1; unc-49; y65b4br.5; pha-4; nxt-1; his-48; c01a2.5; evl-20; r05f9.1; ars-2; ufd-1; t02g5.7; sec-8; pfn-1; ajm-1; t10h9.3; aha-1; f48c1.4* | 0.0735 |
| GO:0015031 | protein transport | *unc-64; rab-10; emo-1; f23h12.2; rab-28; rap-1; sec-8; rab-37; tomm-7; evl-20* | 0.0779 |
| GO:0043057 | backward locomotion | *unc-49; ace-3* | 0.0844 |
| GO:0042003 | masculinization of hermaphrodite soma | *fog-3; fem-2* | 0.0844 |
| GO:0006357 | regulation of transcription from RNA polymerase II promoter | *hlh-1; vab-3; sbp-1; daf-7* | 0.09 |
| GO:0045941 | positive regulation of transcription | *vab-3; sbp-1; daf-7* | 0.0937 |
| GO:0045184 | establishment of protein localization | *unc-64; rab-10; emo-1; f23h12.2; rab-28; rap-1; sec-8; rab-37; tomm-7; evl-20* | 0.0951 |
| GO:0045935 | positive regulation of nucleobase, nucleoside, nucleotide and nucleic acid metabolic process | *vab-3; sbp-1; daf-7* | 0.098 |
| GO:0031325 | positive regulation of cellular metabolic process | *vab-3; sbp-1; daf-7* | 0.098 |
| GO:0048468 | cell development | *pmk-1; aos-1; unc-130; cnd-1; emo-1; unc-32; unc-129; tra-1; cye-1; cul-2; nuc-1; asp-4* | 0.1 |
| GO:0043071 | positive regulation of non-apoptotic programmed cell death | *unc-32; asp-4* | 0.1 |
| GO:0043283 | biopolymer metabolic process | *hlh-1; his-13; jnk-1; rpt-6; r07e5.3; ugt-1; cdk-7; snr-2; k08f4.1; skr-15; nuc-1; vab-15; lin-2; cpar-1; c01f1.3; his-7; max-2; pph-5; ubc-9; gsk-3; daf-7; ubc-20; lon-1; elt-3; hpl-2; hil-3; eri-1; pha-4; lsm-1; y43c5b.2; pmk-1; c37a2.7; c44h4.6; cye-1; ceh-38; y69f12a.1; his-48; cul-2; gly-14; zc204.2; tgt-1; k01g5.5; mxl-1; c39d10.7; ars-2; c29f9.5; unc-130; f21f3.2; ufd-1; vab-3; cul-1; rab-10; ceh-23; mpk-1; snr-3; bre-2; sbp-1; aha-1; f54d10.7; fem-2* | 0.1 |
| GO:0043068 | positive regulation of programmed cell death | *unc-32; tra-1; asp-4* | 0.104 |
| GO:0007610 | behavior | *hlh-1; col-94; rpt-6; mdt-15; r07e5.3; cyh-1; cogc-1; w06e11.1; cyd-1; r06a4.9; vab-15; lin-2; sur-6; c01f1.3; his-7; ubc-9; t04g9.4; cnd-1; cki-1; unc-32; unc-49; skp-1; y65b4br.5; pha-4; nxt-1; his-48; c01a2.5; evl-20; r05f9.1; ars-2; ufd-1; t02g5.7; sec-8; pfn-1; t10h9.3; ajm-1; hlh-8; aha-1; f48c1.4* | 0.106 |
| GO:0000279 | M phase | *tbg-1; sdc-3; apc-10; fog-3; eri-1; cul-2* | 0.108 |
| GO:0006325 | establishment and/or maintenance of chromatin architecture | *cpar-1; his-13; his-7; hpl-2; hil-3; r07e5.3; his-48* | 0.115 |
| GO:0006323 | DNA packaging | *cpar-1; his-13; his-7; hpl-2; hil-3; r07e5.3; his-48* | 0.115 |
| GO:0033058 | directional locomotion | *unc-49; ace-3* | 0.115 |
| GO:0042006 | masculinization of hermaphroditic germ-line | *fog-3; fem-2* | 0.115 |
| GO:0009893 | positive regulation of metabolic process | *vab-3; sbp-1; daf-7* | 0.116 |
| GO:0009653 | anatomical structure morphogenesis | *hlh-1; his-13; col-94; cye-1; cyh-1; cogc-1; snr-2; c01a2.5; evl-20; sur-6; pfd-3; cpar-1; unc-130; ubc-9; sdc-3; cul-1; vab-3; emo-1; cnd-1; cki-1; unc-129; tra-1; t02g5.7; sec-8; sbp-1; f48c1.4* | 0.116 |
| GO:0007067 | mitosis | *tbg-1; sdc-3; apc-10* | 0.116 |
| GO:0000910 | cytokinesis | *tbg-1; tba-1; cye-1; evl-20; csn-5; sur-6; pfn-1* | 0.116 |
| GO:0006366 | transcription from RNA polymerase II promoter | *hlh-1; vab-3; sbp-1; daf-7* | 0.117 |
| GO:0007409 | axonogenesis | *unc-130; cnd-1; unc-129* | 0.117 |
| GO:0007059 | chromosome segregation | *tbg-1; sdc-3; y65b4br.5; eri-1; c34b2.4; cul-2* | 0.117 |
| GO:0030154 | cell differentiation | *pmk-1; aos-1; unc-130; vab-3; cnd-1; emo-1; unc-32; unc-129; tra-1; cye-1; cul-2; hpl-2; nuc-1; asp-4* | 0.117 |
| GO:0046165 | alcohol biosynthetic process | *h04m03.1; bre-1* | 0.117 |
| GO:0046364 | monosaccharide biosynthetic process | *h04m03.1; bre-1* | 0.117 |
| GO:0042001 | hermaphrodite somatic sex determination | *fog-3; fem-2* | 0.117 |
| GO:0019319 | hexose biosynthetic process | *h04m03.1; bre-1* | 0.117 |
| GO:0048869 | cellular developmental process | *pmk-1; aos-1; unc-130; vab-3; cnd-1; emo-1; unc-32; unc-129; tra-1; cye-1; cul-2; hpl-2; nuc-1; asp-4* | 0.117 |
| GO:0040026 | positive regulation of vulval development | *cye-1; lin-2; sur-6* | 0.117 |
| GO:0046083 | adenine metabolic process | *t19b4.3* | 0.117 |
| GO:0006168 | adenine salvage | *t19b4.3* | 0.117 |
| GO:0043525 | positive regulation of neuron apoptosis | *tra-1* | 0.117 |
| GO:0048145 | regulation of fibroblast proliferation | *mdl-1* | 0.117 |
| GO:0051232 | meiotic spindle elongation | *cul-2* | 0.117 |
| GO:0043096 | purine base salvage | *t19b4.3* | 0.117 |
| GO:0009047 | dosage compensation, by hyperactivation of X chromosome | *sdc-3* | 0.117 |
| GO:0007020 | microtubule nucleation | *tbg-1* | 0.117 |
| GO:0012503 | induction of non-apoptotic programmed cell death | *asp-4* | 0.117 |
| GO:0048147 | negative regulation of fibroblast proliferation | *mdl-1* | 0.117 |
| GO:0007214 | gamma-aminobutyric acid signaling pathway | *unc-49* | 0.117 |
| GO:0035188 | hatching | *ace-3* | 0.117 |
| GO:0048144 | fibroblast proliferation | *mdl-1* | 0.117 |
| GO:0051291 | protein heterooligomerization | *hlh-1* | 0.117 |
| GO:0016457 | dosage compensation complex assembly during dosage compensation by hyperactivation of X chromosome | *sdc-3* | 0.117 |
| GO:0042714 | dosage compensation complex assembly | *sdc-3* | 0.117 |
| GO:0045737 | positive regulation of cyclin-dependent protein kinase activity | *cye-1* | 0.117 |
| GO:0048846 | axon extension involved in axon guidance | *unc-130* | 0.117 |
| GO:0032876 | negative regulation of DNA endoreduplication | *lon-1* | 0.117 |
| GO:0043523 | regulation of neuron apoptosis | *tra-1* | 0.117 |
| GO:0015677 | copper ion import | *cuc-1* | 0.117 |
| GO:0000070 | mitotic sister chromatid segregation | *tbg-1; sdc-3* | 0.117 |
| GO:0045786 | negative regulation of progression through cell cycle | *cul-1; cki-1* | 0.117 |
| GO:0008286 | insulin receptor signaling pathway | *ins-18; daf-16* | 0.117 |
| GO:0065004 | protein-DNA complex assembly | *cpar-1; his-13; his-7; sdc-3; hil-3; his-48* | 0.121 |
| GO:0040025 | vulval development | *ubc-9; hpl-2; cye-1; cogc-1; lin-2; sur-6; mpk-1* | 0.124 |
| GO:0065008 | regulation of biological quality | *gsto-3; cul-1; nhx-6; rab-10; emo-1; mdt-15; tra-1; cye-1; ftn-2; t10h9.3; skp-1; snr-3; sbp-1* | 0.124 |
| GO:0051647 | nucleus localization | *tba-1; gpc-2; y65b4br.5; pfd-3* | 0.124 |
| GO:0040023 | establishment of nucleus localization | *tba-1; gpc-2; y65b4br.5; pfd-3* | 0.124 |
| GO:0050896 | response to stimulus | *hlh-1; jnk-1; col-94; rpt-6; mdt-15; r07e5.3; cyh-1; cogc-1; w06e11.1; cyd-1; skr-15; k08f4.1; r06a4.9; vab-15; lin-2; sur-6; c01f1.3; his-7; ubc-9; t04g9.4; cnd-1; cki-1; unc-32; unc-49; skp-1; y65b4br.5; pha-4; nxt-1; pmk-1; his-48; c01a2.5; r05f9.1; evl-20; ars-2; cul-1; ufd-1; t02g5.7; sec-8; pfn-1; t10h9.3; ajm-1; hlh-8; f48c1.4; aha-1* | 0.124 |
| GO:0048667 | neuron morphogenesis during differentiation | *unc-130; cnd-1; unc-129* | 0.124 |
| GO:0000087 | M phase of mitotic cell cycle | *tbg-1; sdc-3; apc-10* | 0.124 |
| GO:0000904 | cellular morphogenesis during differentiation | *unc-130; cnd-1; unc-129* | 0.124 |
| GO:0048812 | neurite morphogenesis | *unc-130; cnd-1; unc-129* | 0.124 |
| GO:0006996 | organelle organization and biogenesis | *tbg-1; his-13; r07e5.3; his-48; cul-2; dlc-2; nuc-1; sur-6; cpar-1; his-7; sdc-3; gsk-3; csn-5; pfn-1; tba-1; hpl-2; hil-3* | 0.124 |
| GO:0000226 | microtubule cytoskeleton organization and biogenesis | *tbg-1; tba-1; gsk-3; csn-5; sur-6; cul-2* | 0.124 |
| GO:0006970 | response to osmotic stress | *pmk-1; jnk-1* | 0.124 |
| GO:0008361 | regulation of cell size | *cul-1; emo-1* | 0.124 |
| GO:0006972 | hyperosmotic response | *pmk-1; jnk-1* | 0.124 |
| GO:0051234 | establishment of localization | *col-89; t05e7.3; col-94; r11.1; y57g11c.23; snt-2; cle-1; cuc-1; k07h8.2; col-91; sedl-1; h17b01.1; lbp-7; b0281.5; nhx-6; unc-32; t23b12.6; rab-28; gpc-2; gsk-3; rap-1; unc-49; ftn-2; tba-1; y65b4br.5; c51e3.6; lbp-1; nxt-1; unc-64; tbg-1; y40b1b.8; cul-2; glc-2; r05f9.1; evl-20; pfd-3; rab-10; c50d2.2; emo-1; f23h12.2; csn-5; sec-8; y51b9a.6; rab-37; tomm-7* | 0.126 |
| GO:0006259 | DNA metabolic process | *cpar-1; his-13; his-7; cul-1; cye-1; r07e5.3; his-48; lon-1; hpl-2; skr-15; k08f4.1; hil-3; nuc-1* | 0.131 |
| GO:0048878 | chemical homeostasis | *nhx-6; rab-10; mdt-15; tra-1; cye-1; ftn-2; t10h9.3; skp-1; snr-3; sbp-1* | 0.135 |
| GO:0051094 | positive regulation of developmental process | *cye-1; lin-2; sur-6* | 0.136 |
| GO:0031175 | neurite development | *unc-130; cnd-1; unc-129* | 0.136 |
| GO:0043070 | regulation of non-apoptotic programmed cell death | *unc-32; asp-4* | 0.136 |
| GO:0006333 | chromatin assembly or disassembly | *cpar-1; his-13; his-7; hpl-2; hil-3; his-48* | 0.136 |
| GO:0006913 | nucleocytoplasmic transport | *rab-37; rab-10; rab-28; rap-1* | 0.139 |
| GO:0051169 | nuclear transport | *rab-37; rab-10; rab-28; rap-1* | 0.139 |
| GO:0000902 | cell morphogenesis | *unc-130; cul-1; cnd-1; emo-1; unc-129* | 0.141 |
| GO:0032989 | cellular structure morphogenesis | *unc-130; cul-1; cnd-1; emo-1; unc-129* | 0.141 |
| GO:0051301 | cell division | *tbg-1; cul-1; cye-1; gsk-3; csn-5; pfn-1; cul-2; tba-1; evl-20; sur-6* | 0.142 |
| GO:0007281 | germ cell development | *aos-1; emo-1; cye-1; cul-2* | 0.142 |
| GO:0009059 | macromolecule biosynthetic process | *c01f1.3; c37a2.7; t04g9.4; ife-3; w09d10.3; bre-1; gly-14; rpl-11.1; rpl-25.2; bre-2; h04m03.1; rps-22; ars-2* | 0.142 |
| GO:0006334 | nucleosome assembly | *cpar-1; his-13; his-7; hil-3; his-48* | 0.142 |
| GO:0012501 | programmed cell death | *pmk-1; unc-32; tra-1; nuc-1; asp-4* | 0.142 |
| GO:0016244 | non-apoptotic programmed cell death | *unc-32; asp-4* | 0.144 |
| GO:0042127 | regulation of cell proliferation | *mdl-1; cye-1* | 0.144 |
| GO:0009451 | RNA modification | *tgt-1; k01g5.5* | 0.144 |
| GO:0031497 | chromatin assembly | *cpar-1; his-13; his-7; hil-3; his-48* | 0.144 |
| GO:0007017 | microtubule-based process | *tbg-1; tba-1; dlc-2; gsk-3; csn-5; sur-6; cul-2* | 0.144 |
| GO:0009163 | nucleoside biosynthetic process | *tgt-1* | 0.144 |
| GO:0019673 | GDP-mannose metabolic process | *bre-1* | 0.144 |
| GO:0046129 | purine ribonucleoside biosynthetic process | *tgt-1* | 0.144 |
| GO:0008618 | 7-methylguanosine metabolic process | *tgt-1* | 0.144 |
| GO:0048815 | hermaphrodite genitalia morphogenesis | *cogc-1* | 0.144 |
| GO:0031119 | tRNA pseudouridine synthesis | *k01g5.5* | 0.144 |
| GO:0008617 | guanosine metabolic process | *tgt-1* | 0.144 |
| GO:0008156 | negative regulation of DNA replication | *lon-1* | 0.144 |
| GO:0009299 | mRNA transcription | *cdk-7* | 0.144 |
| GO:0051231 | spindle elongation | *cul-2* | 0.144 |
| GO:0008616 | queuosine biosynthetic process | *tgt-1* | 0.144 |
| GO:0030071 | regulation of mitotic metaphase/anaphase transition | *apc-10* | 0.144 |
| GO:0046118 | 7-methylguanosine biosynthetic process | *tgt-1* | 0.144 |
| GO:0032875 | regulation of DNA endoreduplication | *lon-1* | 0.144 |
| GO:0030522 | intracellular receptor-mediated signaling pathway | *aha-1* | 0.144 |
| GO:0001555 | oocyte growth | *emo-1* | 0.144 |
| GO:0000082 | G1/S transition of mitotic cell cycle | *cye-1* | 0.144 |
| GO:0006419 | alanyl-tRNA aminoacylation | *ars-2* | 0.144 |
| GO:0050879 | multicellular organismal movement | *ace-3* | 0.144 |
| GO:0006558 | L-phenylalanine metabolic process | *pah-1* | 0.144 |
| GO:0015937 | coenzyme A biosynthetic process | *pnk-1* | 0.144 |
| GO:0046116 | queuosine metabolic process | *tgt-1* | 0.144 |
| GO:0045448 | mitotic cell cycle, embryonic | *cdk-7* | 0.144 |
| GO:0048601 | oocyte morphogenesis | *emo-1* | 0.144 |
| GO:0042451 | purine nucleoside biosynthetic process | *tgt-1* | 0.144 |
| GO:0046114 | guanosine biosynthetic process | *tgt-1* | 0.144 |
| GO:0007258 | JUN phosphorylation | *jnk-1* | 0.144 |
| GO:0042455 | ribonucleoside biosynthetic process | *tgt-1* | 0.144 |
| GO:0006559 | L-phenylalanine catabolic process | *pah-1* | 0.144 |
| GO:0019101 | female somatic sex determination | *tra-1* | 0.144 |
| GO:0046627 | negative regulation of insulin receptor signaling pathway | *ins-18* | 0.144 |
| GO:0009410 | response to xenobiotic stimulus | *aha-1* | 0.144 |
| GO:0040028 | regulation of vulval development | *ubc-9; hpl-2; cye-1; lin-2; sur-6* | 0.147 |
| GO:0016051 | carbohydrate biosynthetic process | *c01f1.3; h04m03.1; bre-1* | 0.148 |
| GO:0009116 | nucleoside metabolic process | *tgt-1; t19b4.3* | 0.149 |
| GO:0007411 | axon guidance | *unc-130; unc-129* | 0.149 |
| GO:0000132 | establishment of mitotic spindle orientation | *tba-1; gsk-3; csn-5* | 0.176 |
| GO:0051294 | establishment of spindle orientation | *tba-1; gsk-3; csn-5* | 0.176 |
| GO:0042592 | homeostatic process | *gsto-3; nhx-6; rab-10; mdt-15; tra-1; cye-1; ftn-2; t10h9.3; skp-1; snr-3; sbp-1* | 0.179 |
| GO:0065003 | macromolecular complex assembly | *hlh-1; cpar-1; his-13; his-7; sdc-3; ife-3; hil-3; his-48* | 0.179 |
| GO:0040021 | hermaphrodite germ-line sex determination | *fog-3; fem-2* | 0.179 |
| GO:0000041 | transition metal ion transport | *cuc-1; ftn-2* | 0.179 |
| GO:0042353 | fucose biosynthetic process | *bre-1* | 0.179 |
| GO:0008054 | cyclin catabolic process | *cul-2* | 0.179 |
| GO:0051259 | protein oligomerization | *hlh-1* | 0.179 |
| GO:0009226 | nucleotide-sugar biosynthetic process | *bre-1* | 0.179 |
| GO:0051402 | neuron apoptosis | *tra-1* | 0.179 |
| GO:0019439 | aromatic compound catabolic process | *pah-1* | 0.179 |
| GO:0001507 | acetylcholine catabolic process in synaptic cleft | *ace-3* | 0.179 |
| GO:0001666 | response to hypoxia | *unc-32* | 0.179 |
| GO:0000079 | regulation of cyclin-dependent protein kinase activity | *cye-1* | 0.179 |
| GO:0042354 | L-fucose metabolic process | *bre-1* | 0.179 |
| GO:0042698 | menstrual cycle | *emo-1* | 0.179 |
| GO:0043101 | purine salvage | *t19b4.3* | 0.179 |
| GO:0006005 | L-fucose biosynthetic process | *bre-1* | 0.179 |
| GO:0001539 | ciliary or flagellar motility | *tnc-2* | 0.179 |
| GO:0009225 | nucleotide-sugar metabolic process | *bre-1* | 0.179 |
| GO:0001542 | ovulation from ovarian follicle | *emo-1* | 0.179 |
| GO:0030237 | female sex determination | *tra-1* | 0.179 |
| GO:0007091 | mitotic metaphase/anaphase transition | *apc-10* | 0.179 |
| GO:0046368 | GDP-L-fucose metabolic process | *bre-1* | 0.179 |
| GO:0042350 | GDP-L-fucose biosynthetic process | *bre-1* | 0.179 |
| GO:0043094 | metabolic compound salvage | *t19b4.3* | 0.179 |
| GO:0022602 | menstrual cycle process | *emo-1* | 0.179 |
| GO:0009074 | aromatic amino acid family catabolic process | *pah-1* | 0.179 |
| GO:0006004 | fucose metabolic process | *bre-1* | 0.179 |
| GO:0022601 | menstrual cycle phase | *emo-1* | 0.179 |
| GO:0006144 | purine base metabolic process | *t19b4.3* | 0.179 |
| GO:0048523 | negative regulation of cellular process | *lon-1; ins-18; cul-1; mdl-1; cki-1* | 0.179 |
| GO:0048666 | neuron development | *unc-130; cnd-1; unc-129* | 0.18 |
| GO:0018988 | molting cycle, protein-based cuticle | *f55f8.2; skp-1; rab-10; rpl-25.2; y65b4br.5* | 0.183 |
| GO:0008283 | cell proliferation | *mdl-1; cye-1* | 0.188 |
| GO:0042303 | molting cycle | *f55f8.2; skp-1; rab-10; rpl-25.2; y65b4br.5* | 0.188 |
| GO:0040016 | embryonic cleavage | *tbg-1; cye-1; gsk-3; csn-5; pfn-1; tba-1; evl-20; sur-6* | 0.19 |
| GO:0043067 | regulation of programmed cell death | *unc-32; tra-1; asp-4* | 0.195 |
| GO:0040006 | protein-based cuticle attachment to epithelium | *f55f8.2; rab-10; rpl-25.2* | 0.195 |
| GO:0040004 | collagen and cuticulin-based cuticle attachment to epithelium | *f55f8.2; rab-10; rpl-25.2* | 0.195 |
| GO:0006520 | amino acid metabolic process | *c10c5.5; zk1127.10; m02d8.4; c10c5.4; pah-1; ars-2* | 0.195 |
| GO:0030104 | water homeostasis | *rab-10; mdt-15; tra-1; cye-1; t10h9.3; skp-1; snr-3; sbp-1* | 0.197 |
| GO:0018987 | osmoregulation | *rab-10; mdt-15; tra-1; cye-1; t10h9.3; skp-1; snr-3; sbp-1* | 0.199 |
| GO:0050878 | regulation of body fluid levels | *rab-10; mdt-15; tra-1; cye-1; t10h9.3; skp-1; snr-3; sbp-1* | 0.2 |
| GO:0035046 | pronuclear migration | *tba-1; gpc-2; pfd-3* | 0.2 |
| GO:0009952 | anterior/posterior pattern formation | *vab-3; cul-2* | 0.2 |
| GO:0007138 | meiotic anaphase II | *cul-2* | 0.2 |
| GO:0046128 | purine ribonucleoside metabolic process | *tgt-1* | 0.2 |
| GO:0046040 | IMP metabolic process | *b0286.3* | 0.2 |
| GO:0042278 | purine nucleoside metabolic process | *tgt-1* | 0.2 |
| GO:0006189 | 'de novo' IMP biosynthetic process | *b0286.3* | 0.2 |
| GO:0045787 | positive regulation of progression through cell cycle | *cye-1* | 0.2 |
| GO:0006879 | cellular iron ion homeostasis | *ftn-2* | 0.2 |
| GO:0006826 | iron ion transport | *ftn-2* | 0.2 |
| GO:0006188 | IMP biosynthetic process | *b0286.3* | 0.2 |
| GO:0045144 | meiotic sister chromatid segregation | *cul-2* | 0.2 |
| GO:0042135 | neurotransmitter catabolic process | *ace-3* | 0.2 |
| GO:0043161 | proteasomal ubiquitin-dependent protein catabolic process | *cul-2* | 0.2 |
| GO:0007088 | regulation of mitosis | *apc-10* | 0.2 |
| GO:0006275 | regulation of DNA replication | *lon-1* | 0.2 |
| GO:0051322 | anaphase | *cul-2* | 0.2 |
| GO:0006581 | acetylcholine catabolic process | *ace-3* | 0.2 |
| GO:0006528 | asparagine metabolic process | *m02d8.4* | 0.2 |
| GO:0051759 | sister chromosome movement towards spindle pole during meiosis II | *cul-2* | 0.2 |
| GO:0051235 | maintenance of localization | *unc-130* | 0.2 |
| GO:0007135 | meiosis II | *cul-2* | 0.2 |
| GO:0006529 | asparagine biosynthetic process | *m02d8.4* | 0.2 |
| GO:0045930 | negative regulation of progression through mitotic cell cycle | *cul-1* | 0.2 |
| GO:0007413 | axonal fasciculation | *cnd-1* | 0.2 |
| GO:0045185 | maintenance of protein localization | *unc-130* | 0.2 |
| GO:0008585 | female gonad development | *emo-1* | 0.2 |
| GO:0055072 | iron ion homeostasis | *ftn-2* | 0.2 |
| GO:0022607 | cellular component assembly | *hlh-1; cpar-1; his-13; his-7; sdc-3; ife-3; hil-3; his-48* | 0.201 |
| GO:0006457 | protein folding | *fkb-3; cct-2; cyn-3; pfd-3* | 0.204 |
| GO:0006519 | amino acid and derivative metabolic process | *c10c5.5; zk1127.10; m02d8.4; c10c5.4; pah-1; ace-3; ars-2* | 0.204 |
| GO:0048858 | cell projection morphogenesis | *unc-130; cnd-1; unc-129* | 0.204 |
| GO:0040024 | dauer larval development | *lgg-1; daf-7* | 0.204 |
| GO:0007097 | nuclear migration | *tba-1; gpc-2; pfd-3* | 0.204 |
| GO:0032990 | cell part morphogenesis | *unc-130; cnd-1; unc-129* | 0.204 |
| GO:0030030 | cell projection organization and biogenesis | *unc-130; cnd-1; unc-129* | 0.204 |
| GO:0007010 | cytoskeleton organization and biogenesis | *tbg-1; gsk-3; csn-5; pfn-1; cul-2; tba-1; dlc-2; sur-6* | 0.21 |
| GO:0019318 | hexose metabolic process | *gpd-2; h04m03.1; bre-1* | 0.21 |
| GO:0022404 | molting cycle process | *f55f8.2; rab-10; rpl-25.2* | 0.21 |
| GO:0018996 | molting cycle, collagen and cuticulin-based cuticle | *f55f8.2; rab-10; rpl-25.2* | 0.21 |
| GO:0051258 | protein polymerization | *tbg-1; tba-1* | 0.211 |
| GO:0045132 | meiotic chromosome segregation | *eri-1; cul-2* | 0.211 |
| GO:0018992 | germ-line sex determination | *fog-3; fem-2* | 0.211 |
| GO:0007264 | small GTPase mediated signal transduction | *tag-218; rab-37; rab-10; rab-28; rap-1; evl-20; mpk-1* | 0.211 |
| GO:0005996 | monosaccharide metabolic process | *gpd-2; h04m03.1; bre-1* | 0.216 |
| GO:0048609 | reproductive process in a multicellular organism | *c01f1.3; cnd-1; emo-1; cki-1; r07e5.3; skp-1; hlh-8; eri-1; f48c1.4; sur-6; lin-2* | 0.22 |
| GO:0032504 | multicellular organism reproduction | *c01f1.3; cnd-1; emo-1; cki-1; r07e5.3; skp-1; hlh-8; eri-1; f48c1.4; sur-6; lin-2* | 0.22 |
| GO:0030182 | neuron differentiation | *unc-130; cnd-1; unc-129* | 0.22 |
| GO:0008285 | negative regulation of cell proliferation | *mdl-1* | 0.22 |
| GO:0016344 | meiotic chromosome movement towards spindle pole | *cul-2* | 0.22 |
| GO:0006094 | gluconeogenesis | *h04m03.1* | 0.22 |
| GO:0051053 | negative regulation of DNA metabolic process | *lon-1* | 0.22 |
| GO:0009067 | aspartate family amino acid biosynthetic process | *m02d8.4* | 0.22 |
| GO:0008291 | acetylcholine metabolic process | *ace-3* | 0.22 |
| GO:0042402 | biogenic amine catabolic process | *ace-3* | 0.22 |
| GO:0045087 | innate immune response | *pmk-1* | 0.22 |
| GO:0030155 | regulation of cell adhesion | *vab-3* | 0.22 |
| GO:0015936 | coenzyme A metabolic process | *pnk-1* | 0.22 |
| GO:0051305 | chromosome movement towards spindle pole | *cul-2* | 0.22 |
| GO:0042439 | ethanolamine and derivative metabolic process | *ace-3* | 0.22 |
| GO:0042219 | amino acid derivative catabolic process | *ace-3* | 0.22 |
| GO:0006261 | DNA-dependent DNA replication | *lon-1; cye-1* | 0.226 |
| GO:0007169 | transmembrane receptor protein tyrosine kinase signaling pathway | *ins-18; daf-16* | 0.226 |
| GO:0010467 | gene expression | *hlh-1; w06e11.1; cdk-7; rpl-11.1; rpl-25.2; vab-15; mdt-6; cnd-1; ife-3; daf-7; elt-3; eri-1; pha-4; rps-22; c37a2.7; w09d10.3; ceh-38; tgt-1; zc204.2; k01g5.5; mdl-1; mxl-1; ars-2; unc-130; c29f9.5; vab-3; sdc-3; rab-10; ceh-23; hlh-8; sbp-1; aha-1* | 0.23 |
| GO:0043284 | biopolymer biosynthetic process | *c01f1.3; c37a2.7* | 0.236 |
| GO:0051293 | establishment of spindle localization | *tba-1; gsk-3; csn-5* | 0.236 |
| GO:0040001 | establishment of mitotic spindle localization | *tba-1; gsk-3; csn-5* | 0.236 |
| GO:0051653 | spindle localization | *tba-1; gsk-3; csn-5* | 0.236 |
| GO:0016071 | mRNA metabolic process | *snr-3; snr-2; lsm-1* | 0.236 |
| GO:0007167 | enzyme linked receptor protein signaling pathway | *lon-1; ins-18; daf-16* | 0.236 |
| GO:0009310 | amine catabolic process | *pah-1; ace-3* | 0.239 |
| GO:0044270 | nitrogen compound catabolic process | *pah-1; ace-3* | 0.239 |
| GO:0006013 | mannose metabolic process | *bre-1* | 0.239 |
| GO:0007050 | cell cycle arrest | *cki-1* | 0.239 |
| GO:0046626 | regulation of insulin receptor signaling pathway | *ins-18* | 0.239 |
| GO:0009066 | aspartate family amino acid metabolic process | *m02d8.4* | 0.239 |
| GO:0006801 | superoxide metabolic process | *sod-5* | 0.239 |
| GO:0006800 | oxygen and reactive oxygen species metabolic process | *sod-5* | 0.239 |
| GO:0007351 | tripartite regional subdivision | *cul-2* | 0.239 |
| GO:0006090 | pyruvate metabolic process | *h04m03.1* | 0.239 |
| GO:0009119 | ribonucleoside metabolic process | *tgt-1* | 0.239 |
| GO:0048511 | rhythmic process | *emo-1* | 0.239 |
| GO:0007052 | mitotic spindle organization and biogenesis | *tbg-1* | 0.239 |
| GO:0051303 | establishment of chromosome localization | *cul-2* | 0.239 |
| GO:0006338 | chromatin remodeling | *r07e5.3* | 0.239 |
| GO:0008595 | determination of anterior/posterior axis, embryo | *cul-2* | 0.239 |
| GO:0016049 | cell growth | *emo-1* | 0.239 |
| GO:0050000 | chromosome localization | *cul-2* | 0.239 |
| GO:0000212 | meiotic spindle organization and biogenesis | *cul-2* | 0.239 |
| GO:0046545 | development of primary female sexual characteristics | *emo-1* | 0.239 |
| GO:0006810 | transport | *col-89; t05e7.3; col-94; r11.1; y57g11c.23; snt-2; cle-1; cuc-1; k07h8.2; col-91; sedl-1; h17b01.1; lbp-7; b0281.5; nhx-6; unc-32; t23b12.6; rab-28; rap-1; unc-49; ftn-2; tba-1; c51e3.6; lbp-1; nxt-1; unc-64; tbg-1; y40b1b.8; glc-2; evl-20; r05f9.1; rab-10; c50d2.2; emo-1; f23h12.2; sec-8; y51b9a.6; rab-37; tomm-7* | 0.244 |
| GO:0050801 | ion homeostasis | *nhx-6; ftn-2* | 0.246 |
| GO:0045138 | tail tip morphogenesis | *unc-130; vab-3* | 0.246 |
| GO:0009566 | fertilization | *tba-1; gpc-2; pfd-3* | 0.254 |
| GO:0007338 | single fertilization | *tba-1; gpc-2; pfd-3* | 0.254 |
| GO:0003002 | regionalization | *vab-3; cul-2* | 0.257 |
| GO:0006914 | autophagy | *lgg-1* | 0.26 |
| GO:0006487 | protein amino acid N-linked glycosylation | *gly-14* | 0.26 |
| GO:0045793 | positive regulation of cell size | *cul-1* | 0.26 |
| GO:0046660 | female sex differentiation | *emo-1* | 0.26 |
| GO:0008284 | positive regulation of cell proliferation | *cye-1* | 0.26 |
| GO:0001522 | pseudouridine synthesis | *k01g5.5* | 0.26 |
| GO:0035112 | genitalia morphogenesis | *cogc-1* | 0.26 |
| GO:0051329 | interphase of mitotic cell cycle | *cye-1* | 0.26 |
| GO:0051052 | regulation of DNA metabolic process | *lon-1* | 0.26 |
| GO:0045226 | extracellular polysaccharide biosynthetic process | *c01f1.3* | 0.26 |
| GO:0007254 | JNK cascade | *jnk-1* | 0.26 |
| GO:0031098 | stress-activated protein kinase signaling pathway | *jnk-1* | 0.26 |
| GO:0046379 | extracellular polysaccharide metabolic process | *c01f1.3* | 0.26 |
| GO:0009308 | amine metabolic process | *c10c5.5; zk1127.10; m02d8.4; c10c5.4; c39d10.7; pah-1; ace-3; ars-2* | 0.262 |
| GO:0050793 | regulation of developmental process | *aos-1; ubc-9; hpl-2; cye-1; lin-2; sur-6* | 0.268 |
| GO:0022008 | neurogenesis | *unc-130; cnd-1; unc-129* | 0.268 |
| GO:0048699 | generation of neurons | *unc-130; cnd-1; unc-129* | 0.268 |
| GO:0006807 | nitrogen compound metabolic process | *c10c5.5; zk1127.10; m02d8.4; c10c5.4; c39d10.7; pah-1; ace-3; ars-2* | 0.271 |
| GO:0008033 | tRNA processing | *tgt-1; k01g5.5* | 0.282 |
| GO:0051325 | interphase | *cye-1* | 0.282 |
| GO:0030728 | ovulation | *emo-1* | 0.282 |
| GO:0008037 | cell recognition | *cnd-1* | 0.282 |
| GO:0008038 | neuron recognition | *cnd-1* | 0.282 |
| GO:0042078 | germ-line stem cell division | *cul-2* | 0.282 |
| GO:0017145 | stem cell division | *cul-2* | 0.282 |
| GO:0045216 | intercellular junction assembly and maintenance | *ajm-1* | 0.282 |
| GO:0000578 | embryonic axis specification | *cul-2* | 0.282 |
| GO:0006928 | cell motility | *tnc-2; unc-130; unc-129; cogc-1* | 0.286 |
| GO:0051674 | localization of cell | *tnc-2; unc-130; unc-129; cogc-1* | 0.286 |
| GO:0040012 | regulation of locomotion | *r07e5.3; unc-49; ace-3; cogc-1; aha-1; sec-8* | 0.3 |
| GO:0035121 | tail morphogenesis | *unc-130; vab-3* | 0.301 |
| GO:0007126 | meiosis | *fog-3; eri-1; cul-2* | 0.301 |
| GO:0048477 | oogenesis | *aos-1; emo-1; csn-5* | 0.301 |
| GO:0007350 | blastoderm segmentation | *cul-2* | 0.303 |
| GO:0007346 | regulation of progression through mitotic cell cycle | *cul-1* | 0.303 |
| GO:0042133 | neurotransmitter metabolic process | *ace-3* | 0.303 |
| GO:0006888 | ER to Golgi vesicle-mediated transport | *sedl-1* | 0.303 |
| GO:0012502 | induction of programmed cell death | *asp-4* | 0.303 |
| GO:0035282 | segmentation | *cul-2* | 0.303 |
| GO:0009057 | macromolecule catabolic process | *ufd-1; rpt-6; gpd-2; nuc-1; eri-1; cul-2* | 0.303 |
| GO:0051327 | M phase of meiotic cell cycle | *fog-3; eri-1; cul-2* | 0.304 |
| GO:0006605 | protein targeting | *emo-1; f23h12.2* | 0.318 |
| GO:0006006 | glucose metabolic process | *gpd-2; h04m03.1* | 0.318 |
| GO:0030163 | protein catabolic process | *ufd-1; rpt-6; cul-2* | 0.32 |
| GO:0051321 | meiotic cell cycle | *fog-3; eri-1; cul-2* | 0.32 |
| GO:0006955 | immune response | *pmk-1* | 0.32 |
| GO:0030262 | apoptotic nuclear changes | *nuc-1* | 0.32 |
| GO:0002376 | immune system process | *pmk-1* | 0.32 |
| GO:0009408 | response to heat | *daf-16* | 0.32 |
| GO:0048675 | axon extension | *unc-130* | 0.32 |
| GO:0009112 | nucleobase metabolic process | *t19b4.3* | 0.32 |
| GO:0040020 | regulation of meiosis | *fog-3* | 0.32 |
| GO:0006309 | DNA fragmentation during apoptosis | *nuc-1* | 0.32 |
| GO:0007271 | synaptic transmission, cholinergic | *ace-3* | 0.32 |
| GO:0006821 | chloride transport | *unc-49* | 0.32 |
| GO:0043065 | positive regulation of apoptosis | *tra-1* | 0.32 |
| GO:0019538 | protein metabolic process | *hlh-1; jnk-1; rpt-6; cdk-7; cct-2; rpl-11.1; rpl-25.2; lin-2; y19d10b.6; asp-4; f42g10.1; c26b9.5; pph-5; max-2; ubc-9; fkb-3; ife-3; cyn-3; gsk-3; ubc-20; c10c5.5; tba-1; rps-22; y43c5b.2; lap-1; tbg-1; pmk-1; c37a2.7; c44h4.6; w09d10.3; y69f12a.1; cul-2; gly-14; c10c5.4; pfd-3; ars-2; ufd-1; f21f3.2; mpk-1; bre-2; fem-2; f54d10.7* | 0.33 |
| GO:0009994 | oocyte differentiation | *aos-1; emo-1* | 0.33 |
| GO:0048599 | oocyte development | *aos-1; emo-1* | 0.33 |
| GO:0008219 | cell death | *pmk-1; unc-32; tra-1; nuc-1; asp-4* | 0.331 |
| GO:0043285 | biopolymer catabolic process | *ufd-1; rpt-6; nuc-1; eri-1; cul-2* | 0.331 |
| GO:0016265 | death | *pmk-1; unc-32; tra-1; nuc-1; asp-4* | 0.331 |
| GO:0044260 | cellular macromolecule metabolic process | *jnk-1; cdk-7; cct-2; rpl-11.1; rpl-25.2; y19d10b.6; asp-4; lin-2; f42g10.1; c01f1.3; c26b9.5; pph-5; max-2; ubc-9; fkb-3; ife-3; cyn-3; gsk-3; ubc-20; c10c5.5; tba-1; rps-22; y43c5b.2; lap-1; tbg-1; pmk-1; c37a2.7; c44h4.6; w09d10.3; y69f12a.1; cul-2; gly-14; c39d10.7; c10c5.4; pfd-3; ars-2; ufd-1; f21f3.2; mpk-1; bre-2; fem-2; f54d10.7* | 0.331 |
| GO:0009628 | response to abiotic stimulus | *pmk-1; jnk-1; daf-16* | 0.331 |
| GO:0048193 | Golgi vesicle transport | *sedl-1* | 0.331 |
| GO:0009127 | purine nucleoside monophosphate biosynthetic process | *b0286.3* | 0.331 |
| GO:0000165 | MAPKKK cascade | *jnk-1* | 0.331 |
| GO:0009126 | purine nucleoside monophosphate metabolic process | *b0286.3* | 0.331 |
| GO:0006921 | cell structure disassembly during apoptosis | *nuc-1* | 0.331 |
| GO:0030005 | cellular di-, tri-valent inorganic cation homeostasis | *ftn-2* | 0.331 |
| GO:0007549 | dosage compensation | *sdc-3* | 0.331 |
| GO:0009167 | purine ribonucleoside monophosphate metabolic process | *b0286.3* | 0.331 |
| GO:0051302 | regulation of cell division | *cul-1* | 0.331 |
| GO:0009168 | purine ribonucleoside monophosphate biosynthetic process | *b0286.3* | 0.331 |
| GO:0006308 | DNA catabolic process | *nuc-1* | 0.331 |
| GO:0007292 | female gamete generation | *aos-1; emo-1; csn-5* | 0.336 |
| GO:0007018 | microtubule-based movement | *tbg-1; tba-1* | 0.338 |
| GO:0019752 | carboxylic acid metabolic process | *c10c5.5; zk1127.10; m02d8.4; c10c5.4; pah-1; h04m03.1; ars-2* | 0.338 |
| GO:0006082 | organic acid metabolic process | *c10c5.5; zk1127.10; m02d8.4; c10c5.4; pah-1; h04m03.1; ars-2* | 0.338 |
| GO:0010468 | regulation of gene expression | *hlh-1; ceh-38; zc204.2; mxl-1; mdl-1; vab-15; mdt-6; c29f9.5; unc-130; rab-10; sdc-3; vab-3; cnd-1; ceh-23; daf-7; elt-3; hlh-8; sbp-1; eri-1; pha-4; aha-1* | 0.34 |
| GO:0007399 | nervous system development | *unc-130; cnd-1; unc-129* | 0.341 |
| GO:0006350 | transcription | *hlh-1; ceh-38; w06e11.1; zc204.2; cdk-7; mxl-1; mdl-1; vab-15; mdt-6; c29f9.5; unc-130; vab-3; rab-10; cnd-1; ceh-23; daf-7; elt-3; hlh-8; sbp-1; pha-4; aha-1* | 0.343 |
| GO:0030705 | cytoskeleton-dependent intracellular transport | *tbg-1; tba-1* | 0.343 |
| GO:0009101 | glycoprotein biosynthetic process | *gly-14; bre-2* | 0.343 |
| GO:0006486 | protein amino acid glycosylation | *gly-14; bre-2* | 0.343 |
| GO:0044262 | cellular carbohydrate metabolic process | *c01f1.3; gpd-2; c39d10.7; h04m03.1; bre-1* | 0.343 |
| GO:0007028 | cytoplasm organization and biogenesis | *ajm-1* | 0.343 |
| GO:0009948 | anterior/posterior axis specification | *cul-2* | 0.343 |
| GO:0006401 | RNA catabolic process | *eri-1* | 0.343 |
| GO:0055066 | di-, tri-valent inorganic cation homeostasis | *ftn-2* | 0.343 |
| GO:0030003 | cellular cation homeostasis | *ftn-2* | 0.343 |
| GO:0019219 | regulation of nucleobase, nucleoside, nucleotide and nucleic acid metabolic process | *hlh-1; ceh-38; zc204.2; mxl-1; mdl-1; vab-15; mdt-6; c29f9.5; unc-130; rab-10; vab-3; cnd-1; ceh-23; daf-7; lon-1; elt-3; hlh-8; sbp-1; pha-4; aha-1* | 0.345 |
| GO:0007389 | pattern specification process | *vab-3; cul-2* | 0.35 |
| GO:0006399 | tRNA metabolic process | *tgt-1; k01g5.5; ars-2* | 0.354 |
| GO:0006950 | response to stress | *pmk-1; jnk-1; cul-1; unc-32; skr-15; k08f4.1* | 0.354 |
| GO:0045860 | positive regulation of protein kinase activity | *cye-1* | 0.354 |
| GO:0033692 | cellular polysaccharide biosynthetic process | *c01f1.3* | 0.354 |
| GO:0000271 | polysaccharide biosynthetic process | *c01f1.3* | 0.354 |
| GO:0051728 | cell cycle switching, mitotic to meiotic cell cycle | *cki-1* | 0.354 |
| GO:0009161 | ribonucleoside monophosphate metabolic process | *b0286.3* | 0.354 |
| GO:0051729 | germline cell cycle switching, mitotic to meiotic cell cycle | *cki-1* | 0.354 |
| GO:0006825 | copper ion transport | *cuc-1* | 0.354 |
| GO:0009156 | ribonucleoside monophosphate biosynthetic process | *b0286.3* | 0.354 |
| GO:0033674 | positive regulation of kinase activity | *cye-1* | 0.354 |
| GO:0055080 | cation homeostasis | *ftn-2* | 0.354 |
| GO:0051347 | positive regulation of transferase activity | *cye-1* | 0.354 |
| GO:0009072 | aromatic amino acid family metabolic process | *pah-1* | 0.354 |
| GO:0009100 | glycoprotein metabolic process | *gly-14; bre-2* | 0.36 |
| GO:0040029 | regulation of gene expression, epigenetic | *sdc-3; eri-1* | 0.36 |
| GO:0044267 | cellular protein metabolic process | *jnk-1; cdk-7; cct-2; rpl-11.1; rpl-25.2; y19d10b.6; asp-4; lin-2; c26b9.5; f42g10.1; pph-5; max-2; ubc-9; fkb-3; ife-3; cyn-3; gsk-3; ubc-20; c10c5.5; tba-1; rps-22; y43c5b.2; lap-1; tbg-1; pmk-1; c37a2.7; c44h4.6; w09d10.3; y69f12a.1; cul-2; gly-14; c10c5.4; pfd-3; ars-2; ufd-1; f21f3.2; mpk-1; bre-2; fem-2; f54d10.7* | 0.362 |
| GO:0006904 | vesicle docking during exocytosis | *sec-8* | 0.372 |
| GO:0006414 | translational elongation | *c37a2.7* | 0.372 |
| GO:0035262 | gonad morphogenesis | *cogc-1* | 0.372 |
| GO:0006885 | regulation of pH | *nhx-6* | 0.372 |
| GO:0043413 | biopolymer glycosylation | *gly-14; bre-2; ugt-1* | 0.379 |
| GO:0045045 | secretory pathway | *sedl-1; sec-8* | 0.382 |
| GO:0022406 | membrane docking | *sec-8* | 0.382 |
| GO:0048278 | vesicle docking | *sec-8* | 0.382 |
| GO:0009124 | nucleoside monophosphate biosynthetic process | *b0286.3* | 0.382 |
| GO:0016568 | chromatin modification | *r07e5.3* | 0.382 |
| GO:0055082 | cellular chemical homeostasis | *ftn-2* | 0.382 |
| GO:0019915 | sequestering of lipid | *sbp-1* | 0.382 |
| GO:0006694 | steroid biosynthetic process | *c01f1.3* | 0.382 |
| GO:0007243 | protein kinase cascade | *jnk-1* | 0.382 |
| GO:0009123 | nucleoside monophosphate metabolic process | *b0286.3* | 0.382 |
| GO:0006873 | cellular ion homeostasis | *ftn-2* | 0.382 |
| GO:0022411 | cellular component disassembly | *nuc-1* | 0.382 |
| GO:0031323 | regulation of cellular metabolic process | *hlh-1; ceh-38; zc204.2; mxl-1; mdl-1; vab-15; mdt-6; c29f9.5; unc-130; rab-10; vab-3; cnd-1; ceh-23; daf-7; lon-1; elt-3; hlh-8; sbp-1; pha-4; aha-1* | 0.385 |
| GO:0016070 | RNA metabolic process | *hlh-1; ceh-38; tgt-1; zc204.2; cdk-7; mxl-1; k01g5.5; snr-2; vab-15; ars-2; c29f9.5; unc-130; rab-10; vab-3; ceh-23; daf-7; snr-3; elt-3; sbp-1; eri-1; lsm-1; pha-4; aha-1* | 0.401 |
| GO:0043085 | positive regulation of catalytic activity | *cye-1* | 0.401 |
| GO:0006952 | defense response | *pmk-1; daf-16* | 0.407 |
| GO:0045449 | regulation of transcription | *hlh-1; ceh-38; zc204.2; mxl-1; mdl-1; vab-15; mdt-6; c29f9.5; unc-130; vab-3; rab-10; cnd-1; ceh-23; daf-7; hlh-8; elt-3; sbp-1; pha-4; aha-1* | 0.408 |
| GO:0006576 | biogenic amine metabolic process | *ace-3* | 0.419 |
| GO:0051603 | proteolysis involved in cellular protein catabolic process | *ufd-1; cul-2* | 0.432 |
| GO:0019941 | modification-dependent protein catabolic process | *ufd-1; cul-2* | 0.432 |
| GO:0006511 | ubiquitin-dependent protein catabolic process | *ufd-1; cul-2* | 0.432 |
| GO:0043632 | modification-dependent macromolecule catabolic process | *ufd-1; cul-2* | 0.432 |
| GO:0009968 | negative regulation of signal transduction | *ins-18* | 0.432 |
| GO:0051338 | regulation of transferase activity | *cye-1* | 0.432 |
| GO:0045859 | regulation of protein kinase activity | *cye-1* | 0.432 |
| GO:0043549 | regulation of kinase activity | *cye-1* | 0.432 |
| GO:0019222 | regulation of metabolic process | *hlh-1; ceh-38; zc204.2; mxl-1; mdl-1; vab-15; mdt-6; c29f9.5; unc-130; rab-10; vab-3; cnd-1; ceh-23; daf-7; lon-1; elt-3; hlh-8; sbp-1; pha-4; aha-1* | 0.438 |
| GO:0044257 | cellular protein catabolic process | *ufd-1; cul-2* | 0.438 |
| GO:0040027 | negative regulation of vulval development | *ubc-9; hpl-2* | 0.438 |
| GO:0009798 | axis specification | *cul-2* | 0.446 |
| GO:0044248 | cellular catabolic process | *ufd-1; gpd-2; pah-1; nuc-1; eri-1; ace-3; cul-2* | 0.455 |
| GO:0051093 | negative regulation of developmental process | *ubc-9; hpl-2* | 0.455 |
| GO:0048519 | negative regulation of biological process | *lon-1; ins-18; cul-1; ubc-9; mdl-1; cki-1; hpl-2* | 0.457 |
| GO:0033057 | reproductive behavior in a multicellular organism | *c01f1.3; cnd-1; cki-1; r07e5.3; skp-1; hlh-8; f48c1.4; lin-2; sur-6* | 0.457 |
| GO:0018991 | oviposition | *c01f1.3; cnd-1; cki-1; r07e5.3; skp-1; hlh-8; f48c1.4; lin-2; sur-6* | 0.457 |
| GO:0008202 | steroid metabolic process | *c01f1.3* | 0.458 |
| GO:0016246 | RNA interference | *eri-1* | 0.458 |
| GO:0035023 | regulation of Rho protein signal transduction | *tag-218* | 0.458 |
| GO:0032940 | secretion by cell | *sedl-1; sec-8* | 0.459 |
| GO:0006512 | ubiquitin cycle | *ubc-9; ubc-20* | 0.468 |
| GO:0006413 | translational initiation | *ife-3* | 0.471 |
| GO:0032259 | methylation | *c18a3.1* | 0.471 |
| GO:0040019 | positive regulation of embryonic development | *aos-1* | 0.471 |
| GO:0019098 | reproductive behavior | *c01f1.3; cnd-1; cki-1; r07e5.3; skp-1; hlh-8; f48c1.4; lin-2; sur-6* | 0.481 |
| GO:0006260 | DNA replication | *lon-1; cye-1* | 0.481 |
| GO:0007266 | Rho protein signal transduction | *tag-218* | 0.481 |
| GO:0045995 | regulation of embryonic development | *aos-1* | 0.481 |
| GO:0006575 | amino acid derivative metabolic process | *ace-3* | 0.481 |
| GO:0022610 | biological adhesion | *vab-3; cle-1* | 0.484 |
| GO:0007155 | cell adhesion | *vab-3; cle-1* | 0.484 |
| GO:0006915 | apoptosis | *tra-1; nuc-1* | 0.484 |
| GO:0008632 | apoptotic program | *nuc-1* | 0.49 |
| GO:0008643 | carbohydrate transport | *h17b01.1* | 0.49 |
| GO:0009880 | embryonic pattern specification | *cul-2* | 0.49 |
| GO:0030198 | extracellular matrix organization and biogenesis | *fbl-1* | 0.49 |
| GO:0006887 | exocytosis | *sec-8* | 0.49 |
| GO:0044264 | cellular polysaccharide metabolic process | *c01f1.3; c39d10.7* | 0.497 |
| GO:0005976 | polysaccharide metabolic process | *c01f1.3; c39d10.7* | 0.497 |
| GO:0015674 | di-, tri-valent inorganic cation transport | *ftn-2* | 0.501 |
| GO:0008652 | amino acid biosynthetic process | *m02d8.4* | 0.501 |
| GO:0009063 | amino acid catabolic process | *pah-1* | 0.501 |
| GO:0007179 | transforming growth factor beta receptor signaling pathway | *lon-1* | 0.501 |
| GO:0006096 | glycolysis | *gpd-2* | 0.513 |
| GO:0008105 | asymmetric protein localization | *cul-2* | 0.513 |
| GO:0008356 | asymmetric cell division | *cul-2* | 0.513 |
| GO:0022618 | protein-RNA complex assembly | *ife-3* | 0.523 |
| GO:0035194 | RNA-mediated posttranscriptional gene silencing | *eri-1* | 0.523 |
| GO:0031047 | RNA-mediated gene silencing | *eri-1* | 0.523 |
| GO:0009266 | response to temperature stimulus | *daf-16* | 0.523 |
| GO:0007178 | transmembrane receptor protein serine/threonine kinase signaling pathway | *lon-1* | 0.523 |
| GO:0016441 | posttranscriptional gene silencing | *eri-1* | 0.523 |
| GO:0016477 | cell migration | *unc-130; unc-129; cogc-1* | 0.548 |
| GO:0007308 | oocyte construction | *aos-1* | 0.548 |
| GO:0042981 | regulation of apoptosis | *tra-1* | 0.548 |
| GO:0007242 | intracellular signaling cascade | *jnk-1; rab-10; rab-28; rap-1; mpk-1; tag-218; rab-37; evl-20; nuc-1; aha-1; y43c5b.2* | 0.548 |
| GO:0030001 | metal ion transport | *b0281.5; cuc-1; nhx-6; t23b12.6; r05f9.1; ftn-2* | 0.578 |
| GO:0016458 | gene silencing | *eri-1* | 0.585 |
| GO:0001505 | regulation of neurotransmitter levels | *ace-3* | 0.585 |
| GO:0040017 | positive regulation of locomotion | *r07e5.3; aha-1; sec-8* | 0.585 |
| GO:0046365 | monosaccharide catabolic process | *gpd-2* | 0.593 |
| GO:0019320 | hexose catabolic process | *gpd-2* | 0.593 |
| GO:0006007 | glucose catabolic process | *gpd-2* | 0.593 |
| GO:0006066 | alcohol metabolic process | *gpd-2; h04m03.1; bre-1* | 0.593 |
| GO:0040032 | post-embryonic body morphogenesis | *hlh-1; his-13; col-94; cki-1; cye-1; t02g5.7; c01a2.5; sbp-1; evl-20; f48c1.4* | 0.595 |
| GO:0009056 | catabolic process | *ufd-1; rpt-6; gpd-2; pah-1; nuc-1; eri-1; ace-3; cul-2* | 0.605 |
| GO:0009886 | post-embryonic morphogenesis | *hlh-1; his-13; col-94; cki-1; cye-1; t02g5.7; c01a2.5; sbp-1; evl-20; f48c1.4* | 0.606 |
| GO:0009309 | amine biosynthetic process | *m02d8.4* | 0.608 |
| GO:0006997 | nuclear organization and biogenesis | *nuc-1* | 0.608 |
| GO:0045595 | regulation of cell differentiation | *hpl-2* | 0.608 |
| GO:0006730 | one-carbon compound metabolic process | *c18a3.1* | 0.608 |
| GO:0030334 | regulation of cell migration | *cogc-1* | 0.608 |
| GO:0044271 | nitrogen compound biosynthetic process | *m02d8.4* | 0.608 |
| GO:0046164 | alcohol catabolic process | *gpd-2* | 0.614 |
| GO:0051270 | regulation of cell motility | *cogc-1* | 0.614 |
| GO:0009058 | biosynthetic process | *b0286.3; c37a2.7; w09d10.3; bre-1; tgt-1; gly-14; rpl-11.1; m02d8.4; rpl-25.2; h04m03.1; ars-2; c01f1.3; t04g9.4; ife-3; pnk-1; bre-2; rps-22* | 0.622 |
| GO:0044265 | cellular macromolecule catabolic process | *ufd-1; gpd-2; eri-1; cul-2* | 0.628 |
| GO:0045934 | negative regulation of nucleobase, nucleoside, nucleotide and nucleic acid metabolic process | *lon-1* | 0.633 |
| GO:0043062 | extracellular structure organization and biogenesis | *fbl-1* | 0.633 |
| GO:0006412 | translation | *c37a2.7; rpl-11.1; ife-3; rpl-25.2; w09d10.3; ars-2; rps-22* | 0.639 |
| GO:0043412 | biopolymer modification | *pmk-1; jnk-1; c44h4.6; y69f12a.1; ugt-1; tgt-1; gly-14; cdk-7; k01g5.5; lin-2; max-2; pph-5; f21f3.2; ubc-9; gsk-3; ubc-20; mpk-1; bre-2; fem-2; f54d10.7; y43c5b.2* | 0.645 |
| GO:0044249 | cellular biosynthetic process | *b0286.3; c37a2.7; ife-3; w09d10.3; pnk-1; bre-1; tgt-1; rpl-11.1; m02d8.4; rpl-25.2; h04m03.1; rps-22; ars-2* | 0.674 |
| GO:0006351 | transcription, DNA-dependent | *hlh-1; ceh-38; zc204.2; mxl-1; cdk-7; vab-15; unc-130; c29f9.5; rab-10; vab-3; ceh-23; daf-7; elt-3; sbp-1; aha-1; pha-4* | 0.677 |
| GO:0007517 | muscle development | *hlh-1* | 0.678 |
| GO:0030259 | lipid glycosylation | *ugt-1* | 0.678 |
| GO:0005975 | carbohydrate metabolic process | *c01f1.3; gpd-2; ugt-1; bre-1; hex-1; c39d10.7; lys-8; h04m03.1* | 0.678 |
| GO:0010171 | body morphogenesis | *hlh-1; his-13; col-94; cki-1; cye-1; t02g5.7; c01a2.5; sbp-1; evl-20; f48c1.4* | 0.68 |
| GO:0046483 | heterocycle metabolic process | *t19b4.3* | 0.684 |
| GO:0032774 | RNA biosynthetic process | *hlh-1; ceh-38; zc204.2; mxl-1; cdk-7; vab-15; unc-130; c29f9.5; rab-10; vab-3; ceh-23; daf-7; elt-3; sbp-1; aha-1; pha-4* | 0.684 |
| GO:0030036 | actin cytoskeleton organization and biogenesis | *pfn-1* | 0.692 |
| GO:0006355 | regulation of transcription, DNA-dependent | *hlh-1; ceh-38; zc204.2; mxl-1; vab-15; unc-130; c29f9.5; rab-10; vab-3; ceh-23; daf-7; elt-3; sbp-1; aha-1; pha-4* | 0.728 |
| GO:0007265 | Ras protein signal transduction | *tag-218; mpk-1* | 0.736 |
| GO:0046903 | secretion | *sedl-1; sec-8* | 0.746 |
| GO:0019725 | cellular homeostasis | *gsto-3; ftn-2* | 0.747 |
| GO:0040015 | negative regulation of multicellular organism growth | *lon-1; cul-1* | 0.758 |
| GO:0045926 | negative regulation of growth | *lon-1; cul-1* | 0.76 |
| GO:0006725 | aromatic compound metabolic process | *t19b4.3; pah-1* | 0.796 |
| GO:0006812 | cation transport | *cuc-1; b0281.5; k07h8.2; nhx-6; t23b12.6; unc-32; r05f9.1; ftn-2* | 0.798 |
| GO:0006281 | DNA repair | *cul-1; skr-15; k08f4.1* | 0.817 |
| GO:0035264 | multicellular organism growth | *k07c5.4; col-94; sdc-3; cul-1; rab-10; snt-2; cyh-1; sec-8; lon-1; y65b4br.5; f48c1.4; aha-1* | 0.817 |
| GO:0040014 | regulation of multicellular organism growth | *k07c5.4; col-94; sdc-3; cul-1; rab-10; snt-2; cyh-1; sec-8; lon-1; y65b4br.5; f48c1.4; aha-1* | 0.817 |
| GO:0006813 | potassium ion transport | *b0281.5; t23b12.6; r05f9.1* | 0.82 |
| GO:0006974 | response to DNA damage stimulus | *cul-1; skr-15; k08f4.1* | 0.833 |
| GO:0009719 | response to endogenous stimulus | *cul-1; skr-15; k08f4.1* | 0.833 |
| GO:0006468 | protein amino acid phosphorylation | *pmk-1; jnk-1; max-2; f21f3.2; c44h4.6; gsk-3; y69f12a.1; mpk-1; cdk-7; lin-2; y43c5b.2* | 0.862 |
| GO:0015698 | inorganic anion transport | *col-89; col-94; col-91; cle-1; unc-49* | 0.88 |
| GO:0006820 | anion transport | *col-89; col-94; col-91; cle-1; unc-49* | 0.883 |
| GO:0015672 | monovalent inorganic cation transport | *b0281.5; nhx-6; t23b12.6; unc-32; r05f9.1* | 0.886 |
| GO:0006464 | protein modification process | *pmk-1; jnk-1; c44h4.6; y69f12a.1; gly-14; cdk-7; lin-2; max-2; pph-5; f21f3.2; ubc-9; gsk-3; ubc-20; mpk-1; bre-2; y43c5b.2; fem-2; f54d10.7* | 0.903 |
| GO:0040018 | positive regulation of multicellular organism growth | *k07c5.4; col-94; sdc-3; rab-10; snt-2; cyh-1; sec-8; y65b4br.5; f48c1.4; aha-1* | 0.906 |
| GO:0006811 | ion transport | *col-89; b0281.5; col-94; nhx-6; t23b12.6; unc-32; cle-1; unc-49; ftn-2; cuc-1; k07h8.2; glc-2; col-91; r05f9.1* | 0.97 |
| GO:0043687 | post-translational protein modification | *pmk-1; jnk-1; max-2; pph-5; f21f3.2; ubc-9; c44h4.6; gsk-3; ubc-20; y69f12a.1; mpk-1; cdk-7; lin-2; fem-2; y43c5b.2* | 1 |
| **Molecular Function** |  |  |  |
| GO:0004046 | aminoacylase activity | *c10c5.5; c10c5.4* | 0.107 |
| GO:0004707 | MAP kinase activity | *pmk-1; jnk-1; mpk-1* | 0.107 |
| GO:0005057 | receptor signaling protein activity | *pmk-1; jnk-1; aha-1; mpk-1* | 0.107 |
| GO:0003924 | GTPase activity | *tbg-1; rab-37; tba-1; rab-10; zk1236.1; rab-28; rap-1* | 0.107 |
| GO:0046982 | protein heterodimerization activity | *mdl-1; mxl-1* | 0.164 |
| GO:0008289 | lipid binding | *t05e7.3; vt23b5.2; f25h2.2; rabs-5; lbp-7; lbp-1* | 0.164 |
| GO:0015450 | P-P-bond-hydrolysis-driven protein transmembrane transporter activity | *emo-1; tomm-7* | 0.164 |
| GO:0008320 | protein transmembrane transporter activity | *emo-1; tomm-7* | 0.164 |
| GO:0022884 | macromolecule transmembrane transporter activity | *emo-1; tomm-7* | 0.164 |
| GO:0046983 | protein dimerization activity | *c10c5.5; mdl-1; mxl-1; c10c5.4; ace-3* | 0.164 |
| GO:0005525 | GTP binding | *tbg-1; rab-10; zk1236.1; rab-28; rap-1; rab-37; tba-1; h04m03.1; evl-20* | 0.169 |
| GO:0032561 | guanyl ribonucleotide binding | *tbg-1; rab-10; zk1236.1; rab-28; rap-1; rab-37; tba-1; h04m03.1; evl-20* | 0.169 |
| GO:0004702 | receptor signaling protein serine/threonine kinase activity | *pmk-1; jnk-1; mpk-1* | 0.169 |
| GO:0008171 | O-methyltransferase activity | *y40b10a.2; y40b10a.6* | 0.215 |
| GO:0019001 | guanyl nucleotide binding | *tbg-1; rab-10; zk1236.1; rab-28; rap-1; rab-37; tba-1; h04m03.1; evl-20* | 0.215 |
| GO:0035091 | phosphoinositide binding | *vt23b5.2; f25h2.2; rabs-5* | 0.215 |
| GO:0043425 | bHLH transcription factor binding | *hlh-1* | 0.215 |
| GO:0004563 | beta-N-acetylhexosaminidase activity | *hex-1* | 0.215 |
| GO:0003999 | adenine phosphoribosyltransferase activity | *t19b4.3* | 0.215 |
| GO:0004639 | phosphoribosylaminoimidazolesuccinocarboxamide synthase activity | *b0286.3* | 0.215 |
| GO:0004505 | phenylalanine 4-monooxygenase activity | *pah-1* | 0.215 |
| GO:0005160 | transforming growth factor beta receptor binding | *unc-129* | 0.215 |
| GO:0005061 | aryl hydrocarbon receptor nuclear translocator activity | *aha-1* | 0.215 |
| GO:0008083 | growth factor activity | *unc-129; daf-7* | 0.215 |
| GO:0032266 | phosphatidylinositol 3-phosphate binding | *vt23b5.2; rabs-5* | 0.246 |
| GO:0005543 | phospholipid binding | *vt23b5.2; f25h2.2; rabs-5* | 0.269 |
| GO:0016763 | transferase activity, transferring pentosyl groups | *tgt-1; t19b4.3* | 0.269 |
| GO:0004638 | phosphoribosylaminoimidazole carboxylase activity | *b0286.3* | 0.269 |
| GO:0004965 | GABA-B receptor activity | *y41g9a.4* | 0.269 |
| GO:0015929 | hexosaminidase activity | *hex-1* | 0.269 |
| GO:0004693 | cyclin-dependent protein kinase activity | *cdk-7* | 0.269 |
| GO:0008199 | ferric iron binding | *ftn-2* | 0.269 |
| GO:0004594 | pantothenate kinase activity | *pnk-1* | 0.269 |
| GO:0016531 | copper chaperone activity | *cuc-1* | 0.269 |
| GO:0004705 | JUN kinase activity | *jnk-1* | 0.269 |
| GO:0008446 | GDP-mannose 4,6-dehydratase activity | *bre-1* | 0.269 |
| GO:0004861 | cyclin-dependent protein kinase inhibitor activity | *cki-1* | 0.269 |
| GO:0008479 | queuine tRNA-ribosyltransferase activity | *tgt-1* | 0.269 |
| GO:0004813 | alanine-tRNA ligase activity | *ars-2* | 0.269 |
| GO:0008897 | phosphopantetheinyltransferase activity | *t04g9.4* | 0.269 |
| GO:0008134 | transcription factor binding | *hlh-1; c29f9.5; rab-10* | 0.292 |
| GO:0019887 | protein kinase regulator activity | *cki-1; cye-1* | 0.305 |
| GO:0016917 | GABA receptor activity | *y41g9a.4; glc-2; unc-49* | 0.306 |
| GO:0019207 | kinase regulator activity | *cki-1; cye-1* | 0.306 |
| GO:0016831 | carboxy-lyase activity | *b0286.3; h04m03.1* | 0.306 |
| GO:0003827 | alpha-1,3-mannosylglycoprotein 2-beta-N-acetylglucosaminyltransferase activity | *gly-14* | 0.306 |
| GO:0016889 | endodeoxyribonuclease activity, producing 3'-phosphomonoesters | *nuc-1* | 0.306 |
| GO:0016439 | tRNA-pseudouridine synthase activity | *k01g5.5* | 0.306 |
| GO:0019210 | kinase inhibitor activity | *cki-1* | 0.306 |
| GO:0004531 | deoxyribonuclease II activity | *nuc-1* | 0.306 |
| GO:0003990 | acetylcholinesterase activity | *ace-3* | 0.306 |
| GO:0004860 | protein kinase inhibitor activity | *cki-1* | 0.306 |
| GO:0004611 | phosphoenolpyruvate carboxykinase activity | *h04m03.1* | 0.306 |
| GO:0016714 | oxidoreductase activity, acting on paired donors, with incorporation or reduction of molecular oxygen, reduced pteridine as one donor, and incorporation of one atom of oxygen | *pah-1* | 0.306 |
| GO:0016903 | oxidoreductase activity, acting on the aldehyde or oxo group of donors | *y39e4a.3; gpd-2* | 0.317 |
| GO:0004785 | copper, zinc superoxide dismutase activity | *sod-5* | 0.339 |
| GO:0004104 | cholinesterase activity | *ace-3* | 0.339 |
| GO:0016909 | SAP kinase activity | *jnk-1* | 0.339 |
| GO:0016538 | cyclin-dependent protein kinase regulator activity | *cye-1* | 0.339 |
| GO:0004066 | asparagine synthase (glutamine-hydrolyzing) activity | *m02d8.4* | 0.339 |
| GO:0009982 | pseudouridine synthase activity | *k01g5.5* | 0.339 |
| GO:0003884 | D-amino-acid oxidase activity | *f20h11.5* | 0.339 |
| GO:0004520 | endodeoxyribonuclease activity | *nuc-1* | 0.339 |
| GO:0008601 | protein phosphatase type 2A regulator activity | *sur-6* | 0.339 |
| GO:0016894 | endonuclease activity, active with either ribo- or deoxyribonucleic acids and producing 3'-phosphomonoesters | *nuc-1* | 0.375 |
| GO:0016597 | amino acid binding | *pah-1* | 0.375 |
| GO:0051766 | inositol trisphosphate kinase activity | *y22d7ar.6* | 0.375 |
| GO:0008440 | inositol trisphosphate 3-kinase activity | *y22d7ar.6* | 0.375 |
| GO:0016624 | oxidoreductase activity, acting on the aldehyde or oxo group of donors, disulfide as acceptor | *y39e4a.3* | 0.375 |
| GO:0016641 | oxidoreductase activity, acting on the CH-NH2 group of donors, oxygen as acceptor | *f20h11.5* | 0.375 |
| GO:0005201 | extracellular matrix structural constituent | *fbl-1* | 0.375 |
| GO:0004536 | deoxyribonuclease activity | *nuc-1* | 0.375 |
| GO:0008565 | protein transporter activity | *unc-64; emo-1; tomm-7* | 0.391 |
| GO:0043565 | sequence-specific DNA binding | *unc-130; his-7; sdc-3; vab-3; tra-1; ceh-23; ceh-38; zc204.2; dro-1; mdl-1; mxl-1; elt-3; mbf-1; vab-15; pha-4* | 0.4 |
| GO:0016879 | ligase activity, forming carbon-nitrogen bonds | *b0286.3; ubc-9; m02d8.4; ubc-20* | 0.4 |
| GO:0016830 | carbon-carbon lyase activity | *b0286.3; h04m03.1* | 0.4 |
| GO:0019208 | phosphatase regulator activity | *sur-6* | 0.4 |
| GO:0004523 | ribonuclease H activity | *rnh-1.1* | 0.4 |
| GO:0019888 | protein phosphatase regulator activity | *sur-6* | 0.4 |
| GO:0016721 | oxidoreductase activity, acting on superoxide radicals as acceptor | *sod-5* | 0.4 |
| GO:0004784 | superoxide dismutase activity | *sod-5* | 0.4 |
| GO:0032553 | ribonucleotide binding | *tbg-1; pmk-1; jnk-1; b0286.3; rpt-6; c44h4.6; lex-1; y69f12a.1; cdk-7; cct-2; k08f4.1; evl-20; h04m03.1; lin-2; ars-2; f55f8.2; max-2; f21f3.2; rab-10; f40f8.1; zk1236.1; rab-28; hum-5; gsk-3; rap-1; ssl-1; mpk-1; pnk-1; rab-37; tba-1; y43c5b.2* | 0.408 |
| GO:0032555 | purine ribonucleotide binding | *tbg-1; pmk-1; jnk-1; b0286.3; rpt-6; c44h4.6; lex-1; y69f12a.1; cdk-7; cct-2; k08f4.1; evl-20; h04m03.1; lin-2; ars-2; f55f8.2; max-2; f21f3.2; rab-10; f40f8.1; zk1236.1; rab-28; hum-5; gsk-3; rap-1; ssl-1; mpk-1; pnk-1; rab-37; tba-1; y43c5b.2* | 0.408 |
| GO:0004540 | ribonuclease activity | *eri-1; rnh-1.1* | 0.408 |
| GO:0016811 | hydrolase activity, acting on carbon-nitrogen (but not peptide) bonds, in linear amides | *c10c5.5; c10c5.4* | 0.408 |
| GO:0003705 | RNA polymerase II transcription factor activity, enhancer binding | *hlh-1* | 0.408 |
| GO:0008067 | metabotropic glutamate, GABA-B-like receptor activity | *y41g9a.4* | 0.408 |
| GO:0008831 | dTDP-4-dehydrorhamnose reductase activity | *c01f1.3* | 0.408 |
| GO:0016638 | oxidoreductase activity, acting on the CH-NH2 group of donors | *f20h11.5* | 0.408 |
| GO:0016884 | carbon-nitrogen ligase activity, with glutamine as amido-N-donor | *m02d8.4* | 0.408 |
| GO:0004365 | glyceraldehyde-3-phosphate dehydrogenase (phosphorylating) activity | *gpd-2* | 0.408 |
| GO:0008943 | glyceraldehyde-3-phosphate dehydrogenase activity | *gpd-2* | 0.408 |
| GO:0004519 | endonuclease activity | *nuc-1; rnh-1.1* | 0.408 |
| GO:0000166 | nucleotide binding | *jnk-1; b0286.3; rpt-6; r09b3.2; cdk-7; cct-2; k08f4.1; rpl-25.2; lin-2; max-2; zk1236.1; f40f8.1; rab-28; gsk-3; hum-5; f07f6.1; ssl-1; rap-1; pnk-1; tba-1; y43c5b.2; tbg-1; rnp-3; pmk-1; c44h4.6; lex-1; y69f12a.1; evl-20; h04m03.1; ars-2; f55f8.2; f21f3.2; rab-10; mpk-1; rab-37; t08g2.3* | 0.408 |
| GO:0005249 | voltage-gated potassium channel activity | *b0281.5; t23b12.6; r05f9.1* | 0.416 |
| GO:0016796 | exonuclease activity, active with either ribo- or deoxyribonucleic acids and producing 5'-phosphomonoesters | *eri-1* | 0.426 |
| GO:0004532 | exoribonuclease activity | *eri-1* | 0.426 |
| GO:0008022 | protein C-terminus binding | *csn-5* | 0.426 |
| GO:0000175 | 3'-5'-exoribonuclease activity | *eri-1* | 0.426 |
| GO:0005200 | structural constituent of cytoskeleton | *tbg-1* | 0.426 |
| GO:0016896 | exoribonuclease activity, producing 5'-phosphomonoesters | *eri-1* | 0.426 |
| GO:0022843 | voltage-gated cation channel activity | *b0281.5; t23b12.6; r05f9.1* | 0.429 |
| GO:0016881 | acid-amino acid ligase activity | *b0286.3; ubc-9; ubc-20* | 0.429 |
| GO:0017076 | purine nucleotide binding | *tbg-1; pmk-1; jnk-1; b0286.3; rpt-6; lex-1; c44h4.6; y69f12a.1; cdk-7; cct-2; k08f4.1; h04m03.1; evl-20; ars-2; lin-2; f55f8.2; max-2; rab-10; f21f3.2; zk1236.1; f40f8.1; rab-28; gsk-3; hum-5; ssl-1; rap-1; mpk-1; pnk-1; rab-37; tba-1; t08g2.3; y43c5b.2* | 0.429 |
| GO:0015631 | tubulin binding | *ebp-2* | 0.429 |
| GO:0008017 | microtubule binding | *ebp-2* | 0.429 |
| GO:0017151 | DEAD/H-box RNA helicase binding | *csn-5* | 0.429 |
| GO:0004364 | glutathione transferase activity | *gsto-3* | 0.429 |
| GO:0019201 | nucleotide kinase activity | *f40f8.1* | 0.429 |
| GO:0004722 | protein serine/threonine phosphatase activity | *fem-2* | 0.429 |
| GO:0008092 | cytoskeletal protein binding | *ajm-1; ebp-2; pfn-1* | 0.429 |
| GO:0004245 | neprilysin activity | *f42g10.1; y19d10b.6* | 0.448 |
| GO:0016866 | intramolecular transferase activity | *k01g5.5* | 0.452 |
| GO:0005451 | monovalent cation:proton antiporter activity | *nhx-6* | 0.452 |
| GO:0015385 | sodium:hydrogen antiporter activity | *nhx-6* | 0.452 |
| GO:0004022 | alcohol dehydrogenase activity | *stdh-1* | 0.452 |
| GO:0022832 | voltage-gated channel activity | *b0281.5; t23b12.6; r05f9.1* | 0.452 |
| GO:0005244 | voltage-gated ion channel activity | *b0281.5; t23b12.6; r05f9.1* | 0.452 |
| GO:0004890 | GABA-A receptor activity | *glc-2; unc-49* | 0.456 |
| GO:0016757 | transferase activity, transferring glycosyl groups | *gly-14; tgt-1; ugt-25; t19b4.3; bre-2; ugt-1; k02e11.5* | 0.463 |
| GO:0051082 | unfolded protein binding | *cct-2; pfd-3* | 0.466 |
| GO:0004713 | protein-tyrosine kinase activity | *pmk-1; jnk-1; max-2; f21f3.2; c44h4.6; gsk-3; y69f12a.1; mpk-1; cdk-7; lin-2; y43c5b.2* | 0.484 |
| GO:0003854 | 3-beta-hydroxy-delta5-steroid dehydrogenase activity | *c01f1.3* | 0.488 |
| GO:0016229 | steroid dehydrogenase activity | *c01f1.3* | 0.488 |
| GO:0016780 | phosphotransferase activity, for other substituted phosphate groups | *t04g9.4* | 0.488 |
| GO:0003735 | structural constituent of ribosome | *c37a2.7; rpl-11.1; rpl-25.2; w09d10.3; rps-22* | 0.495 |
| GO:0005102 | receptor binding | *ins-18; unc-129; daf-7* | 0.495 |
| GO:0004518 | nuclease activity | *nuc-1; eri-1; rnh-1.1* | 0.495 |
| GO:0008061 | chitin binding | *c39d10.7* | 0.495 |
| GO:0016891 | endoribonuclease activity, producing 5'-phosphomonoesters | *rnh-1.1* | 0.495 |
| GO:0015491 | cation:cation antiporter activity | *nhx-6* | 0.495 |
| GO:0016776 | phosphotransferase activity, phosphate group as acceptor | *f40f8.1* | 0.495 |
| GO:0016893 | endonuclease activity, active with either ribo- or deoxyribonucleic acids and producing 5'-phosphomonoesters | *rnh-1.1* | 0.495 |
| GO:0015299 | solute:hydrogen antiporter activity | *nhx-6* | 0.524 |
| GO:0050662 | coenzyme binding | *c01f1.3; qdpr-1; gpd-2; t08g2.3; bre-1* | 0.528 |
| GO:0001871 | pattern binding | *c39d10.7* | 0.54 |
| GO:0016620 | oxidoreductase activity, acting on the aldehyde or oxo group of donors, NAD or NADP as acceptor | *gpd-2* | 0.54 |
| GO:0030247 | polysaccharide binding | *c39d10.7* | 0.54 |
| GO:0004428 | inositol or phosphatidylinositol kinase activity | *y22d7ar.6* | 0.559 |
| GO:0015035 | protein disulfide oxidoreductase activity | *gsto-3* | 0.559 |
| GO:0004521 | endoribonuclease activity | *rnh-1.1* | 0.559 |
| GO:0003677 | DNA binding | *hlh-1; his-13; ceh-38; his-48; zc204.2; dro-1; mdl-1; mxl-1; vab-15; cpar-1; unc-130; his-7; sdc-3; vab-3; cnd-1; tra-1; ceh-23; ssl-1; elt-3; mbf-1; hil-3; pha-4; aha-1* | 0.566 |
| GO:0017111 | nucleoside-triphosphatase activity | *f55f8.2; tbg-1; rab-10; rpt-6; lex-1; zk1236.1; rab-28; rap-1; rab-37; tba-1; k08f4.1* | 0.568 |
| GO:0016616 | oxidoreductase activity, acting on the CH-OH group of donors, NAD or NADP as acceptor | *c01f1.3; stdh-1* | 0.568 |
| GO:0008066 | glutamate receptor activity | *y41g9a.4* | 0.568 |
| GO:0019904 | protein domain specific binding | *mxl-1* | 0.568 |
| GO:0016491 | oxidoreductase activity | *stdh-2; c01f1.3; gsto-3; sod-5; qdpr-1; y51a2b.1; gpd-2; bre-1; cyp-33c3; dhs-18; cyp-13a11; y39e4a.3; f20h11.5; t08g2.3; pah-1; stdh-1; prdx-3* | 0.568 |
| GO:0003676 | nucleic acid binding | *hlh-1; his-13; r09b3.2; dro-1; rpl-25.2; sptf-2; vab-15; cpar-1; his-7; cnd-1; tra-1; ife-3; ssl-1; mnm-2; f57c9.4; elt-3; mbf-1; hil-3; eri-1; pha-4; rnp-3; zk337.2; ceh-38; his-48; zc204.2; c18a3.1; k01g5.5; mdl-1; mxl-1; ars-2; f55f8.2; unc-130; sdc-3; vab-3; rabs-5; ceh-23; rnh-1.1; aha-1* | 0.572 |
| GO:0015300 | solute:solute antiporter activity | *nhx-6* | 0.572 |
| GO:0015036 | disulfide oxidoreductase activity | *gsto-3* | 0.572 |
| GO:0015298 | solute:cation antiporter activity | *nhx-6* | 0.572 |
| GO:0004194 | pepsin A activity | *asp-4* | 0.572 |
| GO:0016614 | oxidoreductase activity, acting on CH-OH group of donors | *c01f1.3; stdh-1* | 0.58 |
| GO:0019205 | nucleobase, nucleoside, nucleotide kinase activity | *f40f8.1* | 0.587 |
| GO:0019787 | small conjugating protein ligase activity | *ubc-9; ubc-20* | 0.587 |
| GO:0008408 | 3'-5' exonuclease activity | *eri-1* | 0.591 |
| GO:0051119 | sugar transmembrane transporter activity | *h17b01.1* | 0.591 |
| GO:0005089 | Rho guanyl-nucleotide exchange factor activity | *tag-218* | 0.591 |
| GO:0005351 | sugar:hydrogen ion symporter activity | *h17b01.1* | 0.591 |
| GO:0043176 | amine binding | *pah-1* | 0.591 |
| GO:0051536 | iron-sulfur cluster binding | *f25b5.5* | 0.594 |
| GO:0003743 | translation initiation factor activity | *ife-3* | 0.594 |
| GO:0016836 | hydro-lyase activity | *bre-1* | 0.594 |
| GO:0051540 | metal cluster binding | *f25b5.5* | 0.594 |
| GO:0005088 | Ras guanyl-nucleotide exchange factor activity | *tag-218* | 0.594 |
| GO:0016859 | cis-trans isomerase activity | *cyn-3* | 0.601 |
| GO:0003755 | peptidyl-prolyl cis-trans isomerase activity | *cyn-3* | 0.601 |
| GO:0015297 | antiporter activity | *nhx-6* | 0.601 |
| GO:0016462 | pyrophosphatase activity | *f55f8.2; tbg-1; rab-10; rpt-6; lex-1; zk1236.1; rab-28; rap-1; rab-37; tba-1; k08f4.1* | 0.601 |
| GO:0016818 | hydrolase activity, acting on acid anhydrides, in phosphorus-containing anhydrides | *f55f8.2; tbg-1; rab-10; rpt-6; lex-1; zk1236.1; rab-28; rap-1; rab-37; tba-1; k08f4.1* | 0.601 |
| GO:0008378 | galactosyltransferase activity | *bre-2* | 0.601 |
| GO:0003712 | transcription cofactor activity | *c29f9.5* | 0.601 |
| GO:0015144 | carbohydrate transmembrane transporter activity | *h17b01.1* | 0.601 |
| GO:0051287 | NAD binding | *gpd-2* | 0.601 |
| GO:0004177 | aminopeptidase activity | *lap-1* | 0.601 |
| GO:0016874 | ligase activity | *b0286.3; ubc-9; m02d8.4; ubc-20; ars-2* | 0.604 |
| GO:0016817 | hydrolase activity, acting on acid anhydrides | *f55f8.2; tbg-1; rab-10; rpt-6; lex-1; zk1236.1; rab-28; rap-1; rab-37; tba-1; k08f4.1* | 0.606 |
| GO:0003682 | chromatin binding | *hpl-2* | 0.607 |
| GO:0016765 | transferase activity, transferring alkyl or aryl (other than methyl) groups | *gsto-3* | 0.607 |
| GO:0016853 | isomerase activity | *k01g5.5; cyn-3* | 0.607 |
| GO:0016835 | carbon-oxygen lyase activity | *bre-1* | 0.633 |
| GO:0003995 | acyl-CoA dehydrogenase activity | *t08g2.3* | 0.633 |
| GO:0003899 | DNA-directed RNA polymerase activity | *w06e11.1* | 0.633 |
| GO:0008375 | acetylglucosaminyltransferase activity | *gly-14* | 0.647 |
| GO:0004857 | enzyme inhibitor activity | *tag-290; cki-1; kal-1* | 0.651 |
| GO:0008168 | methyltransferase activity | *y40b10a.2; c18a3.1; y40b10a.6* | 0.651 |
| GO:0016741 | transferase activity, transferring one-carbon groups | *y40b10a.2; c18a3.1; y40b10a.6* | 0.651 |
| GO:0016772 | transferase activity, transferring phosphorus-containing groups | *pmk-1; jnk-1; c44h4.6; y69f12a.1; w06e11.1; cdk-7; y22d7ar.6; lin-2; max-2; f21f3.2; t04g9.4; f40f8.1; gsk-3; pnk-1; mpk-1; k08e3.5; y43c5b.2* | 0.651 |
| GO:0000287 | magnesium ion binding | *t04g9.4* | 0.665 |
| GO:0003777 | microtubule motor activity | *dlc-2* | 0.669 |
| GO:0003993 | acid phosphatase activity | *b0361.7* | 0.669 |
| GO:0004190 | aspartic-type endopeptidase activity | *asp-4* | 0.669 |
| GO:0048037 | cofactor binding | *c01f1.3; qdpr-1; zk1127.10; gpd-2; t08g2.3; bre-1* | 0.669 |
| GO:0005509 | calcium ion binding | *c54e4.2; tnc-2; f25h2.2; fbl-1; t03f1.11; c56c10.9* | 0.672 |
| GO:0016829 | lyase activity | *b0286.3; h04m03.1; bre-1* | 0.697 |
| GO:0004497 | monooxygenase activity | *cyp-13a11; pah-1; cyp-33c3* | 0.697 |
| GO:0003702 | RNA polymerase II transcription factor activity | *hlh-1* | 0.698 |
| GO:0005267 | potassium channel activity | *b0281.5; t23b12.6; r05f9.1* | 0.701 |
| GO:0008135 | translation factor activity, nucleic acid binding | *ife-3* | 0.703 |
| GO:0004527 | exonuclease activity | *eri-1* | 0.703 |
| GO:0019899 | enzyme binding | *csn-5* | 0.71 |
| GO:0005506 | iron ion binding | *cyp-13a11; pah-1; ftn-2; cyp-33c3* | 0.72 |
| GO:0008194 | UDP-glycosyltransferase activity | *gly-14* | 0.72 |
| GO:0003779 | actin binding | *pfn-1* | 0.72 |
| GO:0016740 | transferase activity | *gsto-3; pmk-1; jnk-1; c44h4.6; y69f12a.1; ugt-1; w06e11.1; gly-14; tgt-1; c18a3.1; ugt-25; cdk-7; y22d7ar.6; y40b10a.6; lin-2; max-2; t04g9.4; f21f3.2; f40f8.1; gsk-3; k02e11.5; mpk-1; pnk-1; y40b10a.2; k08e3.5; t19b4.3; bre-2; y43c5b.2* | 0.725 |
| GO:0005085 | guanyl-nucleotide exchange factor activity | *tag-218* | 0.726 |
| GO:0005524 | ATP binding | *pmk-1; jnk-1; b0286.3; rpt-6; c44h4.6; lex-1; y69f12a.1; cdk-7; cct-2; k08f4.1; lin-2; ars-2; f55f8.2; max-2; f21f3.2; rab-10; f40f8.1; hum-5; gsk-3; ssl-1; mpk-1; pnk-1; y43c5b.2* | 0.748 |
| GO:0032559 | adenyl ribonucleotide binding | *pmk-1; jnk-1; b0286.3; rpt-6; c44h4.6; lex-1; y69f12a.1; cdk-7; cct-2; k08f4.1; lin-2; ars-2; f55f8.2; max-2; f21f3.2; rab-10; f40f8.1; hum-5; gsk-3; ssl-1; mpk-1; pnk-1; y43c5b.2* | 0.749 |
| GO:0016758 | transferase activity, transferring hexosyl groups | *gly-14; ugt-25; bre-2; ugt-1; k02e11.5* | 0.767 |
| GO:0005179 | hormone activity | *ins-18* | 0.771 |
| GO:0022836 | gated channel activity | *b0281.5; glc-2; t23b12.6; r05f9.1; unc-49* | 0.773 |
| GO:0016301 | kinase activity | *pmk-1; jnk-1; max-2; f21f3.2; c44h4.6; f40f8.1; gsk-3; y69f12a.1; pnk-1; mpk-1; cdk-7; y22d7ar.6; lin-2; y43c5b.2* | 0.783 |
| GO:0030554 | adenyl nucleotide binding | *pmk-1; jnk-1; b0286.3; rpt-6; c44h4.6; lex-1; y69f12a.1; cdk-7; cct-2; k08f4.1; lin-2; ars-2; f55f8.2; max-2; f21f3.2; rab-10; f40f8.1; hum-5; gsk-3; ssl-1; mpk-1; pnk-1; t08g2.3; y43c5b.2* | 0.802 |
| GO:0016810 | hydrolase activity, acting on carbon-nitrogen (but not peptide) bonds | *c10c5.5; c10c5.4* | 0.816 |
| GO:0016779 | nucleotidyltransferase activity | *k08e3.5; w06e11.1* | 0.828 |
| GO:0016773 | phosphotransferase activity, alcohol group as acceptor | *pmk-1; jnk-1; max-2; f21f3.2; c44h4.6; gsk-3; y69f12a.1; pnk-1; mpk-1; cdk-7; y22d7ar.6; lin-2; y43c5b.2* | 0.837 |
| GO:0030414 | protease inhibitor activity | *tag-290; kal-1* | 0.852 |
| GO:0015291 | secondary active transmembrane transporter activity | *nhx-6; h17b01.1* | 0.858 |
| GO:0003700 | transcription factor activity | *hlh-1; unc-130; vab-3; cnd-1; ceh-23; ceh-38; zc204.2; mxl-1; elt-3; vab-15; pha-4; aha-1* | 0.977 |
| GO:0004674 | protein serine/threonine kinase activity | *pmk-1; jnk-1; max-2; f21f3.2; c44h4.6; gsk-3; y69f12a.1; mpk-1; cdk-7; lin-2* | 1 |
| GO:0004672 | protein kinase activity | *pmk-1; jnk-1; max-2; f21f3.2; c44h4.6; gsk-3; y69f12a.1; mpk-1; cdk-7; lin-2; y43c5b.2* | 1 |
| **Cellular Component** |  |  |  |
| GO:0005622 | intracellular | *hlh-1; gsto-3; his-13; col-94; rpt-6; snt-2; r07e5.3; bre-1; dro-1; cuc-1; rpl-11.1; sedl-1; sur-6; pph-5; cki-1; unc-32; zk1236.1; rab-28; tra-1; ife-3; rap-1; c10c5.5; skp-1; f57c9.4; elt-3; hpl-2; hil-3; eri-1; pha-4; lsm-1; rps-22; unc-64; tbg-1; nxt-1; lap-1; ebp-2; cye-1; w09d10.3; ceh-38; gly-14; zc204.2; dlc-2; mdl-1; mxl-1; c10c5.4; unc-130; vab-3; sdc-3; rabs-5; tomm-7; lgg-1; aha-1; col-89; jnk-1; r11.1; cle-1; w06e11.1; gly-8; snr-2; col-91; rpl-25.2; sptf-2; nuc-1; vab-15; asp-4; cpar-1; c09g9.7; his-7; mdt-6; cnd-1; fkb-3; hum-5; mnm-2; tba-1; c37a2.7; zk337.2; his-48; cul-2; tag-218; c18a3.1; evl-20; pfd-3; ars-2; unc-57; c29f9.5; rab-10; f23h12.2; ceh-23; csn-5; sec-8; pfn-1; rab-37; snr-3; hlh-8; apc-10; sbp-1* | 0.000172 |
| GO:0044424 | intracellular part | *hlh-1; gsto-3; his-13; col-94; rpt-6; snt-2; r07e5.3; cuc-1; rpl-11.1; sur-6; pph-5; unc-32; cki-1; tra-1; ife-3; c10c5.5; skp-1; elt-3; hpl-2; hil-3; eri-1; pha-4; lsm-1; rps-22; tbg-1; unc-64; ebp-2; w09d10.3; cye-1; ceh-38; zc204.2; gly-14; dlc-2; mdl-1; mxl-1; c10c5.4; unc-130; vab-3; sdc-3; tomm-7; lgg-1; aha-1; col-89; jnk-1; r11.1; cle-1; w06e11.1; gly-8; col-91; snr-2; rpl-25.2; nuc-1; vab-15; asp-4; c09g9.7; cpar-1; mdt-6; his-7; cnd-1; fkb-3; hum-5; tba-1; c37a2.7; his-48; cul-2; c18a3.1; evl-20; pfd-3; ars-2; unc-57; c29f9.5; f23h12.2; ceh-23; csn-5; sec-8; pfn-1; snr-3; hlh-8; apc-10; sbp-1* | 0.000364 |
| GO:0032991 | macromolecular complex | *his-13; b0286.3; snr-2; rpl-11.1; rpl-25.2; sur-6; cpar-1; b0281.5; his-7; unc-32; t23b12.6; hum-5; gpc-2; tba-1; hil-3; rps-22; lsm-1; tbg-1; c37a2.7; ebp-2; cye-1; w09d10.3; his-48; dlc-2; r05f9.1; pfd-3; f23h12.2; csn-5; sec-8; snr-3; apc-10; fem-2* | 0.000826 |
| GO:0043229 | intracellular organelle | *hlh-1; his-13; jnk-1; r11.1; rpt-6; snt-2; r07e5.3; w06e11.1; gly-8; rpl-11.1; rpl-25.2; nuc-1; vab-15; asp-4; cpar-1; c09g9.7; his-7; pph-5; mdt-6; cnd-1; fkb-3; cki-1; unc-32; tra-1; hum-5; skp-1; tba-1; elt-3; hpl-2; hil-3; pha-4; rps-22; tbg-1; unc-64; c37a2.7; ebp-2; cye-1; w09d10.3; ceh-38; his-48; cul-2; gly-14; zc204.2; c18a3.1; dlc-2; mdl-1; mxl-1; evl-20; unc-130; c29f9.5; vab-3; sdc-3; f23h12.2; ceh-23; csn-5; pfn-1; apc-10; hlh-8; tomm-7; lgg-1; sbp-1; aha-1* | 0.00166 |
| GO:0043226 | organelle | *hlh-1; his-13; jnk-1; r11.1; rpt-6; snt-2; r07e5.3; w06e11.1; gly-8; rpl-11.1; rpl-25.2; nuc-1; vab-15; asp-4; cpar-1; c09g9.7; his-7; pph-5; mdt-6; cnd-1; fkb-3; cki-1; unc-32; tra-1; hum-5; skp-1; tba-1; elt-3; hpl-2; hil-3; pha-4; rps-22; tbg-1; unc-64; c37a2.7; ebp-2; cye-1; w09d10.3; ceh-38; his-48; cul-2; gly-14; zc204.2; c18a3.1; dlc-2; mdl-1; mxl-1; evl-20; unc-130; c29f9.5; vab-3; sdc-3; f23h12.2; ceh-23; csn-5; pfn-1; apc-10; hlh-8; tomm-7; lgg-1; sbp-1; aha-1* | 0.00166 |
| GO:0043234 | protein complex | *tbg-1; his-13; b0286.3; ebp-2; cye-1; his-48; dlc-2; r05f9.1; sur-6; pfd-3; cpar-1; b0281.5; his-7; unc-32; t23b12.6; f23h12.2; gpc-2; hum-5; csn-5; sec-8; tba-1; apc-10; hil-3; fem-2* | 0.00653 |
| GO:0043231 | intracellular membrane-bound organelle | *hlh-1; his-13; jnk-1; r11.1; rpt-6; snt-2; r07e5.3; w06e11.1; gly-8; nuc-1; vab-15; asp-4; cpar-1; c09g9.7; his-7; mdt-6; pph-5; cnd-1; fkb-3; cki-1; unc-32; tra-1; skp-1; elt-3; hpl-2; hil-3; pha-4; unc-64; cye-1; ceh-38; his-48; cul-2; gly-14; zc204.2; c18a3.1; mdl-1; mxl-1; c29f9.5; unc-130; vab-3; sdc-3; f23h12.2; ceh-23; csn-5; apc-10; hlh-8; tomm-7; lgg-1; sbp-1; aha-1* | 0.00653 |
| GO:0043227 | membrane-bound organelle | *hlh-1; his-13; jnk-1; r11.1; rpt-6; snt-2; r07e5.3; w06e11.1; gly-8; nuc-1; vab-15; asp-4; cpar-1; c09g9.7; his-7; mdt-6; pph-5; cnd-1; fkb-3; cki-1; unc-32; tra-1; skp-1; elt-3; hpl-2; hil-3; pha-4; unc-64; cye-1; ceh-38; his-48; cul-2; gly-14; zc204.2; c18a3.1; mdl-1; mxl-1; c29f9.5; unc-130; vab-3; sdc-3; f23h12.2; ceh-23; csn-5; apc-10; hlh-8; tomm-7; lgg-1; sbp-1; aha-1* | 0.00654 |
| GO:0005737 | cytoplasm | *col-89; gsto-3; jnk-1; col-94; r11.1; rpt-6; snt-2; cle-1; cuc-1; gly-8; col-91; rpl-11.1; rpl-25.2; asp-4; pph-5; unc-32; fkb-3; tra-1; ife-3; c10c5.5; hpl-2; eri-1; rps-22; tbg-1; c37a2.7; w09d10.3; cul-2; gly-14; c10c5.4; evl-20; pfd-3; ars-2; unc-57; f23h12.2; sec-8; csn-5; tomm-7; lgg-1* | 0.0158 |
| GO:0005634 | nucleus | *hlh-1; his-13; jnk-1; rpt-6; r07e5.3; w06e11.1; vab-15; nuc-1; cpar-1; c09g9.7; mdt-6; pph-5; his-7; cki-1; cnd-1; tra-1; skp-1; elt-3; hpl-2; hil-3; pha-4; unc-64; cye-1; ceh-38; cul-2; his-48; zc204.2; c18a3.1; mdl-1; mxl-1; c29f9.5; unc-130; sdc-3; vab-3; ceh-23; csn-5; hlh-8; apc-10; sbp-1; aha-1* | 0.0177 |
| GO:0031594 | neuromuscular junction | *unc-57; unc-49* | 0.0415 |
| GO:0005741 | mitochondrial outer membrane | *tomm-7; f23h12.2* | 0.0735 |
| GO:0031968 | organelle outer membrane | *tomm-7; f23h12.2* | 0.0905 |
| GO:0005773 | vacuole | *unc-32; lgg-1; asp-4* | 0.0907 |
| GO:0019867 | outer membrane | *tomm-7; f23h12.2* | 0.102 |
| GO:0043232 | intracellular non-membrane-bound organelle | *tbg-1; his-13; c37a2.7; ebp-2; w09d10.3; r07e5.3; his-48; dlc-2; rpl-11.1; rpl-25.2; evl-20; cpar-1; his-7; sdc-3; hum-5; pfn-1; tba-1; hpl-2; hil-3; rps-22* | 0.107 |
| GO:0043228 | non-membrane-bound organelle | *tbg-1; his-13; c37a2.7; ebp-2; w09d10.3; r07e5.3; his-48; dlc-2; rpl-11.1; rpl-25.2; evl-20; cpar-1; his-7; sdc-3; hum-5; pfn-1; tba-1; hpl-2; hil-3; rps-22* | 0.107 |
| GO:0005742 | mitochondrial outer membrane translocase complex | *f23h12.2* | 0.114 |
| GO:0005776 | autophagic vacuole | *lgg-1* | 0.114 |
| GO:0000307 | cyclin-dependent protein kinase holoenzyme complex | *cye-1* | 0.114 |
| GO:0008287 | protein serine/threonine phosphatase complex | *sur-6; fem-2* | 0.114 |
| GO:0005874 | microtubule | *tbg-1; tba-1; ebp-2* | 0.138 |
| GO:0000786 | nucleosome | *cpar-1; his-13; his-7; hil-3; his-48* | 0.153 |
| GO:0044446 | intracellular organelle part | *tbg-1; his-13; r11.1; ebp-2; r07e5.3; his-48; gly-14; dlc-2; evl-20; cpar-1; his-7; unc-32; f23h12.2; hum-5; csn-5; tba-1; apc-10; tomm-7; hpl-2; hil-3* | 0.153 |
| GO:0005694 | chromosome | *cpar-1; his-13; his-7; sdc-3; ebp-2; r07e5.3; his-48; hpl-2; hil-3* | 0.153 |
| GO:0044422 | organelle part | *tbg-1; his-13; r11.1; ebp-2; r07e5.3; his-48; gly-14; dlc-2; evl-20; cpar-1; his-7; unc-32; f23h12.2; hum-5; csn-5; tba-1; apc-10; tomm-7; hpl-2; hil-3* | 0.153 |
| GO:0019861 | flagellum | *tnc-2* | 0.153 |
| GO:0009288 | flagellin-based flagellum | *tnc-2* | 0.153 |
| GO:0009320 | phosphoribosylaminoimidazole carboxylase complex | *b0286.3* | 0.153 |
| GO:0008180 | signalosome | *csn-5* | 0.153 |
| GO:0044427 | chromosomal part | *cpar-1; his-13; his-7; ebp-2; hpl-2; hil-3; his-48* | 0.153 |
| GO:0043025 | cell soma | *unc-64; jnk-1; rab-10* | 0.153 |
| GO:0044459 | plasma membrane part | *unc-64; b0281.5; t23b12.6; gpc-2; cle-1; inx-9; ajm-1; r05f9.1; lin-2* | 0.153 |
| GO:0030054 | cell junction | *ajm-1; cle-1; lin-2; inx-9* | 0.153 |
| GO:0000785 | chromatin | *cpar-1; his-13; his-7; hpl-2; hil-3; his-48* | 0.153 |
| GO:0015630 | microtubule cytoskeleton | *tbg-1; tba-1; dlc-2; ebp-2; evl-20* | 0.169 |
| GO:0031966 | mitochondrial membrane | *r11.1; tomm-7; f23h12.2* | 0.171 |
| GO:0044464 | cell part | *hlh-1; gsto-3; his-13; col-94; rpt-6; y57g11c.23; snt-2; r07e5.3; bre-1; dro-1; cuc-1; rpl-11.1; sedl-1; h17b01.1; lin-2; sur-6; f42g10.1; b0281.5; pph-5; y41g9a.4; cki-1; zk1236.1; unc-32; rab-28; tra-1; ife-3; rap-1; inx-9; c10c5.5; skp-1; f57c9.4; elt-3; hpl-2; hil-3; eri-1; pha-4; lsm-1; rps-22; unc-64; tbg-1; nxt-1; lap-1; ebp-2; cye-1; w09d10.3; ceh-38; y40b1b.8; gly-14; zc204.2; dlc-2; mdl-1; mxl-1; c10c5.4; unc-130; vab-3; sdc-3; c50d2.2; emo-1; rabs-5; y51b9a.6; tomm-7; lgg-1; aha-1; col-89; jnk-1; r11.1; cle-1; w06e11.1; gly-8; col-91; snr-2; rpl-25.2; nuc-1; vab-15; sptf-2; asp-4; y19d10b.6; cpar-1; c09g9.7; tnc-2; his-7; mdt-6; nhx-6; cnd-1; fkb-3; t23b12.6; gpc-2; hum-5; kal-1; unc-49; mnm-2; tba-1; c51e3.6; c37a2.7; zk337.2; his-48; cul-2; tag-218; c18a3.1; glc-2; evl-20; r05f9.1; ars-2; pfd-3; unc-57; c29f9.5; sra-21; rab-10; f23h12.2; ceh-23; tsp-3; csn-5; sec-8; pfn-1; ajm-1; rab-37; snr-3; hlh-8; apc-10; bre-2; sbp-1; fem-2* | 0.173 |
| GO:0005680 | anaphase-promoting complex | *apc-10* | 0.173 |
| GO:0000145 | exocyst | *sec-8* | 0.173 |
| GO:0000159 | protein phosphatase type 2A complex | *sur-6* | 0.173 |
| GO:0030529 | ribonucleoprotein complex | *c37a2.7; w09d10.3; snr-3; rpl-11.1; snr-2; rpl-25.2; lsm-1; rps-22* | 0.177 |
| GO:0044430 | cytoskeletal part | *tbg-1; tba-1; dlc-2; ebp-2; hum-5; evl-20* | 0.204 |
| GO:0000803 | sex chromosome | *sdc-3* | 0.209 |
| GO:0000805 | X chromosome | *sdc-3* | 0.209 |
| GO:0044444 | cytoplasmic part | *tbg-1; c37a2.7; r11.1; snt-2; w09d10.3; gly-14; cuc-1; gly-8; rpl-11.1; rpl-25.2; asp-4; pfd-3; fkb-3; unc-32; f23h12.2; sec-8; tomm-7; lgg-1; hpl-2; rps-22* | 0.223 |
| GO:0005740 | mitochondrial envelope | *r11.1; tomm-7; f23h12.2* | 0.231 |
| GO:0005886 | plasma membrane | *unc-64; b0281.5; t23b12.6; gpc-2; cle-1; inx-9; ajm-1; r05f9.1; evl-20; lin-2* | 0.231 |
| GO:0016327 | apicolateral plasma membrane | *ajm-1* | 0.231 |
| GO:0000152 | nuclear ubiquitin ligase complex | *apc-10* | 0.231 |
| GO:0043296 | apical junction complex | *ajm-1* | 0.231 |
| GO:0005856 | cytoskeleton | *tbg-1; tba-1; dlc-2; ebp-2; hum-5; evl-20; pfn-1* | 0.246 |
| GO:0044455 | mitochondrial membrane part | *f23h12.2* | 0.26 |
| GO:0005818 | aster | *evl-20* | 0.26 |
| GO:0008076 | voltage-gated potassium channel complex | *b0281.5; t23b12.6; r05f9.1* | 0.262 |
| GO:0005829 | cytosol | *cuc-1; pfd-3* | 0.262 |
| GO:0005941 | unlocalized protein complex | *b0286.3* | 0.285 |
| GO:0044429 | mitochondrial part | *r11.1; tomm-7; f23h12.2* | 0.303 |
| GO:0044448 | cell cortex part | *sec-8* | 0.307 |
| GO:0016272 | prefoldin complex | *pfd-3* | 0.307 |
| GO:0005764 | lysosome | *asp-4* | 0.327 |
| GO:0005834 | heterotrimeric G-protein complex | *gpc-2* | 0.327 |
| GO:0000323 | lytic vacuole | *asp-4* | 0.327 |
| GO:0019897 | extrinsic to plasma membrane | *gpc-2* | 0.334 |
| GO:0033176 | proton-transporting V-type ATPase complex | *unc-32* | 0.334 |
| GO:0016471 | vacuolar proton-transporting V-type ATPase complex | *unc-32* | 0.334 |
| GO:0000776 | kinetochore | *ebp-2* | 0.334 |
| GO:0005913 | cell-cell adherens junction | *ajm-1* | 0.334 |
| GO:0005774 | vacuolar membrane | *unc-32* | 0.354 |
| GO:0044437 | vacuolar part | *unc-32* | 0.354 |
| GO:0005578 | proteinaceous extracellular matrix | *cle-1; fbl-1* | 0.36 |
| GO:0000151 | ubiquitin ligase complex | *apc-10* | 0.372 |
| GO:0031090 | organelle membrane | *gly-14; r11.1; unc-32; tomm-7; f23h12.2* | 0.38 |
| GO:0005840 | ribosome | *c37a2.7; rpl-11.1; rpl-25.2; w09d10.3; rps-22* | 0.384 |
| GO:0044421 | extracellular region part | *cle-1; fbl-1* | 0.387 |
| GO:0005911 | intercellular junction | *ajm-1; inx-9* | 0.387 |
| GO:0015629 | actin cytoskeleton | *hum-5; pfn-1* | 0.39 |
| GO:0009986 | cell surface | *kal-1* | 0.39 |
| GO:0016323 | basolateral plasma membrane | *unc-64* | 0.39 |
| GO:0005604 | basement membrane | *cle-1* | 0.443 |
| GO:0019898 | extrinsic to membrane | *gpc-2* | 0.443 |
| GO:0008021 | synaptic vesicle | *snt-2* | 0.443 |
| GO:0005912 | adherens junction | *ajm-1* | 0.454 |
| GO:0000775 | chromosome, pericentric region | *ebp-2* | 0.454 |
| GO:0044445 | cytosolic part | *pfd-3* | 0.469 |
| GO:0044420 | extracellular matrix part | *cle-1* | 0.502 |
| GO:0016459 | myosin complex | *hum-5* | 0.515 |
| GO:0030136 | clathrin-coated vesicle | *snt-2* | 0.528 |
| GO:0005813 | centrosome | *tbg-1* | 0.539 |
| GO:0000139 | Golgi membrane | *gly-14* | 0.545 |
| GO:0005815 | microtubule organizing center | *tbg-1* | 0.545 |
| GO:0031967 | organelle envelope | *r11.1; tomm-7; f23h12.2* | 0.549 |
| GO:0005783 | endoplasmic reticulum | *fkb-3; gly-8; hpl-2* | 0.549 |
| GO:0014704 | intercalated disc | *inx-9* | 0.549 |
| GO:0005921 | gap junction | *inx-9* | 0.549 |
| GO:0005739 | mitochondrion | *r11.1; tomm-7; f23h12.2* | 0.549 |
| GO:0031975 | envelope | *r11.1; tomm-7; f23h12.2* | 0.549 |
| GO:0005887 | integral to plasma membrane | *b0281.5; t23b12.6; r05f9.1* | 0.577 |
| GO:0031226 | intrinsic to plasma membrane | *b0281.5; t23b12.6; r05f9.1* | 0.577 |
| GO:0030135 | coated vesicle | *snt-2* | 0.577 |
| GO:0000228 | nuclear chromosome | *r07e5.3* | 0.577 |
| GO:0005875 | microtubule associated complex | *dlc-2* | 0.578 |
| GO:0005819 | spindle | *evl-20* | 0.578 |
| GO:0005743 | mitochondrial inner membrane | *r11.1* | 0.585 |
| GO:0019866 | organelle inner membrane | *r11.1* | 0.615 |
| GO:0044431 | Golgi apparatus part | *gly-14* | 0.621 |
| GO:0016023 | cytoplasmic membrane-bound vesicle | *snt-2* | 0.626 |
| GO:0005938 | cell cortex | *sec-8* | 0.641 |
| GO:0031988 | membrane-bound vesicle | *snt-2* | 0.655 |
| GO:0031410 | cytoplasmic vesicle | *snt-2* | 0.658 |
| GO:0042995 | cell projection | *tnc-2; jnk-1* | 0.776 |
