# Supplementary material for: Large-scale functional RNAi screen in C. elegans identifies genes that regulate the dysfunction of mutant polyglutamine neurons
Source: BMC Genomics. 2012 Mar 13;13:91. doi: 10.1186/1471-2164-13-91 (PMC3331833; doi:10.1186/1471-2164-13-91)
Supplement: Additional file 8 — Table S7. Gene Ontology classification of genes that aggravated 128Q-neuron dysfunction when knocked-down by RNAi. [file 1471-2164-13-91-S8.DOC]

**Supplementary Table 7.** Gene Ontology classification of genes that aggravate 128Q-neuron dysfunction when knocked-down by RNAi.

Genes were classified based on their functional annotations in the GO categories ‘Biological Process’, ‘Molecular Function’ and ‘Cellular component’ as indicated in the sub-headings of the table. While *P* < 0.05 was considered significant, *P* values for all of the GO terms is shown.

| **GO ID** | **GO description** | **Genes** | **P value** |
| --- | --- | --- | --- |
| **Biological Process** |  |  |  |
| GO:0051649 | establishment of cellular localization | *bmk-1; unc-101; vps-26; ooc-3; aps-2; f45g2.4; klp-4; cap-2; tag-170; tba-6; dab-1; vps-54; unc-84; ddp-1* | 0.0304 |
| GO:0007275 | multicellular organismal development | *sel-8; c35d10.5; tag-170; mup-2; col-14; e02d9.1; ads-1; t22f3.3; eps-8; rpl-22; k07h8.1; anc-1; tra-2; y54g9a.7; d1054.14; eat-1; cyp-31a5; asf-1; rnr-2; eft-3; let-92; pod-2; e02a10.1; pek-1; aps-2; cap-2; ogt-1; dao-3; his-37; ccf-1; f43g9.12; nlp-39; y54e10a.12; r05g9.3; f45g2.4; f22b3.4; r02d3.5; btb-6; crn-1; y51h1a.3; agt-2; ifg-1; tag-203; c49c3.6; dab-1; grd-5; t24h7.3; bli-1; ooc-3; npp-20; col-84; gpi-1; spon-1; cpr-1; y87g2a.1; inx-21; pad-1; tct-1; f28d1.1; vhp-1; pro-1; lin-41; rpl-5; pri-1; unc-84; r05f9.6; calu-1; sdz-28; tre-1* | 0.0304 |
| GO:0051641 | cellular localization | *bmk-1; unc-101; vps-26; ooc-3; aps-2; f45g2.4; klp-4; cap-2; tag-170; tba-6; dab-1; vps-54; unc-84; ddp-1* | 0.0304 |
| GO:0032502 | developmental process | *sel-8; c35d10.5; tag-170; mup-2; col-14; e02d9.1; ads-1; t22f3.3; eps-8; rpl-22; k07h8.1; anc-1; tra-2; y54g9a.7; d1054.14; eat-1; cyp-31a5; asf-1; rnr-2; eft-3; let-92; pod-2; e02a10.1; pek-1; aps-2; cap-2; ogt-1; dao-3; his-37; ccf-1; f43g9.12; nlp-39; y54e10a.12; r05g9.3; f45g2.4; f22b3.4; oma-1; r02d3.5; btb-6; crn-1; y51h1a.3; agt-2; bag-1; ifg-1; tag-203; c49c3.6; dab-1; grd-5; t24h7.3; bli-1; ooc-3; npp-20; col-84; gpi-1; spon-1; cpr-1; y87g2a.1; inx-21; pad-1; tct-1; f28d1.1; vhp-1; pro-1; lin-41; rpl-5; pri-1; unc-84; r05f9.6; calu-1; sdz-28; tre-1* | 0.0347 |
| GO:0016043 | cellular component organization and biogenesis | *bmk-1; vps-26; pro-1; y48a6b.3; aps-2; klp-4; crn-1; cap-2; bag-1; tag-170; his-37; mup-2; dab-1; fhod-1; vps-54; unc-84; ddp-1; unc-101; ooc-3; f45g2.4; anc-1; asf-1; tba-6* | 0.0878 |
| GO:0009790 | embryonic development | *sel-8; r02d3.5; btb-6; crn-1; y51h1a.3; agt-2; tag-170; c35d10.5; ifg-1; tag-203; c49c3.6; mup-2; dab-1; grd-5; ads-1; t24h7.3; t22f3.3; eps-8; rpl-22; bli-1; ooc-3; k07h8.1; npp-20; col-84; y54g9a.7; gpi-1; d1054.14; cpr-1; asf-1; inx-21; cyp-31a5; rnr-2; pad-1; tct-1; let-92; eft-3; f28d1.1; pod-2; e02a10.1; aps-2; cap-2; rpl-5; his-37; ccf-1; pri-1; unc-84; f43g9.12; nlp-39; r05g9.3; y54e10a.12; r05f9.6; f22b3.4; sdz-28; tre-1* | 0.107 |
| GO:0006996 | organelle organization and biogenesis | *bmk-1; ooc-3; pro-1; y48a6b.3; klp-4; crn-1; anc-1; cap-2; his-37; mup-2; tba-6; asf-1; fhod-1; ddp-1* | 0.113 |
| GO:0046907 | intracellular transport | *bmk-1; unc-101; vps-26; aps-2; f45g2.4; klp-4; tba-6; vps-54; ddp-1* | 0.113 |
| GO:0018988 | molting cycle, protein-based cuticle | *tag-170; calu-1; bli-1; dab-1; e02d9.1; anc-1* | 0.113 |
| GO:0042303 | molting cycle | *tag-170; calu-1; bli-1; dab-1; e02d9.1; anc-1* | 0.113 |
| GO:0009792 | embryonic development ending in birth or egg hatching | *sel-8; r02d3.5; crn-1; y51h1a.3; agt-2; tag-170; c35d10.5; ifg-1; tag-203; c49c3.6; dab-1; grd-5; ads-1; t24h7.3; t22f3.3; eps-8; rpl-22; bli-1; ooc-3; k07h8.1; npp-20; col-84; y54g9a.7; gpi-1; d1054.14; asf-1; inx-21; cyp-31a5; rnr-2; pad-1; tct-1; let-92; eft-3; f28d1.1; pod-2; e02a10.1; aps-2; cap-2; rpl-5; his-37; ccf-1; pri-1; unc-84; f43g9.12; nlp-39; r05g9.3; y54e10a.12; r05f9.6; f22b3.4; sdz-28; tre-1* | 0.15 |
| GO:0007626 | locomotory behavior | *tct-1; fat-3; r02d3.5; pod-2; crn-1; tag-170; lin-41; mup-2; his-37; dab-1; e02d9.1; f43g9.12; unc-84; unc-101; eps-8; calu-1; bli-1; dnj-1; f45g2.4; npp-20; trap-4; eat-1; c18e9.2; y87g2a.1* | 0.162 |
| GO:0000910 | cytokinesis | *t22f3.3; tag-170; f22b3.4; let-92; cyp-31a5; pri-1* | 0.191 |
| GO:0048513 | organ development | *sel-8; let-92; r02d3.5; pro-1; crn-1; rpl-5; tag-170; lin-41; tag-203; col-14; mup-2; ccf-1; e02d9.1; f43g9.12; unc-84; tra-2; y87g2a.1; rnr-2* | 0.191 |
| GO:0032787 | monocarboxylic acid metabolic process | *dao-3; gpi-1; fat-3; zc416.6* | 0.191 |
| GO:0040035 | hermaphrodite genitalia development | *sel-8; r02d3.5; pro-1; tra-2; crn-1; tag-170; rpl-5; tag-203; col-14; ccf-1; y87g2a.1; e02d9.1; f43g9.12; rnr-2* | 0.191 |
| GO:0007010 | cytoskeleton organization and biogenesis | *bmk-1; ooc-3; klp-4; anc-1; cap-2; mup-2; tba-6; fhod-1* | 0.191 |
| GO:0050790 | regulation of catalytic activity | *tag-274; vhp-1; y46g5a.1; e01g4.1* | 0.194 |
| GO:0048806 | genitalia development | *sel-8; r02d3.5; pro-1; tra-2; crn-1; tag-170; rpl-5; tag-203; col-14; ccf-1; y87g2a.1; e02d9.1; f43g9.12; rnr-2* | 0.197 |
| GO:0065009 | regulation of a molecular function | *tag-274; vhp-1; y46g5a.1; e01g4.1* | 0.197 |
| GO:0051728 | cell cycle switching, mitotic to meiotic cell cycle | *sel-8; ccf-1* | 0.21 |
| GO:0051729 | germline cell cycle switching, mitotic to meiotic cell cycle | *sel-8; ccf-1* | 0.21 |
| GO:0007526 | larval somatic muscle development | *mup-2* | 0.21 |
| GO:0046329 | negative regulation of JNK cascade | *vhp-1* | 0.21 |
| GO:0006990 | positive regulation of transcription of target genes involved in unfolded protein response | *pek-1* | 0.21 |
| GO:0000188 | inactivation of MAPK activity | *vhp-1* | 0.21 |
| GO:0006890 | retrograde vesicle-mediated transport, Golgi to ER | *f45g2.4* | 0.21 |
| GO:0007525 | somatic muscle development | *mup-2* | 0.21 |
| GO:0042147 | retrograde transport, endosome to Golgi | *vps-54* | 0.21 |
| GO:0048731 | system development | *sel-8; let-92; r02d3.5; pro-1; crn-1; rpl-5; tag-170; lin-41; tag-203; col-14; mup-2; ccf-1; e02d9.1; f43g9.12; unc-84; tra-2; y87g2a.1; rnr-2* | 0.21 |
| GO:0016053 | organic acid biosynthetic process | *fat-3; zc416.6* | 0.21 |
| GO:0046394 | carboxylic acid biosynthetic process | *fat-3; zc416.6* | 0.21 |
| GO:0006633 | fatty acid biosynthetic process | *fat-3; zc416.6* | 0.21 |
| GO:0006112 | energy reserve metabolic process | *tre-1; ogt-1* | 0.216 |
| GO:0007018 | microtubule-based movement | *bmk-1; tba-6; klp-4* | 0.216 |
| GO:0030705 | cytoskeleton-dependent intracellular transport | *bmk-1; tba-6; klp-4* | 0.216 |
| GO:0048518 | positive regulation of biological process | *fat-3; y48a6b.3; crn-1; c35d10.5; ifg-1; mup-2; dab-1; e02d9.1; eps-8; rpl-22; f40f11.4; anc-1; npp-20; d1054.14; y87g2a.1; pad-1; tct-1; f28d1.1; let-92; vhp-1; e02a10.1; col-17; pro-1; pek-1; cap-2; lin-41; rpl-5; ddl-3; his-37; ccf-1; f43g9.12; f45g2.4; y40b1b.5* | 0.216 |
| GO:0016192 | vesicle-mediated transport | *unc-101; dab-1; aps-2; f45g2.4; vps-54* | 0.216 |
| GO:0030036 | actin cytoskeleton organization and biogenesis | *mup-2; fhod-1; cap-2* | 0.216 |
| GO:0040010 | positive regulation of growth rate | *tct-1; fat-3; f28d1.1; e02a10.1; pro-1; y48a6b.3; crn-1; cap-2; c35d10.5; rpl-5; ddl-3; ifg-1; his-37; mup-2; dab-1; e02d9.1; f43g9.12; eps-8; rpl-22; f40f11.4; f45g2.4; npp-20; anc-1; d1054.14; y40b1b.5; y87g2a.1; pad-1* | 0.216 |
| GO:0040009 | regulation of growth rate | *tct-1; fat-3; f28d1.1; e02a10.1; pro-1; y48a6b.3; crn-1; cap-2; c35d10.5; rpl-5; ddl-3; ifg-1; his-37; mup-2; dab-1; e02d9.1; f43g9.12; eps-8; rpl-22; f40f11.4; f45g2.4; npp-20; anc-1; d1054.14; y40b1b.5; y87g2a.1; pad-1* | 0.216 |
| GO:0043549 | regulation of kinase activity | *tag-274; vhp-1* | 0.216 |
| GO:0051338 | regulation of transferase activity | *tag-274; vhp-1* | 0.216 |
| GO:0045859 | regulation of protein kinase activity | *tag-274; vhp-1* | 0.216 |
| GO:0030029 | actin filament-based process | *mup-2; fhod-1; cap-2* | 0.216 |
| GO:0007548 | sex differentiation | *sel-8; r02d3.5; pro-1; tra-2; crn-1; tag-170; rpl-5; tag-203; col-14; ccf-1; y87g2a.1; e02d9.1; f43g9.12; rnr-2* | 0.216 |
| GO:0006493 | protein amino acid O-linked glycosylation | *ogt-1* | 0.216 |
| GO:0043407 | negative regulation of MAP kinase activity | *vhp-1* | 0.216 |
| GO:0019370 | leukotriene biosynthetic process | *zc416.6* | 0.216 |
| GO:0006690 | icosanoid metabolic process | *zc416.6* | 0.216 |
| GO:0018346 | protein amino acid prenylation | *r02d3.5* | 0.216 |
| GO:0006691 | leukotriene metabolic process | *zc416.6* | 0.216 |
| GO:0046328 | regulation of JNK cascade | *vhp-1* | 0.216 |
| GO:0043450 | alkene biosynthetic process | *zc416.6* | 0.216 |
| GO:0043193 | positive regulation of gene-specific transcription | *pek-1* | 0.216 |
| GO:0046456 | icosanoid biosynthetic process | *zc416.6* | 0.216 |
| GO:0043449 | alkene metabolic process | *zc416.6* | 0.216 |
| GO:0032583 | regulation of gene-specific transcription | *pek-1* | 0.216 |
| GO:0018342 | protein prenylation | *r02d3.5* | 0.216 |
| GO:0007097 | nuclear migration | *tag-170; unc-84; cap-2* | 0.217 |
| GO:0006461 | protein complex assembly | *unc-101; aps-2; f45g2.4* | 0.224 |
| GO:0032313 | regulation of Rab GTPase activity | *y46g5a.1; e01g4.1* | 0.224 |
| GO:0032483 | regulation of Rab protein signal transduction | *y46g5a.1; e01g4.1* | 0.224 |
| GO:0032318 | regulation of Ras GTPase activity | *y46g5a.1; e01g4.1* | 0.224 |
| GO:0032482 | Rab protein signal transduction | *y46g5a.1; e01g4.1* | 0.224 |
| GO:0040008 | regulation of growth | *tct-1; fat-3; f28d1.1; e02a10.1; col-17; pro-1; y48a6b.3; crn-1; cap-2; c35d10.5; lin-41; rpl-5; ddl-3; ifg-1; his-37; mup-2; dab-1; ccf-1; e02d9.1; f43g9.12; eps-8; rpl-22; f40f11.4; f45g2.4; npp-20; anc-1; d1054.14; y40b1b.5; c18e9.2; y87g2a.1; pad-1* | 0.224 |
| GO:0040023 | establishment of nucleus localization | *tag-170; unc-84; cap-2* | 0.224 |
| GO:0051647 | nucleus localization | *tag-170; unc-84; cap-2* | 0.224 |
| GO:0016477 | cell migration | *pro-1; dab-1; mig-1; unc-84* | 0.23 |
| GO:0045927 | positive regulation of growth | *tct-1; fat-3; f28d1.1; e02a10.1; col-17; pro-1; y48a6b.3; crn-1; cap-2; c35d10.5; lin-41; rpl-5; ddl-3; ifg-1; his-37; mup-2; dab-1; ccf-1; e02d9.1; f43g9.12; eps-8; rpl-22; f40f11.4; f45g2.4; npp-20; anc-1; d1054.14; y40b1b.5; y87g2a.1; pad-1* | 0.23 |
| GO:0045682 | regulation of epidermis development | *lin-41* | 0.23 |
| GO:0045604 | regulation of epidermal cell differentiation | *lin-41* | 0.23 |
| GO:0046688 | response to copper ion | *vhp-1* | 0.23 |
| GO:0006269 | DNA replication, synthesis of RNA primer | *pri-1* | 0.23 |
| GO:0051348 | negative regulation of transferase activity | *vhp-1* | 0.23 |
| GO:0007034 | vacuolar transport | *vps-26* | 0.23 |
| GO:0006469 | negative regulation of protein kinase activity | *vhp-1* | 0.23 |
| GO:0033673 | negative regulation of kinase activity | *vhp-1* | 0.23 |
| GO:0065007 | biological regulation | *tag-274; sel-8; fat-3; t01d3.2; btb-6; mig-1; y48a6b.3; crn-1; bag-1; c35d10.5; ifg-1; y46g5a.1; mup-2; dab-1; e02d9.1; eps-8; rpl-22; ooc-3; k09c4.5; f40f11.4; npp-20; anc-1; d1054.14; c18e9.2; y87g2a.1; pad-1; tct-1; f28d1.1; let-92; pod-2; vhp-1; pro-1; e02a10.1; col-17; pek-1; nhr-62; cap-2; cey-2; rpl-5; lin-41; ddl-3; his-37; ccf-1; unc-84; f43g9.12; e01g4.1; calu-1; f45g2.4; y40b1b.5* | 0.23 |
| GO:0007281 | germ cell development | *oma-1; pro-1; cyp-31a5* | 0.23 |
| GO:0042254 | ribosome biogenesis and assembly | *pro-1; y48a6b.3* | 0.23 |
| GO:0003006 | reproductive developmental process | *sel-8; r02d3.5; pro-1; tra-2; crn-1; tag-170; rpl-5; tag-203; col-14; ccf-1; y87g2a.1; e02d9.1; f43g9.12; rnr-2* | 0.23 |
| GO:0040016 | embryonic cleavage | *t22f3.3; tag-170; f22b3.4; let-92; cyp-31a5; pri-1* | 0.23 |
| GO:0006928 | cell motility | *pro-1; dab-1; mig-1; unc-84* | 0.23 |
| GO:0051674 | localization of cell | *pro-1; dab-1; mig-1; unc-84* | 0.23 |
| GO:0009059 | macromolecule biosynthetic process | *eft-3; r02d3.5; rpl-22; e02a10.1; ogt-1; gpi-1; lin-41; rpl-5; f22b3.4* | 0.23 |
| GO:0048856 | anatomical structure development | *sel-8; let-92; r02d3.5; vhp-1; pro-1; crn-1; cap-2; bag-1; lin-41; tag-170; rpl-5; ifg-1; tag-203; col-14; mup-2; ccf-1; e02d9.1; f43g9.12; unc-84; bli-1; ooc-3; tra-2; y87g2a.1; rnr-2* | 0.23 |
| GO:0006096 | glycolysis | *gpi-1; pgk-1* | 0.23 |
| GO:0048469 | cell maturation | *oma-1; mup-2* | 0.23 |
| GO:0021700 | developmental maturation | *oma-1; mup-2* | 0.23 |
| GO:0051656 | establishment of organelle localization | *tag-170; ooc-3; unc-84; cap-2* | 0.247 |
| GO:0008340 | determination of adult life span | *dao-3; lin-41; ifg-1; cpr-1; eat-1; rnr-2* | 0.248 |
| GO:0010259 | multicellular organismal aging | *dao-3; lin-41; ifg-1; cpr-1; eat-1; rnr-2* | 0.248 |
| GO:0051640 | organelle localization | *tag-170; ooc-3; unc-84; cap-2* | 0.248 |
| GO:0007568 | aging | *dao-3; lin-41; ifg-1; cpr-1; eat-1; rnr-2* | 0.248 |
| GO:0006471 | protein amino acid ADP-ribosylation | *pme-2* | 0.248 |
| GO:0043086 | negative regulation of catalytic activity | *vhp-1* | 0.248 |
| GO:0048730 | epidermis morphogenesis | *lin-41* | 0.248 |
| GO:0009913 | epidermal cell differentiation | *lin-41* | 0.248 |
| GO:0007413 | axonal fasciculation | *mup-2* | 0.248 |
| GO:0016197 | endosome transport | *vps-54* | 0.248 |
| GO:0002009 | morphogenesis of an epithelium | *lin-41; r02d3.5; vhp-1; y87g2a.1; tra-2; crn-1; rnr-2* | 0.248 |
| GO:0015980 | energy derivation by oxidation of organic compounds | *tre-1; ogt-1* | 0.263 |
| GO:0019752 | carboxylic acid metabolic process | *dao-3; gpi-1; fat-3; y73f4a.3; zc416.6; c12c8.2* | 0.27 |
| GO:0006082 | organic acid metabolic process | *dao-3; gpi-1; fat-3; y73f4a.3; zc416.6; c12c8.2* | 0.27 |
| GO:0009966 | regulation of signal transduction | *vhp-1; y46g5a.1; ooc-3; e01g4.1* | 0.271 |
| GO:0007007 | inner mitochondrial membrane organization and biogenesis | *ddp-1* | 0.271 |
| GO:0006094 | gluconeogenesis | *gpi-1* | 0.271 |
| GO:0009186 | deoxyribonucleoside diphosphate metabolic process | *rnr-2* | 0.271 |
| GO:0009396 | folic acid and derivative biosynthetic process | *dao-3* | 0.271 |
| GO:0045214 | sarcomere organization | *mup-2* | 0.271 |
| GO:0045039 | protein import into mitochondrial inner membrane | *ddp-1* | 0.271 |
| GO:0043405 | regulation of MAP kinase activity | *vhp-1* | 0.271 |
| GO:0007006 | mitochondrial membrane organization and biogenesis | *ddp-1* | 0.271 |
| GO:0007610 | behavior | *tct-1; fat-3; r02d3.5; pod-2; crn-1; tag-170; lin-41; mup-2; his-37; dab-1; e02d9.1; f43g9.12; unc-84; unc-101; eps-8; calu-1; bli-1; dnj-1; f45g2.4; npp-20; trap-4; eat-1; c18e9.2; y87g2a.1* | 0.271 |
| GO:0006007 | glucose catabolic process | *gpi-1; pgk-1* | 0.271 |
| GO:0046365 | monosaccharide catabolic process | *gpi-1; pgk-1* | 0.271 |
| GO:0019320 | hexose catabolic process | *gpi-1; pgk-1* | 0.271 |
| GO:0043087 | regulation of GTPase activity | *y46g5a.1; e01g4.1* | 0.271 |
| GO:0019953 | sexual reproduction | *tct-1; eft-3; pme-2; pro-1; npp-20; tra-2; cap-2; tag-170; lin-41; oma-1; y46g5a.1; mup-2; y87g2a.1; cyp-31a5; e02d9.1; pad-1* | 0.274 |
| GO:0022414 | reproductive process | *sel-8; r02d3.5; pro-1; crn-1; cap-2; rpl-5; tag-170; tag-203; col-14; mup-2; dab-1; ccf-1; e02d9.1; unc-84; f43g9.12; tra-2; y87g2a.1; rnr-2* | 0.275 |
| GO:0051056 | regulation of small GTPase mediated signal transduction | *y46g5a.1; ooc-3; e01g4.1* | 0.286 |
| GO:0030334 | regulation of cell migration | *mig-1; unc-84* | 0.286 |
| GO:0006090 | pyruvate metabolic process | *gpi-1* | 0.286 |
| GO:0006626 | protein targeting to mitochondrion | *ddp-1* | 0.286 |
| GO:0006760 | folic acid and derivative metabolic process | *dao-3* | 0.286 |
| GO:0016049 | cell growth | *bag-1* | 0.286 |
| GO:0043681 | protein import into mitochondrion | *ddp-1* | 0.286 |
| GO:0007292 | female gamete generation | *oma-1; mup-2; cyp-31a5* | 0.286 |
| GO:0046164 | alcohol catabolic process | *gpi-1; pgk-1* | 0.286 |
| GO:0051270 | regulation of cell motility | *mig-1; unc-84* | 0.286 |
| GO:0051336 | regulation of hydrolase activity | *y46g5a.1; e01g4.1* | 0.286 |
| GO:0050896 | response to stimulus | *tct-1; fat-3; r02d3.5; vhp-1; pod-2; pek-1; crn-1; bag-1; lin-41; tag-170; his-37; mup-2; dab-1; e02d9.1; f43g9.12; unc-84; unc-101; eps-8; calu-1; f49e12.1; bli-1; dnj-1; f45g2.4; npp-20; trap-4; eat-1; c18e9.2; y87g2a.1* | 0.301 |
| GO:0006006 | glucose metabolic process | *gpi-1; pgk-1* | 0.301 |
| GO:0016051 | carbohydrate biosynthetic process | *gpi-1; f22b3.4* | 0.301 |
| GO:0009132 | nucleoside diphosphate metabolic process | *rnr-2* | 0.301 |
| GO:0006497 | protein amino acid lipidation | *r02d3.5* | 0.301 |
| GO:0005991 | trehalose metabolic process | *tre-1* | 0.301 |
| GO:0005984 | disaccharide metabolic process | *tre-1* | 0.301 |
| GO:0007254 | JNK cascade | *vhp-1* | 0.301 |
| GO:0001522 | pseudouridine synthesis | *tag-124* | 0.301 |
| GO:0031098 | stress-activated protein kinase signaling pathway | *vhp-1* | 0.301 |
| GO:0042158 | lipoprotein biosynthetic process | *r02d3.5* | 0.301 |
| GO:0050789 | regulation of biological process | *sel-8; fat-3; t01d3.2; btb-6; mig-1; y48a6b.3; crn-1; c35d10.5; ifg-1; y46g5a.1; mup-2; dab-1; e02d9.1; eps-8; rpl-22; k09c4.5; ooc-3; f40f11.4; npp-20; anc-1; d1054.14; c18e9.2; y87g2a.1; pad-1; tct-1; f28d1.1; let-92; vhp-1; e02a10.1; col-17; pro-1; pek-1; nhr-62; cap-2; cey-2; rpl-5; lin-41; ddl-3; his-37; ccf-1; unc-84; f43g9.12; e01g4.1; f45g2.4; y40b1b.5* | 0.304 |
| GO:0009987 | cellular process | *sel-8; fat-3; t06a4.3; c34f11.5; mig-1; y48a6b.3; klp-4; tag-170; y46g5a.1; mup-2; y73f4a.3; e02d9.1; vps-54; t22f3.3; c28a5.6; rpl-22; k09c4.5; anc-1; pgk-1; asf-1; tba-6; cyp-31a5; rnr-2; c49h3.4; let-92; eft-3; e02a10.1; zc416.6; pek-1; aps-2; cap-2; ogt-1; dao-3; k09f6.3; his-37; ccf-1; fhod-1; e01g4.1; f43g9.12; unc-101; f45g2.4; f22b3.4; oma-1; tag-274; bmk-1; r02d3.5; try-2; t01d3.2; vps-26; crn-1; bag-1; ifg-1; dab-1; ddp-1; grd-5; c37h5.3; f44f1.3; pme-2; ooc-3; dnj-1; r11f4.2; gpi-1; ubc-17; spon-1; cpr-1; syd-1; vhp-1; pro-1; f57b10.9; nhr-62; cey-2; c12c8.2; rpl-5; lin-41; pri-1; unc-84; rrc-1; tag-124; gpa-1; zk829.1* | 0.306 |
| GO:0048599 | oocyte development | *oma-1; cyp-31a5* | 0.306 |
| GO:0009994 | oocyte differentiation | *oma-1; cyp-31a5* | 0.306 |
| GO:0032989 | cellular structure morphogenesis | *bag-1; ooc-3; mup-2* | 0.306 |
| GO:0000902 | cell morphogenesis | *bag-1; ooc-3; mup-2* | 0.306 |
| GO:0006631 | fatty acid metabolic process | *fat-3; zc416.6* | 0.306 |
| GO:0007398 | ectoderm development | *lin-41* | 0.306 |
| GO:0010038 | response to metal ion | *vhp-1* | 0.306 |
| GO:0000578 | embryonic axis specification | *cap-2* | 0.306 |
| GO:0030728 | ovulation | *mup-2* | 0.306 |
| GO:0008544 | epidermis development | *lin-41* | 0.306 |
| GO:0007205 | protein kinase C activation | *tag-274* | 0.306 |
| GO:0008037 | cell recognition | *mup-2* | 0.306 |
| GO:0006839 | mitochondrial transport | *ddp-1* | 0.306 |
| GO:0008038 | neuron recognition | *mup-2* | 0.306 |
| GO:0009058 | biosynthetic process | *fat-3; eft-3; r02d3.5; rpl-22; f57c2.5; e02a10.1; zc416.6; ogt-1; gpi-1; dao-3; rpl-5; lin-41; f22b3.4; zk829.1* | 0.319 |
| GO:0005975 | carbohydrate metabolic process | *t22f3.3; gpi-1; srh-61; f22b3.4; r05f9.6; pgk-1; tre-1* | 0.324 |
| GO:0042157 | lipoprotein metabolic process | *r02d3.5* | 0.324 |
| GO:0009306 | protein secretion | *dab-1* | 0.324 |
| GO:0040034 | regulation of development, heterochronic | *lin-41* | 0.324 |
| GO:0009262 | deoxyribonucleotide metabolic process | *rnr-2* | 0.324 |
| GO:0010035 | response to inorganic substance | *vhp-1* | 0.324 |
| GO:0007200 | G-protein signaling, coupled to IP3 second messenger (phospholipase C activating) | *tag-274* | 0.324 |
| GO:0032147 | activation of protein kinase activity | *tag-274* | 0.324 |
| GO:0006725 | aromatic compound metabolic process | *dao-3; c37h5.3; zk829.1* | 0.325 |
| GO:0030104 | water homeostasis | *ddl-3; eps-8; calu-1; pod-2; c18e9.2* | 0.332 |
| GO:0040006 | protein-based cuticle attachment to epithelium | *tag-170; bli-1* | 0.334 |
| GO:0040004 | collagen and cuticulin-based cuticle attachment to epithelium | *tag-170; bli-1* | 0.334 |
| GO:0006309 | DNA fragmentation during apoptosis | *crn-1* | 0.335 |
| GO:0017148 | negative regulation of translation | *lin-41* | 0.335 |
| GO:0030262 | apoptotic nuclear changes | *crn-1* | 0.335 |
| GO:0046165 | alcohol biosynthetic process | *gpi-1* | 0.335 |
| GO:0046364 | monosaccharide biosynthetic process | *gpi-1* | 0.335 |
| GO:0019319 | hexose biosynthetic process | *gpi-1* | 0.335 |
| GO:0042001 | hermaphrodite somatic sex determination | *tra-2* | 0.335 |
| GO:0035046 | pronuclear migration | *tag-170; cap-2* | 0.34 |
| GO:0051301 | cell division | *t22f3.3; tag-170; f22b3.4; let-92; cyp-31a5; pri-1* | 0.34 |
| GO:0048193 | Golgi vesicle transport | *f45g2.4* | 0.34 |
| GO:0031327 | negative regulation of cellular biosynthetic process | *lin-41* | 0.34 |
| GO:0001556 | oocyte maturation | *oma-1* | 0.34 |
| GO:0000165 | MAPKKK cascade | *vhp-1* | 0.34 |
| GO:0009890 | negative regulation of biosynthetic process | *lin-41* | 0.34 |
| GO:0006921 | cell structure disassembly during apoptosis | *crn-1* | 0.34 |
| GO:0006308 | DNA catabolic process | *crn-1* | 0.34 |
| GO:0022613 | ribonucleoprotein complex biogenesis and assembly | *pro-1; y48a6b.3* | 0.34 |
| GO:0007017 | microtubule-based process | *bmk-1; ooc-3; tba-6; klp-4* | 0.34 |
| GO:0008104 | protein localization | *unc-101; ooc-3; c18e9.2; dab-1; aps-2; ddp-1* | 0.34 |
| GO:0051234 | establishment of localization | *bmk-1; fat-3; f14f11.1; vps-26; aps-2; klp-4; cap-2; tag-170; c24g7.1; f31e8.4; col-14; y54g9a.4; dab-1; vps-54; unc-84; ddp-1; unc-101; bli-1; ooc-3; k09c4.5; f45g2.4; col-84; aqp-2; f55g1.12; c18e9.2; tba-6; aqp-4* | 0.34 |
| GO:0022404 | molting cycle process | *tag-170; bli-1* | 0.34 |
| GO:0018996 | molting cycle, collagen and cuticulin-based cuticle | *tag-170; bli-1* | 0.34 |
| GO:0019318 | hexose metabolic process | *gpi-1; pgk-1* | 0.34 |
| GO:0033036 | macromolecule localization | *unc-101; ooc-3; c18e9.2; dab-1; aps-2; ddp-1* | 0.34 |
| GO:0005996 | monosaccharide metabolic process | *gpi-1; pgk-1* | 0.34 |
| GO:0006814 | sodium ion transport | *c24g7.1; f55g1.12* | 0.34 |
| GO:0006937 | regulation of muscle contraction | *mup-2* | 0.34 |
| GO:0006547 | histidine metabolic process | *y73f4a.3* | 0.34 |
| GO:0008361 | regulation of cell size | *bag-1* | 0.34 |
| GO:0006986 | response to unfolded protein | *pek-1* | 0.34 |
| GO:0006548 | histidine catabolic process | *y73f4a.3* | 0.34 |
| GO:0009075 | histidine family amino acid metabolic process | *y73f4a.3* | 0.34 |
| GO:0051789 | response to protein stimulus | *pek-1* | 0.34 |
| GO:0007005 | mitochondrion organization and biogenesis | *ddp-1* | 0.34 |
| GO:0030968 | unfolded protein response | *pek-1* | 0.34 |
| GO:0009077 | histidine family amino acid catabolic process | *y73f4a.3* | 0.34 |
| GO:0006984 | ER-nuclear signaling pathway | *pek-1* | 0.34 |
| GO:0007276 | gamete generation | *tct-1; eft-3; pme-2; pro-1; tra-2; npp-20; lin-41; oma-1; y46g5a.1; mup-2; cyp-31a5; y87g2a.1; e02d9.1; pad-1* | 0.356 |
| GO:0045860 | positive regulation of protein kinase activity | *tag-274* | 0.356 |
| GO:0018993 | somatic sex determination | *tra-2* | 0.356 |
| GO:0033674 | positive regulation of kinase activity | *tag-274* | 0.356 |
| GO:0051347 | positive regulation of transferase activity | *tag-274* | 0.356 |
| GO:0006825 | copper ion transport | *f31e8.4* | 0.356 |
| GO:0002164 | larval development | *sel-8; eft-3; let-92; vhp-1; e02a10.1; pro-1; pek-1; aps-2; ogt-1; cap-2; rpl-5; ifg-1; his-37; mup-2; ccf-1; e02d9.1; pri-1; f43g9.12; unc-84; ads-1; eps-8; rpl-22; calu-1; bli-1; f45g2.4; npp-20; anc-1; d1054.14* | 0.361 |
| GO:0001703 | gastrulation with mouth forming first | *tag-203; crn-1* | 0.361 |
| GO:0048468 | cell development | *bag-1; oma-1; pro-1; mup-2; cyp-31a5; crn-1* | 0.361 |
| GO:0006066 | alcohol metabolic process | *gpi-1; pgk-1; zk829.1* | 0.361 |
| GO:0045445 | myoblast differentiation | *mup-2* | 0.361 |
| GO:0051146 | striated muscle cell differentiation | *mup-2* | 0.361 |
| GO:0048627 | myoblast development | *mup-2* | 0.361 |
| GO:0048628 | myoblast maturation | *mup-2* | 0.361 |
| GO:0030239 | myofibril assembly | *mup-2* | 0.361 |
| GO:0006414 | translational elongation | *eft-3* | 0.361 |
| GO:0031032 | actomyosin structure organization and biogenesis | *mup-2* | 0.361 |
| GO:0055002 | striated muscle cell development | *mup-2* | 0.361 |
| GO:0040012 | regulation of locomotion | *vhp-1; dab-1; mig-1; unc-84* | 0.367 |
| GO:0032940 | secretion by cell | *dab-1; f45g2.4* | 0.367 |
| GO:0046578 | regulation of Ras protein signal transduction | *y46g5a.1; e01g4.1* | 0.367 |
| GO:0048015 | phosphoinositide-mediated signaling | *tag-274* | 0.367 |
| GO:0007243 | protein kinase cascade | *vhp-1* | 0.367 |
| GO:0051248 | negative regulation of protein metabolic process | *lin-41* | 0.367 |
| GO:0055001 | muscle cell development | *mup-2* | 0.367 |
| GO:0009451 | RNA modification | *tag-124* | 0.367 |
| GO:0051239 | regulation of multicellular organismal process | *mup-2* | 0.367 |
| GO:0022411 | cellular component disassembly | *crn-1* | 0.367 |
| GO:0009566 | fertilization | *tag-170; cap-2* | 0.369 |
| GO:0007338 | single fertilization | *tag-170; cap-2* | 0.369 |
| GO:0018987 | osmoregulation | *ddl-3; eps-8; calu-1; pod-2; c18e9.2* | 0.378 |
| GO:0050878 | regulation of body fluid levels | *ddl-3; eps-8; calu-1; pod-2; c18e9.2* | 0.378 |
| GO:0043085 | positive regulation of catalytic activity | *tag-274* | 0.378 |
| GO:0016072 | rRNA metabolic process | *pro-1* | 0.378 |
| GO:0006364 | rRNA processing | *pro-1* | 0.378 |
| GO:0006260 | DNA replication | *pri-1; crn-1* | 0.378 |
| GO:0045184 | establishment of protein localization | *unc-101; c18e9.2; dab-1; aps-2; ddp-1* | 0.378 |
| GO:0006915 | apoptosis | *bag-1; crn-1* | 0.383 |
| GO:0022607 | cellular component assembly | *unc-101; his-37; mup-2; aps-2; f45g2.4* | 0.383 |
| GO:0048741 | skeletal muscle fiber development | *mup-2* | 0.383 |
| GO:0014706 | striated muscle development | *mup-2* | 0.383 |
| GO:0007519 | skeletal muscle development | *mup-2* | 0.383 |
| GO:0006417 | regulation of translation | *lin-41* | 0.383 |
| GO:0009607 | response to biotic stimulus | *pek-1* | 0.383 |
| GO:0048747 | muscle fiber development | *mup-2* | 0.383 |
| GO:0048729 | tissue morphogenesis | *lin-41* | 0.397 |
| GO:0009889 | regulation of biosynthetic process | *lin-41* | 0.397 |
| GO:0009968 | negative regulation of signal transduction | *vhp-1* | 0.397 |
| GO:0031326 | regulation of cellular biosynthetic process | *lin-41* | 0.397 |
| GO:0048477 | oogenesis | *oma-1; cyp-31a5* | 0.402 |
| GO:0007265 | Ras protein signal transduction | *y46g5a.1; e01g4.1* | 0.402 |
| GO:0030001 | metal ion transport | *c24g7.1; f14f11.1; f31e8.4; f55g1.12; y54g9a.4* | 0.402 |
| GO:0007163 | establishment and/or maintenance of cell polarity | *ooc-3* | 0.402 |
| GO:0040021 | hermaphrodite germ-line sex determination | *tra-2* | 0.402 |
| GO:0009798 | axis specification | *cap-2* | 0.402 |
| GO:0000041 | transition metal ion transport | *f31e8.4* | 0.402 |
| GO:0030154 | cell differentiation | *bag-1; lin-41; oma-1; pro-1; mup-2; cyp-31a5; crn-1* | 0.402 |
| GO:0048878 | chemical homeostasis | *ddl-3; eps-8; calu-1; pod-2; c18e9.2* | 0.402 |
| GO:0009791 | post-embryonic development | *sel-8; eft-3; let-92; r02d3.5; vhp-1; e02a10.1; pro-1; pek-1; aps-2; ogt-1; cap-2; rpl-5; ifg-1; his-37; mup-2; ccf-1; e02d9.1; pri-1; f43g9.12; unc-84; ads-1; eps-8; rpl-22; calu-1; bli-1; f45g2.4; npp-20; anc-1; d1054.14* | 0.402 |
| GO:0048869 | cellular developmental process | *bag-1; lin-41; oma-1; pro-1; mup-2; cyp-31a5; crn-1* | 0.406 |
| GO:0042692 | muscle cell differentiation | *mup-2* | 0.416 |
| GO:0019538 | protein metabolic process | *eft-3; r02d3.5; try-2; vhp-1; e02a10.1; f57b10.9; t06a4.3; c34f11.5; pek-1; zc416.6; aps-2; ogt-1; bag-1; lin-41; rpl-5; k09f6.3; e02d9.1; unc-101; f44f1.3; c28a5.6; pme-2; rpl-22; dnj-1; f45g2.4; ubc-17; cpr-1; tba-6* | 0.426 |
| GO:0002119 | larval development (sensu Nematoda) | *sel-8; eft-3; let-92; vhp-1; e02a10.1; pro-1; pek-1; aps-2; ogt-1; cap-2; rpl-5; ifg-1; his-37; ccf-1; e02d9.1; pri-1; f43g9.12; unc-84; ads-1; eps-8; rpl-22; calu-1; bli-1; f45g2.4; npp-20; anc-1; d1054.14* | 0.426 |
| GO:0046903 | secretion | *dab-1; f45g2.4* | 0.426 |
| GO:0040019 | positive regulation of embryonic development | *btb-6* | 0.428 |
| GO:0044275 | cellular carbohydrate catabolic process | *gpi-1; pgk-1* | 0.431 |
| GO:0045995 | regulation of embryonic development | *btb-6* | 0.442 |
| GO:0006412 | translation | *lin-41; rpl-5; eft-3; rpl-22; e02a10.1* | 0.446 |
| GO:0016052 | carbohydrate catabolic process | *gpi-1; pgk-1* | 0.446 |
| GO:0003012 | muscle system process | *mup-2* | 0.446 |
| GO:0009880 | embryonic pattern specification | *cap-2* | 0.446 |
| GO:0006936 | muscle contraction | *mup-2* | 0.446 |
| GO:0008632 | apoptotic program | *crn-1* | 0.446 |
| GO:0017038 | protein import | *ddp-1* | 0.446 |
| GO:0044249 | cellular biosynthetic process | *fat-3; eft-3; rpl-22; e02a10.1; zc416.6; dao-3; gpi-1; rpl-5; lin-41; zk829.1* | 0.446 |
| GO:0040015 | negative regulation of multicellular organism growth | *rpl-22; c18e9.2* | 0.446 |
| GO:0007399 | nervous system development | *mup-2; unc-84* | 0.446 |
| GO:0007369 | gastrulation | *tag-203; crn-1* | 0.449 |
| GO:0045926 | negative regulation of growth | *rpl-22; c18e9.2* | 0.449 |
| GO:0051258 | protein polymerization | *tba-6* | 0.449 |
| GO:0009063 | amino acid catabolic process | *y73f4a.3* | 0.449 |
| GO:0018992 | germ-line sex determination | *tra-2* | 0.449 |
| GO:0008610 | lipid biosynthetic process | *fat-3; zc416.6* | 0.45 |
| GO:0006457 | protein folding | *bag-1; dnj-1* | 0.45 |
| GO:0008105 | asymmetric protein localization | *ooc-3* | 0.46 |
| GO:0007409 | axonogenesis | *mup-2* | 0.471 |
| GO:0006261 | DNA-dependent DNA replication | *pri-1* | 0.471 |
| GO:0051726 | regulation of cell cycle | *sel-8; ccf-1* | 0.473 |
| GO:0043170 | macromolecule metabolic process | *r02d3.5; try-2; t01d3.2; t06a4.3; c34f11.5; crn-1; bag-1; ifg-1; e02d9.1; t22f3.3; f44f1.3; c28a5.6; rpl-22; pme-2; k09c4.5; dnj-1; gpi-1; ubc-17; cpr-1; pgk-1; asf-1; tba-6; c49h3.4; eft-3; vhp-1; pro-1; e02a10.1; f57b10.9; zc416.6; pek-1; nhr-62; aps-2; ogt-1; cey-2; rpl-5; lin-41; k09f6.3; his-37; pri-1; unc-101; srh-61; r05f9.6; f45g2.4; f22b3.4; tag-124; tre-1* | 0.473 |
| GO:0065003 | macromolecular complex assembly | *unc-101; his-37; aps-2; f45g2.4* | 0.478 |
| GO:0019932 | second-messenger-mediated signaling | *tag-274* | 0.478 |
| GO:0043284 | biopolymer biosynthetic process | *eft-3* | 0.478 |
| GO:0007242 | intracellular signaling cascade | *tag-274; vhp-1; y46g5a.1; ooc-3; c34f11.5; pek-1; crn-1; e01g4.1* | 0.479 |
| GO:0012501 | programmed cell death | *bag-1; crn-1* | 0.48 |
| GO:0009888 | tissue development | *lin-41* | 0.482 |
| GO:0044270 | nitrogen compound catabolic process | *y73f4a.3* | 0.482 |
| GO:0007308 | oocyte construction | *cyp-31a5* | 0.482 |
| GO:0040026 | positive regulation of vulval development | *let-92* | 0.482 |
| GO:0009310 | amine catabolic process | *y73f4a.3* | 0.482 |
| GO:0065008 | regulation of biological quality | *bag-1; ddl-3; eps-8; calu-1; pod-2; c18e9.2* | 0.49 |
| GO:0015031 | protein transport | *unc-101; c18e9.2; aps-2; ddp-1* | 0.496 |
| GO:0000904 | cellular morphogenesis during differentiation | *mup-2* | 0.5 |
| GO:0048667 | neuron morphogenesis during differentiation | *mup-2* | 0.5 |
| GO:0048812 | neurite morphogenesis | *mup-2* | 0.5 |
| GO:0043050 | pharyngeal pumping | *eat-1* | 0.507 |
| GO:0019290 | siderophore biosynthetic process | *zk829.1* | 0.507 |
| GO:0009239 | enterobactin biosynthetic process | *zk829.1* | 0.507 |
| GO:0051094 | positive regulation of developmental process | *let-92* | 0.507 |
| GO:0051246 | regulation of protein metabolic process | *lin-41* | 0.507 |
| GO:0040002 | collagen and cuticulin-based cuticle development | *bli-1* | 0.507 |
| GO:0009237 | siderophore metabolic process | *zk829.1* | 0.507 |
| GO:0042755 | eating behavior | *eat-1* | 0.507 |
| GO:0009712 | catechol metabolic process | *zk829.1* | 0.507 |
| GO:0019540 | siderophore biosynthetic process from catechol | *zk829.1* | 0.507 |
| GO:0031175 | neurite development | *mup-2* | 0.507 |
| GO:0009238 | enterobactin metabolic process | *zk829.1* | 0.507 |
| GO:0007592 | protein-based cuticle development | *bli-1* | 0.51 |
| GO:0006352 | transcription initiation | *his-37* | 0.51 |
| GO:0042335 | cuticle development | *bli-1* | 0.51 |
| GO:0007530 | sex determination | *tra-2* | 0.517 |
| GO:0006997 | nuclear organization and biogenesis | *crn-1* | 0.517 |
| GO:0045595 | regulation of cell differentiation | *lin-41* | 0.517 |
| GO:0045944 | positive regulation of transcription from RNA polymerase II promoter | *pek-1* | 0.528 |
| GO:0018958 | phenol metabolic process | *zk829.1* | 0.533 |
| GO:0045893 | positive regulation of transcription, DNA-dependent | *pek-1* | 0.533 |
| GO:0048523 | negative regulation of cellular process | *lin-41; vhp-1* | 0.533 |
| GO:0006810 | transport | *bmk-1; fat-3; f14f11.1; vps-26; aps-2; klp-4; c24g7.1; f31e8.4; col-14; y54g9a.4; dab-1; vps-54; ddp-1; unc-101; bli-1; k09c4.5; f45g2.4; col-84; aqp-2; f55g1.12; c18e9.2; tba-6; aqp-4* | 0.537 |
| GO:0006605 | protein targeting | *ddp-1* | 0.537 |
| GO:0040018 | positive regulation of multicellular organism growth | *lin-41; rpl-5; fat-3; rpl-22; col-17; ccf-1; npp-20; cap-2* | 0.537 |
| GO:0040017 | positive regulation of locomotion | *vhp-1; dab-1* | 0.55 |
| GO:0000132 | establishment of mitotic spindle orientation | *ooc-3* | 0.56 |
| GO:0051294 | establishment of spindle orientation | *ooc-3* | 0.56 |
| GO:0007631 | feeding behavior | *eat-1* | 0.56 |
| GO:0045941 | positive regulation of transcription | *pek-1* | 0.56 |
| GO:0009653 | anatomical structure morphogenesis | *r02d3.5; vhp-1; ooc-3; tra-2; crn-1; cap-2; bag-1; lin-41; ifg-1; tag-203; mup-2; y87g2a.1; rnr-2* | 0.56 |
| GO:0035264 | multicellular organism growth | *lin-41; rpl-5; fat-3; rpl-22; c18e9.2; col-17; ccf-1; npp-20; cap-2* | 0.56 |
| GO:0040014 | regulation of multicellular organism growth | *lin-41; rpl-5; fat-3; rpl-22; c18e9.2; col-17; ccf-1; npp-20; cap-2* | 0.56 |
| GO:0048598 | embryonic morphogenesis | *mup-2* | 0.56 |
| GO:0045935 | positive regulation of nucleobase, nucleoside, nucleotide and nucleic acid metabolic process | *pek-1* | 0.56 |
| GO:0031325 | positive regulation of cellular metabolic process | *pek-1* | 0.56 |
| GO:0006979 | response to oxidative stress | *f49e12.1* | 0.56 |
| GO:0042592 | homeostatic process | *ddl-3; eps-8; calu-1; pod-2; c18e9.2* | 0.56 |
| GO:0007517 | muscle development | *mup-2* | 0.564 |
| GO:0006486 | protein amino acid glycosylation | *ogt-1* | 0.564 |
| GO:0009101 | glycoprotein biosynthetic process | *ogt-1* | 0.564 |
| GO:0042221 | response to chemical stimulus | *vhp-1; f49e12.1; pek-1* | 0.566 |
| GO:0044262 | cellular carbohydrate metabolic process | *gpi-1; pgk-1; tre-1* | 0.566 |
| GO:0046483 | heterocycle metabolic process | *dao-3* | 0.566 |
| GO:0048666 | neuron development | *mup-2* | 0.566 |
| GO:0007389 | pattern specification process | *cap-2* | 0.566 |
| GO:0006886 | intracellular protein transport | *unc-101; aps-2; ddp-1* | 0.575 |
| GO:0009893 | positive regulation of metabolic process | *pek-1* | 0.579 |
| GO:0009100 | glycoprotein metabolic process | *ogt-1* | 0.579 |
| GO:0050793 | regulation of developmental process | *lin-41; let-92; btb-6* | 0.585 |
| GO:0045045 | secretory pathway | *f45g2.4* | 0.6 |
| GO:0031324 | negative regulation of cellular metabolic process | *lin-41* | 0.6 |
| GO:0040024 | dauer larval development | *ogt-1* | 0.603 |
| GO:0032990 | cell part morphogenesis | *mup-2* | 0.603 |
| GO:0048858 | cell projection morphogenesis | *mup-2* | 0.603 |
| GO:0030030 | cell projection organization and biogenesis | *mup-2* | 0.603 |
| GO:0006091 | generation of precursor metabolites and energy | *f49e12.1; pek-1; vps-54; tre-1; ogt-1; y51h1a.3* | 0.603 |
| GO:0007264 | small GTPase mediated signal transduction | *y46g5a.1; ooc-3; e01g4.1* | 0.606 |
| GO:0009057 | macromolecule catabolic process | *gpi-1; pgk-1; crn-1* | 0.61 |
| GO:0006952 | defense response | *vhp-1* | 0.612 |
| GO:0030182 | neuron differentiation | *mup-2* | 0.619 |
| GO:0009892 | negative regulation of metabolic process | *lin-41* | 0.626 |
| GO:0044267 | cellular protein metabolic process | *eft-3; r02d3.5; try-2; vhp-1; e02a10.1; f57b10.9; t06a4.3; c34f11.5; pek-1; zc416.6; ogt-1; bag-1; lin-41; rpl-5; k09f6.3; e02d9.1; f44f1.3; c28a5.6; pme-2; rpl-22; dnj-1; ubc-17; cpr-1; tba-6* | 0.628 |
| GO:0051293 | establishment of spindle localization | *ooc-3* | 0.632 |
| GO:0006752 | group transfer coenzyme metabolic process | *dao-3* | 0.632 |
| GO:0051653 | spindle localization | *ooc-3* | 0.632 |
| GO:0016071 | mRNA metabolic process | *c49h3.4* | 0.632 |
| GO:0040001 | establishment of mitotic spindle localization | *ooc-3* | 0.632 |
| GO:0006950 | response to stress | *bag-1; f49e12.1; pek-1; crn-1* | 0.654 |
| GO:0006512 | ubiquitin cycle | *ubc-17* | 0.655 |
| GO:0044248 | cellular catabolic process | *gpi-1; pgk-1; y73f4a.3; crn-1* | 0.655 |
| GO:0048519 | negative regulation of biological process | *lin-41; rpl-22; vhp-1; c18e9.2* | 0.658 |
| GO:0006357 | regulation of transcription from RNA polymerase II promoter | *pek-1* | 0.664 |
| GO:0044238 | primary metabolic process | *fat-3; r02d3.5; try-2; t01d3.2; t06a4.3; c34f11.5; crn-1; bag-1; ifg-1; y73f4a.3; e02d9.1; t22f3.3; f44f1.3; c28a5.6; rpl-22; pme-2; k09c4.5; dnj-1; gpi-1; ubc-17; cpr-1; pgk-1; asf-1; tba-6; rnr-2; c49h3.4; eft-3; vhp-1; pro-1; e02a10.1; f57b10.9; pek-1; zc416.6; aps-2; nhr-62; cey-2; c12c8.2; ogt-1; rpl-5; lin-41; k09f6.3; his-37; pri-1; f43g9.12; unc-101; srh-61; r05f9.6; f45g2.4; f22b3.4; tag-124; tre-1* | 0.664 |
| GO:0022610 | biological adhesion | *spon-1* | 0.664 |
| GO:0007155 | cell adhesion | *spon-1* | 0.664 |
| GO:0022008 | neurogenesis | *mup-2* | 0.664 |
| GO:0048699 | generation of neurons | *mup-2* | 0.664 |
| GO:0048609 | reproductive process in a multicellular organism | *tag-170; r02d3.5; mup-2; dab-1; unc-84* | 0.703 |
| GO:0032504 | multicellular organism reproduction | *tag-170; r02d3.5; mup-2; dab-1; unc-84* | 0.703 |
| GO:0006366 | transcription from RNA polymerase II promoter | *pek-1* | 0.707 |
| GO:0006812 | cation transport | *c24g7.1; f14f11.1; f31e8.4; f55g1.12; y54g9a.4* | 0.707 |
| GO:0048608 | reproductive structure development | *pro-1* | 0.709 |
| GO:0008406 | gonad development | *pro-1* | 0.709 |
| GO:0044260 | cellular macromolecule metabolic process | *eft-3; r02d3.5; try-2; vhp-1; e02a10.1; f57b10.9; t06a4.3; c34f11.5; pek-1; zc416.6; ogt-1; bag-1; lin-41; rpl-5; k09f6.3; e02d9.1; f44f1.3; c28a5.6; pme-2; rpl-22; dnj-1; ubc-17; cpr-1; tba-6* | 0.742 |
| GO:0006333 | chromatin assembly or disassembly | *his-37; asf-1* | 0.754 |
| GO:0051188 | cofactor biosynthetic process | *dao-3; zk829.1* | 0.766 |
| GO:0006323 | DNA packaging | *his-37; asf-1* | 0.766 |
| GO:0006325 | establishment and/or maintenance of chromatin architecture | *his-37; asf-1* | 0.766 |
| GO:0016265 | death | *bag-1; crn-1* | 0.786 |
| GO:0008219 | cell death | *bag-1; crn-1* | 0.786 |
| GO:0040025 | vulval development | *let-92; unc-84* | 0.786 |
| GO:0006470 | protein amino acid dephosphorylation | *vhp-1; k09f6.3* | 0.789 |
| GO:0006520 | amino acid metabolic process | *y73f4a.3; c12c8.2* | 0.789 |
| GO:0016311 | dephosphorylation | *vhp-1; k09f6.3* | 0.791 |
| GO:0044265 | cellular macromolecule catabolic process | *gpi-1; pgk-1* | 0.791 |
| GO:0006817 | phosphate transport | *bli-1; col-14; col-84* | 0.826 |
| GO:0015698 | inorganic anion transport | *bli-1; col-14; col-84* | 0.843 |
| GO:0022402 | cell cycle process | *sel-8; ooc-3; ccf-1* | 0.845 |
| GO:0006820 | anion transport | *bli-1; col-14; col-84* | 0.847 |
| GO:0009056 | catabolic process | *gpi-1; pgk-1; y73f4a.3; crn-1* | 0.863 |
| GO:0018991 | oviposition | *tag-170; r02d3.5; dab-1; unc-84* | 0.866 |
| GO:0033057 | reproductive behavior in a multicellular organism | *tag-170; r02d3.5; dab-1; unc-84* | 0.866 |
| GO:0006118 | electron transport | *f49e12.1; pek-1; vps-54; y51h1a.3* | 0.866 |
| GO:0019098 | reproductive behavior | *tag-170; r02d3.5; dab-1; unc-84* | 0.867 |
| GO:0006508 | proteolysis | *f44f1.3; try-2; cpr-1; f57b10.9; t06a4.3; zc416.6* | 0.945 |
| GO:0050794 | regulation of cellular process | *sel-8; vhp-1; t01d3.2; ooc-3; k09c4.5; pek-1; mig-1; nhr-62; cey-2; lin-41; y46g5a.1; ccf-1; f43g9.12; e01g4.1; unc-84* | 0.993 |
| GO:0043412 | biopolymer modification | *c28a5.6; r02d3.5; pme-2; vhp-1; c34f11.5; pek-1; ogt-1; bag-1; ubc-17; k09f6.3; tag-124; e02d9.1* | 1 |
| GO:0006464 | protein modification process | *c28a5.6; r02d3.5; pme-2; vhp-1; c34f11.5; pek-1; ogt-1; bag-1; ubc-17; k09f6.3; e02d9.1* | 1 |
| GO:0015672 | monovalent inorganic cation transport | *c24g7.1; f14f11.1; f55g1.12* | 1 |
| **Molecular Function** |  |  |  |
| GO:0030276 | clathrin binding | *dab-1; aps-2* | 0.112 |
| GO:0003779 | actin binding | *zc416.6; fhod-1; anc-1; cap-2* | 0.151 |
| GO:0008565 | protein transporter activity | *unc-101; c18e9.2; aps-2; f45g2.4* | 0.26 |
| GO:0008092 | cytoskeletal protein binding | *zc416.6; fhod-1; anc-1; cap-2* | 0.26 |
| GO:0048256 | flap endonuclease activity | *crn-1* | 0.26 |
| GO:0008097 | 5S rRNA binding | *rpl-5* | 0.26 |
| GO:0004347 | glucose-6-phosphate isomerase activity | *gpi-1* | 0.26 |
| GO:0004645 | phosphorylase activity | *t22f3.3* | 0.26 |
| GO:0016262 | protein N-acetylglucosaminyltransferase activity | *ogt-1* | 0.26 |
| GO:0008579 | JUN kinase phosphatase activity | *vhp-1* | 0.26 |
| GO:0032050 | clathrin heavy chain binding | *dab-1* | 0.26 |
| GO:0005096 | GTPase activator activity | *y46g5a.1; ooc-3; e01g4.1* | 0.286 |
| GO:0033549 | MAP kinase phosphatase activity | *vhp-1* | 0.286 |
| GO:0017017 | MAP kinase tyrosine/serine/threonine phosphatase activity | *vhp-1* | 0.286 |
| GO:0008318 | protein prenyltransferase activity | *r02d3.5* | 0.286 |
| GO:0004360 | glutamine-fructose-6-phosphate transaminase (isomerizing) activity | *f22b3.4* | 0.286 |
| GO:0008409 | 5'-3' exonuclease activity | *crn-1* | 0.286 |
| GO:0004618 | phosphoglycerate kinase activity | *pgk-1* | 0.286 |
| GO:0003951 | NAD+ kinase activity | *y77e11a.2* | 0.286 |
| GO:0008047 | enzyme activator activity | *y46g5a.1; ooc-3; e01g4.1* | 0.286 |
| GO:0005097 | Rab GTPase activator activity | *y46g5a.1; e01g4.1* | 0.29 |
| GO:0019842 | vitamin binding | *t22f3.3; pod-2; cka-2; c12c8.2* | 0.29 |
| GO:0003896 | DNA primase activity | *pri-1* | 0.29 |
| GO:0016844 | strictosidine synthase activity | *f57c2.5* | 0.29 |
| GO:0016774 | phosphotransferase activity, carboxyl group as acceptor | *pgk-1* | 0.29 |
| GO:0016843 | amine-lyase activity | *f57c2.5* | 0.29 |
| GO:0003950 | NAD+ ADP-ribosyltransferase activity | *pme-2* | 0.29 |
| GO:0004197 | cysteine-type endopeptidase activity | *f44f1.3; cpr-1; f57b10.9* | 0.29 |
| GO:0008375 | acetylglucosaminyltransferase activity | *gly-19; ogt-1* | 0.29 |
| GO:0004659 | prenyltransferase activity | *r02d3.5* | 0.29 |
| GO:0016728 | oxidoreductase activity, acting on CH or CH2 groups, disulfide as acceptor | *rnr-2* | 0.29 |
| GO:0004748 | ribonucleoside-diphosphate reductase activity | *rnr-2* | 0.29 |
| GO:0004730 | pseudouridylate synthase activity | *tag-124* | 0.29 |
| GO:0004520 | endodeoxyribonuclease activity | *crn-1* | 0.29 |
| GO:0005099 | Ras GTPase activator activity | *y46g5a.1; e01g4.1* | 0.29 |
| GO:0003777 | microtubule motor activity | *bmk-1; klp-4* | 0.29 |
| GO:0005272 | sodium channel activity | *c24g7.1; f55g1.12* | 0.29 |
| GO:0016725 | oxidoreductase activity, acting on CH or CH2 groups | *rnr-2* | 0.29 |
| GO:0015927 | trehalase activity | *tre-1* | 0.29 |
| GO:0004555 | alpha,alpha-trehalase activity | *tre-1* | 0.29 |
| GO:0016868 | intramolecular transferase activity, phosphotransferases | *r05f9.6* | 0.29 |
| GO:0042329 | structural constituent of collagen and cuticulin-based cuticle | *bli-1* | 0.29 |
| GO:0009374 | biotin binding | *pod-2* | 0.29 |
| GO:0004536 | deoxyribonuclease activity | *crn-1* | 0.29 |
| GO:0005024 | transforming growth factor beta receptor activity | *w05h12.1* | 0.32 |
| GO:0016840 | carbon-nitrogen lyase activity | *f57c2.5* | 0.32 |
| GO:0051087 | chaperone binding | *bag-1* | 0.32 |
| GO:0051015 | actin filament binding | *cap-2* | 0.32 |
| GO:0008194 | UDP-glycosyltransferase activity | *gly-19; ogt-1* | 0.351 |
| GO:0016861 | intramolecular oxidoreductase activity, interconverting aldoses and ketoses | *gpi-1* | 0.357 |
| GO:0005529 | sugar binding | *clec-52; clec-61; clec-207; f22b3.4; clec-265; clec-67; clec-183* | 0.382 |
| GO:0004143 | diacylglycerol kinase activity | *tag-274* | 0.384 |
| GO:0004675 | transmembrane receptor protein serine/threonine kinase activity | *w05h12.1* | 0.384 |
| GO:0016717 | oxidoreductase activity, acting on paired donors, with oxidation of a pair of donors resulting in the reduction of molecular oxygen to two molecules of water | *fat-3* | 0.398 |
| GO:0004500 | dopamine beta-monooxygenase activity | *y73f4a.3* | 0.398 |
| GO:0017151 | DEAD/H-box RNA helicase binding | *let-92* | 0.398 |
| GO:0030695 | GTPase regulator activity | *y46g5a.1; ooc-3; e01g4.1* | 0.398 |
| GO:0003746 | translation elongation factor activity | *eft-3* | 0.415 |
| GO:0016866 | intramolecular transferase activity | *r05f9.6* | 0.415 |
| GO:0005375 | copper ion transmembrane transporter activity | *f31e8.4* | 0.426 |
| GO:0016705 | oxidoreductase activity, acting on paired donors, with incorporation or reduction of molecular oxygen | *fat-3; y73f4a.3* | 0.426 |
| GO:0008235 | metalloexopeptidase activity | *t06a4.3; zc416.6* | 0.426 |
| GO:0016715 | oxidoreductase activity, acting on paired donors, with incorporation or reduction of molecular oxygen, reduced ascorbate as one donor, and incorporation of one atom of oxygen | *y73f4a.3* | 0.426 |
| GO:0016860 | intramolecular oxidoreductase activity | *gpi-1* | 0.426 |
| GO:0032266 | phosphatidylinositol 3-phosphate binding | *zk632.12* | 0.426 |
| GO:0016763 | transferase activity, transferring pentosyl groups | *pme-2* | 0.466 |
| GO:0004198 | calcium-dependent cysteine-type endopeptidase activity | *f44f1.3* | 0.478 |
| GO:0046915 | transition metal ion transmembrane transporter activity | *f31e8.4* | 0.478 |
| GO:0008234 | cysteine-type peptidase activity | *f44f1.3; cpr-1; f57b10.9* | 0.478 |
| GO:0030170 | pyridoxal phosphate binding | *t22f3.3; c12c8.2* | 0.483 |
| GO:0004179 | membrane alanyl aminopeptidase activity | *zc416.6* | 0.487 |
| GO:0031177 | phosphopantetheine binding | *cka-2* | 0.487 |
| GO:0019199 | transmembrane receptor protein kinase activity | *w05h12.1* | 0.487 |
| GO:0016284 | alanine aminopeptidase activity | *zc416.6* | 0.487 |
| GO:0019904 | protein domain specific binding | *dab-1* | 0.487 |
| GO:0046873 | metal ion transmembrane transporter activity | *c24g7.1; f14f11.1; f31e8.4; f55g1.12; y54g9a.4* | 0.489 |
| GO:0016853 | isomerase activity | *gpi-1; r05f9.6* | 0.494 |
| GO:0005083 | small GTPase regulator activity | *y46g5a.1; e01g4.1* | 0.494 |
| GO:0030246 | carbohydrate binding | *clec-52; clec-61; clec-207; f22b3.4; clec-265; clec-67; clec-183* | 0.494 |
| GO:0016986 | transcription initiation factor activity | *his-37* | 0.5 |
| GO:0015082 | di-, tri-valent inorganic cation transmembrane transporter activity | *f31e8.4* | 0.5 |
| GO:0005507 | copper ion binding | *vps-54* | 0.5 |
| GO:0008238 | exopeptidase activity | *t06a4.3; zc416.6* | 0.5 |
| GO:0004182 | carboxypeptidase A activity | *t06a4.3* | 0.5 |
| GO:0050136 | NADH dehydrogenase (quinone) activity | *y51h1a.3* | 0.5 |
| GO:0008137 | NADH dehydrogenase (ubiquinone) activity | *y51h1a.3* | 0.5 |
| GO:0003954 | NADH dehydrogenase activity | *y51h1a.3* | 0.5 |
| GO:0016655 | oxidoreductase activity, acting on NADH or NADPH, quinone or similar compound as acceptor | *y51h1a.3* | 0.5 |
| GO:0016836 | hydro-lyase activity | *tag-124* | 0.5 |
| GO:0004702 | receptor signaling protein serine/threonine kinase activity | *w05h12.1* | 0.5 |
| GO:0004181 | metallocarboxypeptidase activity | *t06a4.3* | 0.5 |
| GO:0004177 | aminopeptidase activity | *zc416.6* | 0.515 |
| GO:0016765 | transferase activity, transferring alkyl or aryl (other than methyl) groups | *r02d3.5* | 0.523 |
| GO:0008483 | transaminase activity | *f22b3.4* | 0.533 |
| GO:0035091 | phosphoinositide binding | *zk632.12* | 0.537 |
| GO:0016835 | carbon-oxygen lyase activity | *tag-124* | 0.537 |
| GO:0003899 | DNA-directed RNA polymerase activity | *pri-1* | 0.537 |
| GO:0003723 | RNA binding | *rpl-5; e02a10.1; e02d9.1; tag-124* | 0.551 |
| GO:0009055 | electron carrier activity | *vps-54; y51h1a.3* | 0.559 |
| GO:0008138 | protein tyrosine/serine/threonine phosphatase activity | *vhp-1* | 0.559 |
| GO:0005057 | receptor signaling protein activity | *w05h12.1* | 0.559 |
| GO:0016651 | oxidoreductase activity, acting on NADH or NADPH | *y51h1a.3* | 0.561 |
| GO:0004519 | endonuclease activity | *crn-1* | 0.561 |
| GO:0003924 | GTPase activity | *eft-3; tba-6* | 0.561 |
| GO:0016757 | transferase activity, transferring glycosyl groups | *t22f3.3; pme-2; gly-19; ogt-1* | 0.561 |
| GO:0008667 | 2,3-dihydro-2,3-dihydroxybenzoate dehydrogenase activity | *zk829.1* | 0.561 |
| GO:0016628 | oxidoreductase activity, acting on the CH-CH group of donors, NAD or NADP as acceptor | *zk829.1* | 0.561 |
| GO:0016769 | transferase activity, transferring nitrogenous groups | *f22b3.4* | 0.561 |
| GO:0004180 | carboxypeptidase activity | *t06a4.3* | 0.561 |
| GO:0005543 | phospholipid binding | *zk632.12* | 0.565 |
| GO:0004553 | hydrolase activity, hydrolyzing O-glycosyl compounds | *srh-61; tre-1* | 0.565 |
| GO:0004527 | exonuclease activity | *crn-1* | 0.565 |
| GO:0008135 | translation factor activity, nucleic acid binding | *eft-3* | 0.565 |
| GO:0019899 | enzyme binding | *let-92* | 0.565 |
| GO:0004601 | peroxidase activity | *f49e12.1* | 0.565 |
| GO:0016684 | oxidoreductase activity, acting on peroxide as acceptor | *f49e12.1* | 0.565 |
| GO:0016798 | hydrolase activity, acting on glycosyl bonds | *srh-61; tre-1* | 0.565 |
| GO:0031072 | heat shock protein binding | *dnj-1* | 0.569 |
| GO:0051082 | unfolded protein binding | *dnj-1* | 0.608 |
| GO:0004252 | serine-type endopeptidase activity | *try-2* | 0.616 |
| GO:0003735 | structural constituent of ribosome | *rpl-5; rpl-22; e02a10.1* | 0.642 |
| GO:0042302 | structural constituent of cuticle | *bli-1; col-14; col-84* | 0.676 |
| GO:0005261 | cation channel activity | *c24g7.1; f14f11.1; f55g1.12* | 0.717 |
| GO:0019001 | guanyl nucleotide binding | *eft-3; tba-6; gpa-1* | 0.725 |
| GO:0019787 | small conjugating protein ligase activity | *ubc-17* | 0.725 |
| GO:0005249 | voltage-gated potassium channel activity | *f14f11.1* | 0.766 |
| GO:0050660 | FAD binding | *ads-1* | 0.766 |
| GO:0008415 | acyltransferase activity | *cpt-2* | 0.777 |
| GO:0016627 | oxidoreductase activity, acting on the CH-CH group of donors | *zk829.1* | 0.777 |
| GO:0022843 | voltage-gated cation channel activity | *f14f11.1* | 0.777 |
| GO:0016881 | acid-amino acid ligase activity | *ubc-17* | 0.777 |
| GO:0016491 | oxidoreductase activity | *fat-3; f49e12.1; y51h1a.3; dhs-7; c07d8.6; y73f4a.3; vps-54; ads-1; rnr-2; zk829.1* | 0.786 |
| GO:0004867 | serine-type endopeptidase inhibitor activity | *spon-1* | 0.789 |
| GO:0022832 | voltage-gated channel activity | *f14f11.1* | 0.8 |
| GO:0005244 | voltage-gated ion channel activity | *f14f11.1* | 0.8 |
| GO:0016779 | nucleotidyltransferase activity | *pri-1* | 0.808 |
| GO:0016829 | lyase activity | *f57c2.5; tag-124* | 0.83 |
| GO:0016301 | kinase activity | *tag-274; y77e11a.2; c28a5.6; c34f11.5; pek-1; f46h5.3; pgk-1; e02d9.1; w05h12.1* | 0.834 |
| GO:0004721 | phosphoprotein phosphatase activity | *vhp-1; k09f6.3* | 0.888 |
| GO:0016758 | transferase activity, transferring hexosyl groups | *t22f3.3; gly-19; ogt-1* | 0.905 |
| GO:0016772 | transferase activity, transferring phosphorus-containing groups | *tag-274; y77e11a.2; c28a5.6; c34f11.5; pek-1; f46h5.3; pgk-1; w05h12.1; e02d9.1; pri-1* | 0.908 |
| GO:0016740 | transferase activity | *tag-274; y77e11a.2; r02d3.5; c34f11.5; pek-1; ogt-1; pri-1; e02d9.1; t22f3.3; c28a5.6; pme-2; f46h5.3; f22b3.4; pgk-1; cpt-2; w05h12.1; gly-19* | 0.917 |
| GO:0048037 | cofactor binding | *t22f3.3; c12c8.2; ads-1* | 0.922 |
| GO:0004175 | endopeptidase activity | *f44f1.3; try-2; cpr-1; f57b10.9* | 0.927 |
| GO:0008233 | peptidase activity | *f44f1.3; try-2; cpr-1; f57b10.9; t06a4.3; zc416.6* | 0.993 |
| GO:0022892 | substrate-specific transporter activity | *unc-101; f14f11.1; aps-2; f45g2.4; c24g7.1; f31e8.4; f55g1.12; c18e9.2; y54g9a.4* | 1 |
| GO:0016773 | phosphotransferase activity, alcohol group as acceptor | *tag-274; y77e11a.2; c28a5.6; c34f11.5; pek-1; e02d9.1; w05h12.1* | 1 |
| **Cellular Component** |  |  |  |
| GO:0030117 | membrane coat | *unc-101; dab-1; aps-2; f45g2.4* | 0.0461 |
| GO:0048475 | coated membrane | *unc-101; dab-1; aps-2; f45g2.4* | 0.0461 |
| GO:0005737 | cytoplasm | *tct-1; eft-3; vhp-1; e02a10.1; pek-1; aps-2; ogt-1; cap-2; y106g6h.1; y51h1a.3; lin-41; rpl-5; col-14; mup-2; dab-1; ddp-1; unc-101; rpl-22; bli-1; ooc-3; f45g2.4; tra-2; anc-1; col-84; f22b3.4; trap-4; oma-1* | 0.0556 |
| GO:0044430 | cytoskeletal part | *bmk-1; oma-1; mup-2; tba-6; klp-4; unc-84; cap-2* | 0.0814 |
| GO:0044444 | cytoplasmic part | *unc-101; rpl-22; ooc-3; e02a10.1; pek-1; f45g2.4; tra-2; aps-2; ogt-1; y106g6h.1; y51h1a.3; rpl-5; trap-4; oma-1; mup-2; dab-1; ddp-1* | 0.0974 |
| GO:0030120 | vesicle coat | *dab-1; f45g2.4* | 0.108 |
| GO:0005856 | cytoskeleton | *bmk-1; oma-1; mup-2; tba-6; klp-4; unc-84; cap-2* | 0.108 |
| GO:0030662 | coated vesicle membrane | *dab-1; f45g2.4* | 0.108 |
| GO:0030659 | cytoplasmic vesicle membrane | *dab-1; f45g2.4* | 0.108 |
| GO:0030119 | AP-type membrane coat adaptor complex | *unc-101; dab-1* | 0.108 |
| GO:0030131 | clathrin adaptor complex | *unc-101; dab-1* | 0.108 |
| GO:0005798 | Golgi-associated vesicle | *dab-1; f45g2.4* | 0.108 |
| GO:0005861 | troponin complex | *mup-2* | 0.108 |
| GO:0005903 | brush border | *eps-8* | 0.108 |
| GO:0030904 | retromer complex | *vps-26* | 0.108 |
| GO:0000299 | integral to membrane of membrane fraction | *unc-84* | 0.108 |
| GO:0048471 | perinuclear region of cytoplasm | *tra-2; ogt-1* | 0.109 |
| GO:0030118 | clathrin coat | *unc-101; dab-1* | 0.11 |
| GO:0044433 | cytoplasmic vesicle part | *dab-1; f45g2.4* | 0.11 |
| GO:0044424 | intracellular part | *bmk-1; sel-8; y48a6b.3; klp-4; crn-1; y51h1a.3; tag-203; mup-2; col-14; dab-1; ddp-1; rpl-22; pme-2; ooc-3; bli-1; tra-2; anc-1; col-84; cpr-1; asf-1; tba-6; c49h3.4; tct-1; eft-3; vhp-1; pro-1; e02a10.1; pek-1; nhr-62; aps-2; cap-2; y106g6h.1; ogt-1; rpl-5; lin-41; his-37; ccf-1; unc-84; f43g9.12; unc-101; f45g2.4; f22b3.4; trap-4; oma-1* | 0.11 |
| GO:0012506 | vesicle membrane | *dab-1; f45g2.4* | 0.11 |
| GO:0030121 | AP-1 adaptor complex | *dab-1* | 0.11 |
| GO:0005652 | nuclear lamina | *unc-84* | 0.11 |
| GO:0030669 | clathrin-coated endocytic vesicle membrane | *dab-1* | 0.11 |
| GO:0030128 | clathrin coat of endocytic vesicle | *dab-1* | 0.11 |
| GO:0005638 | lamin filament | *unc-84* | 0.11 |
| GO:0045334 | clathrin-coated endocytic vesicle | *dab-1* | 0.11 |
| GO:0030122 | AP-2 adaptor complex | *dab-1* | 0.11 |
| GO:0008290 | F-actin capping protein complex | *cap-2* | 0.11 |
| GO:0030666 | endocytic vesicle membrane | *dab-1* | 0.11 |
| GO:0042734 | presynaptic membrane | *syd-1* | 0.11 |
| GO:0005783 | endoplasmic reticulum | *trap-4; ooc-3; pek-1; y106g6h.1* | 0.115 |
| GO:0015630 | microtubule cytoskeleton | *bmk-1; oma-1; tba-6; klp-4* | 0.139 |
| GO:0012510 | trans-Golgi network transport vesicle membrane | *dab-1* | 0.139 |
| GO:0005637 | nuclear inner membrane | *unc-84* | 0.139 |
| GO:0030130 | clathrin coat of trans-Golgi network vesicle | *dab-1* | 0.139 |
| GO:0005622 | intracellular | *bmk-1; sel-8; y48a6b.3; klp-4; crn-1; y51h1a.3; y46g5a.1; tag-203; col-14; mup-2; dab-1; ddp-1; f44f1.3; rpl-22; pme-2; bli-1; k09c4.5; ooc-3; tra-2; anc-1; col-84; cpr-1; asf-1; tba-6; c49h3.4; tct-1; eft-3; vhp-1; syd-1; pro-1; e02a10.1; pek-1; b0035.1; aps-2; nhr-62; ogt-1; cap-2; y106g6h.1; rpl-5; lin-41; his-37; ccf-1; unc-84; f43g9.12; e01g4.1; rrc-1; unc-101; f45g2.4; f22b3.4; trap-4; oma-1* | 0.139 |
| GO:0012505 | endomembrane system | *vps-26; dab-1; f45g2.4; unc-84* | 0.141 |
| GO:0032991 | macromolecular complex | *c49h3.4; bmk-1; unc-101; f14f11.1; rpl-22; vps-26; e02a10.1; y48a6b.3; klp-4; cap-2; rpl-5; his-37; mup-2; tba-6; dab-1; ddp-1* | 0.144 |
| GO:0030125 | clathrin vesicle coat | *dab-1* | 0.144 |
| GO:0030137 | COPI-coated vesicle | *f45g2.4* | 0.144 |
| GO:0005865 | striated muscle thin filament | *mup-2* | 0.144 |
| GO:0030126 | COPI vesicle coat | *f45g2.4* | 0.144 |
| GO:0030139 | endocytic vesicle | *dab-1* | 0.144 |
| GO:0030663 | COPI coated vesicle membrane | *f45g2.4* | 0.144 |
| GO:0030132 | clathrin coat of coated pit | *dab-1* | 0.144 |
| GO:0030665 | clathrin coated vesicle membrane | *dab-1* | 0.144 |
| GO:0030135 | coated vesicle | *dab-1; f45g2.4* | 0.161 |
| GO:0042719 | mitochondrial intermembrane space protein transporter complex | *ddp-1* | 0.161 |
| GO:0005905 | coated pit | *dab-1* | 0.161 |
| GO:0051233 | spindle midzone | *bmk-1* | 0.161 |
| GO:0030140 | trans-Golgi network transport vesicle | *dab-1* | 0.161 |
| GO:0005875 | microtubule associated complex | *bmk-1; klp-4* | 0.164 |
| GO:0005758 | mitochondrial intermembrane space | *ddp-1* | 0.182 |
| GO:0031970 | organelle envelope lumen | *ddp-1* | 0.182 |
| GO:0044431 | Golgi apparatus part | *dab-1; f45g2.4* | 0.198 |
| GO:0030660 | Golgi-associated vesicle membrane | *dab-1* | 0.198 |
| GO:0030658 | transport vesicle membrane | *dab-1* | 0.198 |
| GO:0016023 | cytoplasmic membrane-bound vesicle | *dab-1; f45g2.4* | 0.198 |
| GO:0030529 | ribonucleoprotein complex | *c49h3.4; rpl-5; rpl-22; e02a10.1; y48a6b.3* | 0.227 |
| GO:0031988 | membrane-bound vesicle | *dab-1; f45g2.4* | 0.227 |
| GO:0031410 | cytoplasmic vesicle | *dab-1; f45g2.4* | 0.23 |
| GO:0030133 | transport vesicle | *dab-1* | 0.23 |
| GO:0005882 | intermediate filament | *unc-84* | 0.23 |
| GO:0015629 | actin cytoskeleton | *mup-2; cap-2* | 0.242 |
| GO:0000922 | spindle pole | *bmk-1* | 0.242 |
| GO:0045111 | intermediate filament cytoskeleton | *unc-84* | 0.242 |
| GO:0031982 | vesicle | *dab-1; f45g2.4* | 0.246 |
| GO:0044446 | intracellular organelle part | *bmk-1; pro-1; f45g2.4; klp-4; cap-2; oma-1; mup-2; his-37; tba-6; dab-1; unc-84; ddp-1* | 0.291 |
| GO:0005794 | Golgi apparatus | *dab-1; f45g2.4* | 0.298 |
| GO:0044422 | organelle part | *bmk-1; pro-1; f45g2.4; klp-4; cap-2; oma-1; mup-2; his-37; tba-6; dab-1; unc-84; ddp-1* | 0.306 |
| GO:0043229 | intracellular organelle | *bmk-1; sel-8; vhp-1; e02a10.1; pro-1; pek-1; klp-4; crn-1; nhr-62; ogt-1; cap-2; y106g6h.1; y51h1a.3; rpl-5; tag-203; his-37; mup-2; dab-1; ccf-1; f43g9.12; unc-84; ddp-1; pme-2; rpl-22; ooc-3; f45g2.4; trap-4; oma-1; cpr-1; tba-6; asf-1* | 0.33 |
| GO:0043226 | organelle | *bmk-1; sel-8; vhp-1; e02a10.1; pro-1; pek-1; klp-4; crn-1; nhr-62; ogt-1; cap-2; y106g6h.1; y51h1a.3; rpl-5; tag-203; his-37; mup-2; dab-1; ccf-1; f43g9.12; unc-84; ddp-1; pme-2; rpl-22; ooc-3; f45g2.4; trap-4; oma-1; cpr-1; tba-6; asf-1* | 0.343 |
| GO:0030017 | sarcomere | *mup-2* | 0.352 |
| GO:0043232 | intracellular non-membrane-bound organelle | *bmk-1; rpl-22; e02a10.1; klp-4; cap-2; rpl-5; oma-1; his-37; mup-2; tba-6; unc-84* | 0.352 |
| GO:0043228 | non-membrane-bound organelle | *bmk-1; rpl-22; e02a10.1; klp-4; cap-2; rpl-5; oma-1; his-37; mup-2; tba-6; unc-84* | 0.352 |
| GO:0043234 | protein complex | *bmk-1; unc-101; f14f11.1; vps-26; klp-4; cap-2; mup-2; his-37; dab-1; tba-6; ddp-1* | 0.352 |
| GO:0030016 | myofibril | *mup-2* | 0.352 |
| GO:0005624 | membrane fraction | *unc-84* | 0.352 |
| GO:0044449 | contractile fiber part | *mup-2* | 0.364 |
| GO:0044453 | nuclear membrane part | *unc-84* | 0.409 |
| GO:0030136 | clathrin-coated vesicle | *dab-1* | 0.409 |
| GO:0031965 | nuclear membrane | *unc-84* | 0.409 |
| GO:0043292 | contractile fiber | *mup-2* | 0.409 |
| GO:0045495 | pole plasm | *oma-1* | 0.41 |
| GO:0005813 | centrosome | *oma-1* | 0.41 |
| GO:0043186 | P granule | *oma-1* | 0.41 |
| GO:0005815 | microtubule organizing center | *oma-1* | 0.41 |
| GO:0000139 | Golgi membrane | *dab-1* | 0.41 |
| GO:0000267 | cell fraction | *unc-84* | 0.41 |
| GO:0014704 | intercalated disc | *inx-21* | 0.428 |
| GO:0005921 | gap junction | *inx-21* | 0.428 |
| GO:0031967 | organelle envelope | *unc-84; ddp-1* | 0.442 |
| GO:0005739 | mitochondrion | *ddp-1; y51h1a.3* | 0.461 |
| GO:0005874 | microtubule | *tba-6* | 0.461 |
| GO:0031975 | envelope | *unc-84; ddp-1* | 0.461 |
| GO:0005819 | spindle | *bmk-1* | 0.466 |
| GO:0044459 | plasma membrane part | *f14f11.1; dab-1; inx-21; tra-2* | 0.484 |
| GO:0005635 | nuclear envelope | *unc-84* | 0.491 |
| GO:0043233 | organelle lumen | *pro-1; ddp-1* | 0.492 |
| GO:0019866 | organelle inner membrane | *unc-84* | 0.492 |
| GO:0005887 | integral to plasma membrane | *f14f11.1; tra-2* | 0.501 |
| GO:0031226 | intrinsic to plasma membrane | *f14f11.1; tra-2* | 0.502 |
| GO:0005886 | plasma membrane | *f14f11.1; dab-1; inx-21; tra-2; aqp-4* | 0.507 |
| GO:0005578 | proteinaceous extracellular matrix | *bli-1* | 0.517 |
| GO:0031090 | organelle membrane | *dab-1; f45g2.4; unc-84* | 0.525 |
| GO:0005840 | ribosome | *rpl-5; rpl-22; e02a10.1* | 0.525 |
| GO:0044464 | cell part | *sel-8; fat-3; mig-1; y48a6b.3; klp-4; f31e8.4; y46g5a.1; col-14; y54g9a.4; mup-2; eps-8; rpl-22; k09c4.5; anc-1; tra-2; c18e9.2; asf-1; tba-6; c49h3.4; f14f11.1; eft-3; e02a10.1; pek-1; b0035.1; sre-14; aps-2; y106g6h.1; cap-2; ogt-1; elo-1; c24g7.1; his-37; ccf-1; f43g9.12; e01g4.1; unc-101; f45g2.4; f22b3.4; f55g1.12; trap-4; oma-1; bmk-1; vps-26; crn-1; y51h1a.3; tag-203; dab-1; ddp-1; f44f1.3; pme-2; bli-1; ooc-3; r11f4.2; col-84; cpr-1; inx-21; aqp-4; tct-1; syd-1; vhp-1; pro-1; nhr-62; lin-41; rpl-5; unc-84; rrc-1; tsp-5; t23b3.2; aqp-2; elo-7; w05h12.1; gly-19* | 0.525 |
| GO:0044421 | extracellular region part | *bli-1* | 0.525 |
| GO:0005911 | intercellular junction | *inx-21* | 0.525 |
| GO:0005740 | mitochondrial envelope | *ddp-1* | 0.569 |
| GO:0008076 | voltage-gated potassium channel complex | *f14f11.1* | 0.61 |
| GO:0005654 | nucleoplasm | *pro-1* | 0.618 |
| GO:0044429 | mitochondrial part | *ddp-1* | 0.633 |
| GO:0030054 | cell junction | *inx-21* | 0.634 |
| GO:0043231 | intracellular membrane-bound organelle | *sel-8; vhp-1; pro-1; pek-1; crn-1; nhr-62; ogt-1; y106g6h.1; y51h1a.3; tag-203; his-37; dab-1; ccf-1; f43g9.12; unc-84; ddp-1; pme-2; ooc-3; f45g2.4; trap-4; cpr-1; asf-1* | 0.738 |
| GO:0043227 | membrane-bound organelle | *sel-8; vhp-1; pro-1; pek-1; crn-1; nhr-62; ogt-1; y106g6h.1; y51h1a.3; tag-203; his-37; dab-1; ccf-1; f43g9.12; unc-84; ddp-1; pme-2; ooc-3; f45g2.4; trap-4; cpr-1; asf-1* | 0.75 |
| GO:0044425 | membrane part | *f14f11.1; vps-26; sre-14; aps-2; elo-1; f31e8.4; dab-1; unc-84; unc-101; tsp-5; k09c4.5; r11f4.2; t23b3.2; anc-1; tra-2; f45g2.4; aqp-2; trap-4; c18e9.2; elo-7; inx-21* | 1 |
