# Supplementary material for: Large-scale functional RNAi screen in C. elegans identifies genes that regulate the dysfunction of mutant polyglutamine neurons
Source: BMC Genomics. 2012 Mar 13;13:91. doi: 10.1186/1471-2164-13-91 (PMC3331833; doi:10.1186/1471-2164-13-91)
Supplement: Additional file 9 — Table S8. Modules (n = 137) generated by network-boosted analysis for suppression of 128Q-neuron dysfunction by RNAi. [file 1471-2164-13-91-S9.DOC]

**Supplementary Table 8.** Modules (n = 137) generated by network-boosted analysis for suppression of 128-neuron dysfunction by RNAi.

N indicates the number of genes *per* module. See database at http://www.broca.inserm.fr/EHDN2/RNAiscreen to vizualize the modules and their content.

| **Module ID** | **N** | **Best GO term** | **P value** | **Best pathway term** | **P value** | **Genes of interest** | **P value** |
| --- | --- | --- | --- | --- | --- | --- | --- |
| W_S_16 | 47 | cell body | 5.2e-07 | Oxidative phosphorylation | 0.0002 | synapse | 0.0012 |
| W_core_S_9 | 19 | response to starvation | 0.01060 | p53 pathway by glucose deprivation | 0.0057 |  |  |
| W_S_20 | 16 | nucleotide-sugar metabolic process | 0.00258 | Mannose metabolism | 0.0084 |  |  |
| W_core_S_13 | 15 | nucleoside metabolic process | 0.00049 | DNA replication | 0.0003 |  |  |
| W_core_S_15 | 15 | establishment of organelle localization | 2.4e-06 | Glycosphingolipid biosynthesis - ganglio series | 0.0089 | Htt_partner | 0.0048 |
| W_S_15 | 15 | cell surface | 0.00089 | Aminobutyrate degradation | 0.0293 | synapse | 0.0104 |
| W_core_S_1 | 14 | transferase activity, transferring one-carbon groups | 0.00063 |  |  |  |  |
| W_core_S_18 | 13 | integral to organelle membrane | 0.00239 | Glycosaminoglycan degradation | 0.0078 |  |  |
| W_core_S_2 | 12 | pole plasm | 0.00420 | Vitamin B6 metabolism | 0.0166 |  |  |
| W_core_S_26 | 11 | nucleobase metabolic process | 0.00014 | Starch and sucrose metabolism | 0.0006 |  |  |
| W_core_S_31 | 10 | autophagy | 0.00043 | Starch and sucrose metabolism | 0.0005 | autophagy | 0.0045 |
| W_S_2 | 10 | amine binding | 0.02226 | Cell cycle | 0.0092 |  |  |
| W_S_54 | 10 | glucuronosyltransferase activity | 0.03783 |  |  |  |  |
| W_S_58 | 10 | glucuronosyltransferase activity | 0.03783 |  |  |  |  |
| W_S_56 | 9 |  |  |  |  |  |  |
| W_core_S_36 | 8 | oligosaccharide metabolic process | 0.00074 | Starch and sucrose metabolism | 0.0003 | autophagy | 0.0310 |
| W_core_S_46 | 8 | reactive oxygen species metabolic process | 1.5e-08 | Cholesterol biosynthesis | 0.0095 |  |  |
| W_core_S_40 | 8 | response to xenobiotic stimulus | 0.00268 | Starch and sucrose metabolism | 0.0268 | mitochondria | 0.0353 |
| W_core_S_56 | 8 | cell body | 0.00314 | Interleukin signaling pathway | 0.0331 |  |  |
| W_core_S_67 | 8 | envelope | 1.7e-07 |  |  | mitochondria | 0.0056 |
| W_core_S_6 | 7 | cellular aromatic compound metabolic process | 3.5e-07 | Phenylalanine metabolism | 0.0000 | mitochondria | 0.0204 |
| W_core_S_42 | 7 | extracellular region part | 5.9e-12 | Integrin signaling pathway | 0.0021 |  |  |
| W_core_S_19 | 7 | cell recognition | 0.00138 | Vasopressin synthesis | 0.0048 |  |  |
| W_S_11 | 7 | dosage compensation complex | 0.00147 | Oxytocin receptor mediated signaling pathway | 0.0229 |  |  |
| W_core_S_38 | 7 | outer membrane | 6.0e-09 |  |  | mitochondria | 0.0024 |
| W_S_33 | 7 |  |  |  |  |  |  |
| W_core_S_75 | 6 | O-methyltransferase activity | 4.3e-05 | Metabolism of xenobiotics by cytochrome P450 | 0.0007 |  |  |
| W_core_S_8 | 6 | multicellular organismal movement | 0.00040 | Valine biosynthesis | 0.0048 |  |  |
| W_core_S_60 | 6 |  |  |  |  |  |  |
| W_core_S_4 | 5 | envelope | 0.00011 | Oxidative phosphorylation | 0.0000 | mitochondria | 0.0397 |
| W_core_S_47 | 5 | protein dimerization activity | 0.00098 | Ornithine degradation | 0.0001 |  |  |
| W_core_S_14 | 5 | peptide biosynthetic process | 0.02650 | Valine, leucine and isoleucine degradation | 0.0008 |  |  |
| W_S_17 | 5 |  |  | Angiogenesis | 0.0043 | mitochondria | 0.0480 |
| W_S_29 | 5 | aster | 0.00523 | Ornithine degradation | 0.0072 | mitochondria | 0.0480 |
| W_core_S_10 | 5 | ferric iron binding | 6.0e-05 | Porphyrin and chlorophyll metabolism | 0.0099 |  |  |
| W_core_S_37 | 5 | germ-line sex determination | 0.00028 | Cytoskeletal regulation by Rho GTPase | 0.0345 |  |  |
| W_core_S_53 | 5 | basal part of cell | 2.2e-07 | Integrin signaling pathway | 0.0499 |  |  |
| W_core_S_57 | 5 | extracellular region part | 4.9e-10 | Integrin signaling pathway | 0.0499 |  |  |
| W_S_30 | 5 | positive regulation of lipid storage | 0.00035 |  |  | Htt_partner | 0.0457 |
| W_S_44 | 5 | glycoprotein metabolic process | 0.02866 |  |  |  |  |
| W_S_50 | 5 |  |  |  |  |  |  |
| W_core_S_41 | 5 | establishment or maintenance of transmembrane electrochemical gradient | 0.00197 |  |  |  |  |
| W_S_48 | 4 | inositol or phosphatidylinositol phosphatase activity | 0.04761 | Mucin type O-Glycan biosynthesis | 0.0000 |  |  |
| W_core_S_62 | 4 | tail morphogenesis | 0.00022 | Sulfate assimilation | 0.0003 |  |  |
| W_core_S_27 | 4 | cyclin-dependent protein kinase holoenzyme complex | 0.00108 | Vasopressin synthesis | 0.0014 |  |  |
| W_core_S_16 | 4 | multicellular organismal signaling | 0.01390 | 5-Hydroxytryptamine biosynthesis | 0.0032 |  |  |
| W_core_S_59 | 4 | extracellular region part | 6.3e-05 | Riboflavin metabolism | 0.0032 |  |  |
| W_core_S_63 | 4 | transferase activity, transferring nitrogenous groups | 0.00037 | Parkinson disease | 0.0032 |  |  |
| W_core_S_77 | 4 |  |  | Riboflavin metabolism | 0.0032 |  |  |
| W_S_31 | 4 | sex determination | 3.2e-05 | Insulin_IGF pathway-protein kinase B signaling cascade | 0.0063 | mitochondria | 0.0016 |
| W_S_32 | 4 | establishment of organelle localization | 0.02743 |  |  | synapse | 0.0477 |
| W_S_27 | 4 | digestion | 0.00081 |  |  |  |  |
| W_S_35 | 4 |  |  |  |  |  |  |
| W_S_37 | 4 |  |  |  |  |  |  |
| W_S_45 | 4 | ion channel complex | 0.00845 |  |  |  |  |
| W_S_49 | 4 |  |  |  |  |  |  |
| W_S_5 | 4 |  |  |  |  |  |  |
| W_S_51 | 4 | digestion | 0.00081 |  |  |  |  |
| W_S_52 | 4 | protein glycosylation | 0.04615 |  |  |  |  |
| W_core_S_20 | 4 | positive regulation of lipid storage | 0.00070 |  |  |  |  |
| W_core_S_24 | 4 | regulation of cell death | 0.00827 |  |  |  |  |
| W_core_S_34 | 4 | ubiquitin ligase complex | 0.00042 |  |  |  |  |
| W_core_S_35 | 4 | sex determination | 0.00012 |  |  |  |  |
| W_core_S_39 | 4 |  |  |  |  |  |  |
| W_core_S_43 | 4 | cell surface | 0.00044 |  |  |  |  |
| W_core_S_48 | 4 | cell surface | 0.00033 |  |  |  |  |
| W_core_S_49 | 4 | synapse organization | 0.00499 |  |  |  |  |
| W_core_S_5 | 4 |  |  |  |  |  |  |
| W_core_S_55 | 4 |  |  |  |  |  |  |
| W_core_S_65 | 4 |  |  |  |  |  |  |
| W_core_S_7 | 4 | cell junction organization | 0.00033 |  |  |  |  |
| W_core_S_76 | 4 | muscle structure development | 3.7e-08 |  |  |  |  |
| W_S_7 | 3 | neurotransmitter metabolic process | 1.9e-07 | mRNA splicing | 0.0000 |  |  |
| W_core_S_30 | 3 | DNA-directed RNA polymerase activity | 0.03801 | Pyrimidine metabolism | 0.0005 |  |  |
| W_S_22 | 3 | phosphotransferase activity, for other substituted phosphate groups | 0.01121 | Pantothenate and CoA biosynthesis | 0.0024 |  |  |
| W_S_9 | 3 | carbohydrate binding | 0.01776 | Other types of O-glycan biosynthesis | 0.0028 |  |  |
| W_S_19 | 3 | receptor binding | 0.00169 | Cadherin signaling pathway | 0.0051 |  |  |
| W_core_S_17 | 3 | protein serine/threonine phosphatase complex | 0.00269 | FGF signaling pathway | 0.0226 |  |  |
| W_core_S_58 | 3 | establishment of organelle localization | 0.02743 | Pyrimidine metabolism | 0.0378 |  |  |
| W_core_S_72 | 3 | GABA receptor activity | 0.00014 |  |  | synapse | 0.0108 |
| W_S_47 | 3 |  |  |  |  | Htt_partner | 0.0277 |
| W_S_36 | 3 | nucleic acid phosphodiester bond hydrolysis | 0.00768 |  |  | autophagy | 0.0468 |
| W_S_18 | 3 |  |  |  |  |  |  |
| W_S_21 | 3 | establishment of organelle localization | 0.02743 |  |  |  |  |
| W_S_24 | 3 |  |  |  |  |  |  |
| W_S_28 | 3 | lipid binding | 0.01440 |  |  |  |  |
| W_S_39 | 3 |  |  |  |  |  |  |
| W_S_43 | 3 | muscle structure development | 0.00394 |  |  |  |  |
| W_core_S_3 | 3 | digestive tract morphogenesis | 0.00090 |  |  |  |  |
| W_core_S_33 | 3 | tail morphogenesis | 7.4e-05 |  |  |  |  |
| W_core_S_44 | 3 | apical part of cell | 0.00134 |  |  |  |  |
| W_core_S_45 | 3 | positive regulation of metabolic process | 0.00900 |  |  |  |  |
| W_core_S_50 | 3 |  |  |  |  |  |  |
| W_core_S_52 | 3 | muscle structure development | 0.00394 |  |  |  |  |
| W_core_S_61 | 3 | metal cluster binding | 0.00598 |  |  |  |  |
| W_core_S_64 | 3 | cognition | 0.00625 |  |  |  |  |
| W_core_S_70 | 3 | protein folding | 0.00024 |  |  |  |  |
| W_core_S_29 | 2 | positive regulation of transport | 0.00055 | Salvage pyrimidine ribonucleotides | 0.0000 | synapse | 0.0488 |
| W_core_S_11 | 2 | cell body | 0.00078 | Ornithine degradation | 0.0000 | autophagy | 0.0013 |
| W_core_S_28 | 2 | cellular homeostasis | 0.00983 | Valine, leucine and isoleucine degradation | 0.0001 | mitochondria | 0.0302 |
| W_S_26 | 2 |  |  | Carnitine metabolism | 0.0003 | mitochondria | 0.0056 |
| W_core_S_74 | 2 | cell projection part | 0.00067 | Coenzyme A biosynthesis | 0.0012 |  |  |
| W_S_25 | 2 |  |  | Ionotropic glutamate receptor pathway | 0.0029 |  |  |
| W_S_6 | 2 | cell body | 0.00157 | mRNA splicing | 0.0034 |  |  |
| W_S_42 | 2 | GABA receptor activity | 0.01355 | N-Glycan biosynthesis | 0.0048 |  |  |
| W_S_4 | 2 | ER-nucleus signaling pathway | 0.00505 | Cytoskeletal regulation by Rho GTPase | 0.0057 | autophagy | 0.0315 |
| W_core_S_12 | 2 | cell body | 0.00157 | Heterotrimeric G-protein signaling pathway-Gq alpha and Go alpha mediated pathway | 0.0084 | synapse | 0.0488 |
| W_S_38 | 2 | secretion by cell | 0.03094 | Pyrimidine metabolism | 0.0088 |  |  |
| W_core_S_21 | 2 | dauer larval development | 0.00924 | TGF-beta signaling pathway | 0.0092 | Htt_partner | 0.0466 |
| W_core_S_51 | 2 | neuromuscular junction | 0.01941 | Arginine biosynthesis | 0.0100 | Htt_partner | 0.0466 |
| W_S_13 | 2 | directional locomotion | 0.00235 | Oxidative phosphorylation | 0.0128 |  |  |
| W_core_S_25 | 2 | multicellular organismal metabolic process | 0.00024 | Oxytocin receptor mediated signaling pathway | 0.0175 |  |  |
| W_S_3 | 2 |  |  |  |  | Htt_partner | 0.0185 |
| W_S_34 | 2 | ubiquitin ligase complex | 7.0e-05 |  |  | Htt_partner | 0.0185 |
| W_S_1 | 2 |  |  |  |  |  |  |
| W_S_10 | 2 |  |  |  |  |  |  |
| W_S_12 | 2 |  |  |  |  |  |  |
| W_S_14 | 2 | metallochaperone activity | 0.00048 |  |  |  |  |
| W_S_23 | 2 | dormancy process | 0.00243 |  |  |  |  |
| W_S_40 | 2 | tail morphogenesis | 0.00909 |  |  |  |  |
| W_S_41 | 2 | mesoderm development | 0.00241 |  |  |  |  |
| W_S_46 | 2 |  |  |  |  |  |  |
| W_S_53 | 2 |  |  |  |  |  |  |
| W_S_55 | 2 | pattern specification process | 0.00155 |  |  |  |  |
| W_S_57 | 2 |  |  |  |  |  |  |
| W_S_59 | 2 |  |  |  |  |  |  |
| W_S_60 | 2 |  |  |  |  |  |  |
| W_S_8 | 2 | RNA catabolic process | 0.00528 |  |  |  |  |
| W_core_S_22 | 2 |  |  |  |  |  |  |
| W_core_S_23 | 2 | membrane-enclosed lumen | 6.6e-05 |  |  |  |  |
| W_core_S_32 | 2 | response to abiotic stimulus | 0.00071 |  |  |  |  |
| W_core_S_54 | 2 | phosphatase activator activity | 0.00434 |  |  |  |  |
| W_core_S_66 | 2 | cell projection part | 0.00067 |  |  |  |  |
| W_core_S_68 | 2 |  |  |  |  |  |  |
| W_core_S_69 | 2 | regulation of synapse organization | 0.00058 |  |  |  |  |
| W_core_S_71 | 2 |  |  |  |  |  |  |
| W_core_S_73 | 2 | spliceosomal complex | 0.02024 |  |  |  |  |
