# Supplementary material for: Large-scale functional RNAi screen in C. elegans identifies genes that regulate the dysfunction of mutant polyglutamine neurons
Source: BMC Genomics. 2012 Mar 13;13:91. doi: 10.1186/1471-2164-13-91 (PMC3331833; doi:10.1186/1471-2164-13-91)
Supplement: Additional file 10 — Table S9. Modules (n = 105) generated by network-boosted analysis for aggravation of 128Q-neuron dysfunction by RNAi. [file 1471-2164-13-91-S10.DOC]

**Supplementary Table 9.** Modules (n = 105) generated by network-boosted analysis for aggravation of 128-neuron dysfunction by RNAi.

N indicates the number of genes per module. See database at http://www.broca.inserm.fr/EHDN2/RNAiscreen to vizualize the modules and their content.

| **Module ID** | **N** | **Best GO term** | **P value** | **Best pathway term** | **P value** | **Genes of interest** | **P value** |
| --- | --- | --- | --- | --- | --- | --- | --- |
| W_core_E_4 | 50 | generation of precursor metabolites and energy | 2.15E-06 | Pentose phosphate pathway | 3.37E-05 | mitochondria | 0.001265 |
| W_E_25 | 37 | somatic sex determination | 1.60E-07 | Apoptosis signaling pathway | 0.004587 | synapse | 2.065e-05 |
| W_core_E_24 | 32 | cell projection | 1.00E-05 | Nicotinic acetylcholine receptor signaling pathway | 0.002289 | synapse | 0.01422 |
| W_E_36 | 29 | somatic sex determination | 1.30E-07 | Histamine H1 receptor mediated signaling pathway | 0.0302 |  |  |
| W_E_2 | 21 |  |  |  |  |  |  |
| W_core_E_1 | 19 | proteasome regulatory particle | 1.65E-05 | Glycolysis | 2.05E-05 | mitochondria | 0.03449 |
| W_E_14 | 19 |  |  |  |  |  |  |
| W_E_29 | 14 | regulation of neurological system process | 5.77E-05 | Salvage pyrimidine ribonucleotides | 0.01104 |  |  |
| W_E_3 | 12 | mitochondrion | 0.000382 | Oxidative phosphorylation | 6.19E-11 | mitochondria | 3.675e-07 |
| W_core_E_2 | 11 | peptide metabolic process | 0.001108 | Oxidative phosphorylation | 3.91E-07 | mitochondria | 0.005703 |
| W_core_E_6 | 11 | DNA replication, synthesis of RNA primer | 4.84E-05 | De novo purine biosynthesis | 1.03E-05 |  |  |
| W_E_24 | 11 |  |  |  |  |  |  |
| W_E_21 | 10 | secretion by cell | 0.004798 | Salvage pyrimidine ribonucleotides | 0.007898 | synapse | 0.01076 |
| W_E_28 | 10 | nuclear periphery | 0.000273 |  |  |  |  |
| W_core_E_32 | 10 | somatic sex determination | 2.40E-08 |  |  |  |  |
| W_core_E_22 | 8 | peptide metabolic process | 0.000188 | Linoleic acid metabolism | 4.00E-05 |  |  |
| W_core_E_3 | 8 | regulation of development, heterochronic | 1.90E-07 |  |  |  |  |
| W_core_E_28 | 7 | isoprenoid biosynthetic process | 0.001122 | Terpenoid backbone biosynthesis | 5.33E-05 | mitochondria | 0.0001636 |
| W_E_27 | 7 | lipoprotein metabolic process | 0.006578 | Glycosylphosphatidylinositol(GPI)-anchor biosynthesis | 0.004615 |  |  |
| W_E_12 | 7 |  |  |  |  |  |  |
| W_E_18 | 7 |  |  |  |  |  |  |
| W_core_E_31 | 7 | regulation of development, heterochronic | 1.49E-05 |  |  |  |  |
| W_E_39 | 6 | immune system process | 0.002335 | Tetrahydrofolate biosynthesis | 9.41E-06 |  |  |
| W_core_E_33 | 6 | isoprenoid biosynthetic process | 0.001122 | Terpenoid backbone biosynthesis | 3.81E-05 | mitochondria | 0.01013 |
| W_E_19 | 6 | chromatin binding | 0.007840 | Thiamin metabolism | 0.000396 |  |  |
| W_E_22 | 6 | N-terminal protein amino acid modification | 0.002642 |  |  |  |  |
| W_core_E_15 | 6 | organic acid biosynthetic process | 5.82E-05 |  |  |  |  |
| W_core_E_18 | 6 | exopeptidase activity | 4.16E-07 |  |  |  |  |
| W_core_E_51 | 6 | regulation of development, heterochronic | 0.000786 |  |  |  |  |
| W_core_E_10 | 5 | cellular polysaccharide metabolic process | 9.35E-10 | Heterotrimeric G-protein signaling pathway-Gi alpha and Gs alpha mediated pathway | 1.55E-06 | druggable | 0.04101 |
| W_core_E_30 | 5 | organ growth | 6.05E-07 | 5HT1 type receptor mediated signaling pathway | 0.002991 | druggable | 0.04101 |
| W_E_26 | 5 | clathrin binding | 0.001218 | Cadherin signaling pathway | 0.008558 | autophagy | 0.002434 |
| W_core_E_25 | 5 | microtubule | 4.43E-06 | Cytoskeletal regulation by Rho GTPase | 0.03445 |  |  |
| W_E_10 | 5 | pattern specification process | 0.001551 |  |  |  |  |
| W_E_38 | 5 |  |  |  |  |  |  |
| W_E_47 | 5 |  |  |  |  |  |  |
| W_E_5 | 5 |  |  |  |  |  |  |
| W_E_7 | 5 |  |  |  |  |  |  |
| W_core_E_9 | 5 | gonad morphogenesis | 5.93E-08 |  |  |  |  |
| W_core_E_29 | 4 | regulation of cellular response to stress | 0.000547 | Ornithine degradation | 6.10E-05 | druggable | 0.02577 |
| W_core_E_39 | 4 | phosphorylase kinase complex | 0.002617 | Starch and sucrose metabolism | 6.88E-05 | druggable | 0.02577 |
| W_core_E_36 | 4 | cell leading edge | 0.000674 | Ether lipid metabolism | 0.004783 |  |  |
| W_E_42 | 4 | presynaptic membrane | 0.011111 | Nitrogen metabolism | 0.005537 |  |  |
| W_E_11 | 4 | regulation of cell projection organization | 0.003357 | Glycerophospholipid metabolism | 0.01184 |  |  |
| W_core_E_38 | 4 | organophosphate metabolic process | 0.012992 | Inositol phosphate metabolism | 0.0143 |  |  |
| W_E_1 | 4 |  |  |  |  |  |  |
| W_E_17 | 4 |  |  |  |  |  |  |
| W_E_52 | 4 |  |  |  |  |  |  |
| W_E_9 | 4 | peptide metabolic process | 0.000188 |  |  |  |  |
| W_core_E_14 | 4 | cell fraction | 0.000449 |  |  |  |  |
| W_core_E_20 | 4 | nuclear outer membrane-endoplasmic reticulum membrane network | 0.004181 |  |  |  |  |
| W_core_E_49 | 4 | endoplasmic reticulum | 9.68E-05 |  |  |  |  |
| W_E_35 | 3 |  |  | Mucin type O-Glycan biosynthesis | 6.41E-07 |  |  |
| W_core_E_26 | 3 | pattern specification process | 0.003101 | Fatty acid biosynthesis | 0.001197 |  |  |
| W_E_23 | 3 | organophosphate metabolic process | 0.012992 | Linoleic acid metabolism | 0.001387 |  |  |
| W_E_31 | 3 | regulation of cell differentiation | 0.004682 | Nicotinic acetylcholine receptor signaling pathway | 0.002772 |  |  |
| W_core_E_45 | 3 |  |  | Adrenaline and noradrenaline biosynthesis | 0.005381 |  |  |
| W_core_E_40 | 3 | membrane coat | 5.21E-08 | Huntington disease | 0.03551 | autophagy | 4.564e-05 |
| W_core_E_44 | 3 | spindle | 0.000384 | Huntington disease | 0.03551 | synapse | 0.01079 |
| W_E_13 | 3 |  |  |  |  |  |  |
| W_E_15 | 3 | phagocytosis, engulfment | 0.010322 |  |  |  |  |
| W_E_16 | 3 | spindle | 0.000384 |  |  |  |  |
| W_E_44 | 3 |  |  |  |  |  |  |
| W_E_46 | 3 |  |  |  |  |  |  |
| W_E_48 | 3 |  |  |  |  |  |  |
| W_E_51 | 3 | synapse part | 0.032527 |  |  |  |  |
| W_core_E_11 | 3 | anatomical structure formation involved in morphogenesis | 0.003101 |  |  |  |  |
| W_core_E_12 | 3 | organic acid biosynthetic process | 5.82E-05 |  |  |  |  |
| W_core_E_19 | 3 | copper ion transmembrane transport | 1.02E-05 |  |  |  |  |
| W_core_E_35 | 3 | organic acid biosynthetic process | 0.039419 |  |  |  |  |
| W_core_E_43 | 3 | AP-type membrane coat adaptor complex | 1.38E-06 |  |  |  |  |
| W_core_E_48 | 3 | ion channel complex | 5.75E-07 |  |  |  |  |
| W_core_E_7 | 3 | oocyte differentiation | 7.89E-08 |  |  |  |  |
| W_core_E_23 | 3 | regulation of cell adhesion | 0.001725 |  |  | synapse | 0.01079 |
| W_core_E_5 | 3 | retromer complex | 0.000143 |  |  | synapse | 0.01079 |
| W_E_41 | 3 | cell fraction | 0.001347 |  |  | autophagy | 0.04681 |
| W_E_37 | 2 | protein maturation | 0.003375 | Nicotinic acetylcholine receptor signaling pathway | 0.001849 |  |  |
| W_core_E_42 | 2 | positive regulation of transferase activity | 0.009515 | Sphingolipid metabolism | 0.002793 |  |  |
| W_E_40 | 2 | positive regulation of molecular function | 0.006576 | Heterotrimeric G-protein signaling pathway-Gq alpha and Go alpha mediated pathway | 0.003564 |  |  |
| W_core_E_17 | 2 | AP-type membrane coat adaptor complex | 0.001245 | Starch and sucrose metabolism | 0.006776 |  |  |
| W_core_E_46 | 2 | isomerase activity | 0.000283 | Pyrimidine metabolism | 0.02539 |  |  |
| W_E_20 | 2 |  |  |  |  |  |  |
| W_E_30 | 2 |  |  |  |  |  |  |
| W_E_32 | 2 | gonad morphogenesis | 0.004975 |  |  |  |  |
| W_E_33 | 2 |  |  |  |  |  |  |
| W_E_34 | 2 |  |  |  |  |  |  |
| W_E_4 | 2 | chromosome separation | 0.003584 |  |  |  |  |
| W_E_43 | 2 | morphogenesis of a polarized epithelium | 0.002457 |  |  |  |  |
| W_E_45 | 2 | regulation of protein metabolic process | 0.027063 |  |  |  |  |
| W_E_49 | 2 |  |  |  |  |  |  |
| W_E_50 | 2 |  |  |  |  |  |  |
| W_E_53 | 2 | synapse part | 0.010961 |  |  |  |  |
| W_E_6 | 2 |  |  |  |  |  |  |
| W_E_8 | 2 | intracellular immature spore | 0.000224 |  |  |  |  |
| W_core_E_13 | 2 | regulation of phosphatase activity | 0.003194 |  |  |  |  |
| W_core_E_16 | 2 | regulation of cellular component movement | 3.17E-05 |  |  |  |  |
| W_core_E_21 | 2 |  |  |  |  |  |  |
| W_core_E_27 | 2 | retromer complex | 0.000143 |  |  |  |  |
| W_core_E_34 | 2 |  |  |  |  |  |  |
| W_core_E_37 | 2 | actin filament-based process | 0.003561 |  |  |  |  |
| W_core_E_47 | 2 | organic acid biosynthetic process | 0.039419 |  |  |  |  |
| W_core_E_52 | 2 | negative regulation of protein metabolic process | 0.003094 |  |  |  |  |
| W_core_E_8 | 2 | fluid transport | 0.001038 |  |  |  |  |
| W_core_E_50 | 2 | mitochondrial membrane organization | 3.21E-05 |  |  | mitochondria | 0.03017 |
| W_core_E_41 | 2 | embryonic pattern specification | 0.000624 |  |  | synapse | 0.04884 |
